# Competition between *N*,*C*,*N*-Pincer and *N*,*N*-Chelate Ligands in Platinum(II)

**DOI:** 10.1021/acs.inorgchem.3c00694

**Published:** 2023-06-21

**Authors:** Miguel A. Esteruelas, Sonia Moreno-Blázquez, Montserrat Oliván, Enrique Oñate

**Affiliations:** Departamento de Química Inorgánica, Instituto de Síntesis Química y Catálisis Homogénea (ISQCH), Centro de Innovación en Química Avanzada (ORFEO-CINQA), Universidad de Zaragoza—CSIC, 50009 Zaragoza, Spain

## Abstract

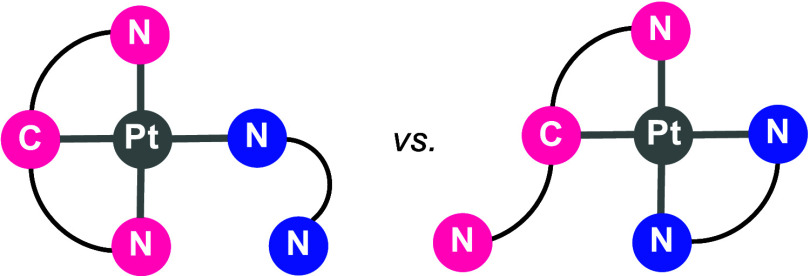

Replacement of the chloride ligand of PtCl{κ^3^-*N*,*C*,*N*-[py-C_6_HR_2_-py]} (R = H (**1**), Me (**2**)) and PtCl{κ^3^-*N*,*C*,*N*-[py-O-C_6_H_3_-O-py]} (**3**) by hydroxido gives Pt(OH){κ^3^-*N*,*C*,*N*-[py-C_6_HR_2_-py]} (R = H (**4**), Me (**5**)) and Pt(OH){κ^3^-*N*,*C*,*N*-[py-O-C_6_H_3_-O-py]} (**6**). These compounds promote deprotonation of 3-(2-pyridyl)pyrazole, 3-(2-pyridyl)-5-methylpyrazole, 3-(2-pyridyl)-5-trifluoromethylpyrazole, and 2-(2-pyridyl)-3,5-bis(trifluoromethyl)pyrrole. The coordination of the anions generates square-planar derivatives, which in solution exist as a unique species or equilibria between isomers. Reactions of **4** and **5** with 3-(2-pyridyl)pyrazole and 3-(2-pyridyl)-5-methylpyrazole provide Pt{κ^3^-*N*,*C*,*N*-[py-C_6_HR_2_-py]}{κ^1^-*N*^1^-[R′pz-py]} (R = H; R′ = H (**7**), Me (**8**). R = Me; R′ = H (**9**), Me (**10**)), displaying κ^1^-*N*^1^-pyridylpyrazolate coordination. A 5-trifluoromethyl substituent causes N^1^-to-N^2^ slide. Thus, 3-(2-pyridyl)-5-trifluoromethylpyrazole affords equilibria between Pt{κ^3^-*N*,*C*,*N*-[py-C_6_HR_2_-py]}{κ^1^-*N*^1^-[CF_3_pz-py]} (R = H (**11a**), Me (**12a**)) and Pt{κ^3^-*N*,*C*,*N*-[py-C_6_HR_2_-py]}{κ^1^-*N*^2^-[CF_3_pz-py]} (R = H (**11b**), Me (**12b**)). 1,3-Bis(2-pyridyloxy)phenyl allows the chelating coordination of the incoming anions. Deprotonations of 3-(2-pyridyl)pyrazole and its substituted 5-methyl counterpart promoted by **6** lead to equilibria between Pt{κ^3^-*N*,*C*,*N*-[pyO-C_6_H_3_-Opy]}{κ^1^-*N*^1^-[R′pz-py]} (R′ = H (**13a**), Me (**14a**)) with a κ-*N*^1^-pyridylpyrazolate anion, keeping the pincer coordination of the di(pyridyloxy)aryl ligand, and Pt{κ^2^-*N*,*C*-[pyO-C_6_H_3_(Opy)]}{κ^2^-*N,N*-[R′pz-py]} (R′ = H (**13c**), Me (**14c**)) with two chelates. Under the same conditions, 3-(2-pyridyl)-5-trifluoromethylpyrazole generates the three possible isomers: Pt{κ^3^-*N*,*C*,*N*-[pyO-C_6_H_3_-Opy]}{κ^1^-*N*^1^-[CF_3_pz-py]} (**15a**), Pt{κ^3^-*N*,*C*,*N*-[pyO-C_6_H_3_-Opy]}{κ^1^-*N*^2^-[CF_3_pz-py]} (**15b**), and Pt{κ^2^-*N*,*C*-[pyO-C_6_H_3_(Opy)]}{κ^2^-*N*,*N*-[CF_3_pz-py]} (**15c**). The N^1^-pyrazolate atom produces a remote stabilizing effect on the chelating form, pyridylpyrazolates being better chelate ligands than pyridylpyrrolates. Accordingly, reactions of **4**–**6** with 2-(2-pyridyl)-3,5-bis(trifluoromethyl)pyrrole yield Pt{κ^3^-*N*,*C*,*N*-[py-C_6_HR_2_-py]}{κ^1^-*N*^1^-[(CF_3_)_2_C_4_(py)HN]} (R = H (**16**), Me (**17**)) or Pt{κ^3^-*N*,*C*,*N*-[pyO-C_6_H_3_-Opy]}{κ^1^-*N*^1^-[(CF_3_)_2_C_4_(py)HN]} (**18**), displaying κ^1^-*N*^1^-pyrrolate coordination. Complexes **7**–**10** are efficient green phosphorescent emitters (488–576 nm). In poly(methyl methacrylate) (PMMA) films and in dichloromethane, they experience self-quenching, due to molecular stacking. Aggregation occurs through aromatic π–π interactions, reinforced by weak platinum–platinum interactions.

## Abstract 2

A study about the competition between the *N*,*C*,*N*-pincer ligands 1,3-bis(2-pyridyl)phenyl and 1,3-bis(2-pyridyloxy)phenyl with the *N*,*N*-chelate ligands pyridylpyrazolate or pyridylpyrrolate in platinum(II) is presented. The study has allowed us to establish coordination priorities between these classes of ligands, isolate complexes with unusual coordination modes, and discover new highly efficient green phosphorescent emitters.

## Introduction

Pincer ligands have risen to prominence in coordination chemistry and organometallics, in the last two decades,^1^ due to the performance of complexes bearing this class of groups in catalysis^2^ and materials science,^3^ among other fields.^4^ Several reasons could be mentioned to explain this fact. The rigid meridional arrangement of the donor atoms of the pincer, in the metal coordination sphere, gives the central ion a remarkable ability to surround itself with uncommon coordination polyhedra and reach unusual oxidation states. As a result, fascinating complexes have been recently discovered. Among them, it is worth highlighting the derivative *mer*-tris(boryl) Ir(Bcat)_3_{κ^3^-*P,O,P*-[xant(P*^i^*Pr_2_)_2_]} (Bcat = catecholboryl; xant(P*^i^*Pr_2_)_2_ = 9,9-dimethyl-4,5-bis(diisopropylphosphino)xanthene), which challenges the concept of trans-influence.^5^ Other families worth mentioning are metalapentalynes and metalapentalenes, which exhibit Möbius planar aromaticity.^6^ In addition, the robustness of this class of complexes, which is a consequence of the strong binding resulting from the tridentate coordination of the pincer, provides them with high thermal stability. This property is highly desirable to stand harsh reaction conditions needed for activating inert bonds in catalytic processes.^7^ It also allows for high-vacuum thermal evaporation, which is a usual procedure for the fabrication of a variety of devices based on transition-metal derivatives.^8^

**Chart 1:**
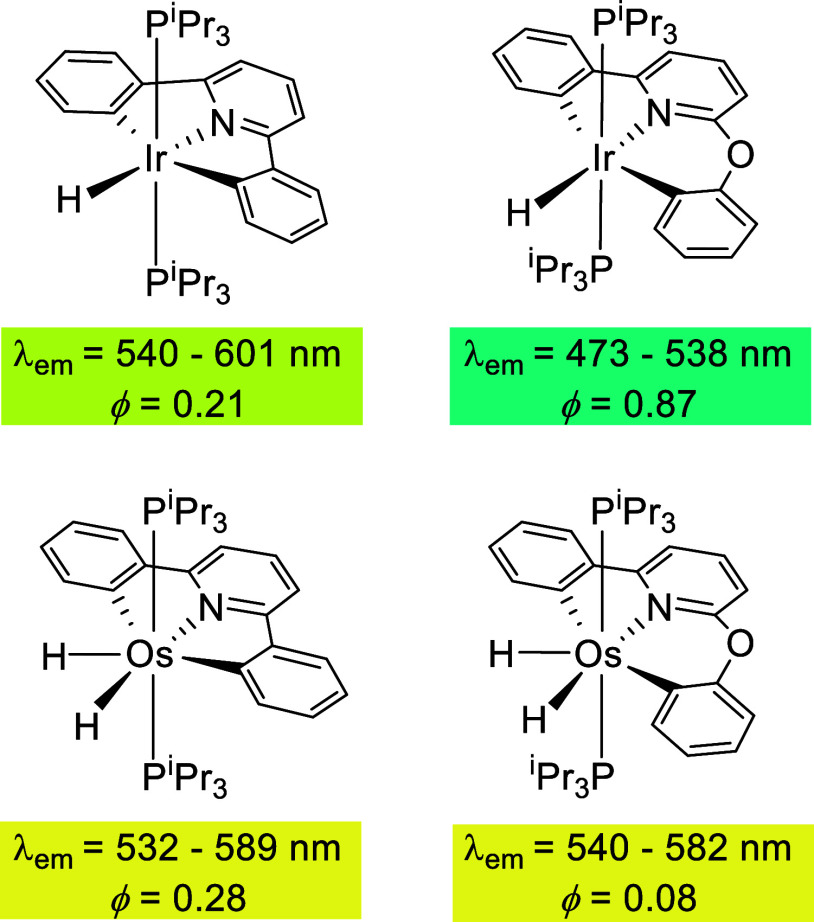
Photophysical Properties of Hydride Complexes Derived from 2,6-Diphenylpyridine and 2-Phenoxy-6-phenylpyridine

The view of complexes carrying pincer ligands is however evolving. In addition to a high thermal stability, species that present a reactivity adapted to the requirements of a certain transformation^9^ or a behavior according to a certain application are sought.^10^ Thus, the concepts of pincer coordinative flexibility and pincer hemilability are emerging in the chemistry of these ligands.^11^ In this context, the fine adjustment of the bite angles results essential to comparatively analyze the influence of their steric and electronic effects on the chemical and physical properties of the complexes^12^ and to establish relationships between the central ion, the coordination polyhedron, the ligand, and the chemical behavior of the complex or a particular property. Reactions of polyhydride complexes IrH_5_(P*^i^*Pr_3_)_2_ and OsH_6_(P*^i^*Pr_3_)_2_ with 2,6-diphenylpyridine and 2-phenoxy-6-phenylpyridine, as well as the chemical behavior and photophysical properties of the resulting hydride derivatives (Chart 1), are a nice example of this.^13^ Their geometries have a marked dependence on the presence of the oxygen atom between the pyridine and a phenyl group. The oxygen atom favors an octahedral arrangement of donor atoms around the d^6^-iridium center, as a consequence of the proximity of the pyridine-Ir-phenoxy angle to the ideal value of 90°. However, it disfavors a pentagonal–bipyramidal arrangement around the seven-coordinate d^4^-osmium center, due to the deviation of the pyridine-Os-phenoxy angle from the ideal value of 72°. The distortions have a dramatic influence on the emissive features of these compounds, which are phosphorescent emitters upon photoexcitation. The presence of the oxygen atom increases the energy of the emission and the efficiency of the emitter in the iridium case, while it ruins the efficiency of the emitter for osmium.^13b^

The *N*,*C*,*N*-ligands occupy a distinguished place among those of this class.^14^ The *N*,*C*,*N*-pincer resulting from the activation of the C–H bond at position 2 of the central ring of 1,3-di(2-pyridyl)benzene was strongly implied in the early stages of the development of this chemistry.^15^ However, in 2004, Williams and co-workers observed that the pyridyl-assisted activation of the C–H bond at position 4 can also occur, generating bidentate coordination instead of the desired pincer.^16^ In the search for blocking such binding mode, the focus later shifted toward the use of analogous substituted at 5-position of the phenyl group^17^ or alternatively at 4 and 6 positions, receiving special attention the pro-ligand 1,3-di(2-pyridyl)-4,6-dimethylbenzene.^18^ Among the complexes supported by these scaffolds, platinum(II) derivatives attract growing interest, partially due to their use for application in photonics, optoelectronics, and medicinal chemistry.^19^ The rigidity of the M(κ^3^-*N*,*C*,*N*) scaffold and the stability of the tridentate bonding are among the main features provided by the *mer*-coordination.^20^ Nevertheless, platinum(II) is no stranger to the new tendencies in pincer chemistry. Exploring systems with greater coordinating flexibility, the use of 1,3-bis(2-pyridyloxy)benzene has been recently introduced. In contrast to 1,3-bis(2-pyridyl)benzene analogues, this pro-ligand generates 2 six-membered fused metallacycles; its chloride–platinum(II) derivative is highly efficient to silylcyanation reactions of aldehydes and imines.^21^

**Chart 2:**
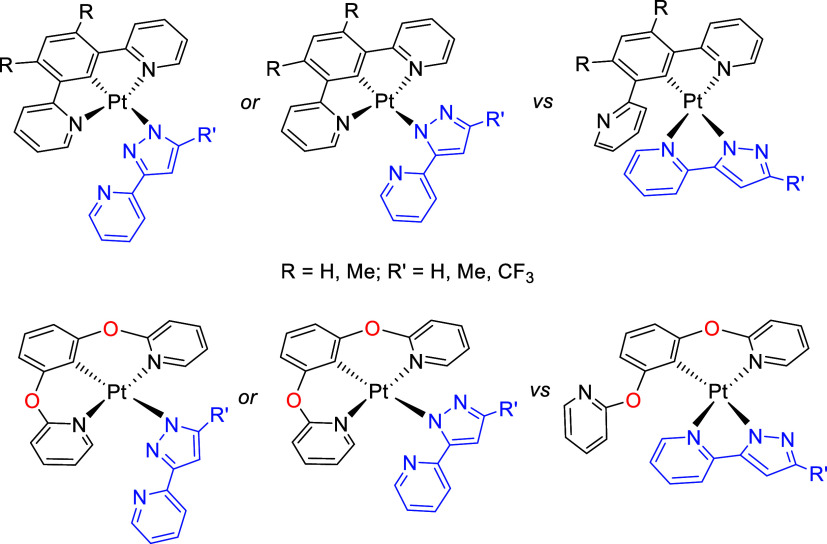
Possible Isomers Resulting from the Dispute in Question

We are interested in developing phosphorescent emitters^8,10^ and catalysts^22^ of 5d platinum group metals with a pincer scaffold. The emitters would be robust with a strong coordination of the pincer, while the coordination capacity of the scaffolding of the catalysts should be flexible, the pincer adapting to the requirements of the reactions. For information in this respect on features of *N*,*C*,*N*-ligands based on a 1,3-bis(2-dipyridyl)aryl skeleton, we have studied the pincer performance of scaffolds 1,3-bis(2-dipyridyl)- and 1,3-bis(2-pyridyloxy)aryl *versus* the chelating ability of 3-(2-pyridyl)pyrazolate in platinum(II) (Chart 2). The 3-(2-pyridyl)pyrazolate anion was selected for the study because the almost exclusive coordination mode of this ligand in mononuclear species is chelate,^23^ while complexes coordinating it as monodentade are very rare.^24^ Although some five-coordinate platinum(II) complexes are known,^25^ platinum(II) was adopted as the central d^8^*-*ion of the resulting compounds. The reason for such selection was that it shows a greater tendency to form square-planar complexes than the d^8^-ions of the other 5d elements of the platinum group, iridium(I) and osmium(0) (Os(0) < Ir(I) < Pt(II)).

This paper reports on the results of the dispute in question, which reveal notable findings.

## Results and Discussion

### Preparation of Platinum(II)-Hydroxide Precursors

**Scheme 1:**
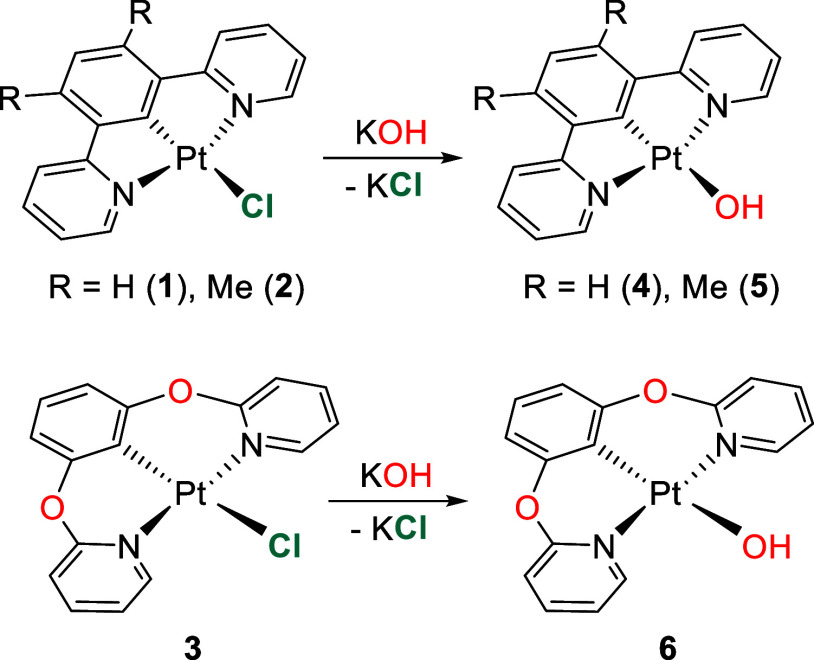
Synthesis of the Platinum(II)-Hydroxide Precursors

Pyrazole molecules include an acidic hydrogen atom,^26^ which manifests its Brønsted character by deprotonation with metal-hydroxides to form pyrazolate derivatives.^27^ Thus, a straightforward procedure to coordinate a 3-(2-pyridyl)pyrazolate anion to platinum(II) seemed to be the deprotonation of 3-(2-pyridyl)pyrazole with platinum(II)-hydroxide precursors; around 20 compounds of this type have been previously characterized.^28^ Accordingly, we decided to replace the chloride ligand of complexes PtCl{κ^3^-*N*,*C*,*N*-[py-C_6_HR_2_-py]} (R = H (**1**), Me (**2**)) and PtCl{κ^3^-*N*,*C*,*N*-[py-O-C_6_H_3_-O-py]} (**3**) by a hydroxide ligand as a previous step, before initiating the study (Scheme 1).

The substitution of the chloride of **1** and **2** by a hydroxide group required more drastic conditions than those used for the substitution of the chloride of **3**. The reason was the lower solubility of the dipyridyl precursors in the reaction solvent, tetrahydrofuran (THF). The exchange between the anions in **1** and **2** was carried out by stirring the respective suspensions with 20 equiv of KOH, at 65 °C, for 48 h, while the substitution in the dipyridyloxy precursor only required 24 h, at room temperature, and an excess of 4 equiv of base. The hydroxide derivatives Pt(OH){κ^3^-*N*,*C*,*N*-[py-C_6_HR_2_-py]} (R = H (**4**), Me (**5**)) and Pt(OH){κ^3^-*N*,*C*,*N*-[py-O-C_6_H_3_-O-py]} (**6**) were isolated as yellow-orange solids in high yields, about 80%. The presence of the hydroxide ligand in the compounds is strongly supported by the ^1^H NMR spectra of **5** and **6** in tetrahydrofuran-*d*_8_, which show a characteristic broad signal at about −0.20 ppm due to the OH-hydrogen atom.

### Dipyridyl-*N*,*C*,*N*-pincers *versus* 3-(2-Pyridyl)pyrazolate Anions

**Scheme 2:**
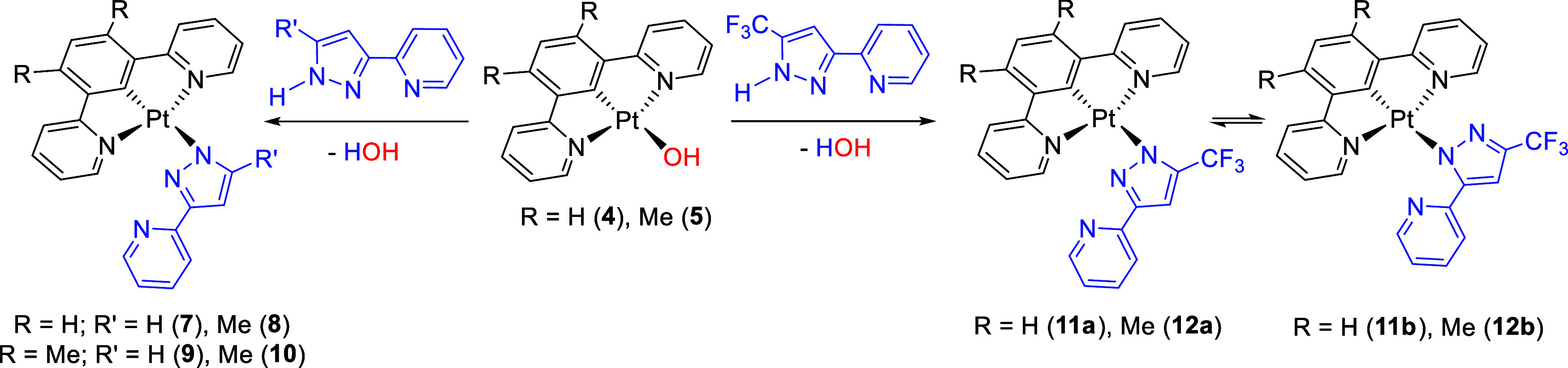
Reactions of Complexes **4** and **5** with 3-(2-Pyridyl)pyrazoles

Hydroxide complexes **4** and **5** promote the abstraction of the acidic hydrogen atom of 3-(2-pyridyl)pyrazole and its 5-methyl and 5-trifluoromethyl substituted analogs, as expected. We reasoned that the presence of a substituent in position 5 of the pyrazolate group should discourage the coordination of the nitrogen atom in position 1, with respect to that in position 2, and therefore favor the chelating bond of the incoming reagent. Although interesting behavioral differences were observed between the pyridylpyrazolate anions, depending on the substituent at position 5, the pincer-to-chelate transformation of the polydentate ligand of the precursors, as a result of chelate coordination of the incoming anions, was not observed (Scheme 2).

**Figure 1:**
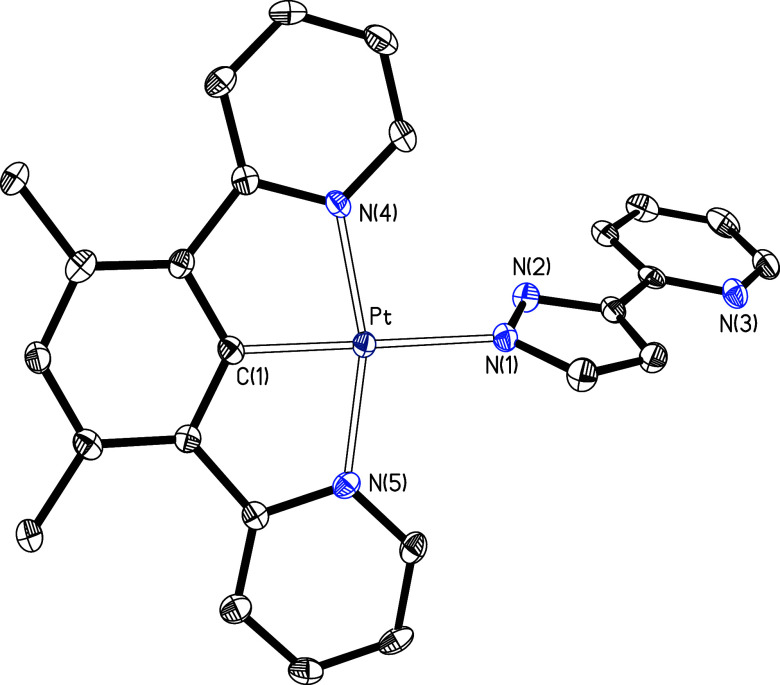
Molecular structure in the crystal of complex **9** (displacement ellipsoids shown at 50% probability). All hydrogen atoms are omitted for clarity. Selected bond distances (Å) and angles (deg): Pt–C(1) = 1.924(3), Pt–N(1) = 2.117(3), Pt–N(4) = 2.023(3), Pt–N(5) = 2.020(3); N(4)–Pt–N(5) = 161.82(11), N(4)–Pt–C(1) = 81.04(12), N(5)–Pt–C(1) = 80.79(13), C(1)–Pt–N(1) = 176.70(12), N(1)–Pt–N(4) = 98.74(10), N(1)–Pt–N(5) = 99.38(11).

3-(2-Pyridyl)pyrazole and its 5-methyl-substituted counterpart provide a single isomer of the two possible ones, resulting from the κ^1^-coordination of the pyrazolate unit of the pyridylpyrazolate anion. Such an isomer is the one corresponding to the coordination of the N-atom in position 1. These complexes, Pt{κ^3^-*N*,*C*,*N*-[py-C_6_HR_2_-py]}{κ^1^-*N*^1^-[R′pz-py]} (R = H; R′ = H (**7**), Me (**8**). R = Me; R′ = H (**9**), Me (**10**)), were isolated as yellow solids in 50–60% yield. The formation of a single isomer is strongly supported by the ^1^H, ^13^C{^1^H}, and ^195^Pt{^1^H} NMR spectra, in dichloromethane-*d*_2_, of the obtained yellow solids. The ^1^H and ^13^C{^1^H} spectra show only one set of signals for each coordinated ligand (Figures S6–S17), whereas the ^195^Pt{^1^H} spectra contain only a singlet between −3558 and −3597 ppm (Table 1). The coordination of the N-atom at position 1 of the pyrazolate group to the platinum(II) ion was confirmed by means of the X-ray diffraction structure of **9**. Figure 1 gives a view of the molecule. The coordination around the metal center is the typical square-planar arrangement for a d^8^ ion, distorted as a consequence of the bite angles of the rigid *N*,*C*,*N*-pincer ligand, which display values strongly deviated from 180° (N(4)–Pt–N(5) = 161.82(11)°) and 90° (N(4)–Pt–C(1) = 81.04(12)° and N(5)–Pt–C(1) = 80.79(13)°). In contrast, the angle C(1)–Pt–N(1), between the central aryl group of the pincer and the pyrazolate of the monodentate anion, of 176.70(12)° approximates to the ideal value of 180°. In spite of it, the coordination of the pyridyl groups of the pincer seems to be significantly stronger than the coordination of the pyrazolate group. This is strongly suggested by the comparison of the platinum–pyridyl distances, Pt–N(4) and Pt–N(5), and the platinum–pyrazolate bond length, Pt–N(1). The former, 2.023(3) and 2.020(3) Å, are about 0.1 Å shorter than the latter, 2.117(3) Å.

**Table 1:** Chemical Shifts of Isomers **a**–**c** of Complexes **7**–**18** in the ^195^Pt{^1^H} NMR Spectra (85.6 MHz)

	^195^Pt{^1^H} chemical shifts (δ)^a^		
complex	isomer **a**	isomer **b**	isomer **c**
**7**	–3597		
**8**	–3579		
**9**	–3567		
**10**	–3558		
**11**	–3584	–3650	
**12**	–3562	–3624	
**13**	–3150		–3220
**14**	–3141		–3211
**15**	–3134	–3210	–3233
**16**		–3554	
**17**		–3537	
**18**		–3163	

a Spectra recorded in dichloromethane-*d*_2_ (chloroform-*d* for **10**) at 298 K.

**Figure 2:**
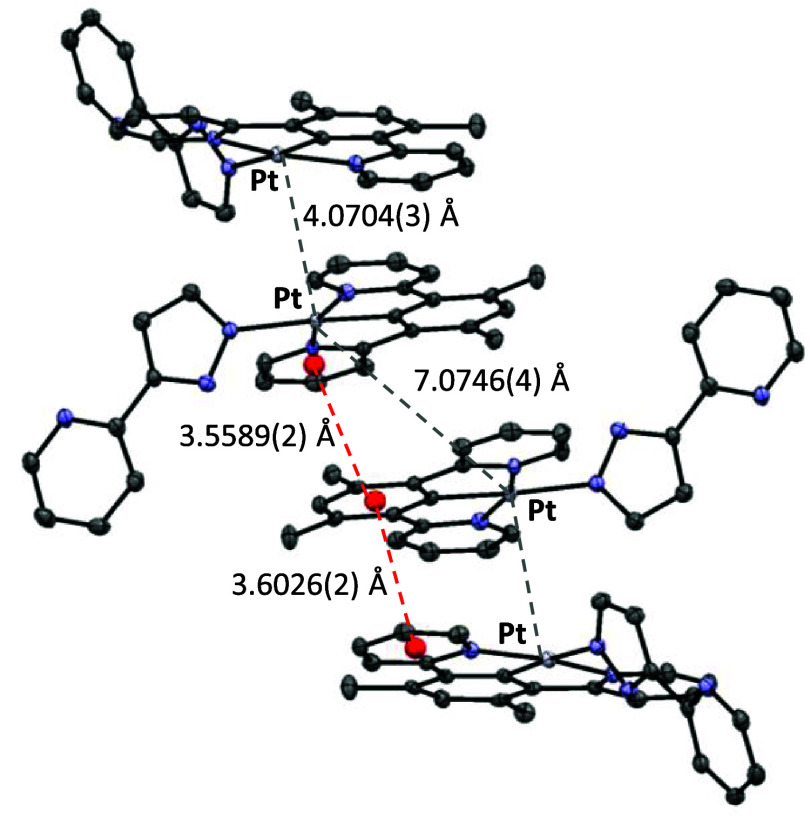
Extended view of the packing for complex **9** showing the intermolecular interactions in the solid state.

A detailed view of the packing reveals that the molecules are stacked as a consequence of the existence of π–π interactions between the aromatic rings of the pincer ligand (Figure 2).^29^ Each interaction involves three rings of three different molecules: the aryl linker from the pincer of one molecule and a pyridyl group of the pincer of the molecules above and below. To explain the stacking sequence, molecules can be grouped into pairs that interact with each other. The connection between the molecules of the pairs is reinforced by weak platinum–platinum interactions, which was confirmed by using an AIM approach (Figure S61). Thus, two parameters define the interaction within each pair and between pairs: the centroid–centroid separation of the rings involved in the interaction and the distance between the metal centers. Within each pair, the centroid–centroid separations are 3.6026(2) Å, whereas the platinum–platinum distance has a value of 4.0704(3) Å. The interaction between pairs occurs with shorter centroid–centroid separations of 3.5589(2) Å; however, the metal–metal separation increases up to 7.0746(4) Å. The rings involved in each interaction show a slightly offset conformation, with slips between them in the range of 1.099–1.329 Å.

**Figure 3:**
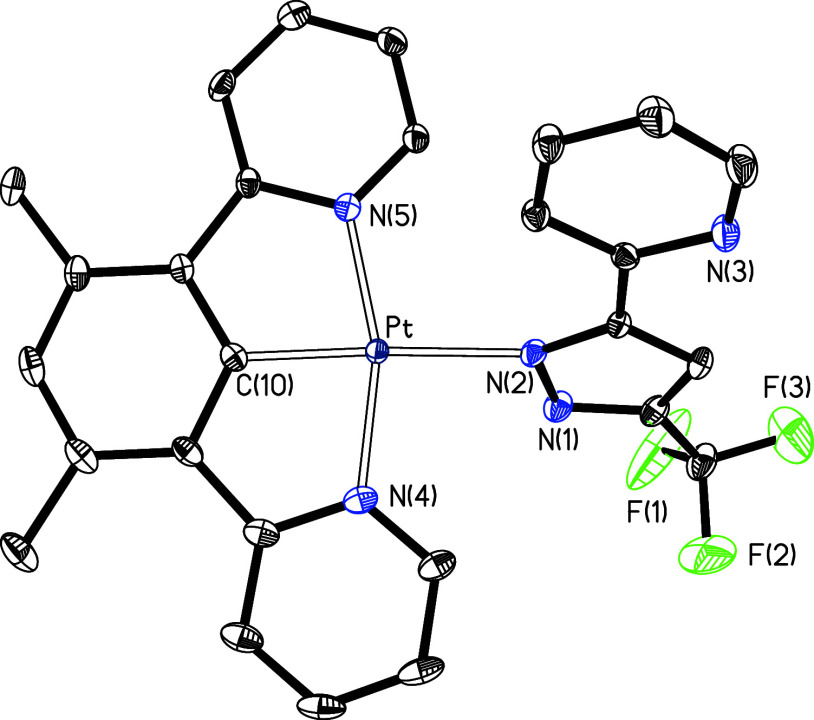
Molecular structure in the crystal of complex **12b** (displacement ellipsoids shown at 50% probability). All hydrogen atoms are omitted for clarity. Selected bond distances (Å) and angles (deg): Pt–C(10) = 1.918(3), Pt–N(2) = 2.123(2), Pt–N(4) = 2.019(2), Pt–N(5) = 2.015(2); N(4)–Pt–N(5) = 162.24(9), N(4)–Pt–C(10) = 81.13(10), N(5)–Pt–C(10) = 81.12(10), C(10)–Pt–N(2) = 174.64(9), N(2)–Pt–N(4) = 96.69(9), N(2)–Pt–N(5) = 101.06(9).

5-Trifluoromethyl substituent reduces the coordination selectivity of the pyridylpyrazolate anion. Unlike the 5-methyl counterpart, 3-(2-pyridyl)-5-trifluoromethylpyrazole reacts with **4** and **5** to give yellow solids in about 60% yield. In dichloromethane, the solids provide the two possible isomers, Pt{κ^3^-*N*,*C*,*N*-[py-C_6_HR_2_-py]}{κ^1^-*N*^1^-[CF_3_pz-py]} (R = H (**11a**), Me (**12a**)) and Pt{κ^3^-*N*,*C*,*N*-[py-C_6_HR_2_-py]}{κ^1^-*N*^2^-[CF_3_pz-py]} (R = H (**11b**), Me (**12b**)), which can be generated as a consequence of the κ^1^-coordination of the pyrazolyl group. The unprecedented κ^1^-N^2^-coordination in **11b** and **12b** was confirmed by the X-ray structure of **12b**. Single crystals suitable for diffraction analysis were obtained from a solution of the solid, in dichloromethane, by vapor diffusion of pentane. The structure (Figure 3) resembles that of **9** with the pyridylpyrazolate anion coordinated by N(2) instead of N(1). The bond lengths and angles involving the pincer and the metal are nearly equal to **9**, while the distance between the metal and the coordinated pyrazolate nitrogen atom, N(2), is approximately 0.01 Å longer than in **9** (compare captions of Figures 1 and 3). This suggests that the coordination of the pyridylpyrazolate anion through the nitrogen atom at position 2 is even slightly weaker than through the nitrogen atom at position 1. In agreement with this, density functional theory (DFT) calculations about the isomerization of **12a** into **12b** (B3LYP(G)//SDD(f)/6-31Gg**), in chloroform, at 298 K revealed that isomer **12a** is 3.6 kcal·mol^–1^ more stable than **12b**. The isomerization takes place *via* a five coordination transition state, bearing the pyrazolate unit of the pyridylpyrazolate ligand κ^2^-N,N-coordinated. This transition state lies 13.4 kcal·mol^–1^ above **12b** (Figure S62). Despite the structural similarities between **12b** and **9**, a packing analogous to that shown in Figure 2 is not observed in the case of **12b**; most likely, the bigger obstacle of trifluoromethyl with respect to methyl prevents the approach of the molecules.

The ^1^H, ^13^C{^1^H}, ^19^F{^1^H}, and ^195^Pt{^1^H} NMR spectra, in dichloromethane-*d*_2_ or chloroform-*d*, of the yellow solids are consistent with the presence of isomers **a** and **b** in both **11** and **12** (Figures S18–S27). The ^1^H and ^13^C{^1^H} spectra contain two sets of resonances for each coordinated ligand (one per isomer), while ^19^F{^1^H} and ^195^Pt{^1^H} show two singlets between −57 and −61 ppm and between −3580 and −3660 ppm, respectively. Furthermore, they indicate that the isomers are in equilibrium. The intensity of the signals, and therefore the molar ratio between the isomers, depends on the temperature of the sample. Equilibria in chloroform-*d* or dichloromethane-*d*_2_, between 333 and 223 K, were studied by ^19^F{^1^H} (Figures S53, S55, S57, and S59). The equilibrium constants *K*_11_ and *K*_12_, at each temperature, for the isomerization of the κ^1^-N^2^-coordinated species (**b**) into the κ^1^-N^1^-coordinated derivatives (**a**) were determined by integrating the signals assigned to each one of them, while the analysis of the line shape of the signals allowed the calculation of the rate constants *k*_11_ and *k*_12_. Table 2 gives the obtained values. The temperature dependence of the equilibrium constants (Figures S54 and S56) provides values for Δ*H*°, Δ*S*°, and Δ*G*_298_° of 0.7 ± 0.2 kcal·mol^–1^, 3.2 ± 1.0 cal·K^–1^·mol^–1^, and −0.3 ± 0.1 kcal·mol^–1^ for **11** and 0.6 ± 0.2 kcal·mol^–1^, 2.6 ± 1.0 cal·K^–1^·mol^–1^, and −0.2 ± 0.1 kcal·mol^–1^ for **12**, respectively, which thermodynamically characterize the equilibria. Similarly, the kinetic analysis resulting from the respective Eyring plots (Figures S58 and S60) yields values for the activation parameters Δ*H*^⧧^, Δ*S*^⧧^, and Δ*G*_298_^⧧^ of 15.2 ± 0.8 kcal·mol^–1^, −0.4 ± 1.6 cal·K^–1^·mol^–1^, and 15.3 ± 1.3 kcal·mol^–1^ for **11** and 16.2 ± 1.3 kcal·mol^–1^, 2.6 ± 2.5 cal·K^–1^·mol^–1^, and 15.4 ± 2.0 kcal·mol^–1^ for **12**, respectively. Both Δ*G*_298_° and Δ*G*_298_^⧧^ satisfactorily agree with the values obtained by DFT calculations for **12**. Furthermore, their similarity suggests that the presence of two methyl substituents in positions 4 and 6 of the aryl linker does not have any significant influence on the behavior of these pincer complexes.

**Table 2:** Equilibrium Constants *K*_11_ and *K*_12_, and Rate Constants *k*_11_ and *k*_12_ for the Equilibria between Isomers **a** and **b** of Complexes **11** and **12**^c^

	complex **11**		complex **12**	
*T* (K)	*K*_11_^a^	*k*_11_ (s^–1^)^b^	*K*_12_^a^	*k*_12_ (s^–1^)^b^
333		548.62		521.84
328				354.82
323		255.76		
318				161.80
313		116.20		
308		79.88		70.87
298	1.66	30.24	1.28	25.28
280		5.95		
273	1.41		1.20	
263	1.37		1.13	
253	1.31		1.08	
243	1.24		1.02	
233	1.18		0.97	
223	1.12		0.91	

a In dichloromethane-*d*_2_.

b In chloroform-*d*.

c *K*_11_ = [**11a**]/[**11b**]; *K*_12_ = [**12a**]/[**12b**].

### 1,3-Bis(2-pyridyloxy)phenyl *versus* 3-(2-Pyridyl)pyrazolates

**Scheme 3:**
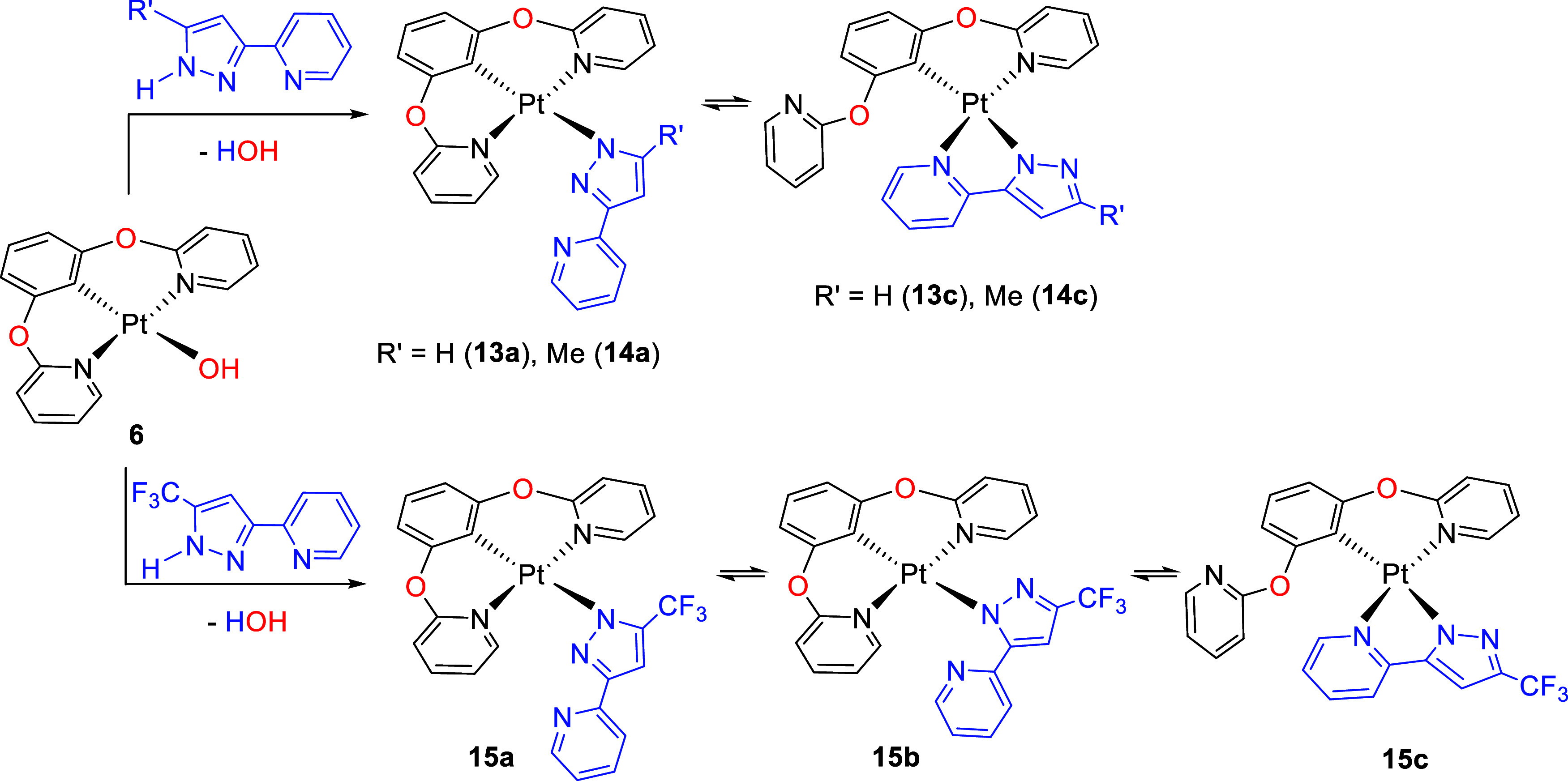
Reactions of Complex **6** with 3-(2-Pyridyl)pyrazoles

The hydroxide ligand of complex **6** also promotes the abstraction of the acidic hydrogen atom of 3-(2-pyridyl)pyrazole and its 5-methyl and 5-trifluoromethyl substituted analogues. Surprisingly, however, the isolated complexes **13**–**15** (Scheme 3) exist in dichloromethane or chloroform solutions as combinations of several pyrazolate coordination isomers, which result from pincer *versus* chelate competition between the ligands and from possible κ^1^-N^1^ or κ^1^-N^2^ coordination of the pyridylpyrazolate anions. Although from a geometric point of view the pyridyloxy group should favor square-planar coordination, since it opens the bite angles of the pincer, bringing them closer to their ideal values of 90 and 180°,^21^ it seems to electronically destabilize tridentate coordination. Thus, 3-(2-pyridyl)pyrazole and its substituted 5-methyl counterpart provide Pt{κ^3^-*N*,*C*,*N*-[pyO-C_6_H_3_-Opy]}{κ^1^-*N*^1^-[R′pz-py]} (R′ = H (**13a**), Me (**14a**)) with a κ-N^1^-pyridylpyrazolate anion, keeping the pincer coordination of the di(pyridyloxy)aryl ligand, and Pt{κ^2^-*N*,*C*-[pyO-C_6_H_3_(Opy)]}{κ^2^-*N*,*N*-[R′pz-py]} (R′ = H (**13c**), Me (**14c**)) with two chelates. At 298 K, the **a**/**c** molar ratios are 1:0.6 for **13** and 1:0.5 for **14**. Consistent with the previously noted ability of the 5-trifluoromethyl substituent to cause the N^1^-to-N^2^ sliding, 5-trifluoromethylpyrazole generates the three possible isomers Pt{κ^3^-*N*,*C*,*N*-[pyO-C_6_H_3_-Opy]}{κ^1^-*N*^1^-[CF_3_pz-py]} (**15a**), Pt{κ^3^-*N*,*C*,*N*-[pyO-C_6_H_3_-Opy]}{κ^1^-*N*^2^-[CF_3_pz-py]} (**15b**), and Pt{κ^2^-*N*,*C*-[pyO-C_6_H_3_(Opy)]}{κ^2^-*N*,*N*-[CF_3_pz-py]} (**15c**), in an **a**/**b**/**c** molar ratio of 0.25:0.20:1 at 298 K.

**Figure 4:**
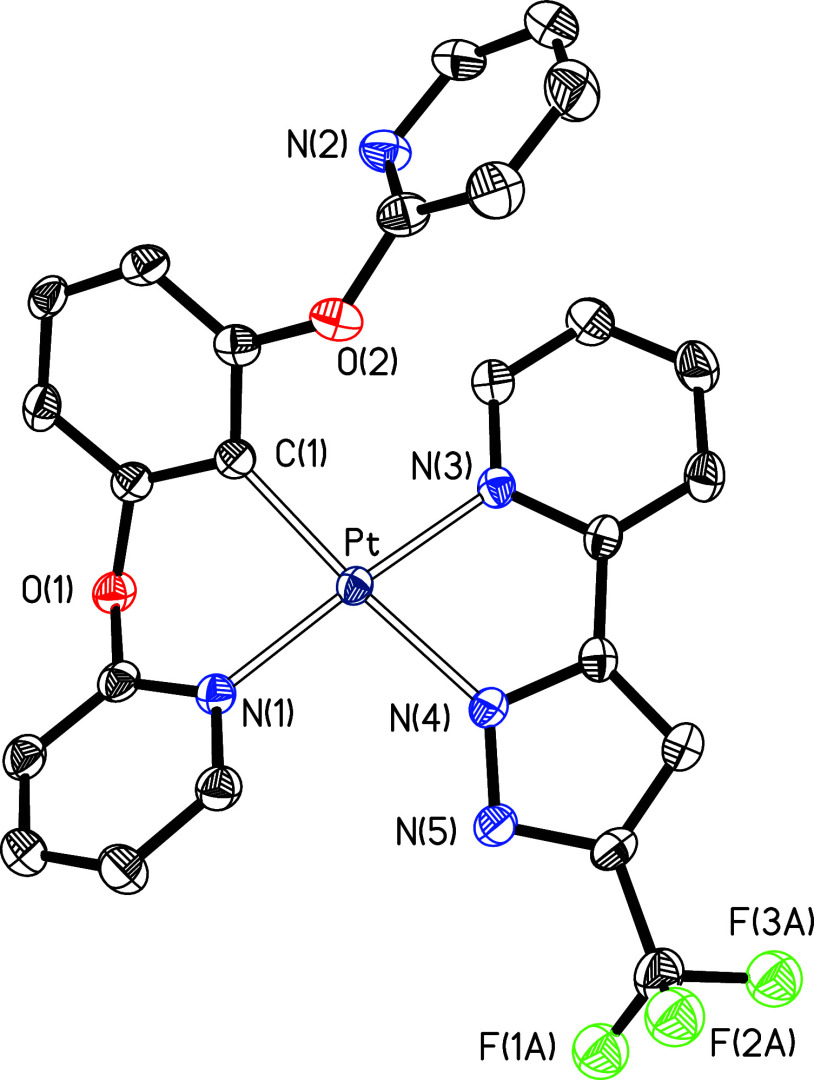
Molecular structure in the crystal of complex **15c** (displacement ellipsoids shown at 50% probability). All hydrogen atoms are omitted for clarity. Selected bond distances (Å) and angles (deg): Pt–C(1) = 2.005(4), Pt–N(1) = 2.020(3), Pt–N(3) = 2.030(3), Pt–N(4) = 2.069(4); C(1)–Pt–N(1) = 85.51(16), N(3)–Pt–N(4) = 78.82(14), C(1)–Pt–N(4) = 174.62(16), N(1)–Pt–N(3) = 176.61(14), C(1)–Pt–N(3) = 97.54(16), N(1)–Pt–N(4) = 98.23(14).

Complexes **13**–**15** were isolated as white solids in approximately 60% yield. The presence of isomers **c**, which coordinate two chelates, was confirmed by the X-ray diffractometric analysis on **15c**. A solution of **15** in dichloromethane provided single crystals suitable for diffraction analysis, by pentane vapor diffusion, at 4 °C. The structure (Figure 4) proves the transformation from *N*,*C*,*N*-pincer to *C*,*N*-chelate of the di(pyridyloxy)aryl ligand (N(1)–Pt–C(1) = 85.51(16)°) and the chelating coordination of the incoming pyridylpyrazolate anion (N(3)–Pt–N(4) = 78.82(14)°). Thus, the geometry around the metal center can be described as square planar with a trans arrangement C(1)–to–N(4) (C(1)–Pt–N(4) = 174.62(16)°). The platinum–pyridyl distances of 2.020(3) (Pt–N(1)) and 2.030(3) (Pt–N(3)) Å are similar to the platinum–pyridyl bond lengths in **9** and **12b**, while platinum–pyrazolate distance Pt–N(4) of 2.069(4) Å is about 0.06 A shorter than that in **12b**. Although small, the difference indicates that the chelate coordination of the pyridylpyrazolate anion increases the strength of the Pt–N^2^ bond, as expected.

The isomers are in equilibrium as supported by the ^1^H, ^13^C{^1^H}, and ^195^Pt{^1^H} NMR spectra of **13–15** and the ^19^F{^1^H} NMR spectrum of **15**, in dichloromethane-*d*_2_ or chloroform-*d*, which show resonances with intensities that are a function of temperature. The most notable feature of the ^1^H and ^13^C{^1^H} spectra (Figures S28–S30, S32–S34, and S36–S38) is the presence of sets of resonances corresponding to different situations involving equivalent pyridyloxy groups, assigned to isomers **a** and **b**, and inequivalent ones due to isomers **c**. The ^195^Pt{^1^H} spectra (Figures S31, S35, and S40) are also consistent with the presence of two or three different isomers in solution and further reveal that the signals shift to a higher field according to the sequence **a** < **b** < **c** (Table 1). Although a quantitative analysis of the equilibria was not possible due to the complexity of the ^1^H spectra and the slowness with which they are reached, mainly at temperatures below 263 K, some qualitative conclusions can be inferred from the spectra. Their comparative analysis reveals that the trifluoromethyl substituent favors the chelating coordination of the pyridylpyrazolate anion, as a consequence of its ability to promote the coordination of the N^2^ atom. Thus, while the isomer **a** is the main component of **13** and **14**, the isomer **c** is the major complex of **15**, and only a small amount of **b** is present in the latter.

### Relevance of the N^1^ Atom of the Pyrazolate Unit in the Chelating Coordination of the Pyridylpyrazolate Ligands: Reactions with 2-(2-Pyridyl)-3,5-bis(trifluoromethyl)pyrrole

**Scheme 4:**
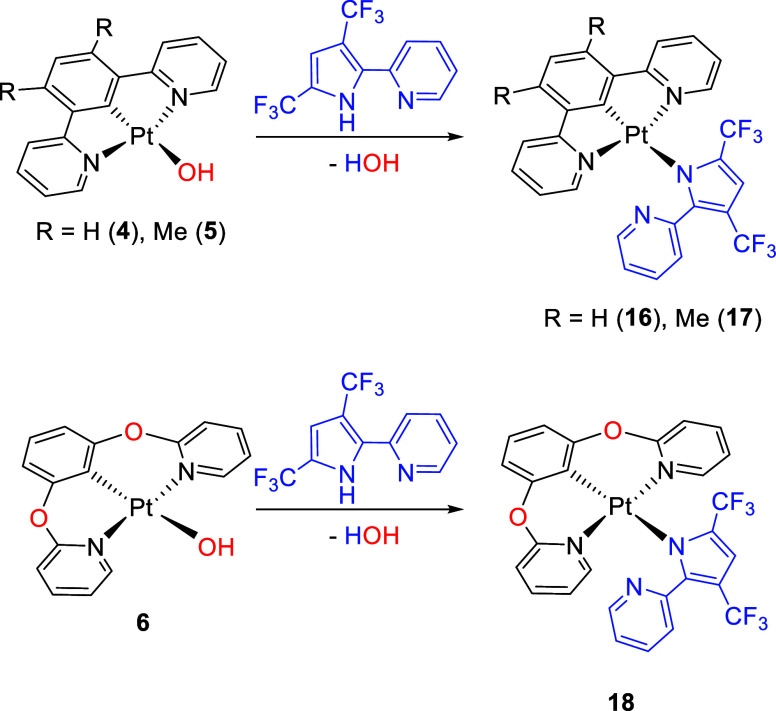
Reactions with 2-(2-Pyridyl)-3,5-bis(trifluoromethyl)pyrrole

To avoid the formation of **a**-type isomers, we decided to remove the N atom at position 1 of the pyrazole. We reasoned that the use of a 2-pyridylpyrrolate anion should also allow us to know the remote influence of the N^1^-pyrazolate atom on the equilibria between type **b** and **c** isomers. In addition, we introduced two trifluoromethyl substituents at positions 3 and 5 of the five-membered ring, which in principle should favor chelating coordination of the incoming anion, since the isomer **c** is by far the most abundant among the isomers of **15**. Reactions of hydroxido complexes **4**–**6** with 2-(2-pyridyl)-3,5-bis(trifluoromethyl)pyrrole afford yellow solids in a moderated yield of about 50%. Their ^1^H, ^13^C{^1^H}, ^19^F{^1^H}, and ^195^Pt{^1^H} NMR spectra in dichloromethane-*d*_2_ (Figures S41–S52) reveal that they correspond to **b**-type isomers of formula Pt{κ^3^-*N*,*C*,*N*-[py-C_6_HR_2_-py]}{κ^1^-*N*^1^-[(CF_3_)_2_C_4_(py)HN]} (R = H (**16**), Me (**17**)) or Pt{κ^3^-*N*,*C*,*N*-[pyO-C_6_H_3_-Opy]}{κ^1^-*N*^1^-[(CF_3_)_2_C_4_(py)HN]} (**18**). Thus, the spectra contain only one set of signals for each coordinated ligand, in particular ^1^H and ^13^C{^1^H} spectra indicate that the pyridyl groups of the tridentate ligand are equivalent. Because no chelating ability of the incoming anion is observed in any case, even in competition with the *N*,*C*,*N*-pincer di(pyridyloxy)aryl ligand of **18**, it should be pointed out that the isolated complexes according to Scheme 4 support a surprisingly remarkable remote influence of the N atom, at position 1 of the pyrazolate group, of 2-pyridylpyrazolate anions, on the coordination capacity of the pyridyl group. The reason for this effect could be an additional stabilization of the diheterometallacycle, resulting from the chelating coordination of the anion, as a consequence of the extension of the π-system that allows the delocalization of the free electron pair on the N^1^ atom. In this context, it should be mentioned that despite the fact that the diheterometallacycle generated from the chelating coordination of the 2-pyridylpyrrolate anion does not undergo such additional stabilization, the chelating coordination of this anion is almost the only one observed. Monodentate coordination, as in **16**–**18**, has only been previously observed in one case, among the 2-pyridylpyrrolate transition-metal complexes characterized by X-ray diffraction analysis. The anion of such a compound reduces the coordination ability of its pyridyl group by steric hindrance from a phenyl group attached to the carbon arranged ortho to the heteroatom.^30^

**Figure 5:**
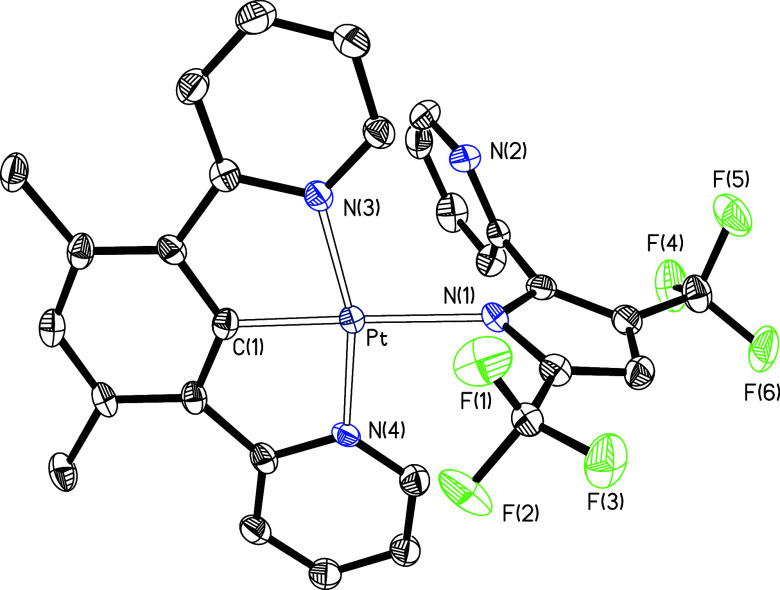
Molecular structure in the crystal of complex **17** (displacement ellipsoids shown at 50% probability). All hydrogen atoms are omitted for clarity. Selected bond distances (Å) and angles (deg): Pt–C(1) = 1.921(4), Pt–N(3) = 2.019(4), Pt–N(4) = 2.022(3), Pt–N(1) = 2.126(4); C(1)–Pt–N(3) = 81.58(16), C(1)–Pt–N(4) = 81.50(16), N(3)–Pt–N(4) = 162.89(14), N(1)–Pt–C(1) = 176.64(15), N(1)–Pt–N(3) = 100.87(14), N(1)–Pt–N(4) = 95.94(14).

**Figure 6:**
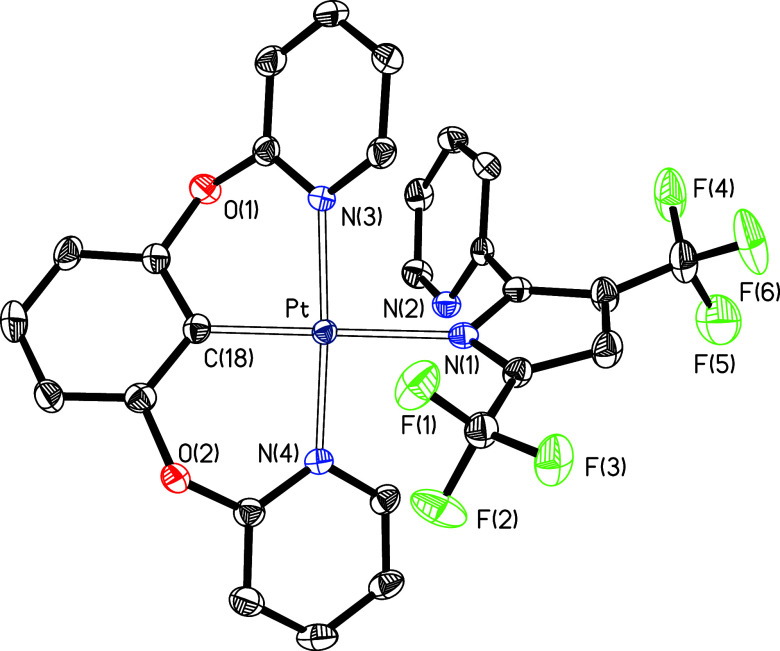
Molecular structure in the crystal of complex **18** (displacement ellipsoids shown at 50% probability). All hydrogen atoms are omitted for clarity. Selected bond distances (Å) and angles (deg): Pt–C(18) = 1.962(2), Pt–N(3) = 2.027(3), Pt–N(4) = 2.008(3), Pt–N(1) = 2.112(3); C(18)–Pt–N(3) = 88.04(13), C(18)–Pt–N(4) = 87.98(13), N(3)–Pt–N(4) = 175.36(11), N(1)–Pt–C(18) = 178.95(13), N(1)–Pt–N(3) = 91.80(11), N(1)–Pt–N(4) = 92.22(11).

The κ^1^-N coordination of the pyrrolate group of the incoming anion of **16**–**18** was confirmed by the X-ray diffractometric analysis on single crystals of **17** and **18**. In addition, these structures allow us to analyze the effect produced by the introduction of an oxygen atom, between the aryl and pyridyl groups of the pincer, on the geometric parameters of these systems. Figures 5 and 6 give views of the respective molecules. In both cases, the coordination around the platinum(II) ion is the expected square-planar arrangement with the pyrrolate group disposed trans to the pincer carbon atom (N(1)–Pt–C(1) = 176.64(15)° for **17** and 178.95(13)° for **18**). However, complex **17** presents a more distorted coordination than **18**, as a consequence of the differences in the bite angles. According to the previous structures, the di(pyridyl)aryl pincer coordinates with angles that deviate from the ideal values of 90 and 180° more than the di(pyridyloxy)aryl ligand (162.89(14) *versus* 175.36(11)° (N(3)–Pt–N(4)), 81.58(16) *versus* 88.04(13)° (N(3)–Pt–C(1)), and 81.50(16) *versus* 87.98(13)° (N(4)–Pt–C(1))). Despite this, the lengths of the platinum–nitrogen and platinum–carbon bonds are very similar in both compounds, suggesting similar stability for the coordination of both pincers. The oxygen atoms between the phenyl and pyridyl groups allow for more comfortable coordination of the pincer but prevent delocalization of electrons within the heterometallacycle. Such electron delocalization, which is only possible in di(pyridyl)aryl pincers, gives rise to some degree of aromaticity, implying further stabilization with respect to the heterometallacycles of the di(pyridyloxy)aryl ligand. This stability due to resonance compensates for that resulting from a more comfortable coordination, even exceeding it by far, as is evident from the comparison of Schemes 2 and 3.

### Photophysical and Electrochemical Properties of **7**–**10** and **16**–**18**

The square-planar platinum(II) d^8^-complexes constitute one of the noble families of phosphorescent complexes,^3d,31^ which ranks on the same level of importance as the iridium(III) and osmium(II) d^6^-emitters.^32^ This along with the novelty of the κ^1^-coordination of the incoming anions led us to study the absorption and emission characteristics of the discovered compounds that exist in solution as a single isomer.

Figures S63–S69 provide UV–vis spectra of 10^–5^ M solutions of **7**–**10** and **16**–**18** in dichloromethane at room temperature, whereas Table 3 lists selected absorptions. The spectra of the seven compounds are very similar. They display bands with intensities that depend on the spectral region in which they are found. Very intense absorptions are observed below 300 nm (ε ≈ 85 000–40 000 M^–1^·cm^–1^), while in the intermediate region between 330 and around 400–450 nm, intense bands appear (ε ≈ 17 000–8000 M^–1^·cm^–1^). Much fainter bands can also be discerned at energies below 450 nm (ε ≈ 200–800 M^–1^·cm^–1^). Molecular stacking as the one shown in Figure 2 does not occur under spectral measurement conditions. Accordingly, the spectra of **9** displayed good agreement with Beer’s law in the concentration range of (5.56 × 10^–6^)–(1.00 × 10^–4^) M at 390 nm (Figure S70). Spectra were calculated and bands were assigned based on DFT (TD-DFT) calculations (B3LYP-D3//SDD(f)/6-31G**) in dichloromethane. Figures S71–S77 represent the most relevant orbitals, and Tables S8–S14 summarize the fragments involved in said orbitals. The bands correspond to transitions into states with metal-to-pincer charge-transfer character combined with transitions into states with intra/interligand charge-transfer character involving the monodentate group and the pincer. The tails after 450 nm imply formal spin-forbidden transitions, which are caused by large spin–orbit coupling resulting from the presence of platinum.

**Table 3:** Selected Calculated (TD-DFT in CH_2_Cl_2_) and Experimental UV–Vis Absorptions for **7**–**10** and **16**–**18** (in CH_2_Cl_2_) and Their Major Contributions

λ exp (nm)	ε (M^–1^·cm^–1^)	exc. energy (nm)	oscillator strength, *f*	excited state character
Complex **7**				
260	65 600	256	0.1992	HOMO – 3 → LUMO + 3 (63%)
384	15 600	400	0.0613	HOMO → LUMO + 1 (93%)
472	200	471 (T_1_)	0	HOMO – 3 → LUMO + 1 (25%)
				HOMO – 1 → LUMO (22%)
				HOMO → LUMO (34%)
Complex **8**				
262	41 700	262	0.0407	HOMO – 3 → LUMO + 2 (61%)
387	10 100	409	0.0266	HOMO → LUMO + 1 (96%)
472	500	470 (T_1_)	0	HOMO – 3 → LUMO + 1 (26%)
				HOMO – 2 → LUMO (25%)
				HOMO → LUMO (25%)
Complex **9**				
267	82 800	258	0.1783	HOMO – 1 → LUMO + 4 (66%)
390	14 500	391	0.0716	HOMO → LUMO + 1 (92%)
471	800	475 (T_1_)	0	HOMO – 2 → LUMO + 1 (30%)
				HOMO – 1 → LUMO (26%)
				HOMO → LUMO (28%)
Complex **10**				
265	52700	264	0.4185	HOMO – 3 → LUMO + 3 (78%)
390	10500	397	0.0329	HOMO → LUMO + 1 (95%)
477	600	474 (T_1_)	0	HOMO – 3 → LUMO + 1 (34%)
				HOMO – 1 → LUMO (25%)
				HOMO → LUMO (17%)
Complex **16**				
265	47700	256	0.1088	HOMO – 3 → LUMO + 4 (73%)
383	13430	375	0.0973	HOMO – 1 → LUMO + 1 (52%)
				HOMO → LUMO + 1 (34%)
463	300	464 (T_1_)	0	HOMO – 4 → LUMO + 1 (19%)
				HOMO – 1 → LUMO (41%)
Complex **17**				
264	47800	255	0.0434	HOMO – 1 → LUMO + 5 (63%)
389	11300	355	0.0142	HOMO → LUMO + 1 (70%)
450	500	452 (T_1_)	0	HOMO – 3 → LUMO + 1 (41%)
				HOMO – 2 → LUMO (20%)
Complex **18**				
289	4610	290	0.0386	HOMO – 3 → LUMO + 1 (81%)
345	850	345	0.0209	HOMO → LUMO (85%)
372	330	371 (T_1_)	0	HOMO – 1 → LUMO + 2 (11%)
				HOMO → LUMO + 2 (56%)

The HOMO is mainly centered on the monodentate group (72–85% for **7**–**10** and 66–92% for **16**–**18**), with significant contributions of the metal center (10–14% for **7**–**10** and 5–22% for **16**–**18**) and the pincer (6–12% for **7**–**10** and 3–15% for **16**–**18**). On the contrary, the LUMO is delocalized on pincer (≈90%), and partially on the metal center (≈10%). In order to obtain experimental information about these occupied and unoccupied states, the redox properties of the complexes were also evaluated by cyclic voltammetry. The oxidation and reduction potentials were measured under an atmosphere of argon, in dichloromethane, using [Bu_4_N]PF_6_ as supporting electrolyte (0.1 M). Figures S78–S84 show the voltammograms. Their patterns are relatively the same with small changes in redox potentials. Table 4 lists the potential values *versus* Fc/Fc^+^. The seven complexes show two irreversible oxidation processes, between 0.01 and 1.43 V, and two irreversible reduction waves, between −1.57 and −2.12 V for **7**–**10** and between −0.98 and −1.91 V for **16**–**18**. Table 4 also collects the HOMO energy levels estimated from the potentials of the first oxidation and the LUMO energy levels estimated from both the potentials of the first reduction and the optical gap obtained from the onset of emission (*E*_00_), as well as the HOMO and LUMO energy levels calculated by DFT. There is a relatively good agreement between the HOMO energy levels estimated from the experimental potential values and those calculated by DFT. However, three significantly different LUMO energy level values are obtained depending on the method used for the calculation.

**Table 4:** Electrochemical and DFT Molecular Orbitals Energy Data for **7**–**10** and **16**–**18**

			obs (eV)			calcd (eV)	
complex	*E*_ox_ (V)^a^	*E*_red_ (V)	HOMO/LUMO^b^	*E*_00_^c^	LUMO from *E*_00_	HOMO/LUMO	HLG^d^
**7**	0.39, 1.43	–1.57, −2.11	–5.19/–3.23	2.61	–2.58	–5.38/–1.81	3.57
**8**	0.76, 1.34	–1.58, −2.07	–5.56/–3.22	2.59	–2.97	–5.43/–1.77	3.66
**9**	0.40, 1.25	–1.58, −2.09	–5.20/–3.22	2.60	–2.60	–5.37/–1.77	3.60
**10**	0.72, 1.34	–1.73, −2.12	–5.52/–3.07	2.61	–2.91	–5.42/–1.73	3.69
**16**	0.26, 1.18	–0.99, −1.60	–5.06/–3.81	2.62	–2.44	–5.61/–1.76	3.85
**17**	0.20, 1.19	–0.98, −1.60	–5.00/–3.82	2.59	–2.41	–5.64/–1.66	3.98
**18**	0.01, 1.40	–1.14, −1.91	–4.81/–3.63	2.68	–2.13	–5.69/–1.41	4.28

a Measured under argon in dichloromethane/[Bu_4_N]PF_6_ (0.1 M), *versus* Fc/Fc^+^.

b HOMO = −[*E*_ox_*versus* Fc/Fc^+^ + 4.8] eV; LUMO = −[*E*_red_*versus* Fc/Fc^+^ + 4.8] eV.

c *E*_00_ = onset of emission.

d HLG = LUMO – HOMO.

Pyridylpyrazolate complexes **7**–**10** are among the most efficient green phosphorescent emitters of platinum(II) (488–576 nm) described so far. The study of the emission, obtained by photoexcitation, was carried out on doped poly(methyl methacrylate) (PMMA) films at 298 K, dichloromethane at 298 K, and frozen matrices of dichloromethane at 77 K. Table 5 summarizes the most relevant findings. The green photoluminescence stems from the respective T_1_ excited states. This origin is supported by the excellent agreement between the wavelengths of the emission maxima in dichloromethane and the calculated values for the energy differences between the optimized T_1_ triplet states and the S_0_ singlet states in the same solvent. In all cases, the bands appear highly structured, as expected for a significant contribution from ligand-centered ^3^π–π* character in the excited states.

**Table 5:** Selected^a^ Photophysical Data for Complexes **7**–**10** and **16**–**18**

calcd λ_em_ (nm)	medium	*T* (K)	concentration	λ_em_ (nm)^b^	τ (μs) green-shifted band^c^	τ (μs) red-shifted band	Φ_L_^d^
Complex **7**							
499	PMMA	298	2 wt %	**490**, 524, 564, 638	5.1 (76.9%), 2.8 (23.1%)	4.9 (45.0%), 2.0 (55.0%)	0.60
	CH_2_Cl_2_	298	1 × 10^–5^ M	**490**, 524,564	4.2		0.60
	CH_2_Cl_2_	77	1 × 10^–5^ M	**484**, 522, 636	7.2 (42.3%), 4.8 (57.7%)	3.8 (54.7%), 2.3 (45.3%)	
Complex **8**							
501	PMMA	298	2 wt %	**488**, 526, 560	5.1 (78.1%), 3.0 (21.9%)		0.72
	CH_2_Cl_2_	298	1 × 10^–5^ M	**488**, 522,562	4.2		0.56
	CH_2_Cl_2_	77	1 × 10^–5^ M	**482**, 518, 552, 650	7.3 (51.1%), 4.2 (48.9%)	35.0 (34.2%), 5.3 (65.8%)	
Complex **9**							
498	PMMA	298	2 wt %	**496**, 532, 574, 640	5.4 (71.0%), 2.4 (29.0%)	4.9 (30.6%), 2.2 (69.4%)	0.57
	CH_2_Cl_2_	298	1 × 10^–5^ M	**494**, 530, 570	4.7		0.62
	CH_2_Cl_2_	77	1 × 10^–5^ M	486, **496**, 526, 625	6.5 (70.3%), 3.0 (29.7%)	4.4 (42.4%), 2.3 (57.6%)	
Complex **10**							
501	PMMA	298	2 wt %	**494**, 528, 566, 646	5.6 (78.4%), 2.7 (21.6%)	4.9 (45.0%), 2.0 (55.0%)	0.75
	CH_2_Cl_2_	298	1 × 10^–5^ M	**494**, 526, 560	5.6		0.60
	CH_2_Cl_2_	77	1 × 10^–5^ M	**498**, 530, 648	8.0 (64.0%), 3.7 (36.0%)	3.8 (73.1%), 1.4 (26.9%)	
Complex **16**							
492	PMMA	298	5 wt %	**490**, 523, 561	1.5 (36.9%), 4.2 (63.1%)		0.01
	CH_2_Cl_2_	298	1 × 10^–5^ M	**491**, 525, 563	3.6		0.03
	CH_2_Cl_2_	77	1 × 10^–5^ M	**482**, 519, 556, 650	10.4 (48.3%), 6.5 (51.7%)		
Complex **17**							
496	PMMA	298	5 wt %	**495**, 527, 571	0.5 (5.4%), 3.6 (94.6%)		0.03
	CH_2_Cl_2_	298	1 × 10^–5^ M	**494**, 527, 569	3.6		0.03
	CH_2_Cl_2_	77	1 × 10^–5^ M	**496**, 530, 572	8.8 (62.8%), 3.4 (37.2%)		
Complex **18**							
439	PMMA	298	5 wt %	483, **511**	0.4 (2.5%), 5.6 (97.5%)		0.12
	CH_2_Cl_2_	298	1 × 10^–5^ M	**495**, 525, 570	23.4 (12.6%), 12.2 (87.4%)		0.10
	CH_2_Cl_2_	77	1 × 10^–5^ M	**492**, 525, 562	51.0 (44.9%), 10.1 (55.1%)		

a Table 5 summarizes the data in Table S15.

b The most intense peak is in bold.

c Relative amplitudes (%) are given in parentheses for biexponential decays.

d Absolute quantum yield.

**Figure 7:**
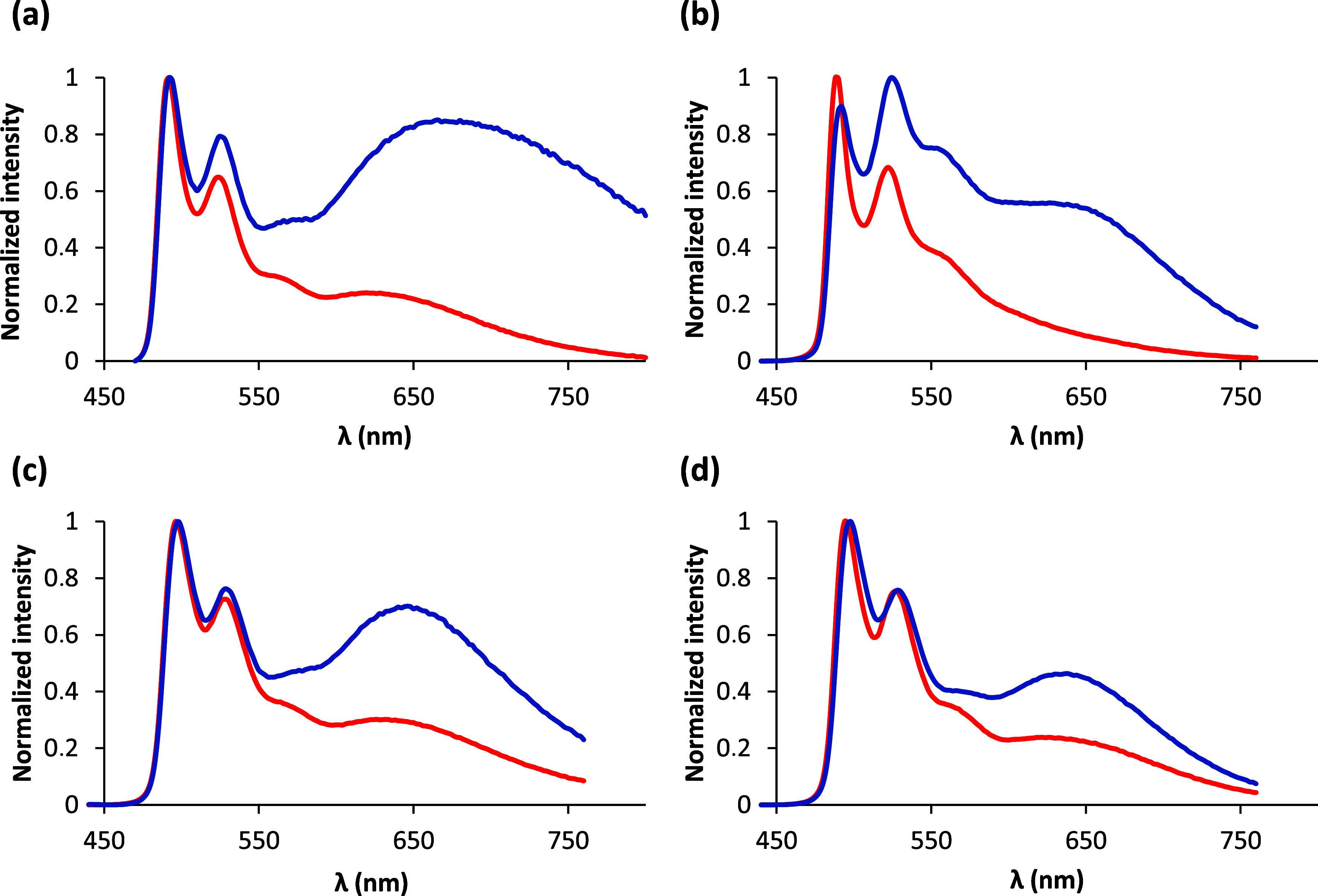
Emission spectra of complexes **7** (a), **8** (b), **9** (c), and **10** (d) in 5 wt % (blue lines) and 2 wt % (red lines) PMMA films at 298 K.

The spectra in PMMA depend on the concentration of the emitter in the film (Figure 7). For a 5 wt % concentration (PMMA_5%_), the spectra contain two narrow bands and a shoulder in the green region along with a very broad band centered around 640–670 nm. The dilution of the emitter up to 2 wt % (PMMA_2%_) produces a significant decrease in the intensity of the broad band, while the intensities of the thin bands and the shoulder are maintained. In addition, the quantum yields experience a remarkable increase of 50–100% after dilution, going from 0.30–0.50 to 0.57–0.75. The same phenomenon is observed for chlorido precursors **1** and **2** (Figures S89, S90, S99, and S100), although the quantum yields for concentrations of 2 wt % do not exceed 0.67 (Table S15). The broad band at the red region stems from aggregates with excimeric character,^33^ which quench the green emission. Their formation is consistent with the ability of this class of compounds to undergo molecular aggregation as proven in Figure 2.

**Figure 8:**
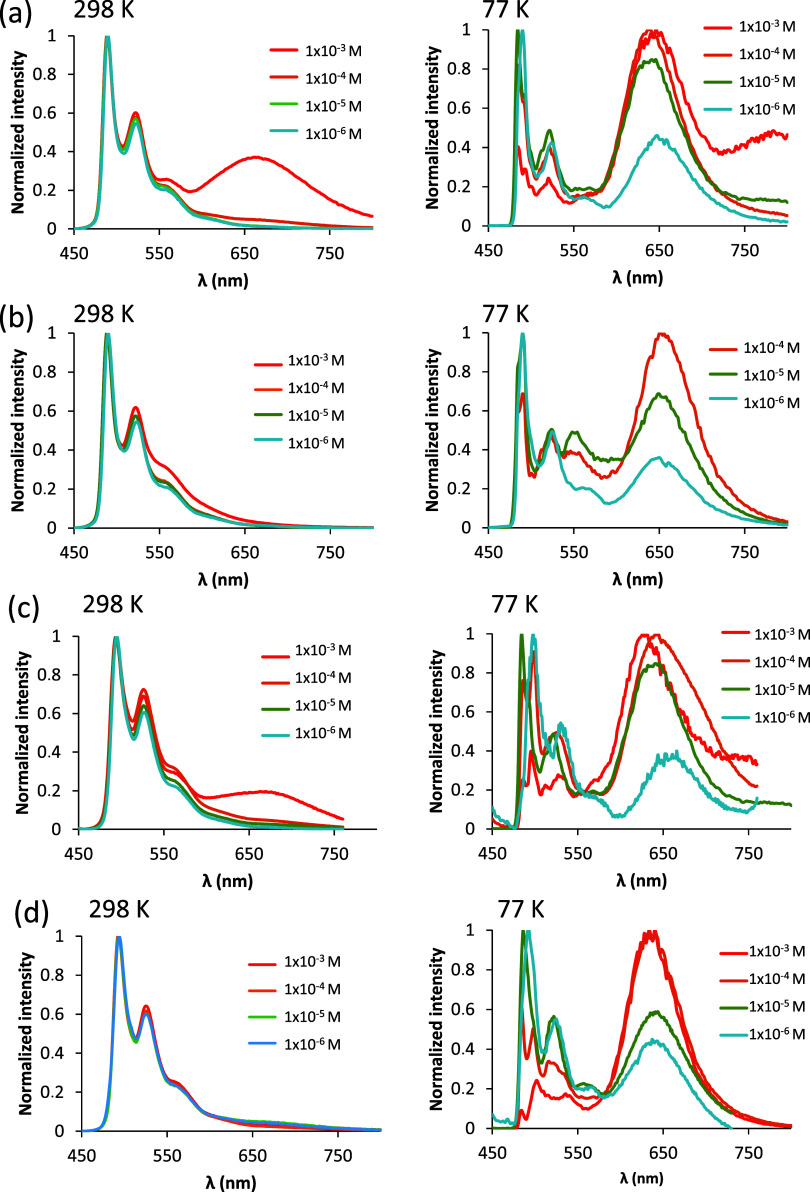
Emission spectra of complexes **7** (a), **8** (b), **9** (c), and **10** (d) in dichloromethane solutions at 298 K (left) and frozen matrices of dichloromethane at 77 K (right).

The emission spectra of solutions between 1 × 10^–6^ and 1 × 10^–4^ M of **7** and **9**, in dichloromethane, at 298 K are independent of concentration and almost superimposable with those observed in the green region of the spectra in film of PMMA. However, for 1 × 10^–3^ M, a concentration for which Beer’s law does not hold, the spectra of both emitters also show a structureless band at about 664 nm (Figure 8a [left] and c [left]). Unlike these complexes carrying an unsubstituted pyridylpyrazolate anion, the spectra of the pyridylmethylpyrazolate counterparts, **8** and **10**, do not show such red-shifted emission (Figure 8b [left] and d [left]), most likely because the methyl substituent hinders molecular aggregation and thus self-quenching. For all four emitters, the lifetimes corresponding to the most intense green band and the quantum yields also point to self-quenching, since both parameters increase as the emitter concentration decreases from 1 × 10^–3^ to 1 × 10^–6^ M; the first ones from 0.3–2.3 to 5.1–6.7 μs and the second ones from 0.05–0.17 to 0.56–0.60. As expected, spectra in frozen matrices of dichloromethane at 77 K also show the broad band in the red region (Figure 8, right).

The rate of emission decay (*k*_obs_ = 1/τ) adjusts to the modified Stern–Volmer expression (eq 1), where *k*_q_ is the rate constant for the excimer formation, [Pt] is the emitter concentration, and *k*_0_ (=1/τ_0_) is the rate of excited-state decay at infinite dilution. The respective plots of *k*_obs_*versus* [Pt] (Figures S85–S88) provide the corresponding values for the self-quenching rate constant *k*_q_ and the intrinsic lifetime τ_0_, which are collected in Table 6. These values compare well with those reported for other emissive platinum(II) compounds.^34^
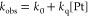
1The pyridylpyrrolate derivatives **16**–**18** are also green emitters, with similar behavior to **7**–**10** in dichloromethane. However, the observed quantum yields are low, reaching a value of only 0.12 at best. Low quantum yields have been associated in some cases to the existence of thermally accessible triplet excited states, centered on the metal, which provide nonradiative decay pathways.^35^ Indeed, this is the case. In contrast to **7**–**10**, complexes **16**–**18** have a five-coordinate triplet excited state of slightly lower energy than the emissive triplet of square-planar geometry (567–593 *versus* 439–496 nm), which is mainly centered in the orbital *dz*^2^ of the metal (Figures S293–S296). Thus, the impossibility of the N^1^ atom of the pyrazolate group to participate in a chelating coordination explains the difference in efficiency observed between the pyridylpyrazolate and pyridylpyrrolate emitters. In this context, it should be mentioned that hypothetical isomers **7b**–**10b**, with the pyrazolate group coordinated by the N^2^ atom, also have five-coordinate triplet excitation states, similar to those of **16**–**18**.

**Table 6:** Values of Intrinsic Lifetime and Self-Quenching Rate Constants for Complexes **7**–**10**

complex	τ_o_ (μs)	*k*_q_ (M^–1^·s^–1^)
**7**	4.4	3.8 × 10^9^
**8**	4.4	0.9 × 10^9^
**9**	6.2	2.5 × 10^9^
**10**	5.8	0.3 × 10^9^

## Concluding Remarks

This study has revealed that the substitution of chloride by hydroxide in platinum(II) square-planar complexes, which carry an *N*,*C*,*N*-pincer of the type 1,3-bis(2-pyridyl)phenyl or 1,3-bis(2-pyridyloxy)phenyl, gives platinum(II)-hydroxide complexes. These species are synthetic intermediates, which promote deprotonation of 3-(2-pyridyl)pyrazoles and 2-(2-pyridyl)-3,5-bis(trifluoromethyl)pyrrole. Subsequent coordination of the resulting anions leads to compounds with *N*,*C*,*N*-tridentate and *N*,*N*-bidentate groups. Together, the ligands of these classes do not impose a five-coordinate coordination on the platinum(II) ion in any case. This is because the tridentate group is a chelating ligand when the bidentate group acts as a chelate, while the bidentate group is a monodentate ligand when the tridentate group acts as a pincer.

The 1,3-bis(2-pyridyl)phenyl moiety is a better pincer ligand than the 1,3-bis(2-pyridyloxy)phenyl one. Although the oxygen atoms between phenyl and pyridyl in the latter allow for more comfortable coordination of the pincer, they prevent delocalization of electrons within the generated metallaheterocycle. Such delocalization in the 1,3-bis(2-pyridyl)phenyl derivatives stabilizes tridentate coordination, compensating and exceeding for the increase of comfort provided by the 1,3-bis(2-pyridyloxy)phenyl coordination. Along the same lines, pyridylpyrazolate anions are better chelate ligands than pyridylpyrrolate. The N^1^-pyrazolate atom produces a remote stabilizing effect on the chelate, which seems to be a consequence of the delocalization of its lone pair in the diheterometallacycle resulting from coordination. When the pyridylpyrazolate anions act as a monodentate ligand, the coordination of the pyrazolate group is preferred, with N^1^ being favored over N^2^. However, a trifluoromethyl group at 5-position of the pyrazolate unit promotes the slippage of the platinum(II) ion from N^1^ to N^2^ and thus benefits chelate coordination.

This cocktail of features is present in the conflict between *N*,*C*,*N*-pincer ligands and *N*,*N*-chelating groups, mentioned above, and manifests itself in different ways. The 1,3-bis(2-pyridyl)phenyl groups always act as pincers. Thus, in their presence, both types of anions, pyridylpyrazolate and pyridylpyrrolate, coordinate as monodentate ligands. In contrast, the 1,3-bis(2-pyridyloxy)phenyl moiety only acts selectively as a pincer in the presence of pyridylpyrrolate anions. Together, the ligands 1,3-bis(2-pyridyloxy)phenyl and pyridylpyrazolate give rise to equilibria between all possible square-planar isomers resulting from their different coordination possibilities, particularly when the pyrazolate unit of pyridylpyrazolate bears a trifluoromethyl substituent at the 5-position.

Complexes containing pincer ligands of the type 1,3-bis(2-pyridyl)phenyl and κ^1^-*N*^1^-pyridylpyrazolate are among the most efficient green phosphorescent emitters of platinum(II). On PMMA film and in dichloromethane, at high concentrations, these compounds experience self-quenching, which is provided by their strong tendency to undergo molecular stacking. Aggregation occurs as a consequence of π–π interactions between the aromatic rings of the pincers, which are reinforced by weak platinum–platinum interactions. The existence of both has been confirmed using an AIM approach.

In summary, a study on the conflict posed by the joint coordination of ligands of the 1,3-bis(2-pyridyl)phenyl- or 1,3-bis(2-pyridyloxy)phenyl-type, and pyridylpyrazolate- or pyridylpyrrolate-type ligands to platinum(II) has allowed us to establish coordination priorities between these classes of ligands, isolate and characterize complexes with unusual coordination modes of the ligands involved, and discover new highly efficient green phosphorescent emitters.^36^

## Experimental Section

### General Information

All reactions were carried out with exclusion of air using Schlenk-tube techniques or in a dry box. Instrumental methods and X-ray diffractometry analysis details are given in the Supporting Information. In the NMR spectra (Figures S1–S52), the chemical shifts (in ppm) are referenced to residual solvent peaks (^1^H, ^13^C{^1^H}), external CFCl_3_ (^19^F{^1^H}) or Na_2_PtCl_6_ (^195^Pt{^1^H}), while coupling constants are given in hertz. In the ^13^C{^1^H} NMR spectra, not all ^13^C–^195^Pt couplings could be resolved. PtCl{κ^3^-*N*,*C*,*N*-[py-C_6_H_3_-py]} (**1**),^15a^ PtCl{κ^3^-*N*,*C*,*N*-[py-C_6_HMe_2_-py]} (**2**),^18b^ and PtCl{κ^3^-*N*,*C*,*N*-[py-O-C_6_H_3_-O-py]} (**3**)^21^ were prepared according to the reported procedures.

### Preparation of **4**

A suspension of **1** (200 mg, 0.433 mmol) in tetrahydrofuran (10 mL) was treated with KOH (574 mg, 8.7 mmol), and the resulting mixture was stirred for 48 h at 65 °C to get an orange suspension. After this time, it was cooled to room temperature, the supernatant solution was removed, and the orange solid was washed with water (4 × 7 mL) and dried under vacuum. Yield: 154 mg (80%). Anal. calcd for C_16_H_12_N_2_OPt: C, 43.34; H, 2.73; N, 6.32. Found: C, 43.05; H, 2.75; N, 6.28. High-resolution mass spectrometry (HRMS) (electrospray, *m*/*z*) calcd for C_16_H_13_N_2_Pt [M + H]^+^: 444.0672; found: 444.0671. IR (cm^–1^): ν(O–H) 3476 (w), ν(C=N), ν(C=C), 1606 (m), 1557 (m). The low solubility of the complex prevented getting its ^1^H, ^13^C{^1^H}, and ^195^Pt{^1^H} NMR spectra.

### Preparation of **5**

A suspension of **2** (200 mg, 0.41 mmol) in tetrahydrofuran (10 mL) was treated with KOH (528 mg, 8 mmol), and the resulting mixture was stirred for 48 h at 65 °C to get an orange suspension. After this time, it was cooled to room temperature, the supernatant solution was removed, and the orange solid was washed with water (4 × 7 mL) and dried under vacuum. Yield: 154 mg (80%). Anal. calcd for C_18_H_16_N_2_OPt: C, 45.86; H, 3.42; N, 5.94. Found: C, 45.49; H, 3.25; N, 5.77. HRMS (electrospray, *m*/*z*) calcd for C_18_H_15_N_2_Pt [M – OH]^+^: 454.0879; found: 454.0908. IR (cm^–1^): ν(O–H) 3480 (w), ν(C=N), ν(C=C), 1601 (m), 1545 (m). ^1^H NMR (400.1 MHz, THF-*d*_8_, 338 K): δ 9.29 (d with ^195^Pt satellites, *J*_H–H_ = 5.1, *J*_H–Pt_ = 41.9, 2H, py), 8.01–7.85 (4H, py), 7.24 (t, *J*_H–H_ = 5.8, 2H, py), 6.71 (s, 1H, Ph), 2.63 (s, 6H, CH_3_), −0.25 (broad singlet, 1H, OH). ^195^Pt{^1^H} NMR (85.6 MHz, THF-*d*_8_, 298 K): δ −3383 (s). The low solubility of the complex prevented getting its ^13^C{^1^H} NMR spectrum.

### Preparation of **6**

A suspension of **3** (200 mg, 0.405 mmol) in tetrahydrofuran (7 mL) was treated with KOH (107 mg, 1.62 mmol), and the resulting mixture was stirred for 24 h at room temperature to get a yellow solution. The solvent was removed, and the yellow solid obtained was washed with water (4 × 5 mL) and dried under vacuum. Yield: 153 mg (79%). Anal. calcd for C_16_H_12_N_2_O_3_Pt: C, 40.43; H, 2.54; N, 5.94. Found: C, 40.03; H, 2.81; N, 6.17. HRMS (electrospray, *m*/*z*) calcd for C_16_H_13_N_2_O_3_Pt [M + H]^+^: 476.0570; found: 476.0575. IR (cm^–1^): ν(O–H) 3052 (w), ν(C=N), ν(C=C), 1611 (m), 1567 (m). ^1^H NMR (400.1 MHz, THF-*d*_8_, 298 K): δ 10.03 (dd with ^195^Pt satellites, *J*_H–H_ = 6.3, 2.0, *J*_H–Pt_ = 43.9, 2H, py-NCN), 7.98–7.91 (m, 2H, py-NCN), 7.26–7.16 (m, 2H py NCN), 7.10–7.05 (m, 2H py NCN), 6.98–6.92 (m, 1H Ph), 6.86–6.79 (m, 2H Ph), −0.20 (s, 1H OH). The high instability in solution of the complex prevented getting its ^13^C{^1^H} and ^195^Pt{^1^H} NMR spectra at 298 K. For this reason, these spectra were recorded at 223 K. ^13^C{^1^H}-APT NMR (100.63 MHz, THF-*d*_8_, 223 K): δ 158.5 (s, C NCN), 154.4 (s, C NCN), 150.1 (s, CH py NCN), 141.4 (s, CH py NCN), 124.0 (s, CH Ph), 119.4 (s, CH py NCN), 115.4 (s, CH py NCN), 112.4 (s, CH Ph), 104.5 (s, C NCN). ^195^Pt{^1^H} NMR (85.6 MHz, THF-*d*_8_, 223 K): δ −2922 (s).

### Preparation of **7**

3-(2-Pyridyl)pyrazole (66 mg, 0.45 mmol) was added to an orange suspension of **4** (200 mg, 0.45 mmol) in acetone (7 mL), and the resulting mixture was stirred for 1 h at room temperature to get a yellow suspension. The solution was removed, and the yellow solid was washed with cold acetone (3 × 5 mL) and dried under vacuum. Yield: 132 mg (51%). Anal. calcd for C_24_H_17_N_5_Pt: C, 50.53; H, 3.00; N, 12.28. Found: C, 50.24; H, 2.95; N, 12.16. HRMS (electrospray, *m*/*z*) calcd for C_24_H_18_N_5_Pt [M + H]^+^: 571.1206; found: 571.1221. IR (cm^–1^): ν(C=N), ν(C=C) 1607 (w), 1592 (m). NMR spectra of the yellow solid in CD_2_Cl_2_ reveal the presence of a unique isomer. ^1^H NMR (300.13 MHz, CD_2_Cl_2_, 298 K): δ 8.62 (d with ^195^Pt satellites, *J*_H–H_ = 5.5, *J*_H–Pt_ = 41.9, 2H py-NCN), 8.56 (d, *J*_H–H_ = 4.7, 1H py), 8.18 (d, *J*_H–H_ = 7.5, 1H py), 7.96 (t, *J*_H–H_ = 7.5, 2H py-NCN), 7.83–7.71 (3H, 2H py-NCN + 1H pz), 7.66 (t, *J*_H–H_ = 8.3, 1H py), 7.55 (d, *J*_H–H_ = 8.3, 2H Ph), 7.29–7.20 (3H, 2H py-NCN + 1H Ph), 7.10–7.06 (2H, 1H py + 1H pz). ^13^C{^1^H}-APT NMR (75.48 MHz, CD_2_Cl_2_, 298 K): δ 168.8 (s, C py-NCN), 167.6 (s, Pt-C NCN), 155.9 (s, C py), 153.6 (s, C pz), 152.3 (s, CH py-NCN), 149.5 (s, CH py), 142.7 (s, C NCN), 141.3 (s, CH pz), 139.6 (s, CH py-NCN), 136.3 (s, CH py), 124.3 (s with ^195^Pt satellites, *J*_C–Pt_ = 30.0, CH Ph), 123.7 (s with ^195^Pt satellites, *J*_C–Pt_ = 24.0, CH Ph), 120.9 (s, CH py), 119.9 (s, with ^195^Pt satellites, *J*_C–Pt_ = 48.0, *J*_C–Pt_ = 24.0, CH py-NCN) 119.7 (s, CH py), 103.5 (s, CH pz). ^195^Pt{^1^H} NMR (85.6 MHz, CD_2_Cl_2_, 298 K): δ −3597 (s).

### Preparation of **8**

3-(2-Pyridyl)-5-methylpyrazole (108 mg, 0.676 mmol) was added to a suspension of **4** (200 mg, 0.451 mmol) in acetone (8 mL), and the resulting mixture was stirred at room temperature for 24 h to get a yellow suspension. The yellow suspension was decanted, the supernatant solution was removed, and the yellow solid was washed with cold acetone (3 × 2 mL) and diethyl ether (3 × 3 mL), and dried under vacuum. Yield: 158 mg (60%). Anal. calcd for C_25_H_19_N_5_Pt: C, 51.37; H, 3.28; N, 11.98. Found: C, 51.03; H, 3.31; N, 11.90. HRMS (electrospray, *m*/*z*) calcd for C_25_H_20_N_5_Pt [M + H]^+^: 585.1361; found: 585.1357. IR (cm^–1^): ν(C=N), ν(C=C) 1605 (w), 1591 (m). NMR spectra of the yellow solid in CD_2_Cl_2_ reveal the presence of a unique isomer. ^1^H NMR (400.1 MHz, CD_2_Cl_2_, 298 K): δ 8.52 (d, *J*_H–H_ = 3.7, 1H, py), 8.22 (d with ^195^Pt satellites, *J*_H–H_ = 4.9, *J*_H–Pt_ = 43.0, 2H, py-NCN), 8.10 (d, *J*_H–H_ = 7.7, 1H, py), 7.95 (t, *J*_H–H_ = 7.5, 2H, py-NCN), 7.77 (d, *J*_H–H_ = 7.8, 2H, py-NCN), 7.62 (t, *J*_H–H_ = 7.8, 1H, py), 7.56 (d, *J*_H–H_ = 7.5, 2H, Ph), 7.28 (t, *J*_H–H_ = 7.5, 1H, Ph), 7.17 (t, *J*_H–H_ = 5.8, 2H, py-NCN), 7.04 (t, *J*_H–H_ = 5.9, 1H, py), 6.83 (s, 1H, pz), 2.45 (s, 3H, CH_3_). ^13^C{^1^H}-APT NMR (100.63 MHz, CD_2_Cl_2_, 298 K): δ 168.7 (s with ^195^Pt satellites, *J*_C–Pt_ = 91.7, Pt-C), 167.2 (s, C py-NCN), 155.9 (s, C py), 154.2 (s, C pz), 152.5 (s, CH py-NCN), 149.3 (s, CH py), 147.4 (s, C pz), 142.8 (s, C NCN), 139.7 (s, CH py-NCN), 136.2 (s, CH py), 124.4 (s, CH Ph), 124.0 (s with ^195^Pt satellites, *J*_C–Pt_ = 32.4, CH py-NCN), 123.8 (s, CH Ph), 120.6 (s, CH py), 120.0 (s with ^195^Pt satellites, *J*_C–Pt_ = 47.3, CH py-NCN), 119.5 (s, CH py), 102.9 (s, CH pz), 14.1 (s, CH_3_). ^195^Pt{^1^H} NMR (85.6 MHz, CD_2_Cl_2_, 298 K): δ −3579 (s).

### Preparation of **9**

3-(2-Pyridyl)pyrazole (62 mg, 0.424 mmol) was added to a suspension of **5** (200 mg, 0.424 mmol) in acetone (7 mL), and the resulting mixture was stirred for 1 h at room temperature to get a yellow suspension. The solution was removed, and the yellow solid was washed with cold acetone (3 × 5 mL) and dried under vacuum. Yield: 130 mg (51%). Crystals suitable for X-ray diffraction analysis were obtained at 4 °C by vapor diffusion of pentane into a dichloromethane solution of the complex. Anal. calcd for C_26_H_21_N_5_Pt: C, 52.17; H, 3.54; N, 11.70. Found: C, 51.92; H, 3.50; N, 11.58. HRMS (electrospray, *m*/*z*) calcd for C_26_H_22_N_5_Pt [M + H]^+^: 599.1520; found: 599.1546. IR (cm^–1^): ν(C=N), ν(C=C) 1603 (w), 1560 (m). NMR spectra of the yellow solid in CD_2_Cl_2_ reveal the presence of a unique isomer. ^1^H NMR (300.13 MHz, CD_2_Cl_2_, 298 K): δ 8.55 (d, *J*_H–H_ = 4.1, 1H py), 8.48 (d with ^195^Pt satellites, *J*_H–H_ = 5.6, *J*_H–Pt_ = 43.0, 2H py-NCN), 8.17 (d, *J*_H–H_ = 8.3, 1H py), 7.97–7.83 (m, 4H py-NCN), 7.72 (s, 1H pz), 7.65 (t, *J*_H–H_ = 8.1, 1H py), 7.18–7.01 (4H, 2H py-NCN + 1H py + 1H pz), 6.84 (s, 1H Ph), 2.66 (s, 6H CH_3_). ^13^C{^1^H} NMR (100.63 MHz, CD_2_Cl_2_, 298 K): δ 169.5 (s with ^195^Pt satellites, *J*_C–Pt_ = 107.0, C py), 169.0 (s, Pt–C), 155.9 (s, C py), 153.6 (s, C pz), 152.1 (s, CH py-NCN), 149.4 (s, CH py), 139.6 (s, CH pz), 139.3 (s, CH py-NCN), 137.0 (s with ^195^Pt satellites, *J*_C–Pt_ = 30.0, C Ph), 136.2 (s, CH py), 131.7 (s, CH Ph), 123.1 (s with ^195^Pt satellites, *J*_C–Pt_ = 49.0, CH py-NCN), 122.8 (s with ^195^Pt satellites, *J*_C–Pt_ = 36.0, CH py-NCN), 120.8 (s, CH py), 119.7 (s, CH py), 103.5 (s with ^195^Pt satellites, *J*_C–Pt_ = 15.0, CH pz), 22.1 (s, CH_3_). ^195^Pt{^1^H} NMR (85.6 MHz, CD_2_Cl_2_, 298 K): δ −3567 (s).

### Preparation of **10**

3-(2-Pyridyl)-5-methylpyrazole (40.5 mg, 0.254mmol) was added to an orange suspension of **5** (100 mg, 0.212 mmol) in acetone (4 mL), and the resulting mixture was stirred at room temperature for 1 h to get a yellow suspension. The suspension was decanted, the supernatant solution was removed, and the yellow solid was washed with cold acetone (3 × 2 mL) and diethyl ether (3 × 3 mL), and dried under vacuum. Yield: 84 mg (65%). Anal. calcd for C_27_H_23_N_5_Pt: C, 52.94; H, 3.78; N, 11.43. Found: C, 52.59; H, 3.91; N, 11.26. HRMS (electrospray, *m*/*z*) calcd for C_27_H_24_N_5_Pt [M + H]^+^: 613.1676; found: 613.1661. IR (cm^–1^): ν(C=N), ν(C=C) 1602 (m), 1590 (m). NMR spectra of the yellow solid in CDCl_3_ reveal the presence of a unique isomer. ^1^H NMR (400.1 MHz, CDCl_3_, 298 K): δ 8.59 (d, *J*_H–H_ = 4.6, 1H py), 8.26 (d with ^195^Pt satellites, *J*_H–H_ = 5.5, *J*_H–Pt_ = 42.1, 2H py NCN), 8.19 (d, *J*_H–H_ = 7.9, 1H py), 7.91–7.82 (4H py NCN), 7.61 (t, *J*_H–H_ = 7.4, 1H py), 7.09–7.02 (3H, 1H py + 2H py NCN), 6.94 (s, 1H pz), 6.82 (s, 1H Ph NCN), 2.67 (s, 6H CH_3_ NCN), 2.47 (s, 3H CH_3_ pz). ^13^C{^1^H}-APT NMR (100.63 MHz, CDCl_3_, 298 K): δ 169.7 (s, C py), 169.3 (s with ^195^Pt satellites, *J*_C–Pt_ = 94.5, Pt-C), 155.5 (s, C py), 154.1 (s, C pz), 152.4 (s, CH py NCN), 149.1 (s, CH py), 147.3 (s, C pz), 139.0 (s, C NCN), 138.8 (s, CH py NCN), 136.5 (s with ^195^Pt satellites, *J*_C–Pt_ = 31.2, C NCN), 136.0 (s, CH py), 131.3 (s, CH Ph NCN), 122.6 (s with ^195^Pt satellites, *J*_C–Pt_ = 34.6, CH py NCN), 122.5 (s with ^195^Pt satellites, *J*_C–Pt_ = 24.6, CH py NCN), 120.5 (s, CH py), 119.9 (s, CH py), 102.7 (s, CH pz), 22.0 (s, CH_3_ NCN), 14.1 (s, CH_3_ pz). ^195^Pt{^1^H} NMR (85.6 MHz, CDCl_3_, 298 K): δ −3558 (s).

### Preparation of **11**

3-(2-Pyridyl)-5-trifluoromethylpyrazole (192 mg, 0.902 mmol) was added to an orange suspension of **4** (200 mg, 0.451 mmol) in acetone (8 mL), and the resulting mixture was stirred at room temperature for 24 h to get a yellow suspension. After this time, the suspension was decanted, the solution was removed, and the resulting yellow solid was washed with cold acetone (3 × 4 mL) and diethyl ether (3 × 5 mL), and dried under vacuum. Yield: 173 mg (60%). Anal. calcd for C_25_H_16_F_3_N_5_Pt: C, 47.03; H, 2.53; N, 10.97. Found: C, 47.35; H, 2.78; N, 10.73. HRMS (electrospray, *m*/*z*) calcd for C_25_H_17_F_3_N_5_Pt [M + H]^+^: 639.1080; found: 639.1077. IR (cm^–1^): ν(C=N), ν(C=C) 1608 (m), 1595 (m), 1567 (w). NMR spectra of the yellow solid in CD_2_Cl_2_ reveal the presence of isomers **11a** and **11b** in a 1:0.89 molar ratio at 223 K.

#### Spectroscopic Data for **11a**

^1^H NMR (400.1 MHz, CD_2_Cl_2_, 223 K): δ 8.75 (d, *J*_H–H_ = 8.0, 1H, CH py), 8.45 (d, *J*_H–H_ = 4.7, 1H, py), 8.0 (d with ^195^Pt satellites, *J*_H–H_ = 5.3, *J*_H–Pt_ = 40.3, 2H, py NCN), 7.97–7.87 (m, 2H, py NCN), 7.76 (t, *J*_H–H_ = 7.1, 2H, py NCN), 7.54 (d, *J*_H–H_ = 7.7, 2H, Ph), 7.45 (dt, *J*_H–H_ = 7.5, 1.9, 1H, py), 7.29 (s, 1H, pz), 7.27 (t, *J*_H–H_ = 7.7, 1H, Ph), 7.07 (t, *J*_H–H_ = 5.8, 2H, py NCN), 7.03–6.98 (m, 1H, py). ^13^C{^1^H} NMR (100.63 MHz, CD_2_Cl_2_, 223 K): δ 167.4 (s, with ^195^Pt satellites, *J*_H–Pt_ = 104.2, C NCN), 163.7 (s, C NCN), 151.8 (s, C py), 151.6 (s, C pz), 151.2 (s, CH py NCN), 149.2 (s, CH py), 143.6 (q, *J*_C–F_ = 36, *C*CF_3_), 142.0 (s, with ^195^Pt satellites, *J*_C–Pt_ = 90.0, C NCN), 139.5 (s, CH py NCN), 136.0 (s, CH py), 123.9 (s, CH Ph), 123.8 (s, CH py NCN), 123.6 (s, CH Ph), 122.6 (q, *J*_C–F_ = 268, CF_3_), 121.6 (s, CH py), 120.5 (s, CH py), 119.8 (s, CH py NCN), 103.4 (s, CH pz). ^19^F{^1^H} NMR (376 MHz, CD_2_Cl_2_, 223 K): δ −60.1 (s, CF_3_). ^195^Pt{^1^H} NMR (85.6 MHz, CD_2_Cl_2_, 298 K): δ −3584 (s).

#### Spectroscopic Data for **11b**

^1^H NMR (400.1 MHz, CD_2_Cl_2_, 223 K): δ 8.56 (d, *J*_H–H_ = 4.9, 1H, py), 8.12 (d, *J*_H–H_ = 8.1, 1H, py), 7.97–7.87 (m, 2H, py NCN), 7.84 (d, *J*_H–H_ = 5.5, 2H, py NCN), 7.76 (t, *J*_H–H_ = 7.1, 2H, py NCN), 7.67 (dt, *J*_H–H_ = 7.7, 1.8, 1H, py), 7.54 (d, *J*_H–H_ = 7.7, 2H, Ph), 7.41 (s, 1H, pz), 7.27 (t, *J*_H–H_ = 7.7, 1H Ph), 7.17–7.09 (3H, 1H py + 2H py NCN). ^13^C{^1^H} NMR (100.63 MHz, CD_2_Cl_2_, 223 K): δ 167.5 (s, with ^195^Pt satellites, *J*_H–Pt_ = 104.2, C NCN), 163.1 (s, C NCN), 153.3 (s, C py), 153.0 (s, C pz), 151.4 (s, CH py NCN), 149.1 (s, CH py), 142.1 (s, with ^195^Pt satellites, *J*_C–Pt_ = 90.0, C NCN), 140.9 (q, *J*_C–F_ = 35, *C*CF_3_), 139.7 (s, CH py NCN), 136.4 (s, CH py), 124.0 (s, CH Ph), 123.8 (s, CH py NCN), 123.5 (s, CH Ph), 123.2 (q, *J*_C–F_ = 266, CF_3_), 121.4 (s, CH py), 119.9 (s, CH py NCN), 119.0 (s, CH py), 103.3 (s, CH pz). ^19^F{^1^H} NMR (376 MHz, CD_2_Cl_2_, 223 K): δ −58.2 (s, CF_3_). ^195^Pt{^1^H} NMR (85.6 MHz, CD_2_Cl_2_, 298 K): δ −3650 (s).

### Preparation of **12**

3-(2-Pyridyl)-5-trifluoromethylpyrazole (181 mg, 0.848 mmol) was added to an orange suspension of **5** (200 mg, 0.424 mmol) in acetone (7 mL), and the resulting mixture was stirred at room temperature for 1 h. After this time, the suspension was decanted, the solution was removed, and the resulting yellow solid was washed with cold acetone (2 × 5 mL) and diethyl ether (3 × 5 mL), and dried under vacuum. Yield: 170 mg (60%). Crystals suitable for X-ray diffraction analysis were obtained by vapor diffusion of pentane into a dichloromethane solution of the complex at 4 °C. Anal. calcd for C_27_H_20_F_3_N_5_Pt: C, 46.65; H, 3.02; N, 10.51. Found: C, 46.40; H, 2.95; N, 10.35. HRMS (electrospray, *m*/*z*) calcd for C_27_H_21_F_3_N_5_Pt [M + H]^+^: 667.1394; found: 667.1406. IR (cm^–1^): ν(C=N), ν(C=C) 1591 (w), 1562 (w). NMR spectra of the yellow solid in CD_2_Cl_2_ reveal the presence of isomers **12a** and **12b** in a 1:0.89 molar ratio at 223 K.

#### Spectroscopic Data for **12a**

^1^H NMR (400.1 MHz, CD_2_Cl_2_, 223 K): δ 8.93 (d, *J*_H–H_ = 8.1, 1H CH py), 8.47 (d, *J*_H–H_ = 5.3, 1H CH py), 7.91–7.87 (4H, py NCN), 7.76 (d, *J*_H–H_ = 5.3, 2H py NCN), 7.44 (t, *J*_H–H_ = 8.1, 1H CH py), 7.31 (s, 1H CH pz), 7.04–7.00 (3H, 2H py NCN + 1H CH py), 6.81 (s, 1H CH NCN), 2.61 (s, 6H CH_3_). ^13^C{^1^H} NMR (100.63 MHz, CD_2_Cl_2_, 223 K): δ 168.1 (s, with ^195^Pt satellites, *J*_C–Pt_ = 106.2, C NCN), 165.7 (s, C NCN), 151.8 (s, C py), 151.4 (s, C pz), 151.1 (s, CH py NCN), 149.2 (s, CH py), 143.5 (q, *J*_C–F_ = 35, *C*CF_3_), 139.1 (s, CH py NCN), 138.2 (s, C NCN), 136.7 (s, C NCN), 136.4 (s, CH py), 131.4 (s, CH Ph NCN), 123.2 (q, *J*_C–F_ = 268, CF_3_), 122.9, 122.7 (both s, CH py NCN), 121.5 (s, CH py), 120.2 (s, CH py), 103.5 (s, CH pz), 21.9 (s, CH_3_). ^19^F{^1^H} NMR (376 MHz, CD_2_Cl_2_, 223 K): δ −60.0 (s, CF_3_). ^195^Pt{^1^H} NMR (85.6 MHz, CD_2_Cl_2_, 298 K): δ −3562 (s).

#### Spectroscopic Data for **12b**

^1^H NMR (400.1 MHz, CD_2_Cl_2_, 223 K): δ 8.56 (d, *J*_H–H_ = 5.3, 1H CH py), 8.12 (d, *J*_H–H_ = 7.3, 1H CH py), 7.91–7.87 (6H py NCN), 7.68 (t, *J*_H–H_ = 8.1, 1H CH py), 7.39 (s, 1H CH pz), 7.14 (t, *J*_H–H_ = 7.0, 1H CH py), 7.04–7.00 (m, 2H py NCN), 6.79 (s, 1H CH NCN), 2.60 (s, 6H CH_3_). ^13^C{^1^H} NMR (100.63 MHz, CD_2_Cl_2_, 223 K): δ 168.2 (s, with ^195^Pt satellites, *J*_C–Pt_ = 106.2, C NCN), 165.2 (s, C NCN), 153.3 (s, C py), 153.0 (s, C pz), 150.9 (s, CH py NCN), 149.1 (s, CH py), 140.7 (q, *J*_C–F_ = 35, *C*CF_3_), 139.3 (s, CH py NCN), 138.3 (s, C NCN), 136.6 (s, C NCN), 135.9 (s, CH py), 131.2 (s, CH Ph NCN), 122.8, 122.7 (s, CH py NCN), 122.5 (q, *J*_C–F_ = 266, CF_3_), 121.4 (s, CH py), 118.9 (s, CH py), 103.4 (s, CH pz), 21.8 (s, CH_3_). ^19^F{^1^H} NMR (376 MHz, CD_2_Cl_2_, 223 K): δ −58.2 (s, CF_3_). ^195^Pt{^1^H} NMR (85.6 MHz, CD_2_Cl_2_, 298 K): δ −3624 (s).

### Preparation of **13**

3-(2-Pyridyl)pyrazole (73 mg, 0.5 mmol) was added to a pale yellow suspension of **6** (200 mg, 0.42 mmol) in acetone (7 mL), and the resulting mixture was stirred for 1 h at room temperature to get a light yellow solution. The solvent was evaporated, and the addition of diethyl ether (4 mL) afforded a yellowish-white solid that was washed with diethyl ether (3 × 4 mL) and dried under vacuum. Yield: 127 mg (50%). Anal. calcd for C_24_H_17_N_5_O_2_Pt: C, 47.84; H, 2.84; N, 11.62. Found: C, 47.45; H, 2.81; N, 11.50. HRMS (electrospray, *m*/*z*) calcd for C_24_H_18_N_5_O_2_Pt [M + H]^+^: 603.1105; found: 603.1119. IR (cm^–1^): ν(C=N), ν(C=C) 1613 (m), 1566 (m). NMR spectra of the solid in CD_2_Cl_2_ reveal the presence of isomers **13a** and **13c** in a 1:0.60 molar ratio at 253 K.

#### Spectroscopic Data for Isomer **13a**

^1^H NMR (400.1 MHz, CD_2_Cl_2_, 253 K): δ 8.53 (d, *J*_H–H_ = 4.9, 1H py), 8.04 (d, *J*_H–H_ = 8.1, 1H py), 7.85–7.76 (m, 2H py NCN), 7.72 (dd, *J*_H–H_ = 6.2, 1.6, 2H py NCN), 7.59 (dd, *J*_H–H_ = 12.7, 1.9, 1H py), 7.28 (d, *J*_H–H_ = 8.5, 2H py NCN), 7.19–7.01 (4H, 1H py + 3H Ph), 6.97 (d, *J*_H–H_ = 1.9, 1H pz), 6.79–6.72 (m, 2H py NCN), 6.63 (d, *J*_H–H_ = 2.0, 1H pz). ^13^C{^1^H}-APT NMR (100.63 MHz, CD_2_Cl_2_, 253 K): δ 161.6 (s, C Ph), 159.2 (s, C py NCN), 158.1 (s, C-Pt Ph), 153.9 (s, C py), 153.3 (s, C pz), 151.0 (s, CH py NCN), 149.1 (s, CH py), 141.3 (s, CH py), 139.0 (s, CH py NCN), 125.7 (s, CH Ph), 121.0 (s, CH py), 119.8 (s, CH py), 119.3 (s, CH py), 115.8 (s, CH py NCN), 112.6 (s, CH Ph), 104.2 (s, CH pz), 103.1 (s, CH pz). ^195^Pt{^1^H} NMR (85.6 MHz, CD_2_Cl_2_, 298 K): δ −3150 (s).

#### Spectroscopic Data for Isomer **13c**

^1^H NMR (400.1 MHz, CD_2_Cl_2_, 253 K): δ 9.23 (dd, *J*_H–H_ = 6.0, 1.6, 1H py), 8.71 (d, *J*_H–H_ = 6.0, 1H py), 8.02–7.99 (m, 1H py NCN), 7.95–7.89 (m, 1H py), 7.85–7.76 (m, 1H py), 7.67–7.54 (m, 1H py), 7.48–7.41 (m, 1H py NCN), 7.36–7.31 (m, 1H py), 7.19–7.01 (3H, 1H py + 2H Ph), 6.95–6.88 (3H, 1H py + 1H pz + 1H Ph), 6.87–6.81 (2H, 1H py NCN + 1H pz), 6.25 (d, *J*_H–H_ = 8.2, 1H py NCN). ^13^C{^1^H}-APT NMR (100.63 MHz, CD_2_Cl_2_, 253 K): δ 164.3 (s, C py NCN), 159.8 (s, C py), 155.8 (s, C py), 154.5 (s, C-Pt Ph), 153.8 (s, CH py), 153.5 (s, CH py), 149.6 (s, C pz), 147.3 (s, CH py NCN), 141.6 (s, CH py), 139.1 (s, CH py NCN), 138.1 (s, CH py), 136.3 (s, CH Ph), 121.7 (s, C Ph), 120.7 (s, CH py), 120.4 (s, CH py), 119.1 (s, CH Ph), 118.4 (s, CH py), 117.9 (s, CH py NCN), 115.2 (s, CH py), 113.1 (s, CH Ph), 111.4 (s, CH py NCN), 105.3 (s, C Ph). ^195^Pt{^1^H} NMR (85.6 MHz, CD_2_Cl_2_, 298 K): δ −3220 (s).

### Preparation of **14**

3-(2-Pyridyl)-5-methylpyrazole (80 mg, 0.5 mmol) was added to a suspension of **6** (200 mg, 0.42 mmol) in acetone (7 mL), and the resulting mixture was stirred for 1 h at room temperature to get a light yellow solution. The solvent was evaporated, and the addition of diethyl ether (4 mL) afforded a yellowish-white solid that was washed with diethyl ether (3 × 4 mL) and dried under vacuum. Yield: 161 mg (55%). Anal. calcd for C_25_H_19_N_5_O_2_Pt: C, 48.70; H, 3.11; N, 11.36. Found: C, 48.31; H, 3.08; N, 11.25. HRMS (electrospray, *m*/*z*) calcd for C_25_H_20_N_5_O_2_Pt [M + H]^+^: 617.1261; found: 617.1235. IR (cm^–1^): ν(C=N), ν(C=C) 1612 (m), 1567 (m). NMR spectra of the solid in CD_2_Cl_2_ reveal the presence of isomers **14a** and **14c** in a 1:0.50 molar ratio at 298 K.

#### Spectroscopic Data for **14a**

^1^H NMR (400.1 MHz, CD_2_Cl_2_, 298 K): δ 8.50 (d, *J*_H–H_ = 5.0, 1H py), 7.98 (d, *J*_H–H_ = 8.1, 1H py), 7.88–7.82 (m, 2H py NCN), 7.76 (dd, *J*_H–H_ = 6.3, 1.6, 2H py NCN), 7.59 (td, *J*_H–H_ = 7.7, 1.5, 1H py), 7.30 (dd, *J*_H–H_ = 8.3, 1.1, 2H py NCN), 7.16–7.11 (m, 1H Ph), 7.06–7.01 (3H, 1H py + 2H Ph), 6.83–6.78 (m, 2H py NCN), 6.73 (s, 1H pz), 2.24 (s, 3H CH_3_). ^13^C{^1^H}-APT NMR (100.63 MHz, CD_2_Cl_2_, 298 K): δ 160.5 (s, C Ph), 159.6 (s, C py NCN), 158.6 (s, C-Pt Ph), 155.4 (s, C py), 154.5 (s, C pz), 151.6 (s, CH py NCN), 149.3 (s, CH py), 146.2 (s, C-CH_3_), 141.4 (s, CH py NCN), 136.2 (s, CH py), 125.9 (s, CH Ph), 120.9 (s, CH py), 120.0 (s, CH py NCN), 119.6 (s, CH py), 115.9 (s, CH py NCN), 112.8 (s, CH Ph), 103.9 (s, CH pz), 13.4 (s, CH_3_). ^195^Pt{^1^H} NMR (85.6 MHz, CD_2_Cl_2_, 298 K): δ −3141 (s).

#### Spectroscopic Data for Isomer **14c**

^1^H NMR (400.1 MHz, CD_2_Cl_2_, 298 K): δ 9.35 (dd with ^195^Pt satellites, *J*_H–H_ = 6.0, 1.8, *J*_H–Pt_ = 42.6, 1H py), 8.71 (d with ^195^Pt satellites, *J*_H–H_ = 6.0, *J*_H–Pt_ = 49.3, 1H py), 8.03–8.00 (m, 1H py NCN), 7.97–7.91 (m, 1H py), 7.78 (d, 1H py), 7.49–7.46 (m, 1H py), 7.45–7.40 (m, 1H py NCN), 7.33 (dd, *J*_H–H_ = 8.0, 1.0, 1H py), 7.21–7.17 (m, 1H py), 7.08 (dd, *J*_H–H_ = 8.0, 1.3, 2H Ph), 6.91 (dd, *J*_H–H_ = 7.7, 1.4, 1H Ph), 6.89–6.85 (m, 1H py), 6.84 (dd, *J*_H–H_ = 4.9, 1.0, 1H py NCN), 6.38 (s, 1H pz), 6.26 (d, *J*_H–H_ = 8.4, 1H py NCN), 2.28 (s, 3H CH_3_). ^13^C{^1^H}-APT NMR (100.63 MHz, CD_2_Cl_2_, 298 K): δ 164.8 (s, C py NCN), 162.2 (s, C py), 156.7 (s, C py), 154.3 (s, C-Pt Ph), 154.2 (s, CH py) 154.1 (s, CH py), 150.7 (s, C pz), 148.5 (s, C-CH_3_), 147.6 (s, CH py NCN), 141.6 (s, CH py), 139.1 (s, CH py NCN), 126.0 (s, CH Ph), 122.1 (s, C Ph), 120.6 (s, CH py), 120.5 (s, CH py), 119.3 (s, CH Ph), 118.4 (s, CH py), 118.2 (s, CH py NCN), 115.3 (s, CH py), 113.2 (s, CH Ph), 111.8 (s, CH py NCN), 106.2 (s, C Ph), 102.5 (s, CH pz), 14.1 (s, CH_3_). ^195^Pt{^1^H} NMR (85.6 MHz, CD_2_Cl_2_, 298 K): δ −3211 (s).

### Preparation of **15**

3-(2-Pyridyl)-5-trifluoromethylpyrazole (107 mg, 0.5 mmol) was added to a pale yellow suspension of **6** (200 mg, 0.42 mmol) in acetone (7 mL), and the resulting yellow solution was stirred at room temperature for 1 h. After this time, the solvent was evaporated and the addition of diethyl ether (4 mL) afforded a yellowish-white solid that was washed with diethyl ether (3 × 4 mL) and dried under vacuum. Yield: 161 mg (57%). Anal. calcd for C_25_H_16_F_3_N_5_O_2_Pt: C, 44.78; H, 2.41; N, 10.44. Found: C, 44.39; H, 2.38; N, 10.33. HRMS (electrospray, *m*/*z*) calcd for C_25_H_17_F_3_N_5_O_2_Pt [M + H]^+^: 671.0979; found: 671.0995. IR (cm^–1^): ν(C=N), ν(C=C) 1615 (m), 1569 (m). NMR spectra of the solid in CD_2_Cl_2_ reveal the presence of isomers **15a**, **15b**, and **15c** in a 0.25:0.20:1 molar ratio at 298 K.

#### Spectroscopic Data for Isomer **15a**

^1^H NMR (500 MHz, CD_2_Cl_2_, 298 K): δ 8.42 (d, *J*_H–H_ = 4.4, 1H py), 8.37 (d, *J*_H–H_ = 8.1, 1H py), 7.80–7.74 (m, 2H py NCN), 7.73–7.64 (m, 2H py NCN), 7.56–7.50 (m, 1H py), 7.25–7.20 (m, 2H py NCN), 7.19–7.08 (m, 1H pz + 1H Ph), 7.07–6.99 (m, 1H py + 2H Ph), 6.77–6.73 (m, 2H py NCN). ^13^C{^1^H}-APT NMR (100.63 MHz, CD_2_Cl_2_, 298 K): δ 159.7 (s, C py NCN), 158.4 (s, C-Pt Ph), 154.3 (s, C pz), 154.2 (s, C py), 152.5 (s, C py) 151.3 (s, CH py NCN), 151.0 (s, C Ph), 149.4 (s, CH py), 141.2 (s, CH py NCN), 140.5 (q, *J*_C–F_ = 35, *C*-CF_3_), 136.1 (s, CH py), 125.9 (s, CH Ph), 124.2 (q, *J*_C–F_ = 268, CF_3_), 122.0 (s, CH py), 121.2 (s, CH py), 120.0 (s, CH py NCN), 115.8 (s, CH py NCN), 112.9 (s, CH Ph), 104.6 (s, CH pz). ^19^F{^1^H} NMR (376 MHz, CD_2_Cl_2_, 298 K): δ −60.5 (s). ^195^Pt{^1^H} NMR (85.6 MHz, CD_2_Cl_2_, 298 K): δ −3134 (s).

#### Spectroscopic Data for Isomer **15b**

^1^H NMR (500 MHz, CD_2_Cl_2_, 298 K): δ 8.56 (d, *J*_H–H_ = 3.7, 1H py), 8.05 (d, *J*_H–H_ = 8.0, 1H py), 7.90–7.83 (m, 2H py NCN), 7.73–7.64 (m, 2H py), 7.35–7.30 (3H, 2H py NCN + 1H pz), 7.19–7.08 (3H Ph), 7.07–6.99 (m, 2H py NCN), 6.82–6.77 (m, 2H py NCN). ^13^C{^1^H}-APT NMR (100.63 MHz, CD_2_Cl_2_, 298 K): δ 164.7 (s, C py), 159.7 (s, C py NCN), 158.4 (s, C-Pt Ph), 154.0 (s, C pz), 151.0 (s, C Ph), 150.9 (s, CH py NCN), 149.6 (s, CH py), 141.5 (s, CH py NCN), 140.5 (q, *J*_C–F_ = 35, *C*-CF_3_), 136.6 (s, CH py), 126.2 (s, CH Ph), 124.2 (q, *J*_C–F_ = 268, CF_3_), 122.0 (s, CH py), 120.1 (s, CH py), 116.1 (s, CH py NCN), 113.0 (s, CH Ph), 105.4 (s, CH pz). ^19^F{^1^H} NMR (376 MHz, CD_2_Cl_2_, 298 K): δ −59.0 (s). ^195^Pt{^1^H} NMR (85.6 MHz, CD_2_Cl_2_, 298 K): δ −3210 (s).

#### Spectroscopic Data for Isomer **15c**

^1^H NMR (500 MHz, CD_2_Cl_2_, 298 K): δ 9.20 (dd with ^195^Pt satellites, *J*_H–H_ = 6.0, 1.5, *J*_H–Pt_ = 41.3, 1H py), 8.80 (d with ^195^Pt satellites, *J*_H–H_ = 5.7, *J*_H–Pt_ = 46.1, 1H py), 8.03–7.99 (m, 1H py NCN), 7.99–7.94 (m, 1H py), 7.90–7.83 (m, 1H py), 7.61–7.56 (m, 1H py), 7.46–7.40 (m, 1H py NCN), 7.39–7.35 (m, 1H py), 7.25–7.20 (m, 1H py), 7.19–7.08 (m, 1H Ph), 7.07–6.99 (m, 1H py), 6.93 (dd, *J*_H–H_ = 7.8, 1.2, 1H Ph), 6.87 (s, 1H pz), 6.86–6.82 (m, 1H py NCN), 6.26 (dt, *J*_H–H_ = 8.3, 1H py NCN). ^13^C{^1^H}-APT NMR (100.63 MHz, CD_2_Cl_2_, 298 K): δ 164.7 (s, C py NCN), 162.1 (s, C py), 160.3 (s, C-Pt Ph), 159.7 (s, C py NCN), 158.4 (s, C Ph), 155.3 (s, C py), 154.6 (s with ^195^Pt satellites, *J*_C–Pt_ = 59.8, CH py), 153.7 (s, CH py), 151.0 (s, C pz), 147.7 (s, CH py NCN), 142.6 (q, *J*_C–F_ = 36, *C*-CF_3_), 142.0 (s, CH py), 139.6 (s, CH py), 139.2 (s, CH py NCN), 126.3 (s, CH Ph), 124.5 (q, *J*_C–F_ = 268, CF_3_),122.2 (s, CH py), 120.8 (s with ^195^Pt satellites, *J*_C–Pt_ = 35.9, CH py), 120.4 (s, C Ph), 119.3 (s, CH Ph), 119.1 (s, CH py), 119.0 (s, CH py), 118.4 (s, CH py NCN), 115.5 (s, CH py), 113.3 (s, CH Ph), 111.9 (s, CH py NCN), 102.1 (s, CH pz). ^19^F{^1^H} NMR (376 MHz, CD_2_Cl_2_, 298 K): δ −60.8 (s). ^195^Pt{^1^H} NMR (85.6 MHz, CD_2_Cl_2_, 298 K): δ −3233 (s).

### Preparation of **16**

2-(2-Pyridyl)-3,5-bis(trifluoromethyl)pyrrole (253 mg, 0.90 mmol) was added to a suspension of **4** (200 mg, 0.45 mmol) in acetone (7 mL), and the resulting mixture was stirred for 24 h at room temperature to get a yellow solution that was filtered through Celite and evaporated to dryness to get a yellow residue. This residue was extracted with diethyl ether (3 × 10 mL), and the combined extracts were evaporated under vacuum. Addition of pentane (5 mL) afforded a yellow solid that was washed with pentane (3 × 5 mL) and dried under vacuum. Yield: 100 mg (31%). Anal. calcd for C_27_H_16_F_6_N_4_Pt: C, 45.96; H, 2.28; N, 7.94. Found: C, 46.34; H, 2.31; N, 7.67. HRMS (electrospray, *m*/*z*) calcd for C_27_H_17_F_6_N_4_Pt [M + H]^+^: 706.1002; found: 706.1006. IR (cm^–1^): ν(C=N) 1608 (w). NMR spectra of the yellow solid in CD_2_Cl_2_ reveal the presence of a unique isomer. ^1^H NMR (300.13 MHz, CD_2_Cl_2_, 298 K): δ 8.24 (m, 1H, py), 8.03 (d with ^195^Pt satellites, *J*_H–H_ = 5.2, *J*_H–Pt_ = 41.9, 2H, py-NCN), 7.92 (m, 2H, py-NCN),7.74–7.64 (3H, 2H py-NCN + 1H py), 7.50–7.44 (3H, 2H Ph + 1H py), 7.22 (t, *J*_H–H_ = 7.7, 1H, Ph), 7.18 (m, 2H, py-NCN), 7.06 (s, 1H, pyrrolate), 6.97 (m, 1H, py). ^13^C{^1^H} NMR (75.48 MHz, CD_2_Cl_2_, 298 K): δ 168.0 (s with ^195^Pt satellites, *J*_C–Pt_ = 98.9, C py-NCN), 163.4 (s, Pt–C), 155.3 (s, Cpy), 152.5 (s, CH py-NCN), 148.9 (s, CH py), 142.5 (s, C pyrrolate), 142.4 (s, C Ph), 139.5 (s, CH py-NCN), 135.7 (s, CH py), 128.6 (q, *J*_C–F_ = 35.6, *C*-CF_3_), 125.6 (q, *J*_C–F_ = 267.0, CF_3_), 124.4 (s, CH py), 124.3 (s with ^195^Pt satellites, *J*_C–Pt_ = 33.1, CH py-NCN), 123.8 (q, *J*_C–F_ = 266.0, CF_3_), 123.7 (s with ^195^Pt satellites, *J*_C–Pt_ = 31.2, CH py-NCN), 121.9 (s, CH py), 119.7 (s with ^195^Pt satellites, *J*_C–Pt_ = 48.5, CH py-NCN), 112.7 (q, *J*_C–F_ = 35.5, *C*-CF_3_), 110.8 (s, CH pyrrolate). ^19^F{^1^H} NMR (282.40 MHz, CD_2_Cl_2_, 298 K): δ −53.0, −58.2 (both s, CF_3_). ^195^Pt{^1^H} NMR (85.6 MHz, CD_2_Cl_2_, 298 K): δ −3554 (s).

### Preparation of **17**

2-(2-Pyridyl)-3,5-bis(trifluoromethyl)pyrrole (119 mg, 0.424 mmol) was added to a suspension of **5** (200 mg, 0.424 mmol) in acetone (7 mL), and the resulting mixture was stirred for 1 h at room temperature to get a yellow solution that was evaporated to dryness to get a yellow residue. This residue was extracted with diethyl ether (3 × 10 mL), and the combined extracts were evaporated under vacuum. Addition of pentane (5 mL) afforded a yellow solid that was washed with pentane (3 × 5 mL) and dried under vacuum. Yield: 167 mg (54%). Crystals of **17** suitable for X-ray diffraction analysis were obtained at 4 °C by vapor diffusion of pentane into an acetone solution of the complex. Anal. calcd for C_29_H_20_F_6_N_4_Pt: C, 47.48; H, 2.75; N, 7.64. Found: C, 47.11; H, 2.85; N, 7.77. HRMS (electrospray, *m*/*z*) calcd for C_29_H_21_F_6_N_4_Pt [M + H]^+^: 734.1315; found: 734.1304. IR (cm^–1^): ν(C=N), ν(C=C) 1597 (w), 1543 (m). NMR spectra of the yellow solid in CD_2_Cl_2_ reveal the presence of a unique isomer. ^1^H NMR (300.13 MHz, CD_2_Cl_2_, 298 K): δ 8.26 (m, 1H, py), 8.04 (d with ^195^Pt satellites, *J*_H–H_ = 5.8, *J*_H–Pt_ = 42.0, 2H, py-NCN), 7.94–7.77 (m, 4H, py-NCN), 7.63 (d, *J*_H–H_ = 7.9, 1H, py), 7.46 (td, *J*_H–H_ = 7.9, 1.8, 1H, py), 7.12 (m, 2H, py-NCN), 7.05 (s, 1H, pyrrole), 6.97 (ddd, *J*_H–H_ = 7.6, 4.8, 1.0, 1H, py), 6.83 (s, 1H, Ph), 2.62 (s, 6H, CH_3_). ^13^C{^1^H} NMR (75.48 MHz, CD_2_Cl_2_, 298 K): δ 168.8 (s with ^195^Pt satellites, *J*_C–Pt_ = 110.7, C py-NCN), 165.6 (s with ^195^Pt satellites, *J*_C–Pt_ = 101.9, C Ph), 155.4 (s, C py), 152.2 (s, CH py-NCN), 148.9 (s, CH py), 142.2 (s, C pyrrolate), 139.2 (s, CH py-NCN), 138.7 (s, C py), 137.1 (s with ^195^Pt satellites, *J*_C–Pt_ = 35.6, C Ph), 135.6 (s, CH py), 131.5 (s, CH Ph), 128.5 (q, *J*_C–F_ = 36.1, *C*-CF_3_), 125.7 (q, *J*_C–F_ = 267.0, CF_3_), 124.6 (s, CH py), 123.8 (q, *J*_C–F_ = 268.0, CF_3_), 122.8 (s with ^195^Pt satellites, *J*_C–Pt_ = 48.3, CH py-NCN), 122.7 (s with ^195^Pt satellites, *J*_C–Pt_ = 31.1, CH py-NCN), 121.9 (s, CH py), 112.8 (q, *J*_C–F_ = 35.3, *C*-CF_3_), 110.8 (s, CH pyrrolate), 22.0 (s, CH_3_). ^19^F{^1^H} NMR (282.40 MHz, CD_2_Cl_2_, 298 K): δ −53.0, −58.1 (both s, CF_3_). ^195^Pt{^1^H} NMR (85.6 MHz, CD_2_Cl_2_, 298 K): δ −3537 (s).

### Preparation of **18**

2-(2-Pyridyl)-3,5-bis(trifluoromethyl)pyrrole (140 mg, 0.5 mmol) was added to a suspension of **6** (200 mg, 0.42 mmol) in acetone (7 mL), and the resulting mixture was stirred for 1 h at room temperature to get a yellow solution that was evaporated to dryness to get a pale yellow residue. This residue was extracted with diethyl ether (3 × 10 mL), and the combined extracts were evaporated under vacuum. Addition of pentane (5 mL) afforded a yellowish-white solid that was washed with pentane (3 × 5 mL) and dried under vacuum. Yield: 156 mg (50%). Anal. calcd for C_27_H_16_F_6_N_4_O_2_Pt: C, 43.97; H, 2.19; N, 7.60. Found: C, 43.58; H, 2.16; N, 7.52. HRMS (electrospray, *m*/*z*) calcd for C_27_H_16_F_6_N_4_NaO_2_Pt [M + Na]^+^: 760.0720; found: 760.0686. IR (cm^–1^): ν(C=N), ν(C=C) 1614 (m), 1567 (w). NMR spectra of the solid in CD_2_Cl_2_ reveal the presence of a unique isomer. ^1^H NMR (300.13 MHz, CD_2_Cl_2_, 298 K): δ 8.45 (m, 1H, py), 8.00 (dd with ^195^Pt satellites, *J*_H–H_ = 6.2, 1.9, *J*_H–Pt_ = 46.2, 2H, py-NCN), 7.83 (m, 2H, py-NCN), 7.39 (m, 1H, py), 7.17 (m, 2H, py-NCN), 7.11–6.86 (m, 8H, py, pyrrolate, NCN). ^13^C{^1^H} NMR (100.62 MHz, CD_2_Cl_2_, 298 K): δ 159.7 (s, C py-NCN), 155.3 (s, C py), 153.8 (s, C-Pt), 151.8 (s, CH py-NCN), 149.3 (s, CH py), 141.5 (s, C pyrrolate), 141.2 (s, CH py-NCN), 135.5 (s, CH py), 127.2 (q, *J*_C–F_ = 36.2, *C*-CF_3_), 125.7 (s, CH Ph-NCN), 125.3 (q, *J*_C–F_ = 266.0, CF_3_), 124.4 (s, CH py), 123.5 (q, *J*_C–F_ = 266.0, CF_3_), 122.1 (s, CH py), 120.0 (s, CH py-NCN), 115.5 (s, CH py-NCN), 112.9 (s, CH Ph-NCN), 112.8 (q, *J*_C–F_ = 36.0, *C*-CF_3_), 111.1 (m, CH pyrrolate), 104.4 (s, C Ph). ^19^F{^1^H} NMR (282.40 MHz, CD_2_Cl_2_, 298 K): δ −53.2, −58.9 (both s, CF_3_). ^195^Pt{^1^H} NMR (85.6 MHz, CD_2_Cl_2_, 298 K): δ −3163 (s).

## Supporting Information Available

General information for the experimental section; NMR spectra; equilibrium and kinetic analysis for complexes **11** and **12**; structural analysis of complexes **9**, **12b**, **15c**, **17**, and **18**; computational details; energies of optimized structures; observed and calculated UV–vis spectra of complexes **7**–**10** and **16**–**18**; analysis of computed UV/vis data; theoretical analysis of molecular orbitals; cyclic voltammograms of complexes **7**–**10** and **16**–**18**; and photophysical studies (PDF)
Atomic coordinates of optimized complexes (XYZ)

## Supplementary Material

ic3c00694_si_001.pdf

ic3c00694_si_002.xyz
