# Supplementary material for: Competition between N,C,N-Pincer and N,N-Chelate Ligands in Platinum(II)
Source: Inorg Chem. 2023 Jun 21;62(26):10152–70. doi: 10.1021/acs.inorgchem.3c00694 (PMC11003652; doi:10.1021/acs.inorgchem.3c00694)
Supplement: Supplementary file 1 — ic3c00694_si_001.pdf [file ic3c00694_si_001.pdf]

SUPPORTING INFORMATION

Competition between N,C,N-Pincer and N,N-Chelate Ligands in Platinum(II)

Miguel A. Esteruelas,* Sonia Moreno-Blázquez, Montserrat Oliván, and Enrique Oñate

Departamento de Química Inorgánica – Instituto de Síntesis Química y Catálisis Homogénea (ISQCH) – Centro de Innovación en Química Avanzada (ORFEO-CINQA), Universidad de Zaragoza – CSIC, 50009 Zaragoza, Spain

* e-mail: maester@unizar.es

Contents:

- Experimental Section: General Information	S2
- NMR spectra	S4
- Equilibrium and Kinetic Analysis for Complexes **11** and **12**	S56
- Structural Analysis of Complexes **9**, **12b**, **15c**, **17**, and **18**	S61
- Computational Details and Energies of Calculated Complexes	S63
- UV-vis Spectra (Observed and Calculated) and Selected Excited Electronic Configurations for the Calculated Spectra of Complexes **7-10** and **16-18**	S70
- Theoretical Analysis of Molecular Orbitals of Complexes **7-10** and **16-18**	S77
- Cyclic Voltammograms	S91
- Photophysical Studies	S95
- References	S189

● **Experimental Section: General Information.**

All reactions were carried out with exclusion of air using Schlenk-tube techniques or in a drybox. Pentane and diethyl ether were obtained oxygen- and water-free from an MBraun solvent purification apparatus, while acetone and tetrahydrofuran were dried and distilled under argon prior to use. ^{1}H, $^{13}C\{^{1}H\}$, $^{19}F\{^{1}H\}$, and $^{195}Pt\{^{1}H\}$ NMR spectra were recorded on Bruker 300 ARX, Bruker Avance 300 MHz, Bruker Avance 400 MHz, or Bruker Avance 500 MHz instruments. Chemical shifts (expressed in ppm) are referenced to residual solvent peaks (^{1}H, $^{13}C\{^{1}H\}$), external $CFCl_3$ ($^{19}F\{^{1}H\}$) or Na_2PtCl_6 ($^{195}Pt\{^{1}H\}$). Coupling constant *J* are given in hertz. Attenuated total reflection infrared spectra (ATR-IR) of solid samples were run on a PerkinElmer Spectrum 100 FT-IR spectrometer. C, H, and N analyses were carried out in a PerkinElmer 2400 CHNS/O analyzer. High-resolution electrospray mass spectra were acquired using a MicroTOF-Q hybrid quadrupole time-of-flight spectrometer (Bruker Daltonics, Bremen, Germany). UV-visible spectra were recorded on an Evolution 600 spectrophotomer. Steady-state photoluminescence spectra were recorded with either a Jobin-Yvon Horiba Fluorolog FL-3-11 Tau 3 spectrometer (PMMA films) or with a PicoQuant FluoTime 300 spectrometer (CH_2Cl_2 solutions). Lifetime measurements were performed at the maximum emission wavelength of the complexes either on a Jobin-Yvon Horiba Fluorolog FL-3-11 Tau 3 spectrometer (PMMA films) or a PicoQuant FluoTime 300 spectrometer (CH_2Cl_2 solutions). Data were fitted to either monoexponential or biexponential functions. Relative amplitudes are given in parentheses for biexponential decays. Quantum yields were measured using the Hamamatsu Absolute PL Quantum Yield Measurement System C11347-11 (an uncertainty of ±5% is estimated). PMMA films at 5 wt % were prepared in a glove box dissolving 1 mg of compound and 19 mg of PMMA (average Mw 97,000, average Mn 46,000) in CH_2Cl_2 (1 mL), while PMMA films at 2 wt % were prepared in a glove box by dissolving 1 mg of compound and 49 mg of PMMA in CH_2Cl_2 (1 mL). The solutions were filtered through a PTFE syringe filter (0.22 micron pore size, 17 mm diameter) and then drop-coated onto the quartz plates and dried. The CH_2Cl_2 solutions were prepared in a glove box filled with argon. Cyclic voltammetry measurements were performed using a Voltalab PST050 potentiostat with Pt wire as working electrode, Pt wire as counter electrode, and saturated calomel (SCE) as reference electrode. The experiments were carried out under argon in dichloromethane solutions (10^{-3} M), with $[Bu_4N]PF_6$ as supporting electrolyte (0.1 M). Scan rate was 100 mV s^{-1}. The potentials

were referenced to the ferrocene/ferrocenium (Fc/Fc^{+}) couple. PtCl{κ^3-*N,C,N*-[py-C_6H_3-py]} (**1**),[1] PtCl{κ^3-*N,C,N*-[py-C_6HMe_2-py]} (**2**)[2] and PtCl{κ^3-*N,C,N*-[py-O-C_6H_3-O-py]}) (**3**)[3] were prepared according to the reported procedures.

● **NMR Spectra**

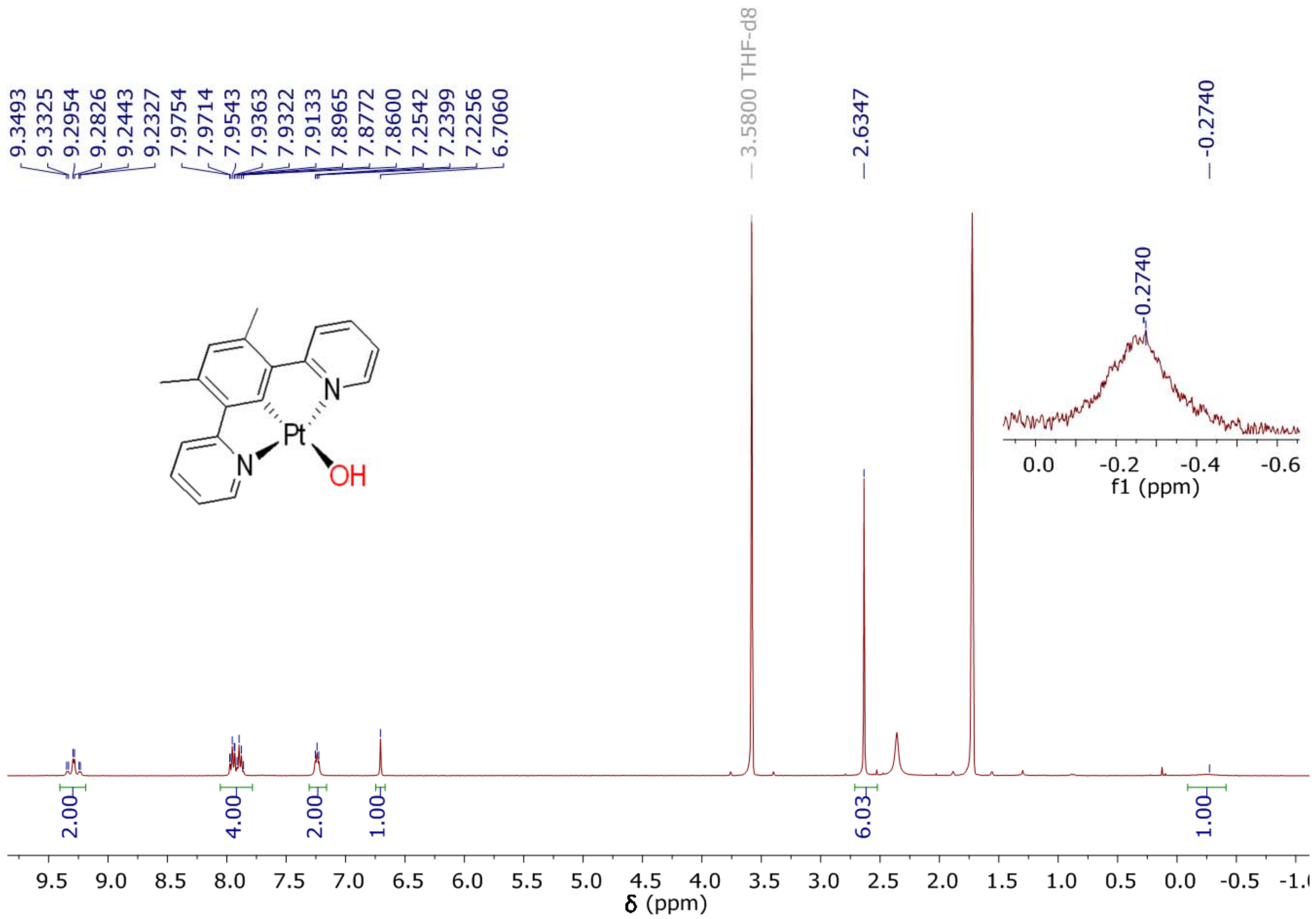

Figure S1. ^{1}H NMR spectrum (400.1 MHz, THF-d_8, 338 K) of compound **5**.

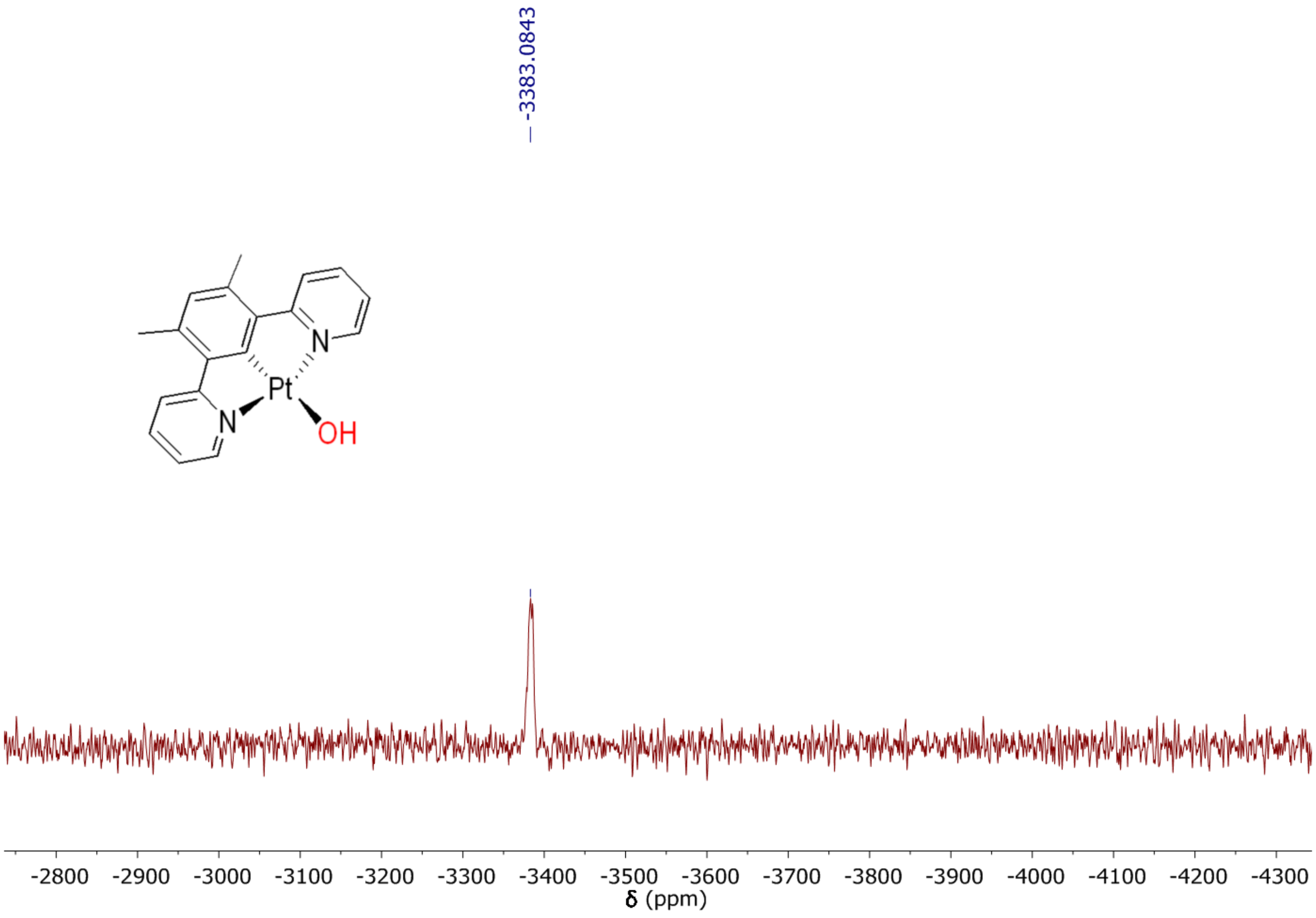

Figure S2. ^{195}Pt{^{1}H} NMR spectrum (85.6 MHz, THF-d_8, 298 K) of compound **5**.

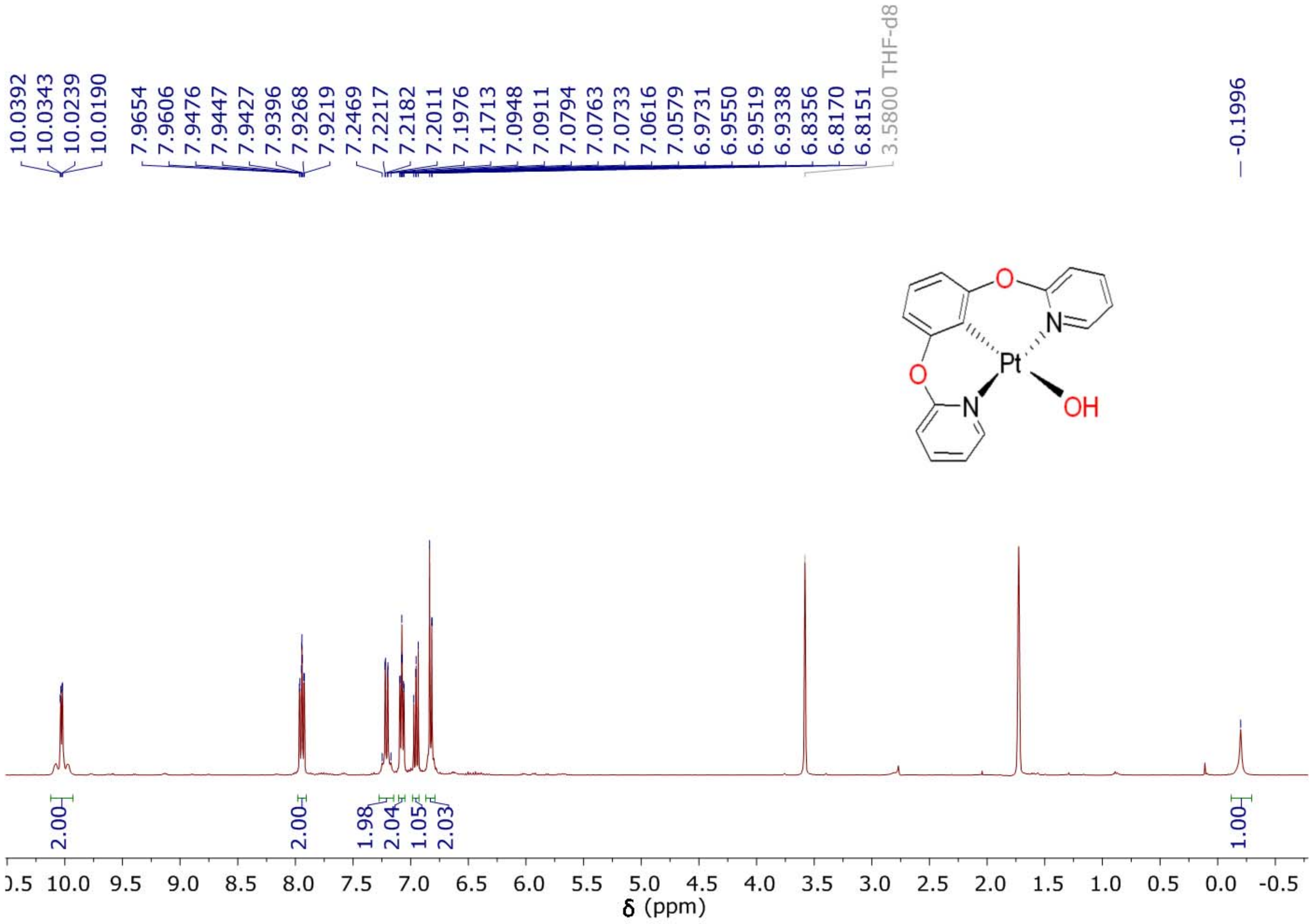

Figure S3. ^{1}H NMR spectrum (400.1 MHz, THF-d_8, 298 K) of compound **6**.

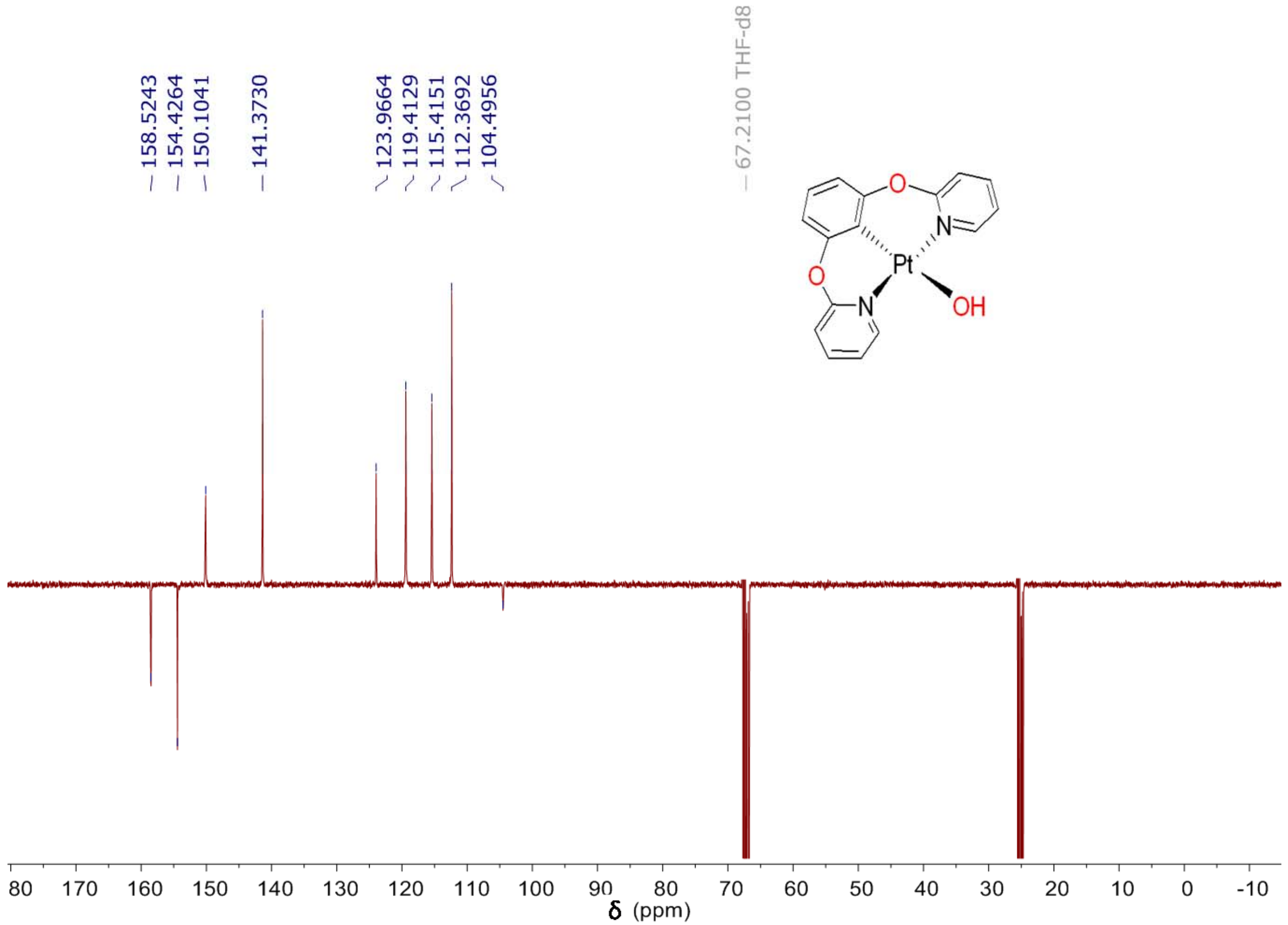

Figure S4. $^{13}C\{^{1}H\}$-apt NMR spectrum (100.63 MHz, THF-d_8, 223 K) of compound **6**.

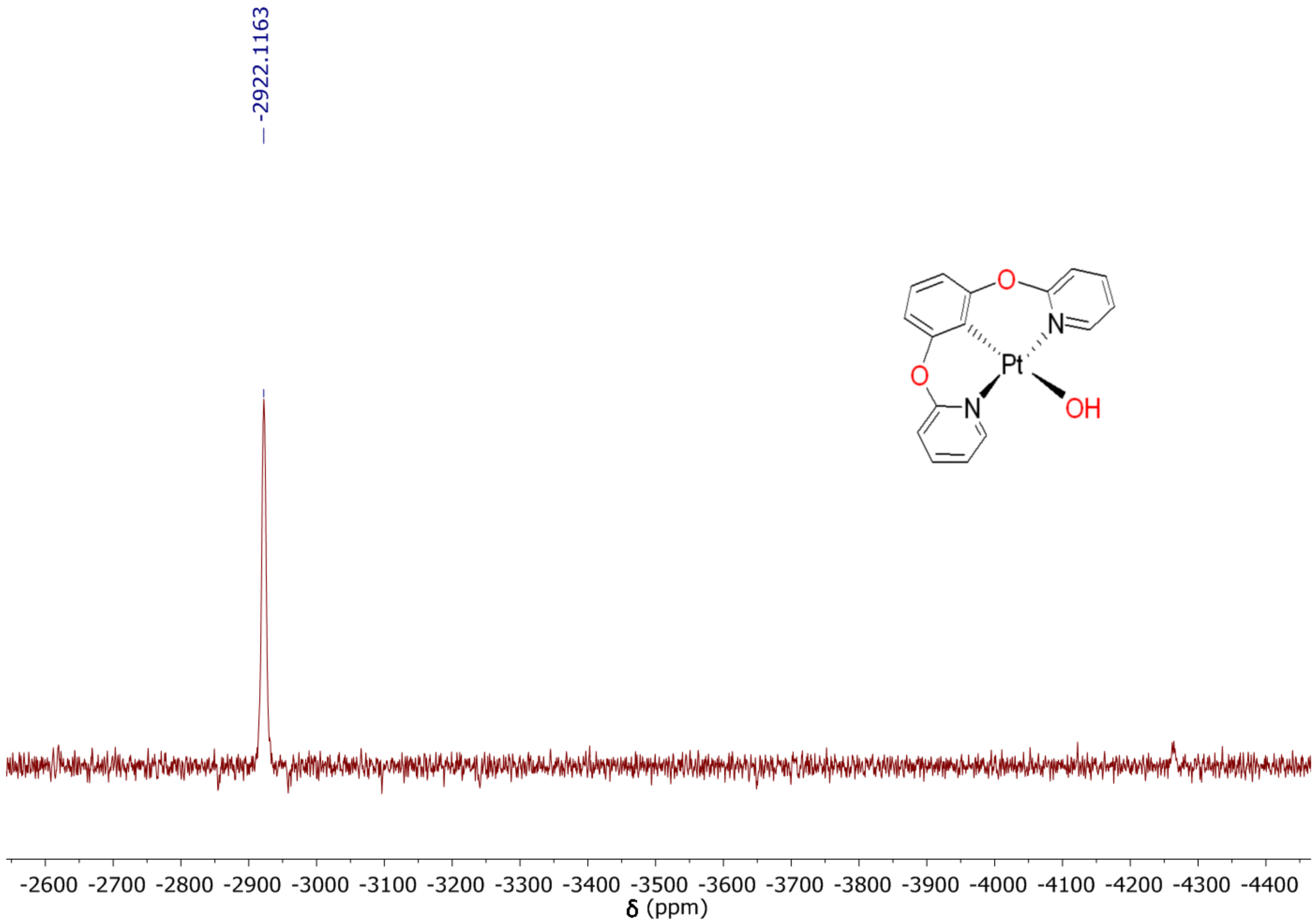

Figure S5. ^{195}Pt{^{1}H} NMR spectrum (85.6 MHz, THF-d_8, 223 K) of compound **6**.

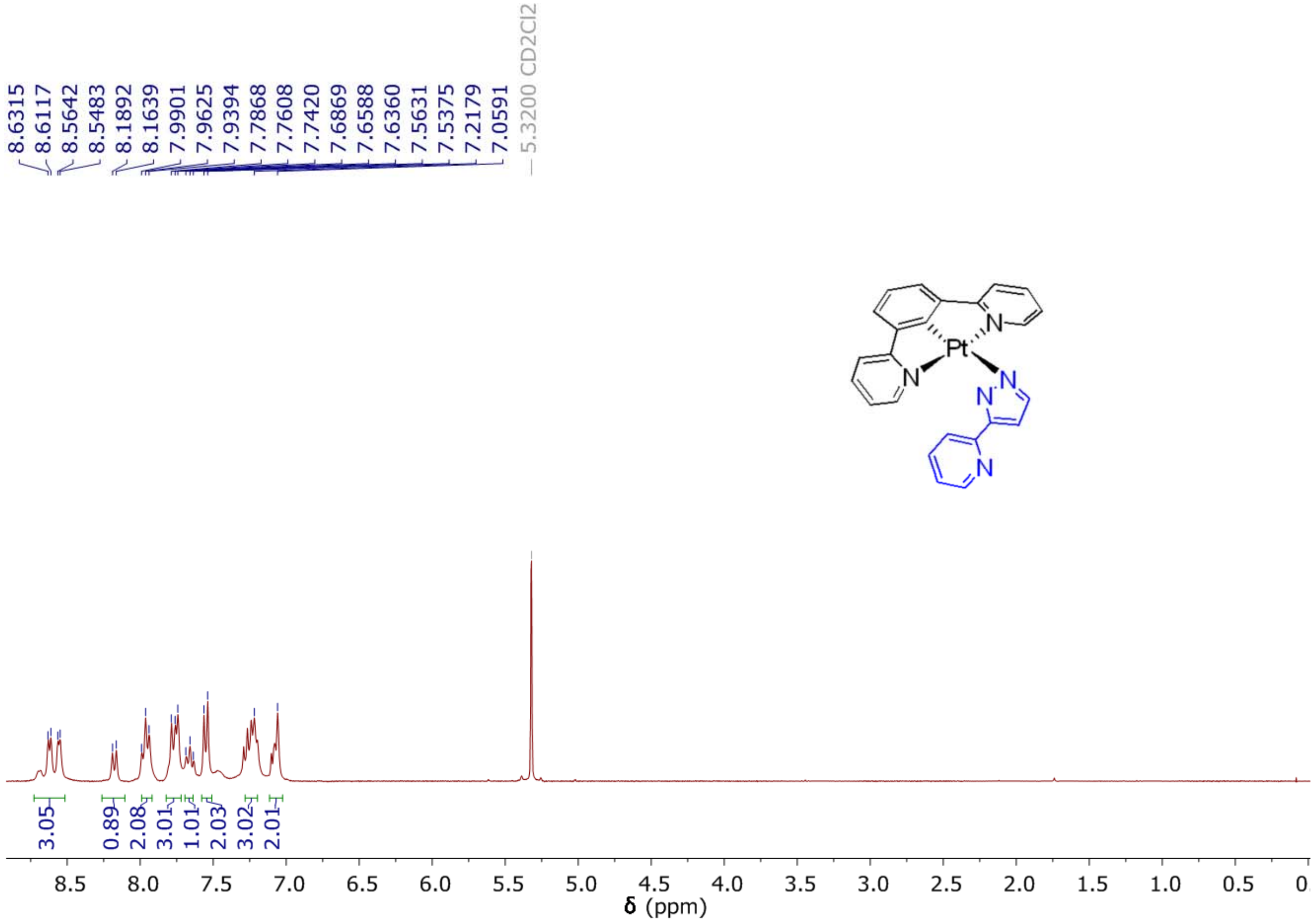

Figure S6. ^{1}H NMR spectrum (300.13 MHz, CD_2Cl_2, 298 K) of compound **7**.

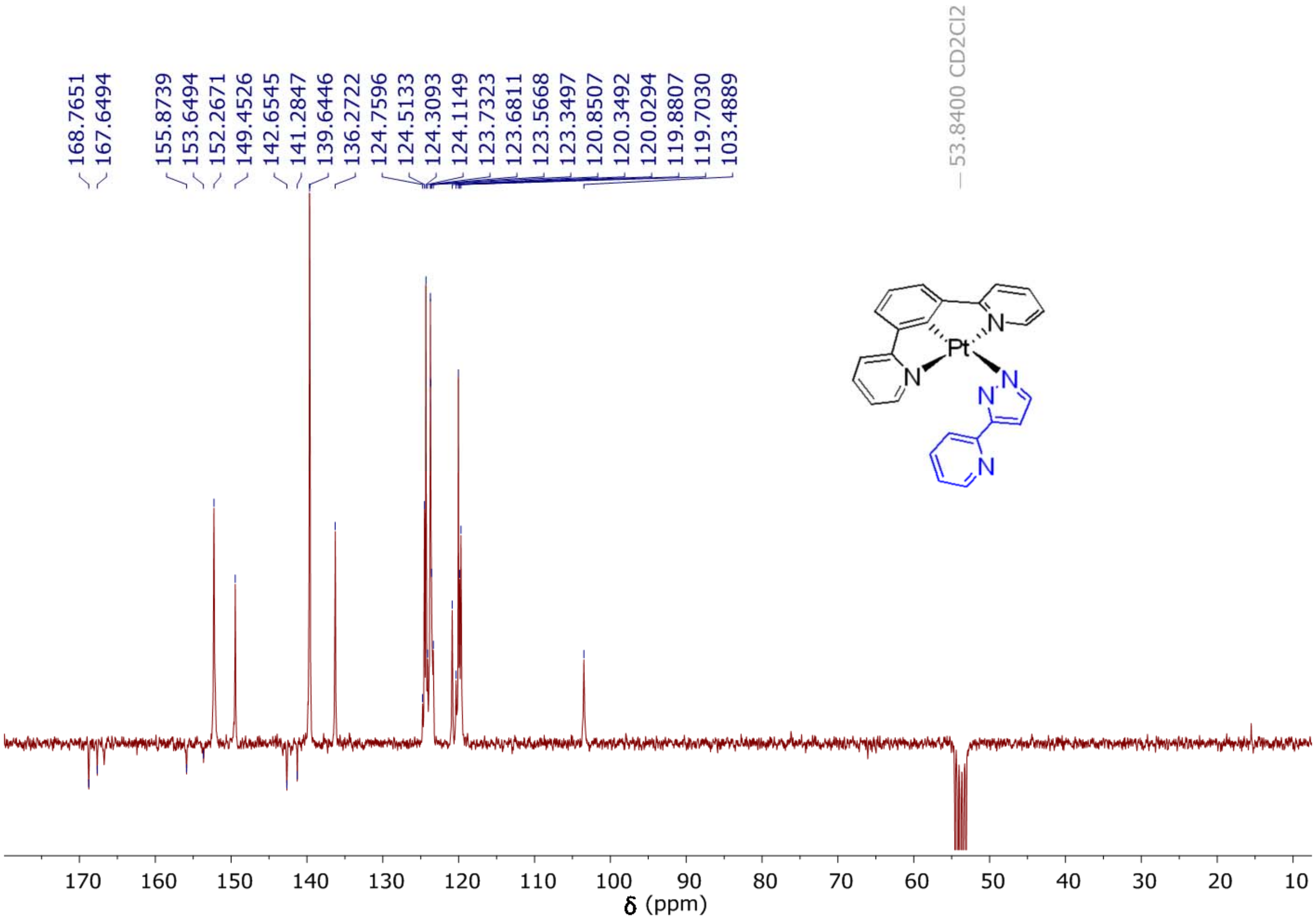

Figure S7. $^{13}C\{^{1}H\}$-apt NMR spectrum (75.48 MHz, CD_2Cl_2, 298 K) of compound **7**.

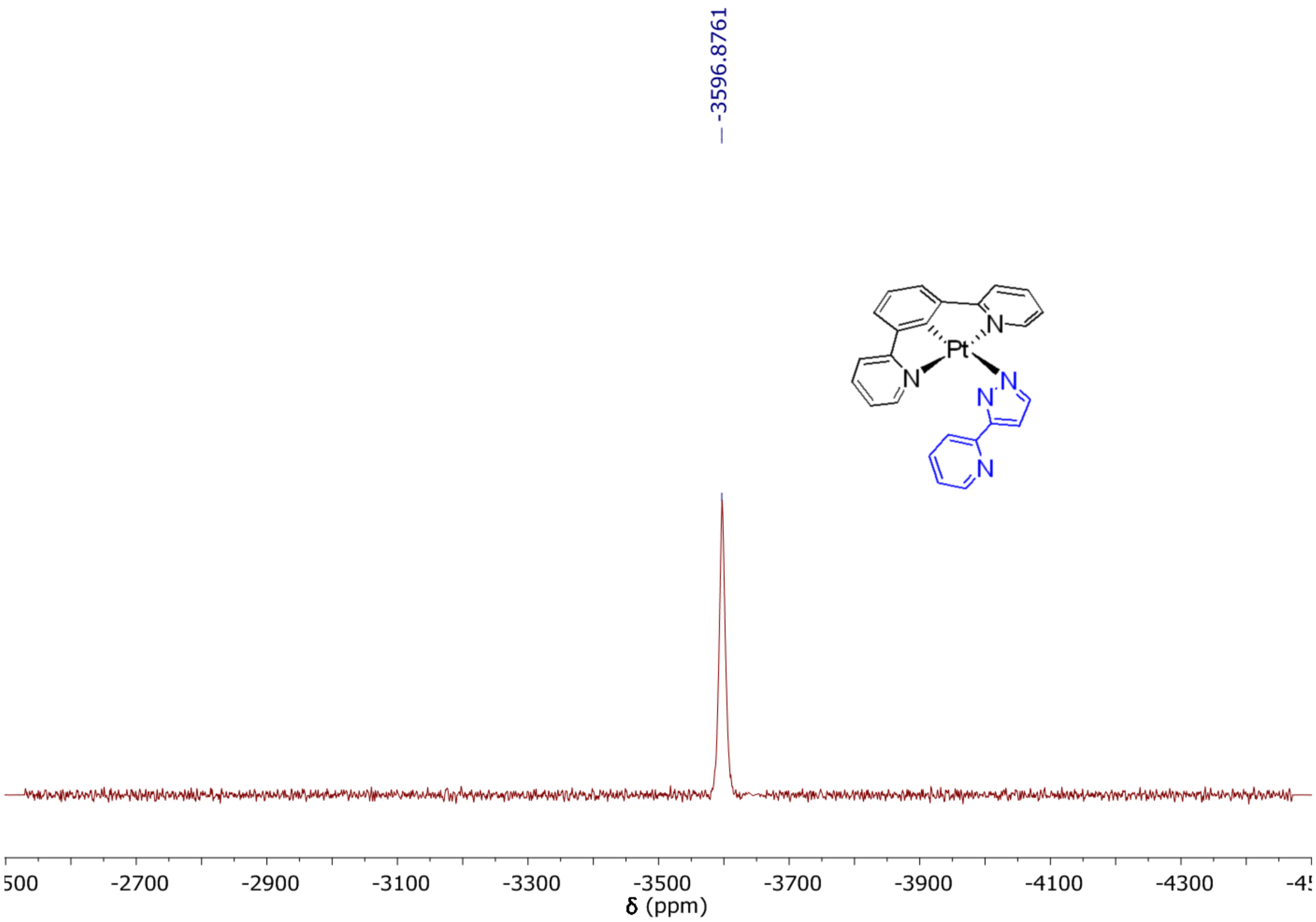

Figure S8. $^{195}Pt\{^1H\}$ NMR spectrum (85.6 MHz, CD_2Cl_2, 298 K) of compound **7**.

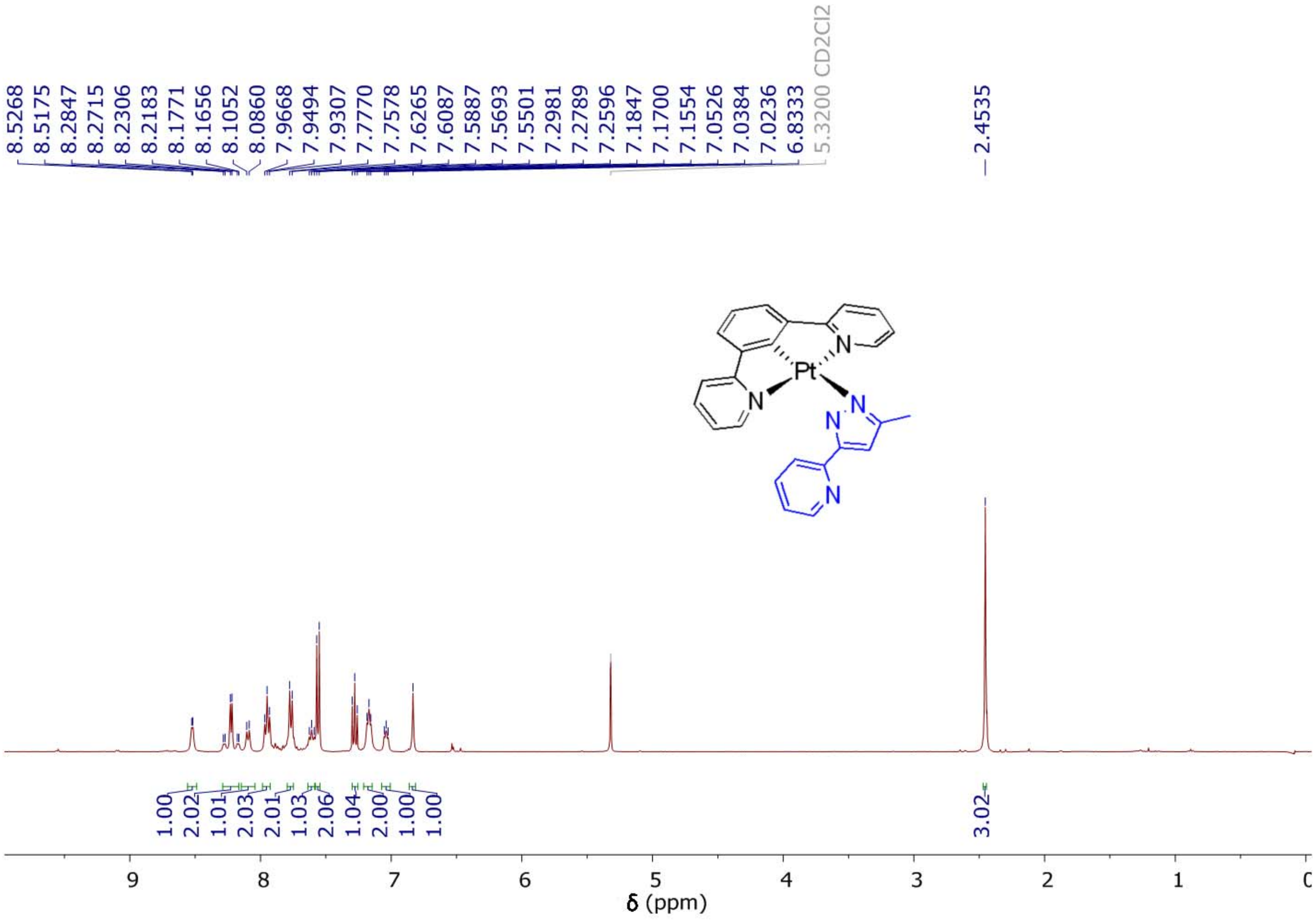

Figure S9. ^{1}H NMR spectrum (400.1 MHz, CD_2Cl_2, 298 K) of compound **8.**

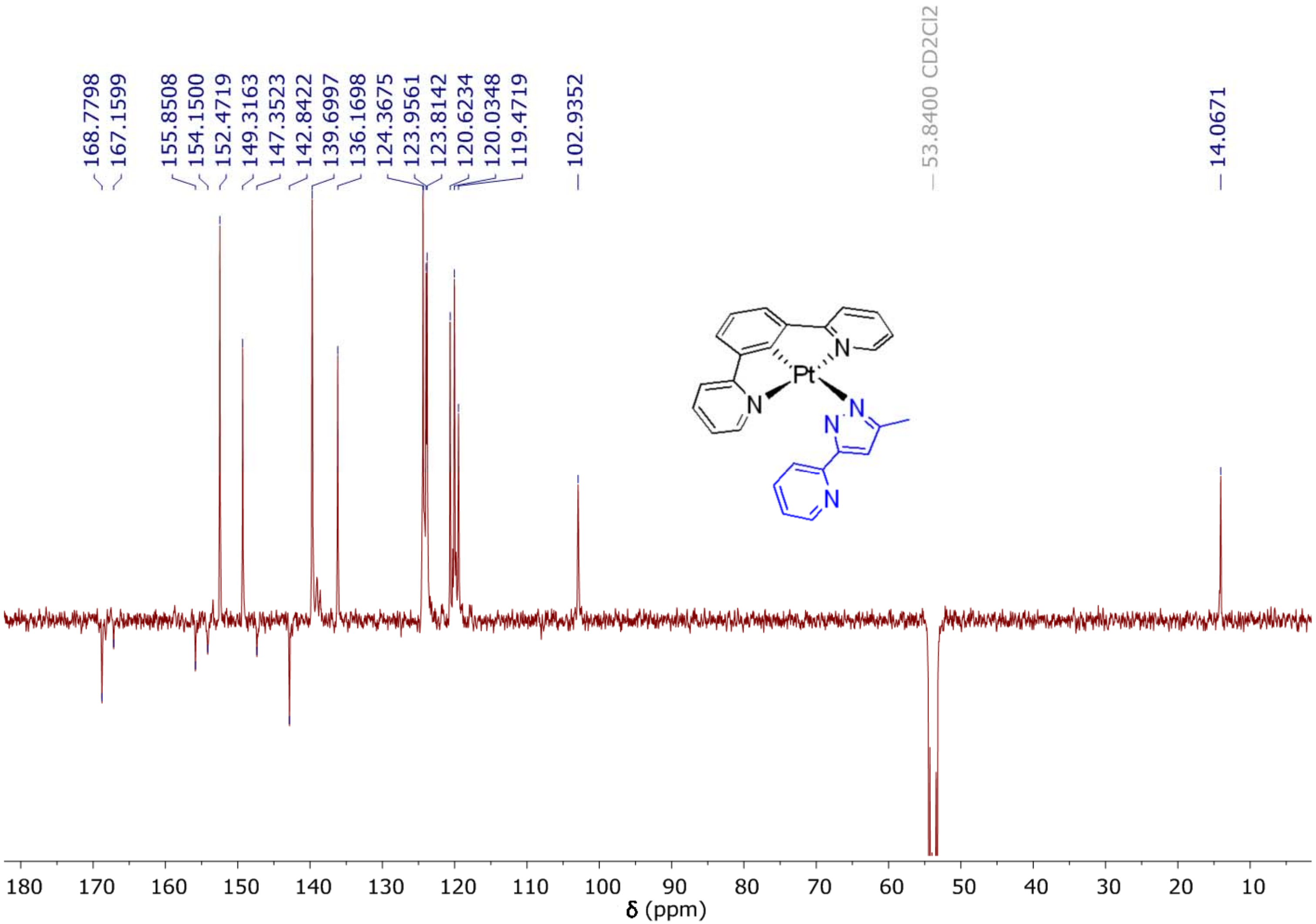

Figure S10. $^{13}C\{^{1}H\}$-apt NMR spectrum (100.63 MHz, CD_2Cl_2, 298 K) of compound **8**.

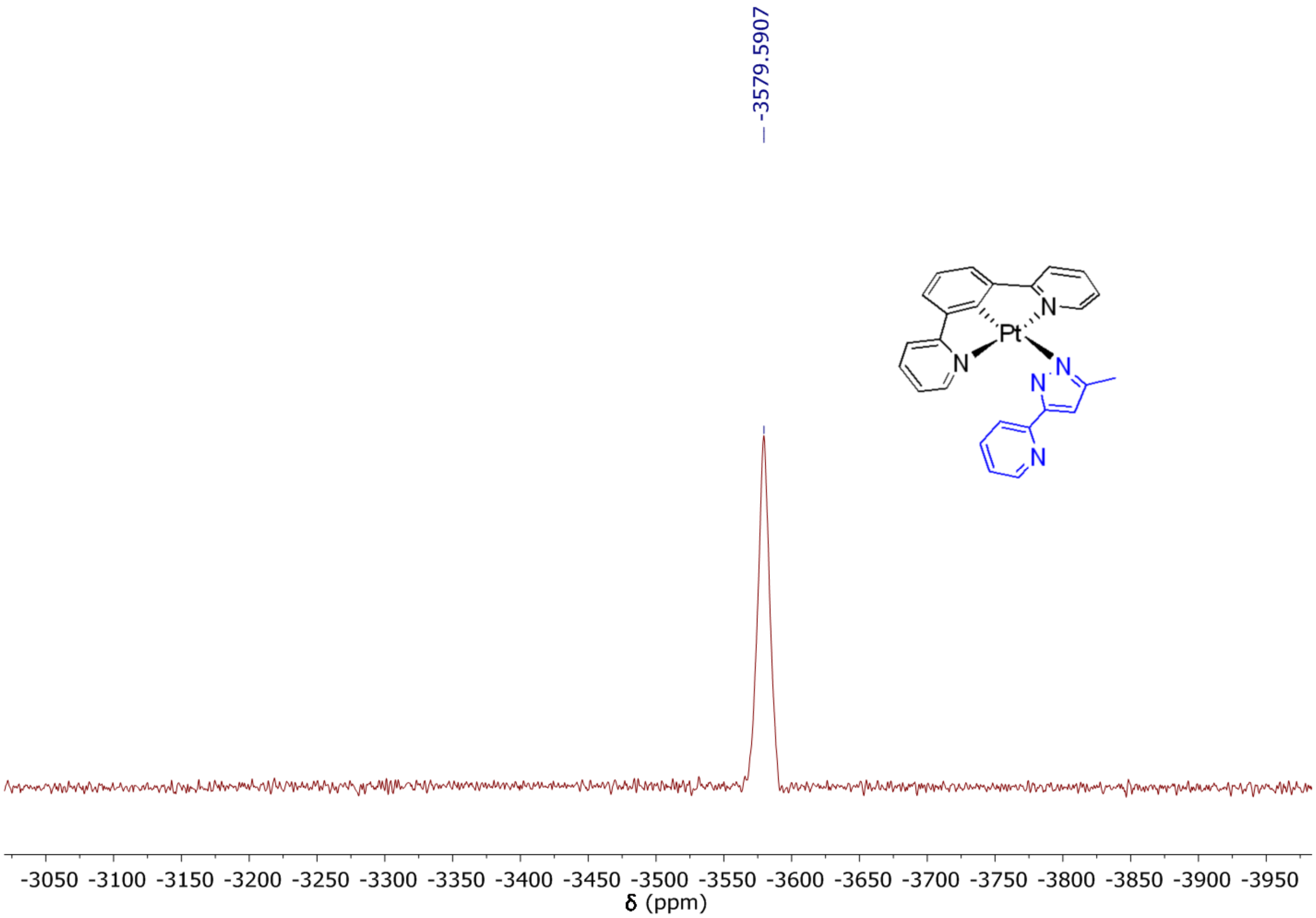

Figure S11. $^{195}Pt\{^{1}H\}$ NMR spectrum (85.6 MHz, CD_2Cl_2, 298 K) of compound **8**.

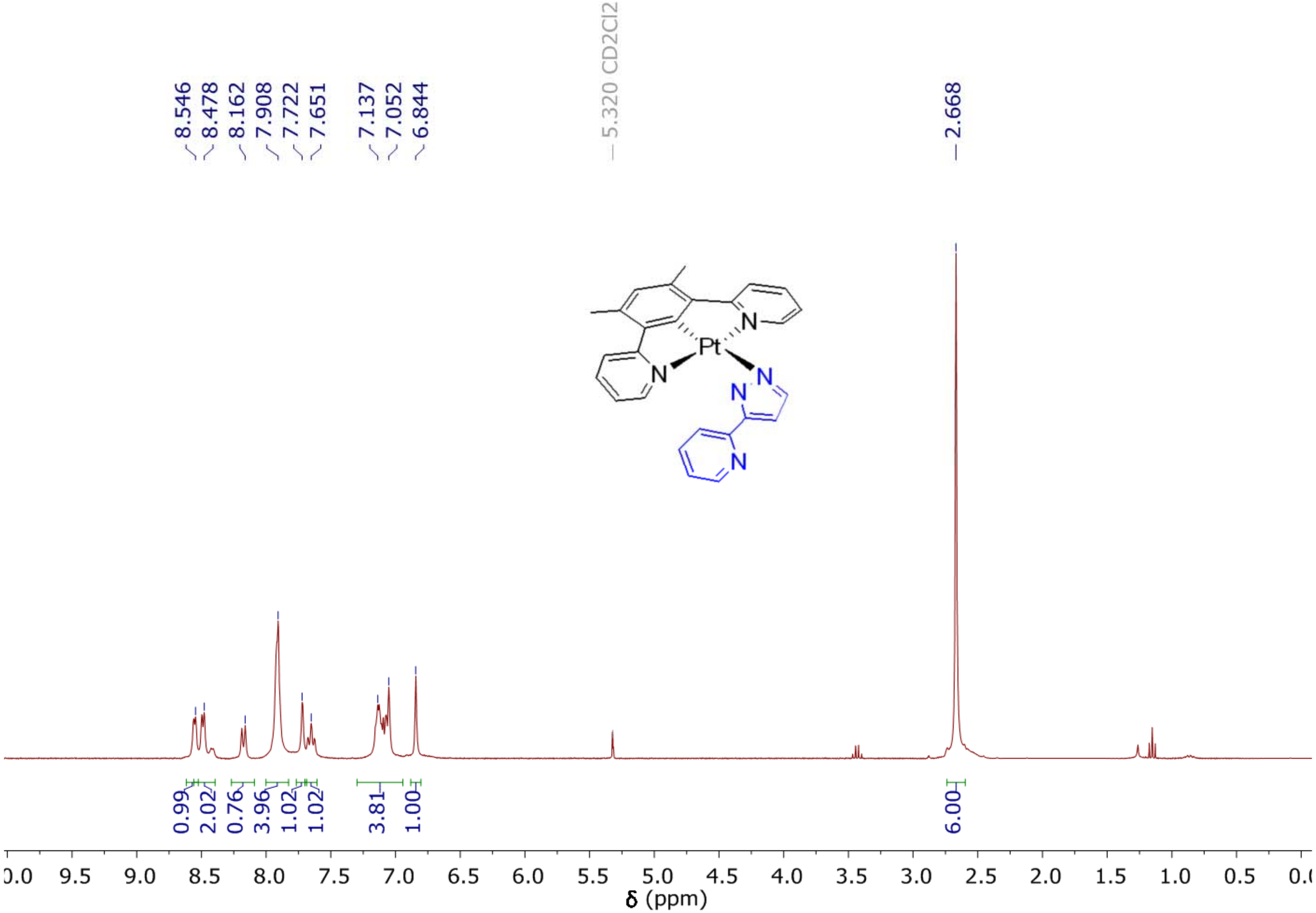

Figure S12. ^{1}H NMR spectrum (300.13 MHz, CD_2Cl_2, 298 K) of compound **9.**

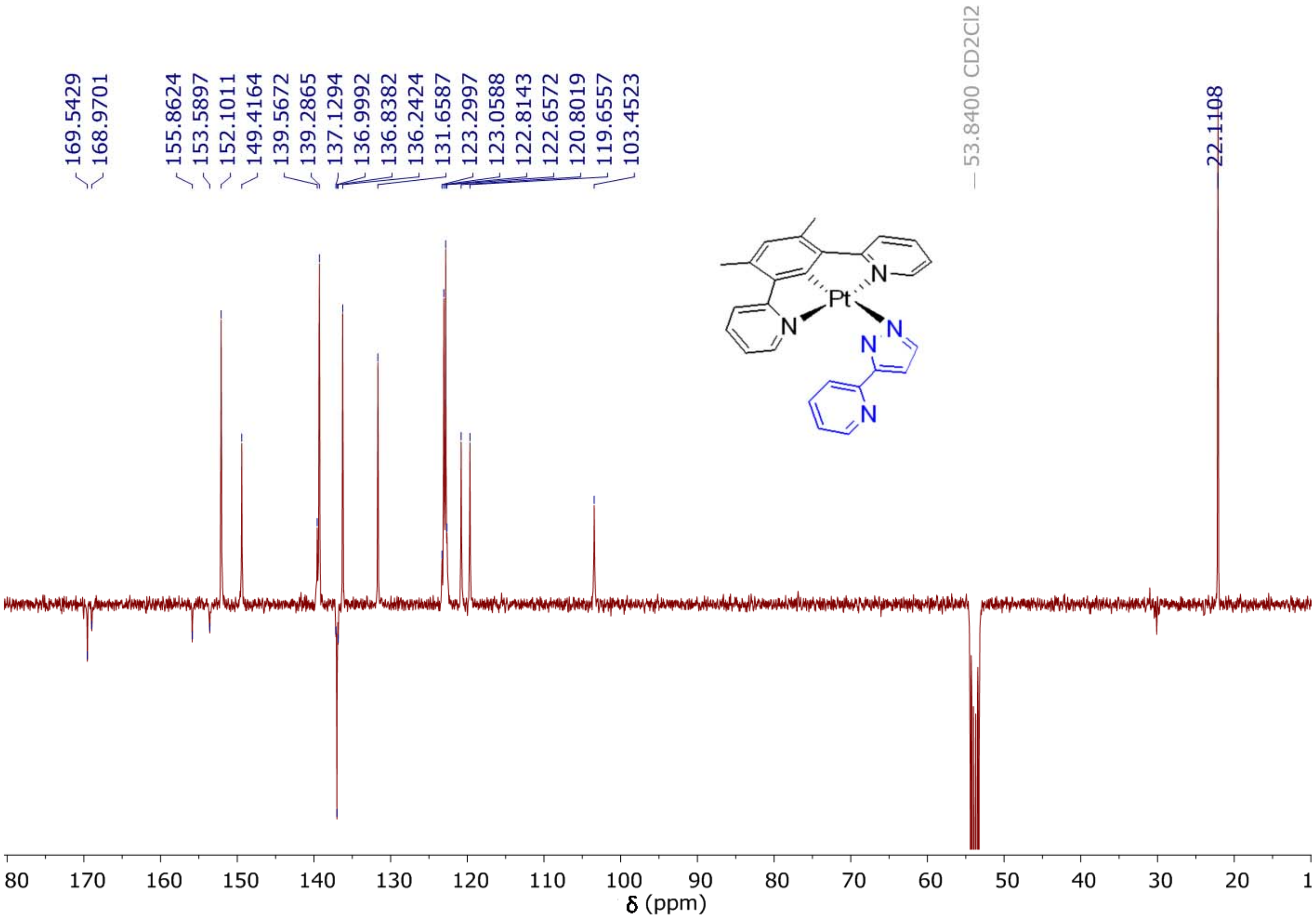

Figure S13. $^{13}C\{^{1}H\}$-apt NMR spectrum (100.63 MHz, CD_2Cl_2, 298 K) of compound **9**.

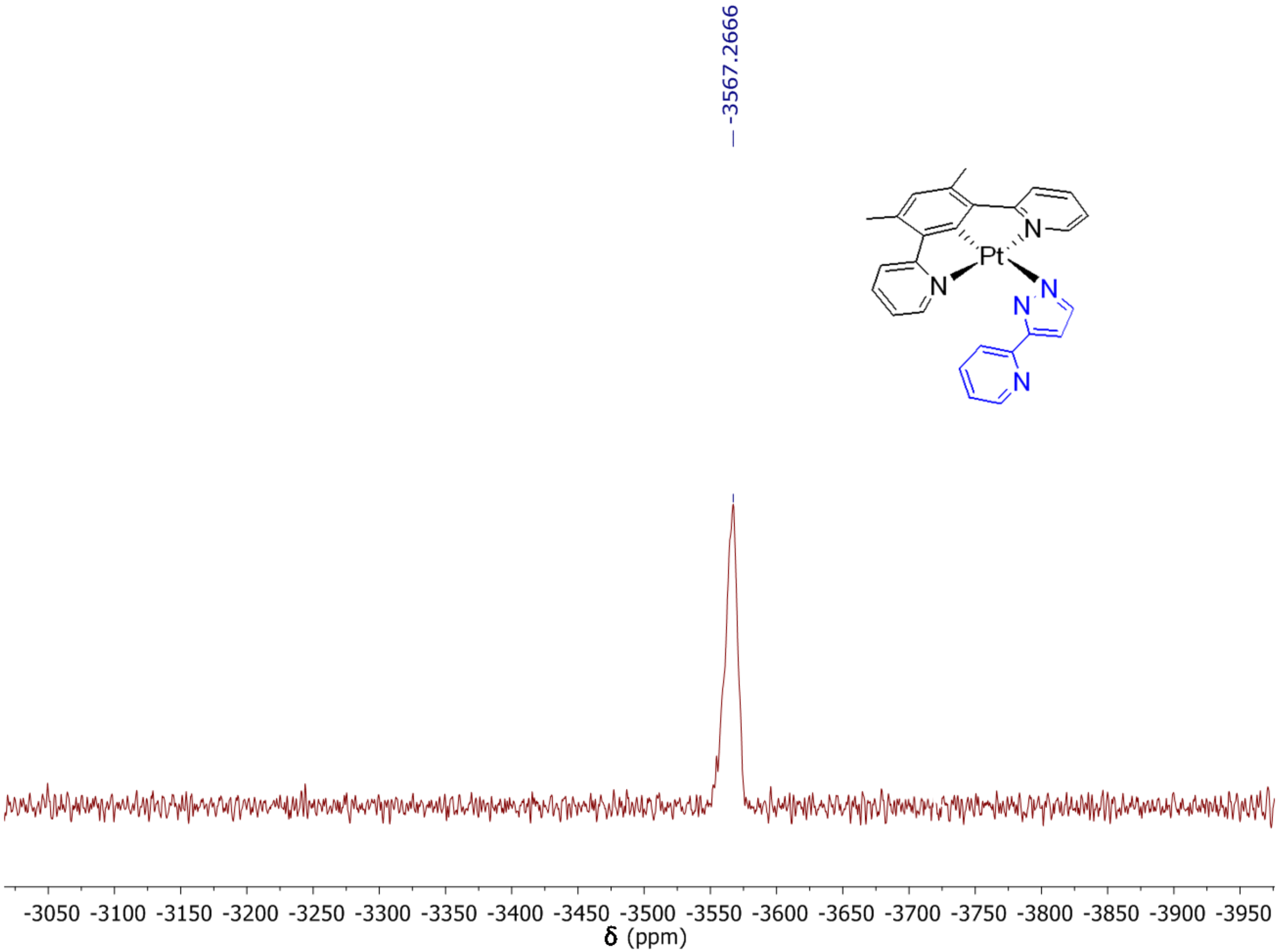

Figure S14. $^{195}Pt\{^1H\}$ NMR spectrum (85.6 MHz, CD_2Cl_2, 298 K) of compound **9**.

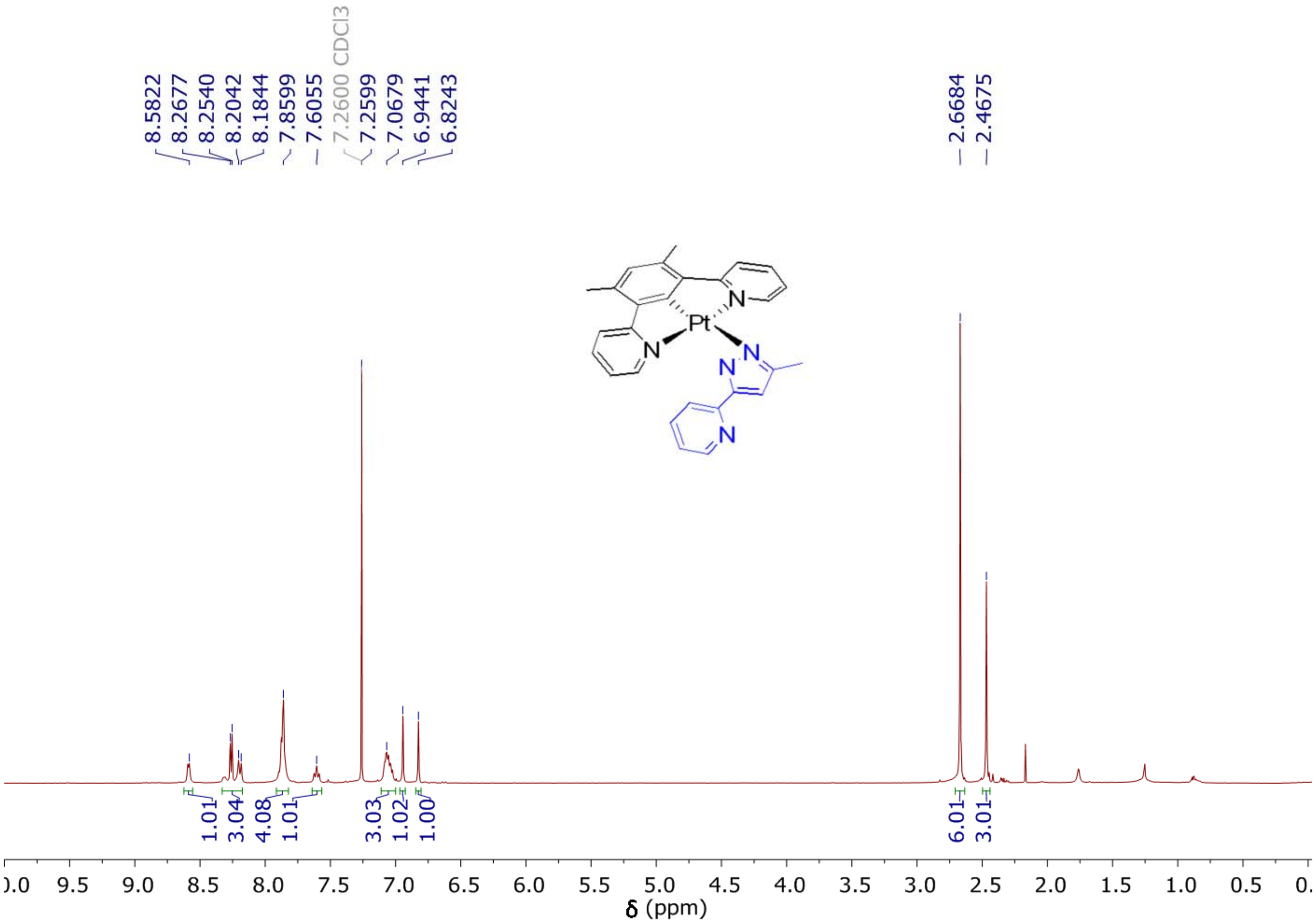

Figure S15. ^{1}H NMR spectrum (400.1 MHz, $CDCl_3$, 298 K) of compound **10.**

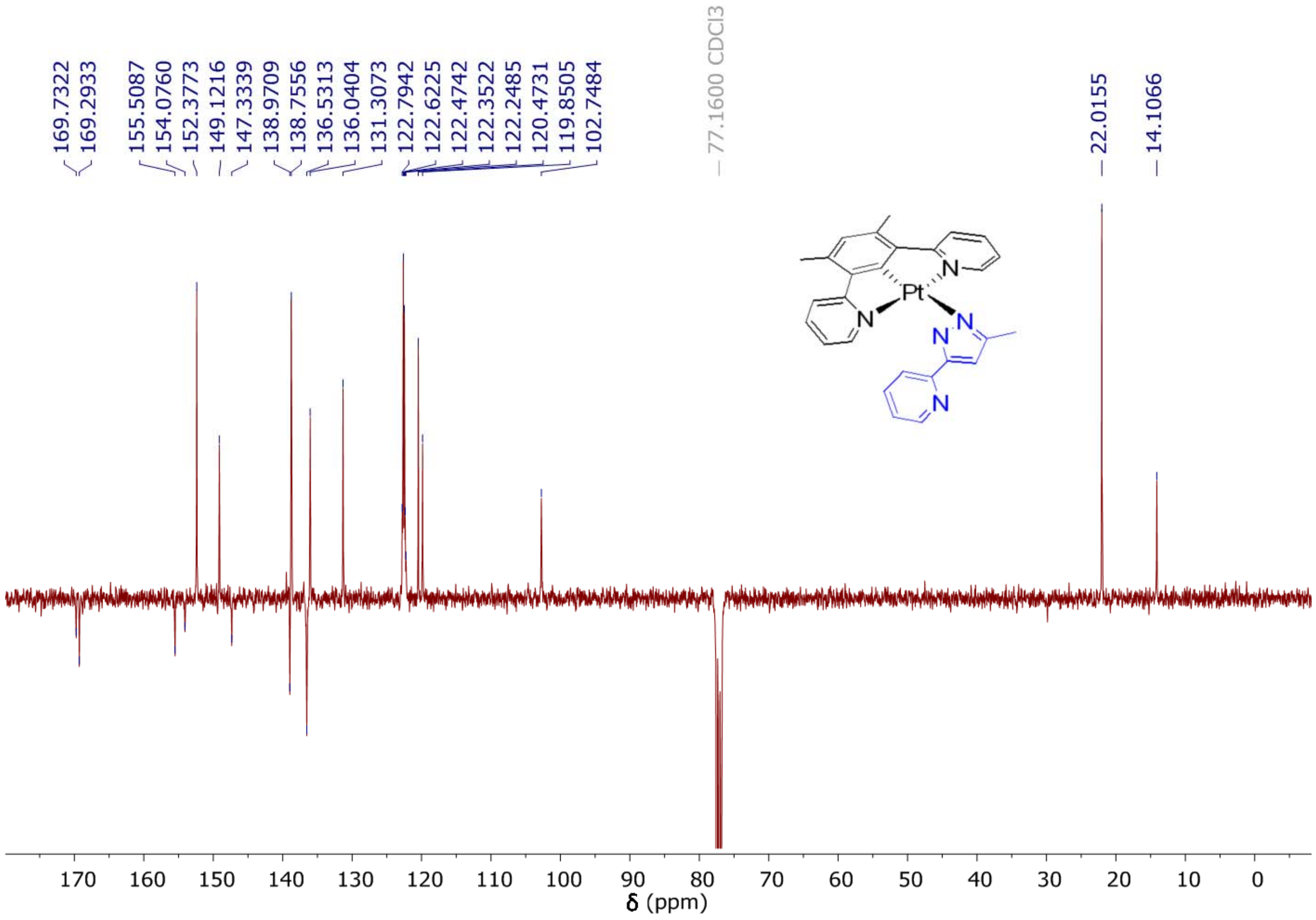

Figure S16. $^{13}C\{^1H\}$-apt NMR spectrum (100.63 MHz, $CDCl_3$, 298 K) of compound **10**.

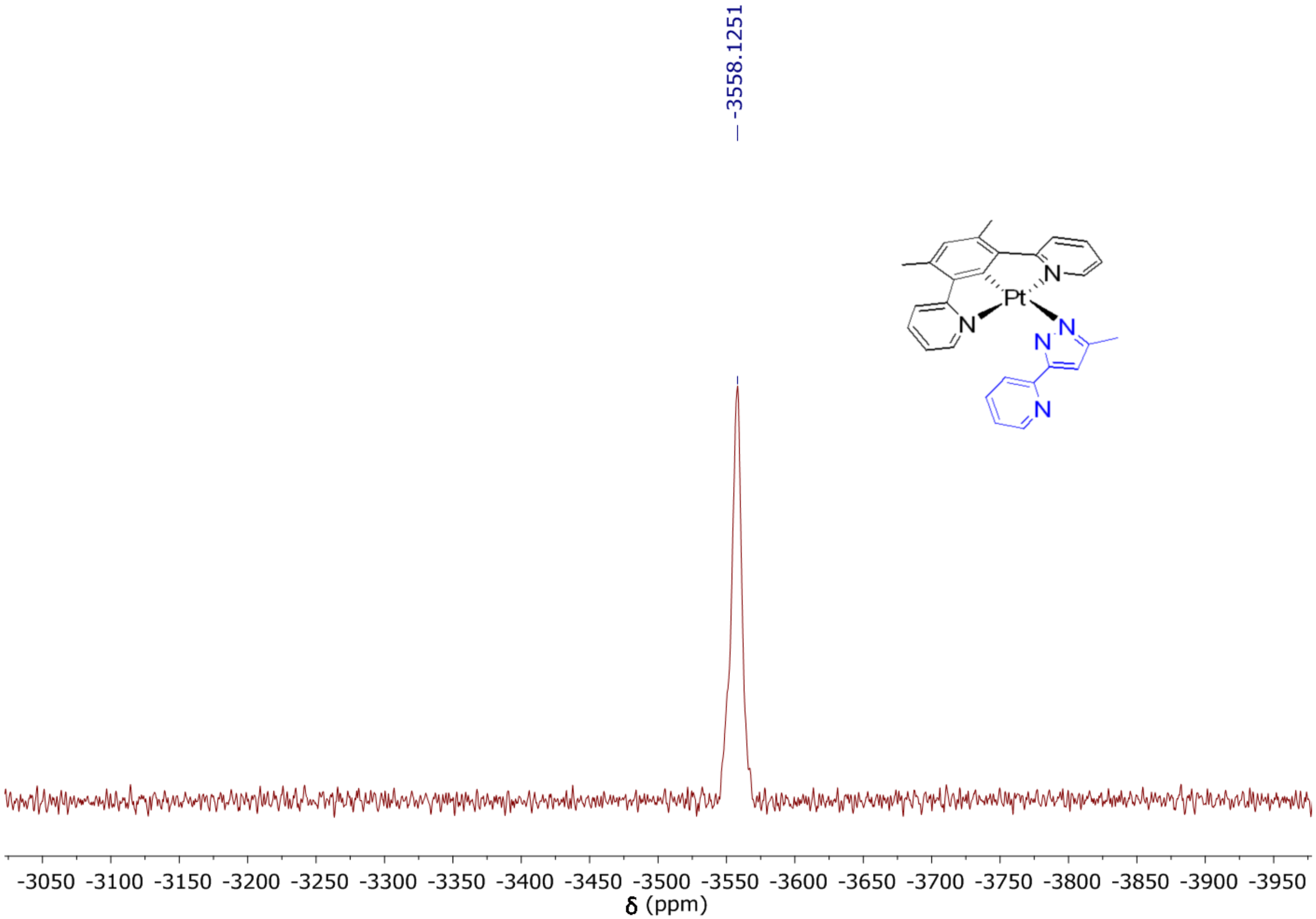

Figure S17. ^{195}Pt{^{1}H} NMR spectrum (85.6 MHz, $CDCl_3$, 298 K) of compound **10**.

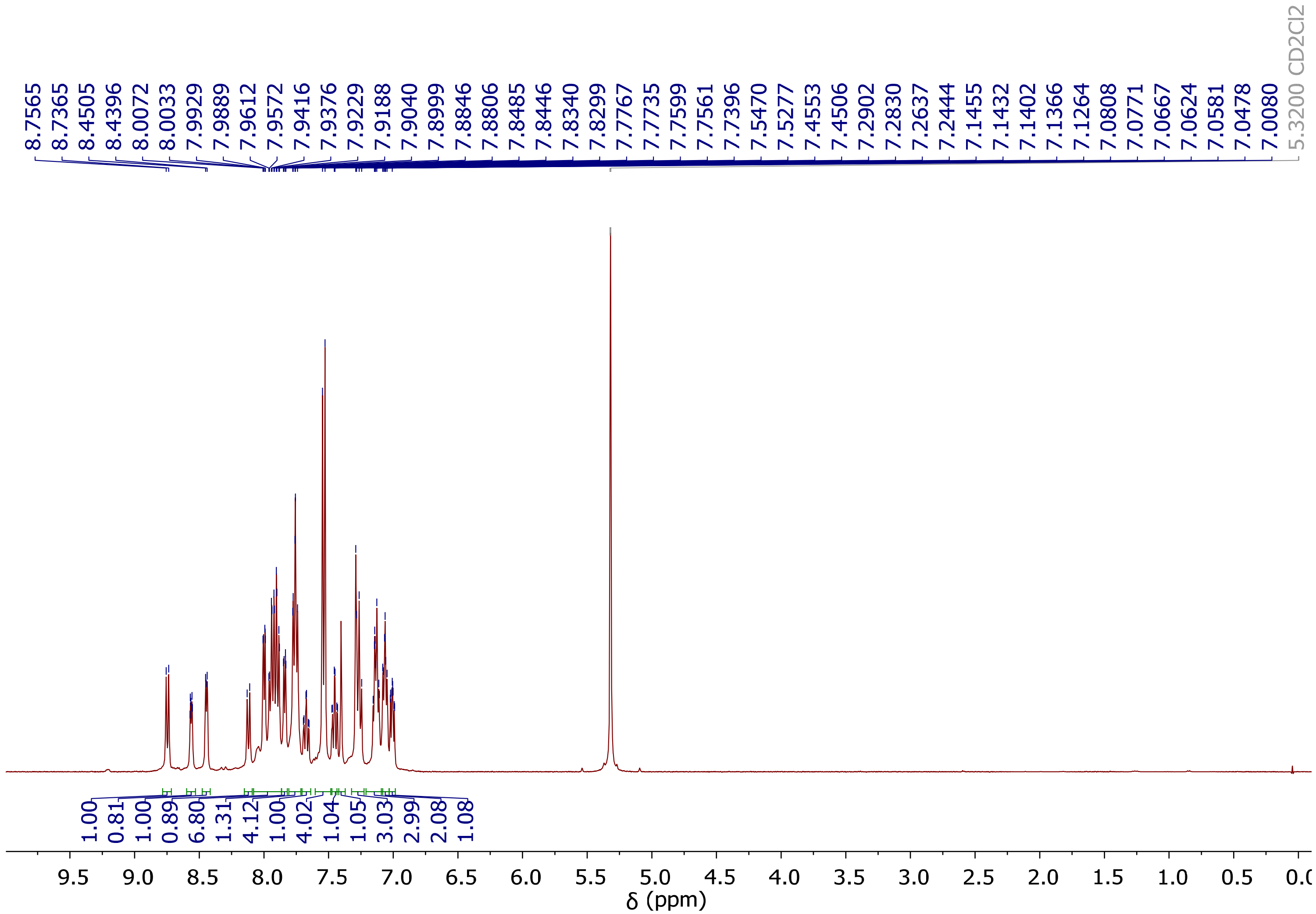

Figure S18. ^{1}H NMR spectrum (400.1 MHz, CD_2Cl_2, 223 K) of the isomeric mixture of complexes **11a** and **11b**.

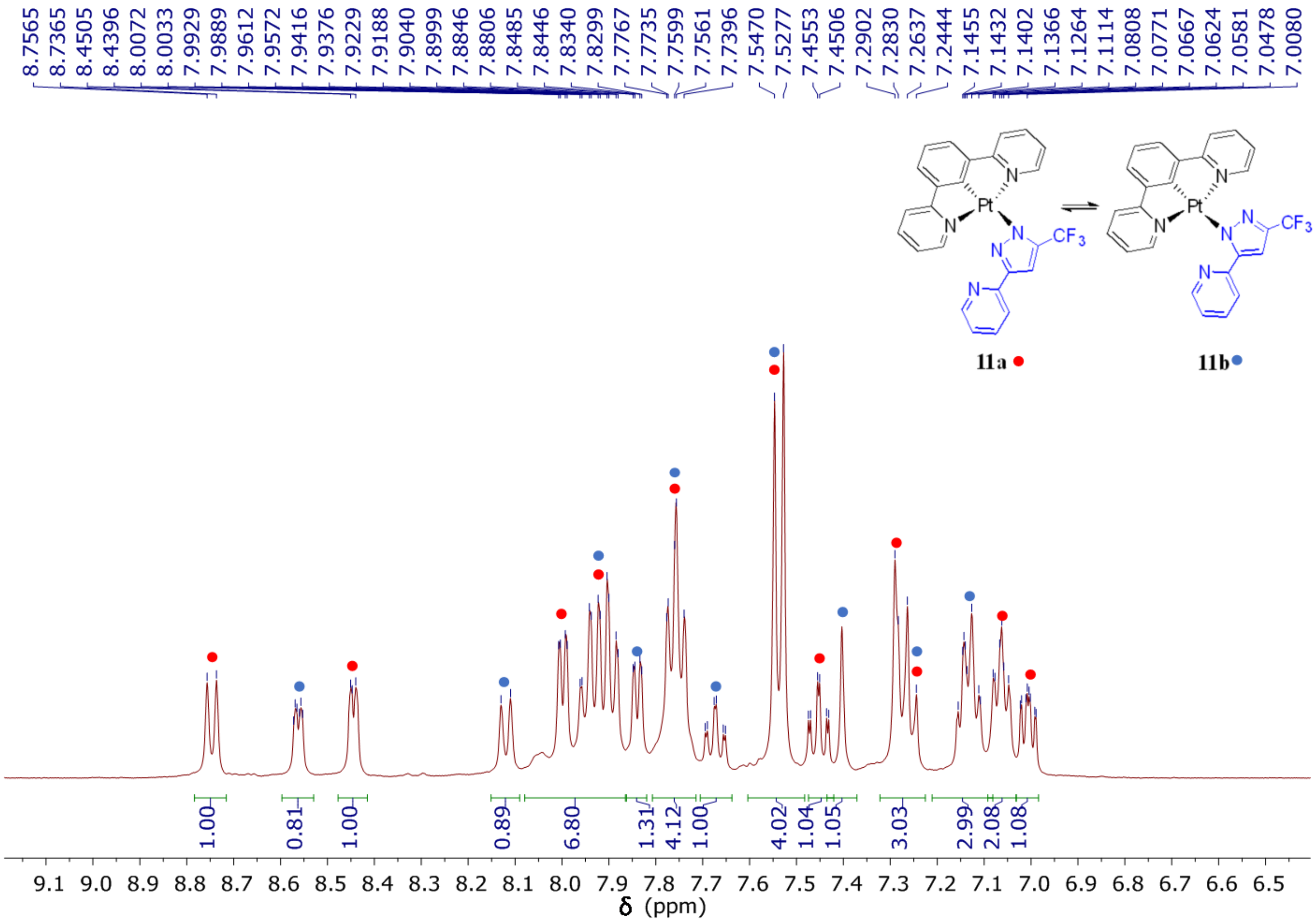

Figure S19. Aromatic region of the ^{1}H NMR spectrum (400.1 MHz, CD_2Cl_2, 223 K) of the isomeric mixture of complexes **11a** and **11b**.

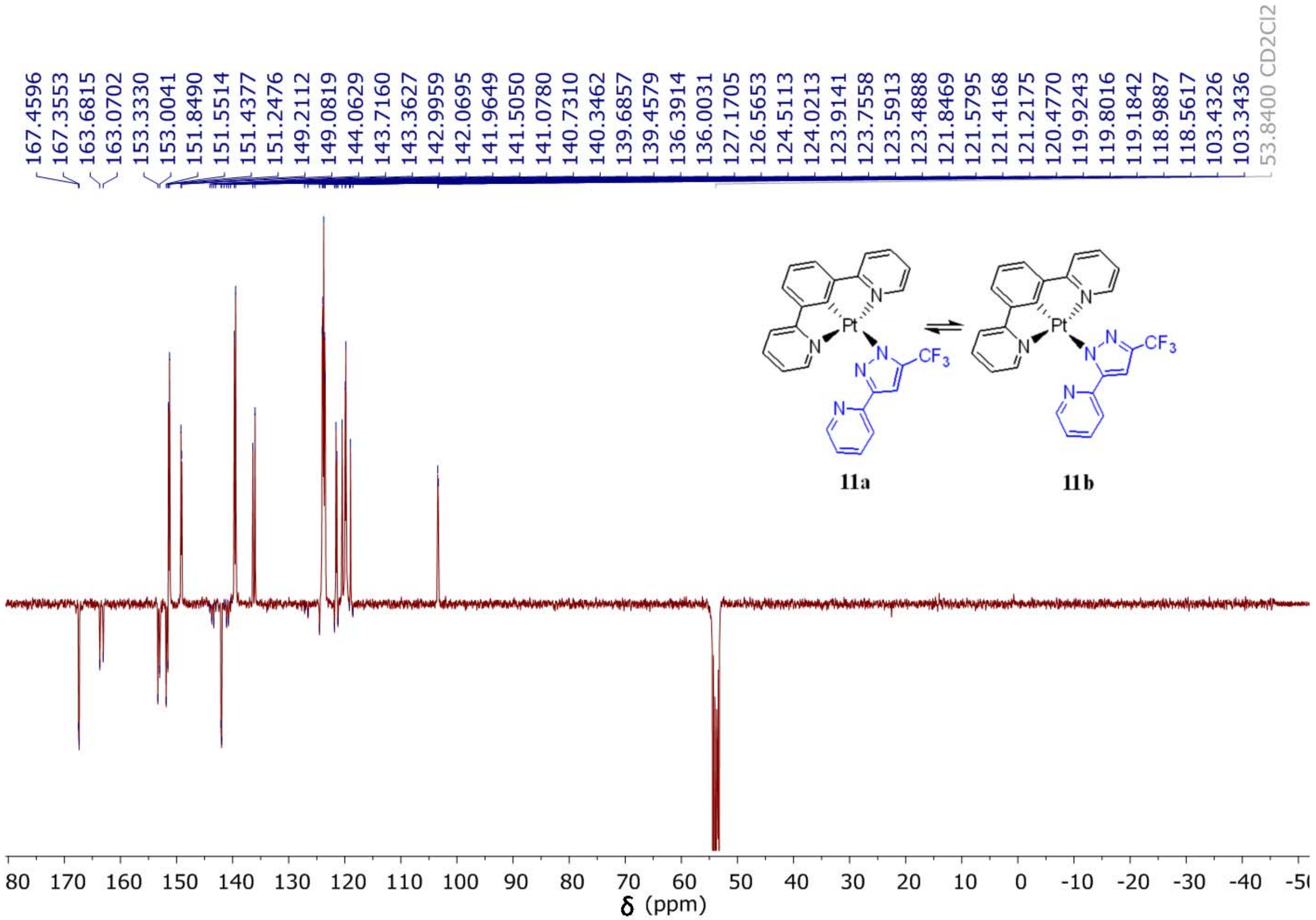

Figure S20. $^{13}C\{^{1}H\}$-apt NMR spectrum (100.63 MHz, CD_2Cl_2, 223 K) of the isomeric mixture of complexes **11a** and **11b**.

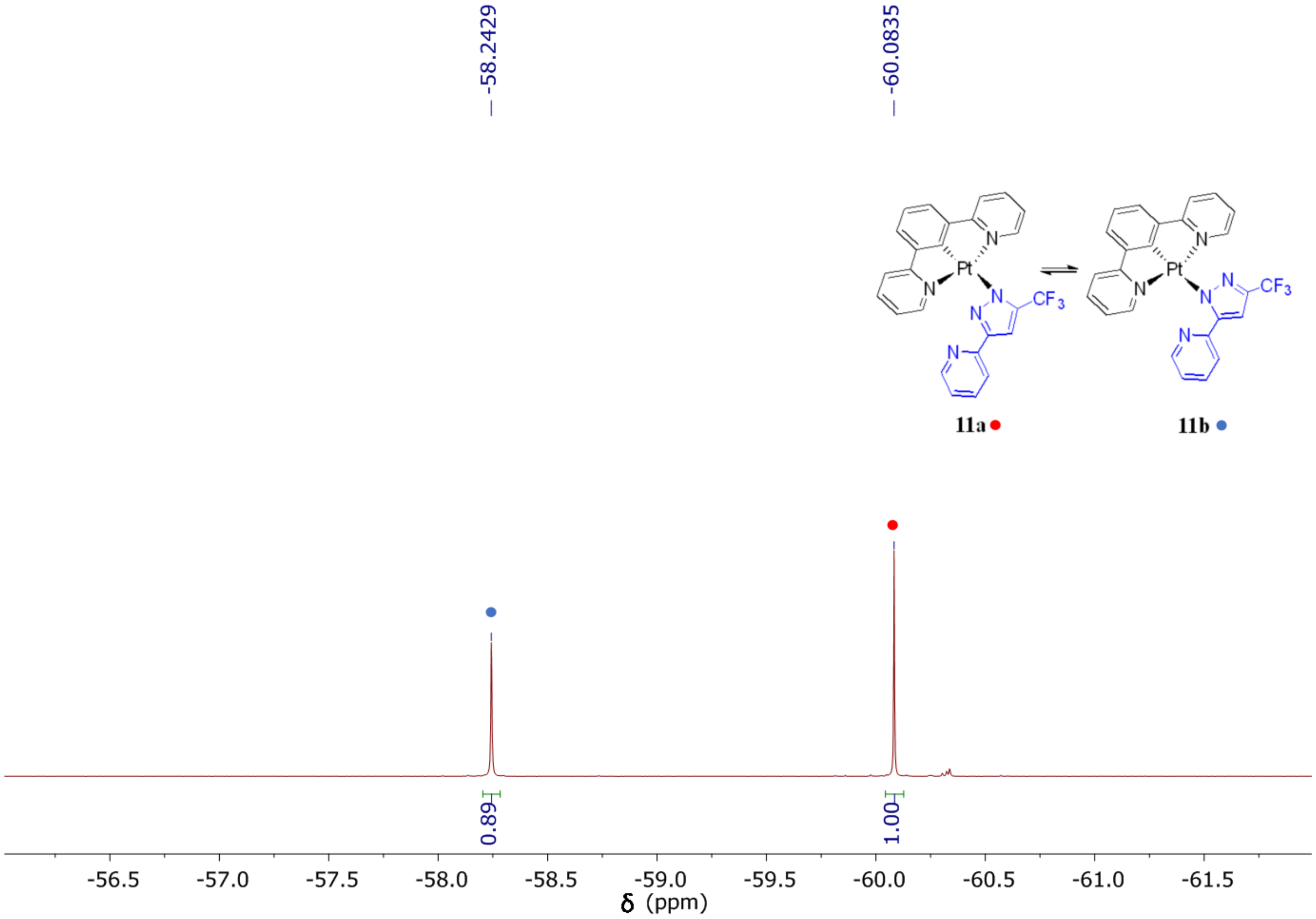

Figure S21. $^{19}F\{^{1}H\}$ NMR spectrum (282.40 MHz, CD_2Cl_2, 223 K) of the isomeric mixture of complexes **11a** and **11b**.

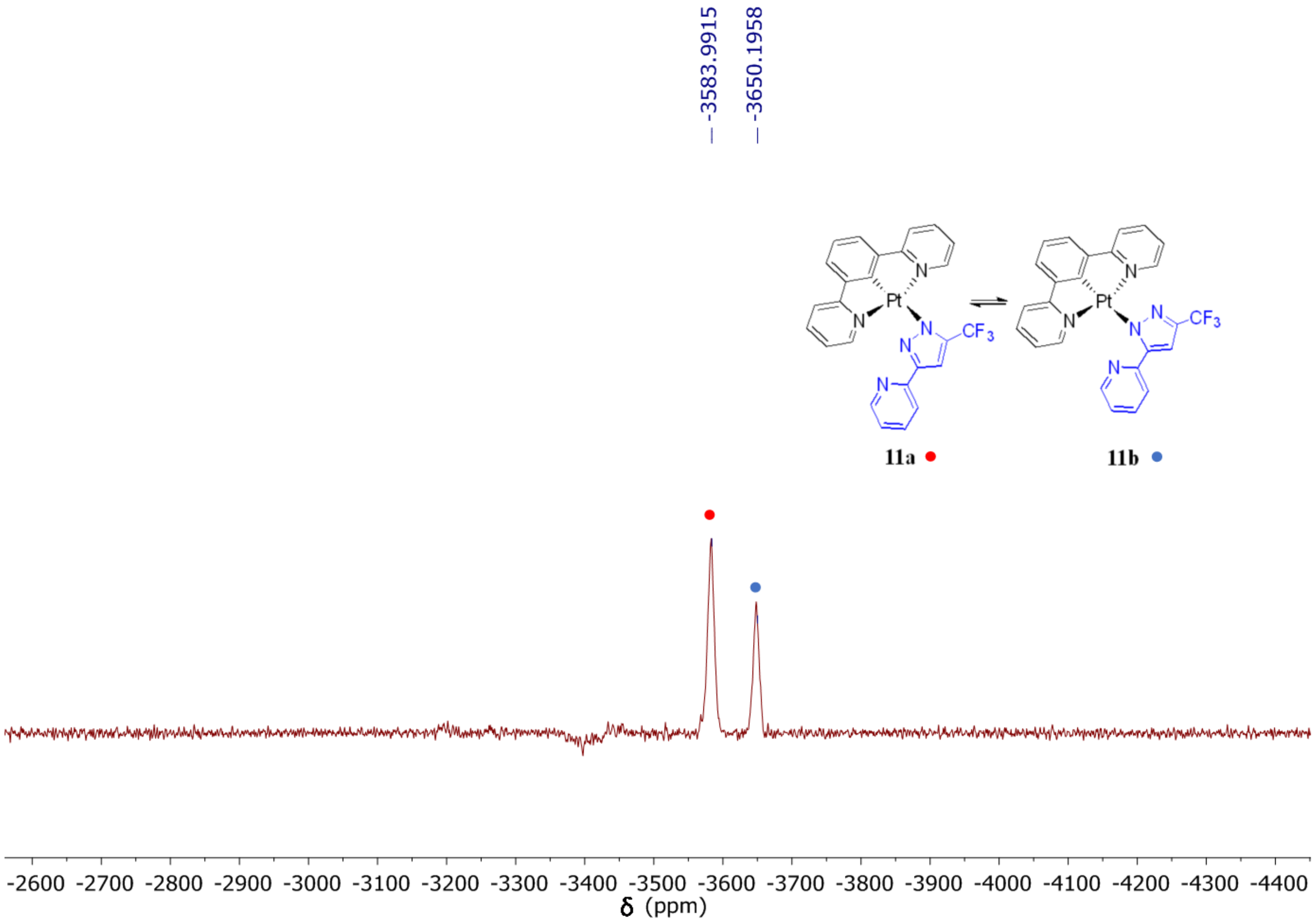

Figure S22. $^{195}Pt\{^{1}H\}$ NMR spectrum (85.6 MHz, CD_2Cl_2, 298 K) of the isomeric mixture of complexes **11a** and **11b**.

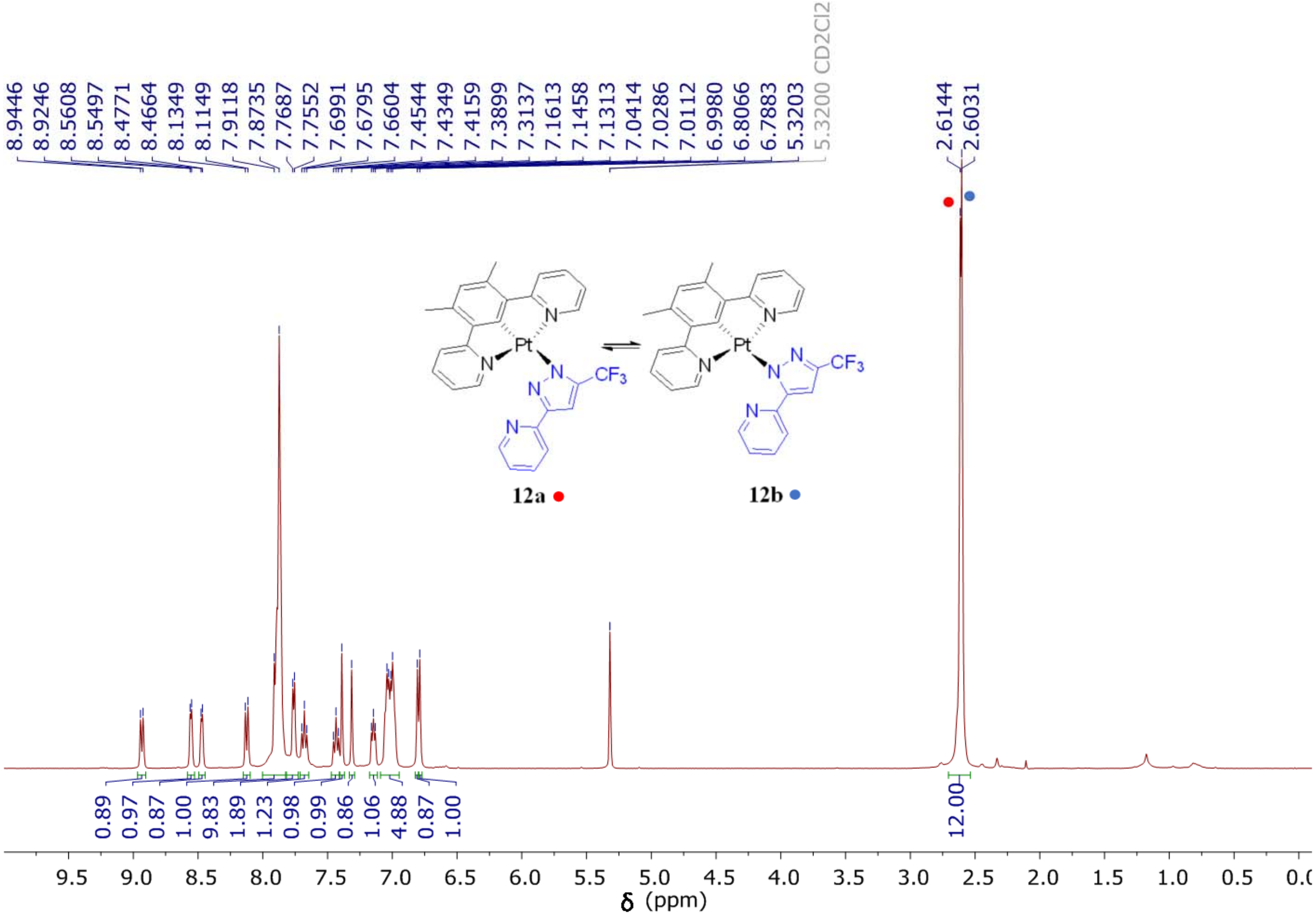

Figure S23. ^{1}H NMR spectrum (400.1 MHz, CD_2Cl_2, 223 K) of the isomeric mixture of complexes **12a** and **12b**.

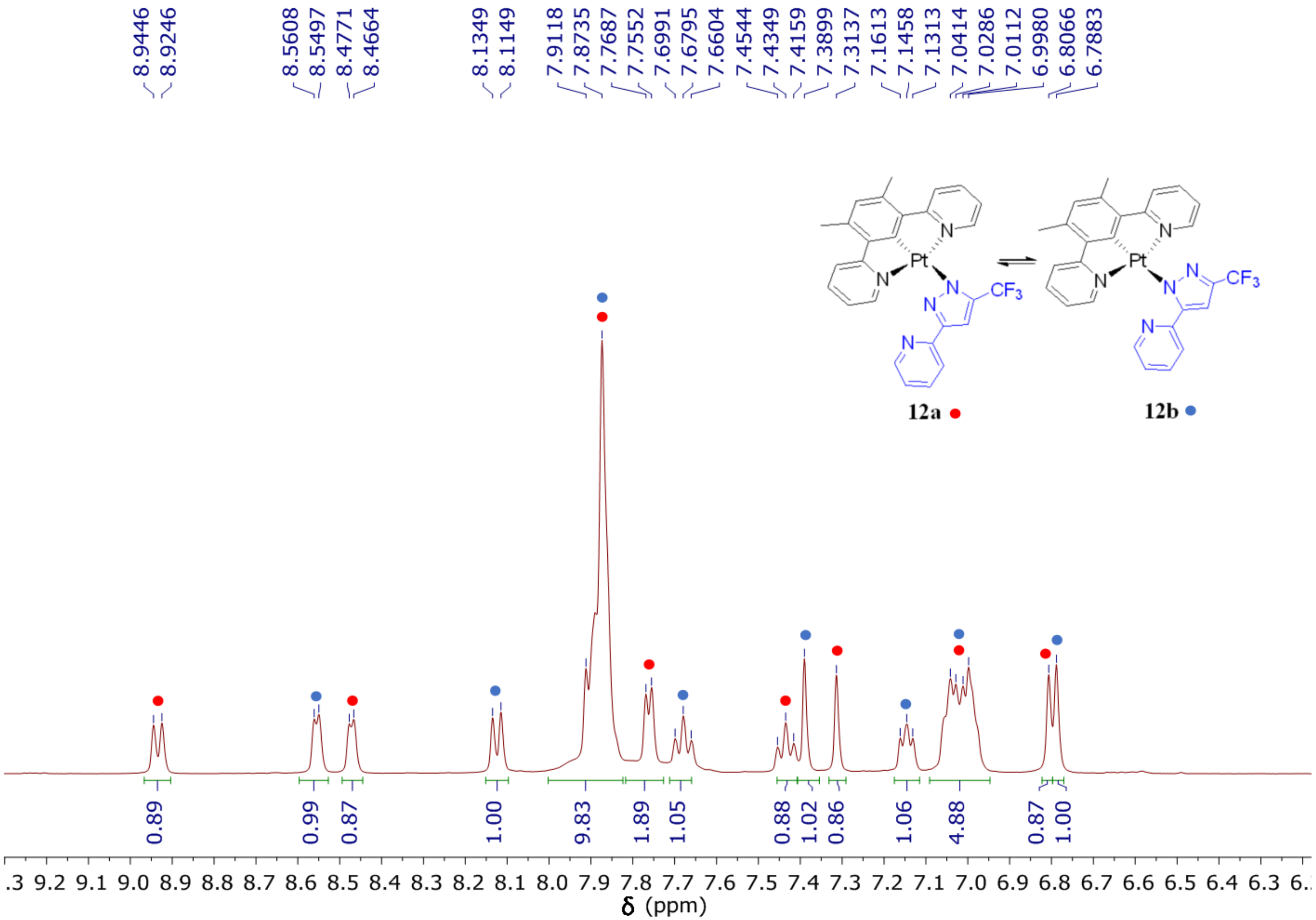

Figure S24. Aromatic region of the ^{1}H NMR spectrum (400.1 MHz, CD_2Cl_2, 223 K) of the isomeric mixture of complexes **12a** and **12b**.

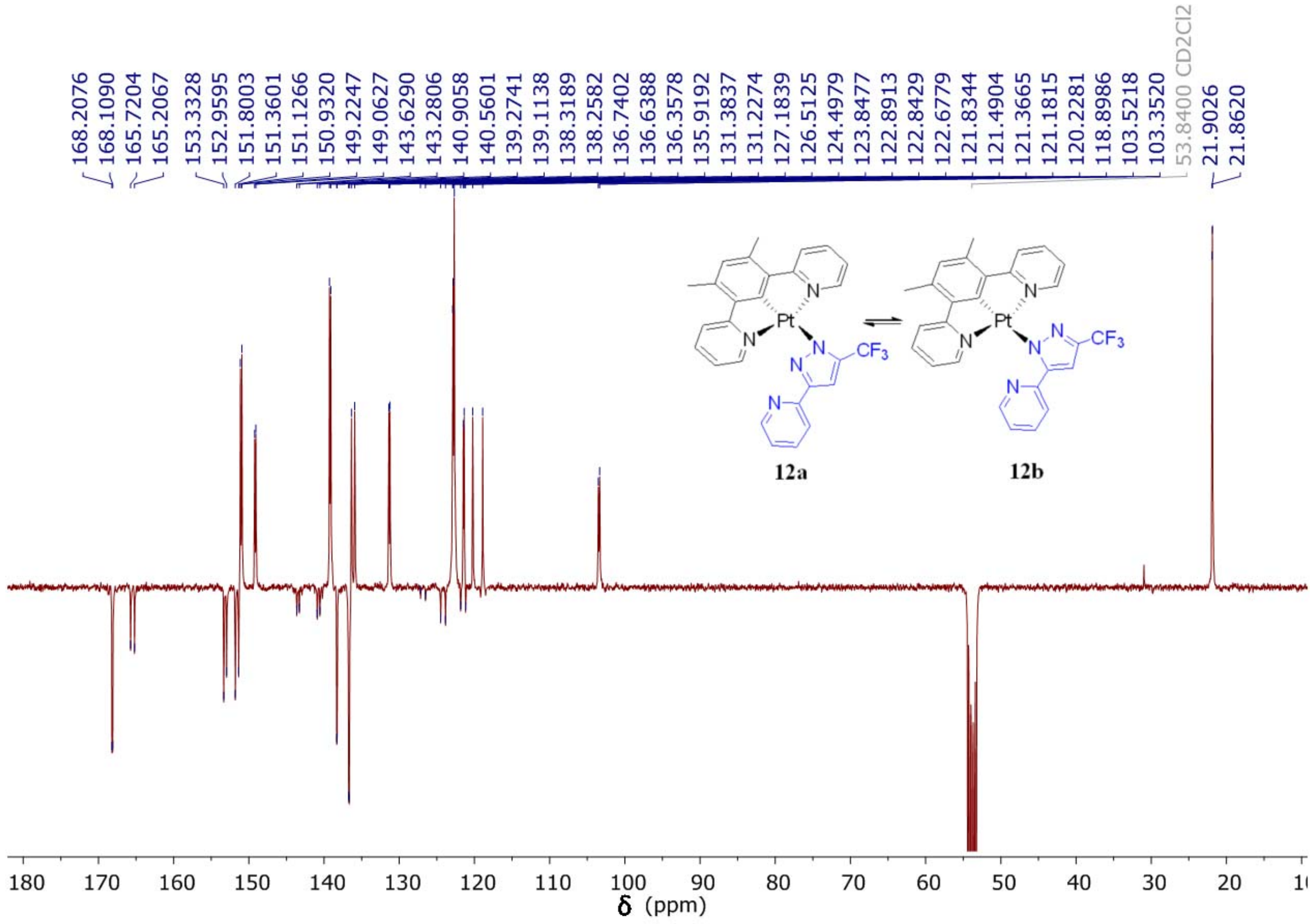

Figure S25. $^{13}C\{^{1}H\}$-apt NMR spectrum (100.63 MHz, CD_2Cl_2, 223 K) of the isomeric mixture of complexes **12a** and **12b**.

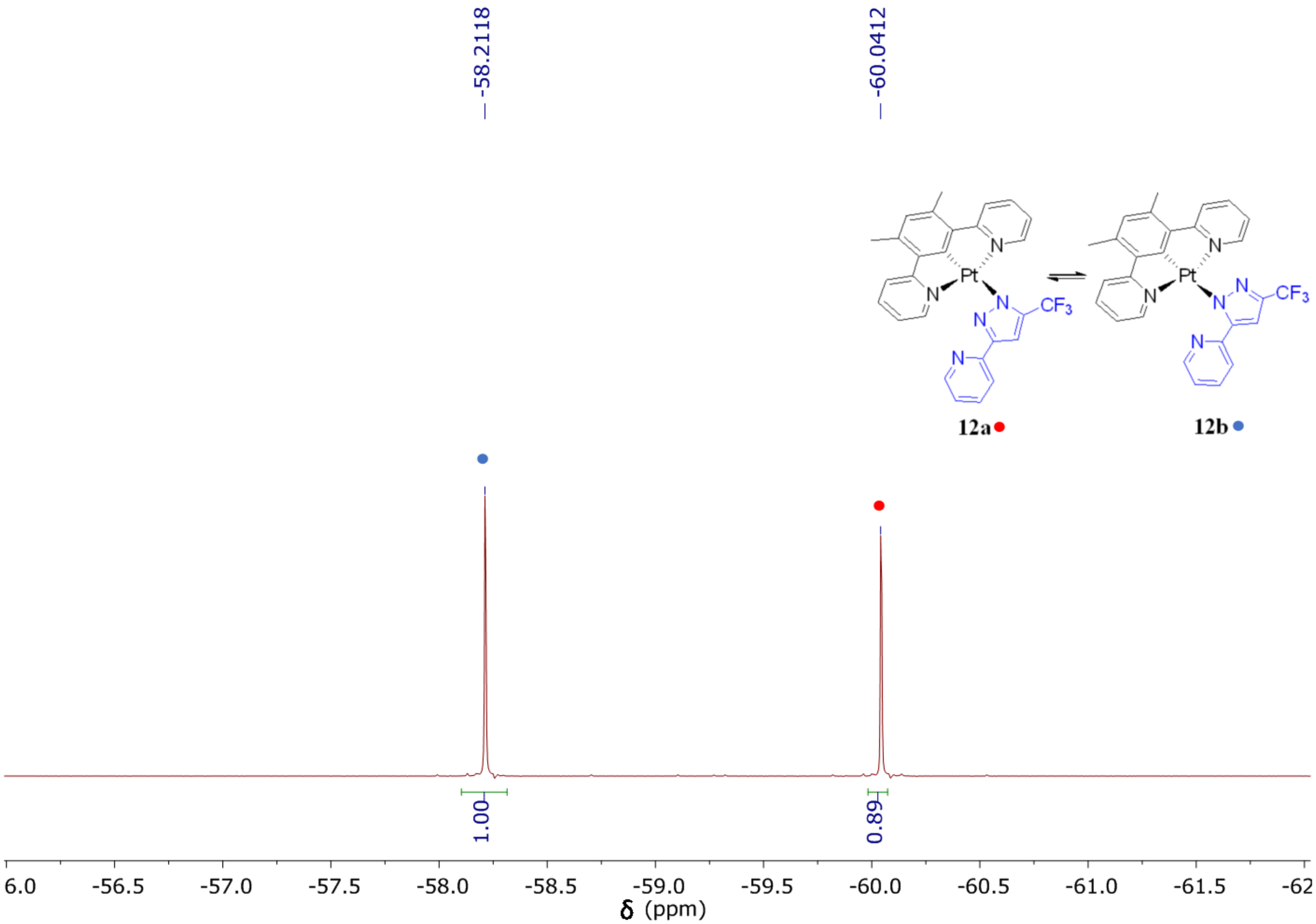

Figure S26. $^{19}F\{^1H\}$ NMR spectrum (282.40 MHz, CD_2Cl_2, 223 K) of the isomeric mixture of complexes **12a** and **12b**.

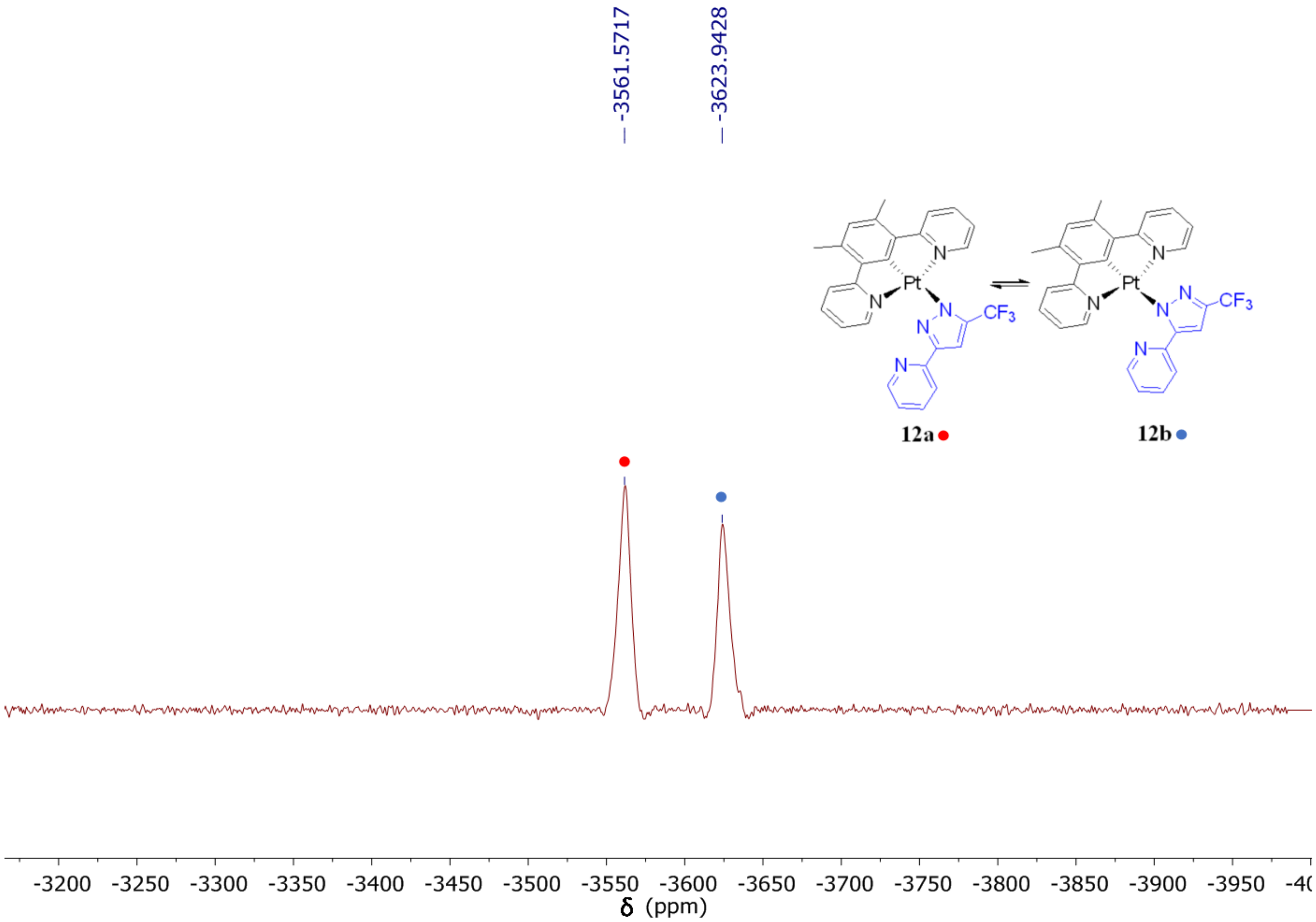

Figure S27. $^{195}Pt\{^1H\}$ NMR spectrum (85.6 MHz, CD_2Cl_2, 298 K) of the isomeric mixture of complexes **12a** and **12b**.

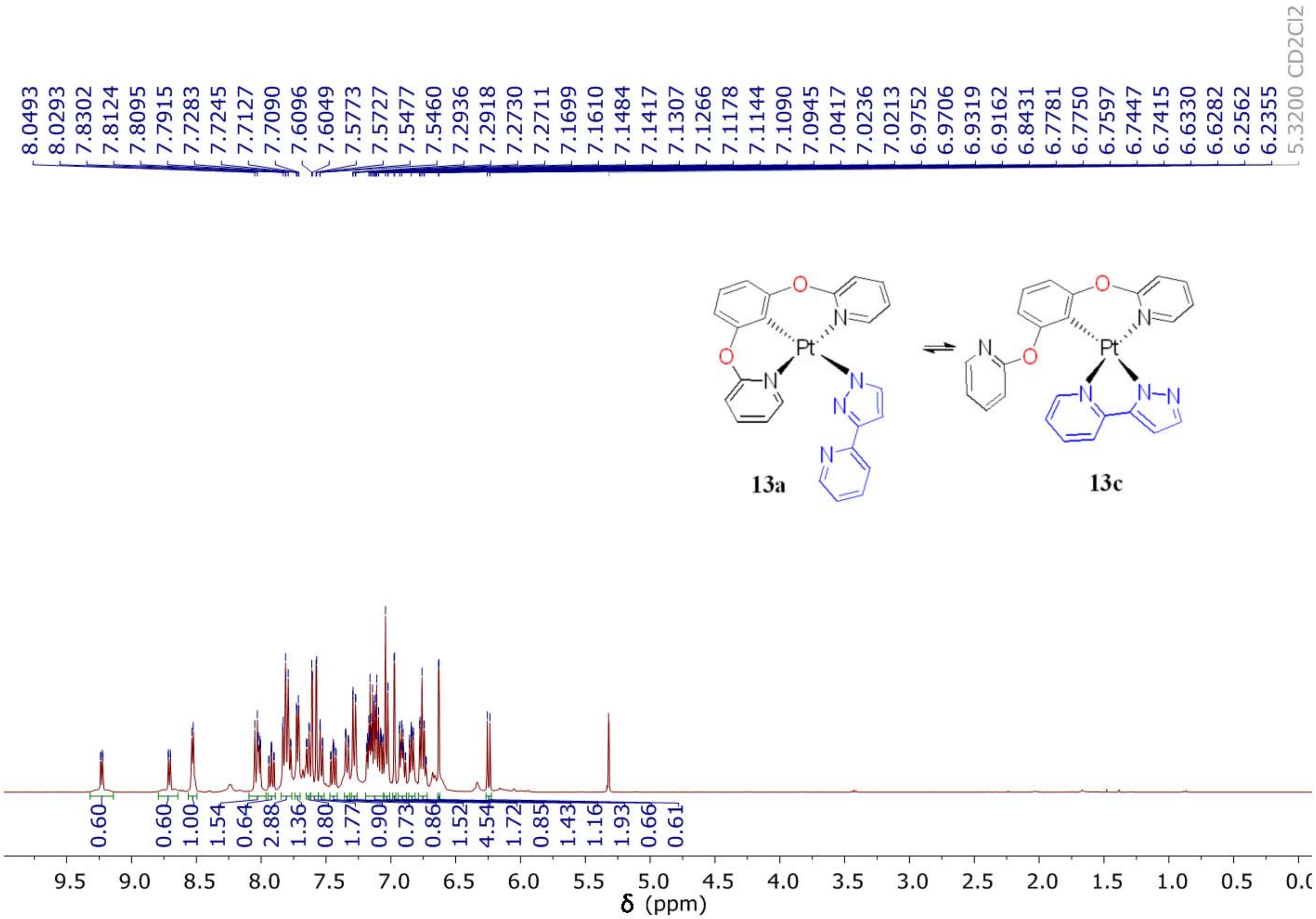

Figure S28. ^{1}H NMR (400.1 MHz, CD_2Cl_2, 253 K) of the isomeric mixture of complexes **13a** and **13c**.

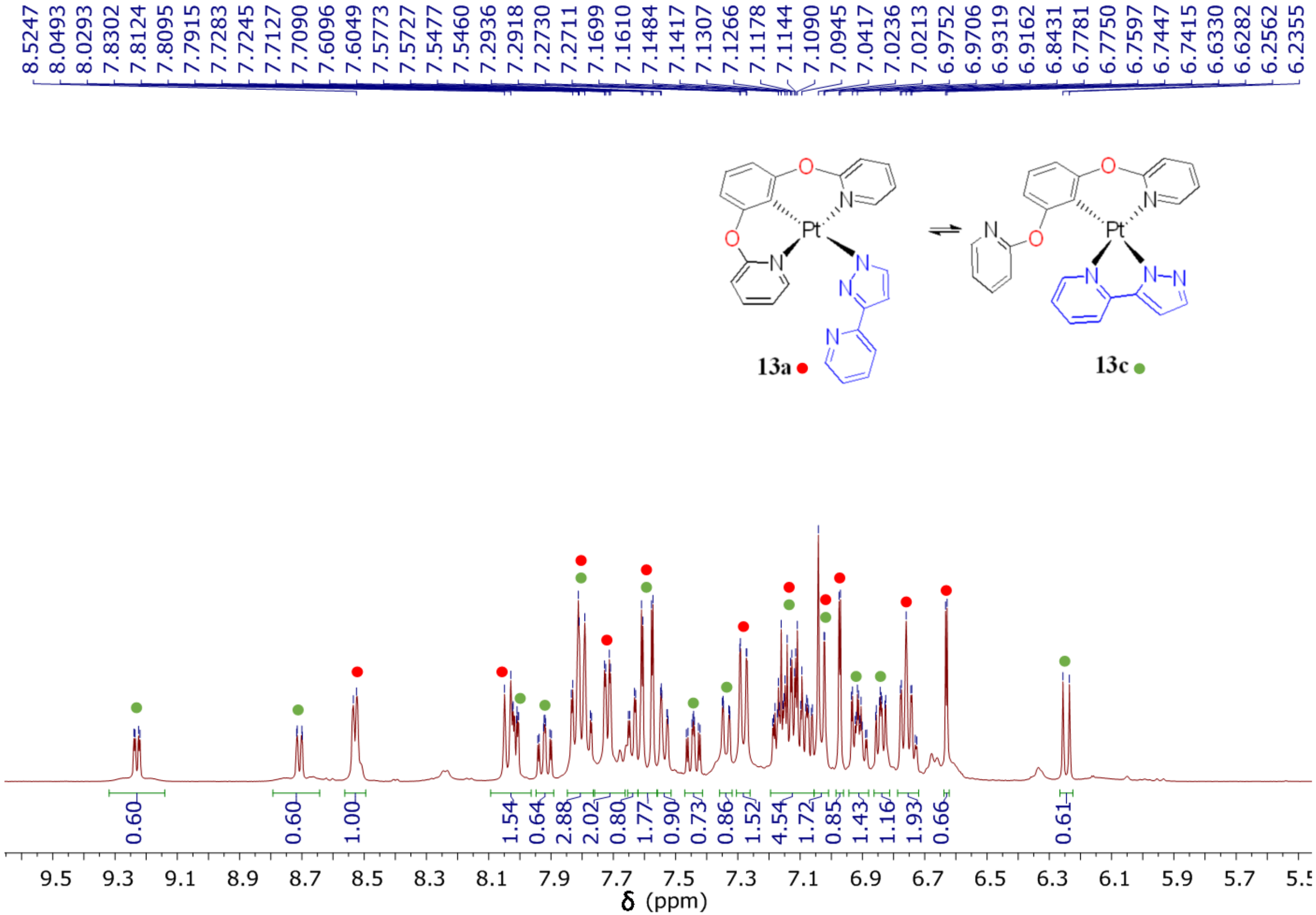

Figure S29. Aromatic region of the ^{1}H NMR spectrum (400.1 MHz, CD_2Cl_2, 253 K) of the isomeric mixture of complexes **13a** and **13c**.

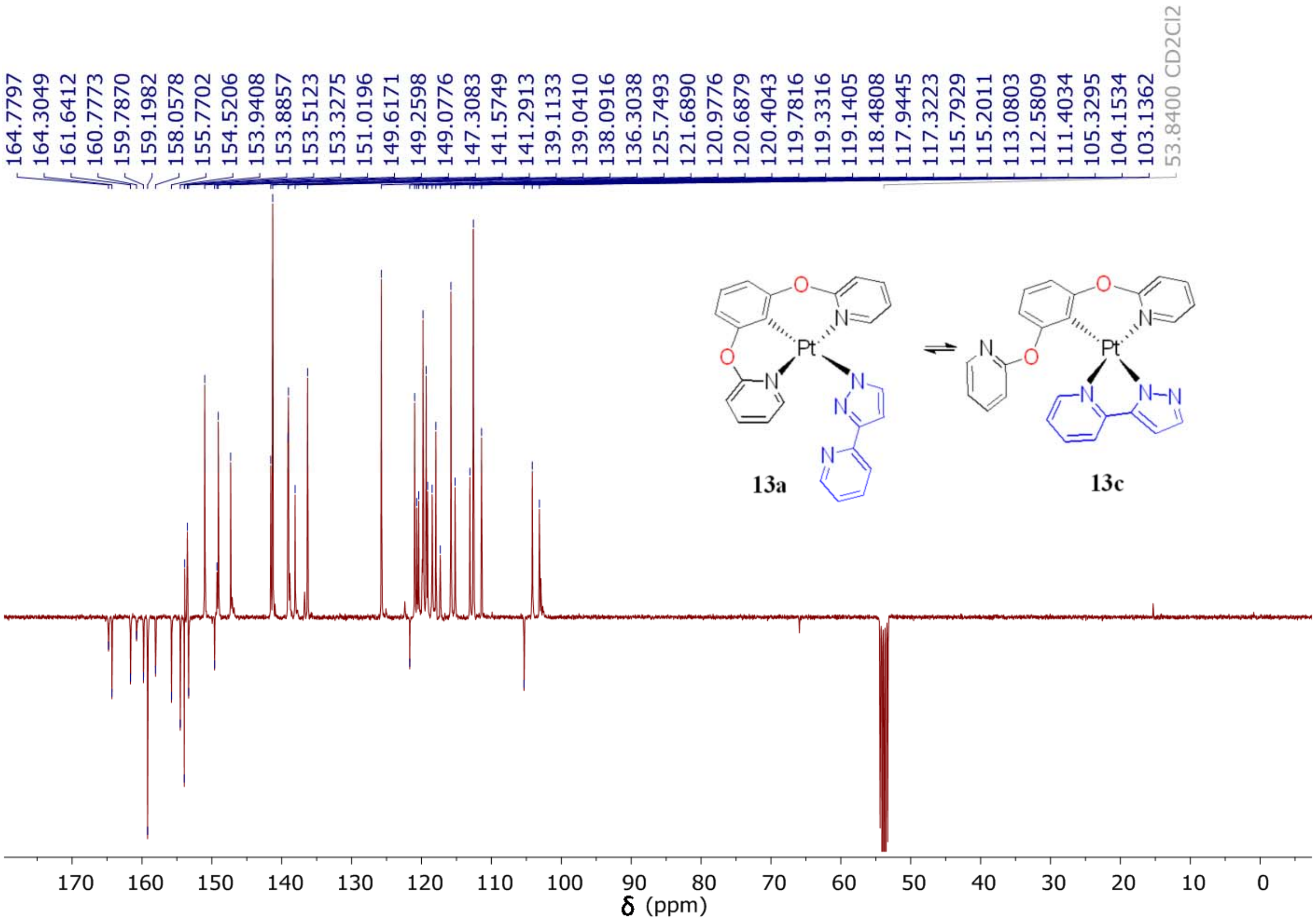

Figure S30. $^{13}C\{^1H\}$-apt NMR spectrum (100.63 MHz, CD_2Cl_2, 253 K) of the isomeric mixture of complexes **13a** and **13b**.

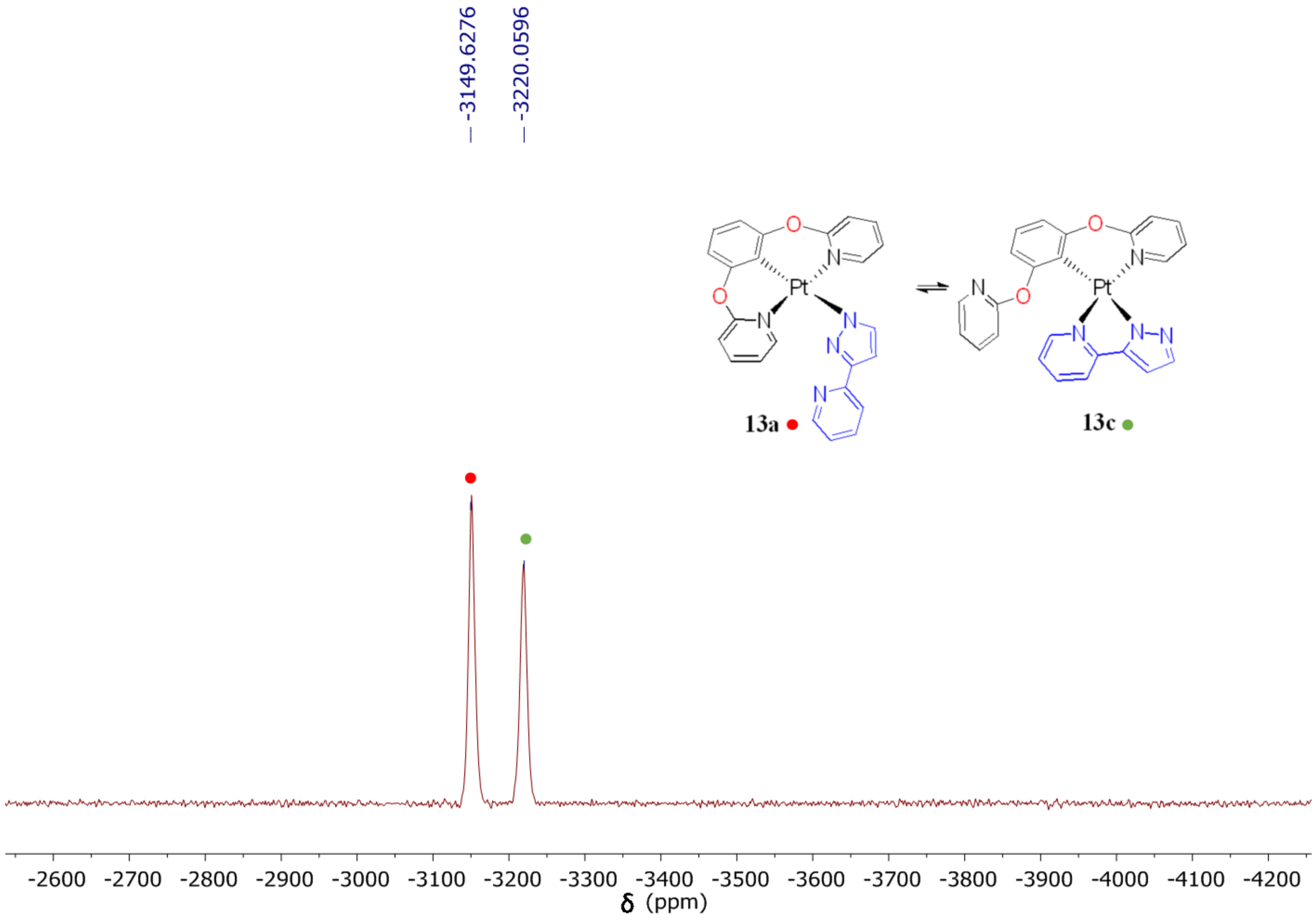

Figure S31. $^{195}Pt\{^1H\}$ NMR spectrum (85.6 MHz, CD_2Cl_2, 298 K) of the isomeric mixture of complexes **13a** and **13b**.

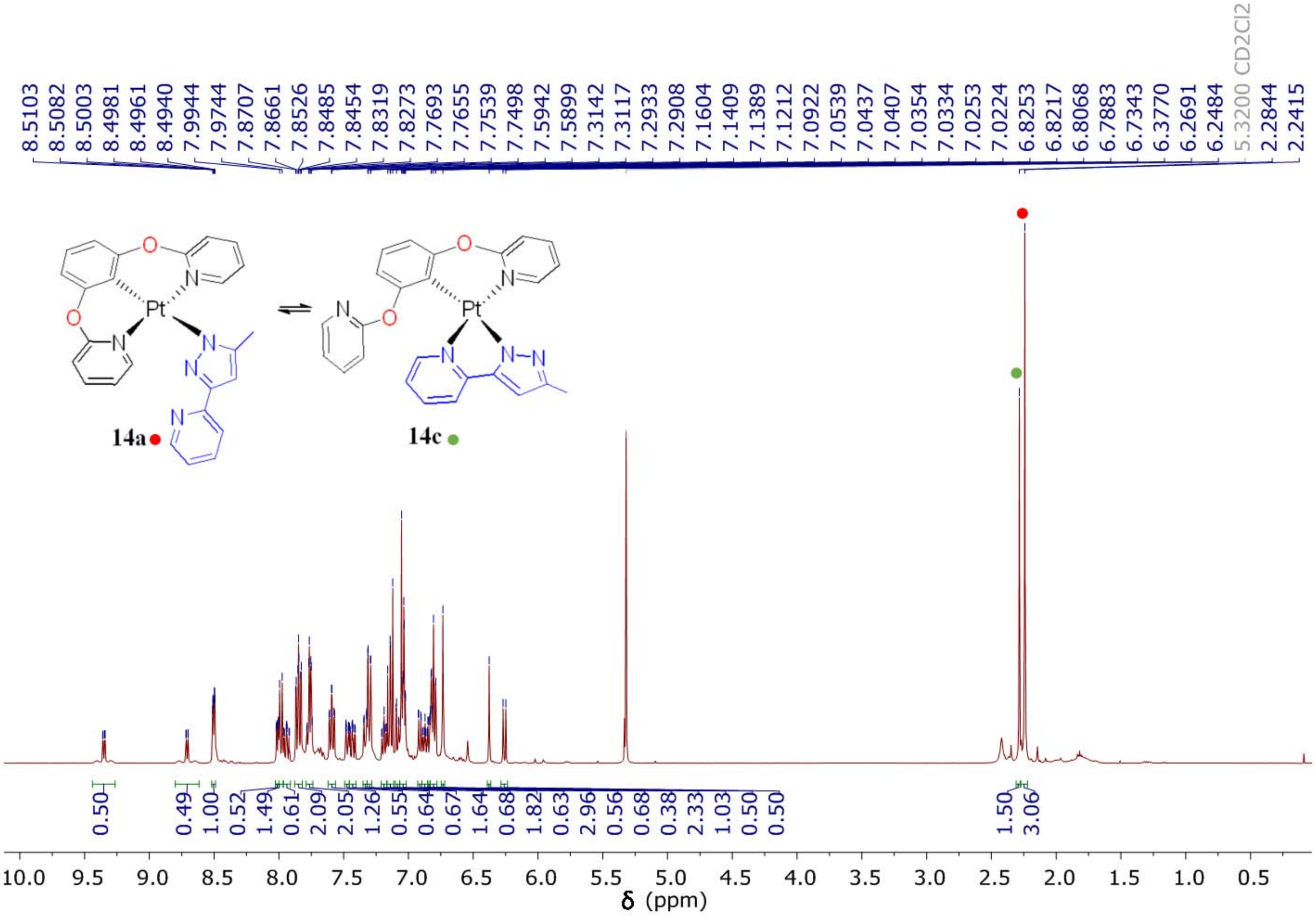

Figure S32. ^{1}H NMR spectrum (400.1 MHz, CD_2Cl_2, 298 K) of the isomeric mixture of complexes **14a** and **14c**.

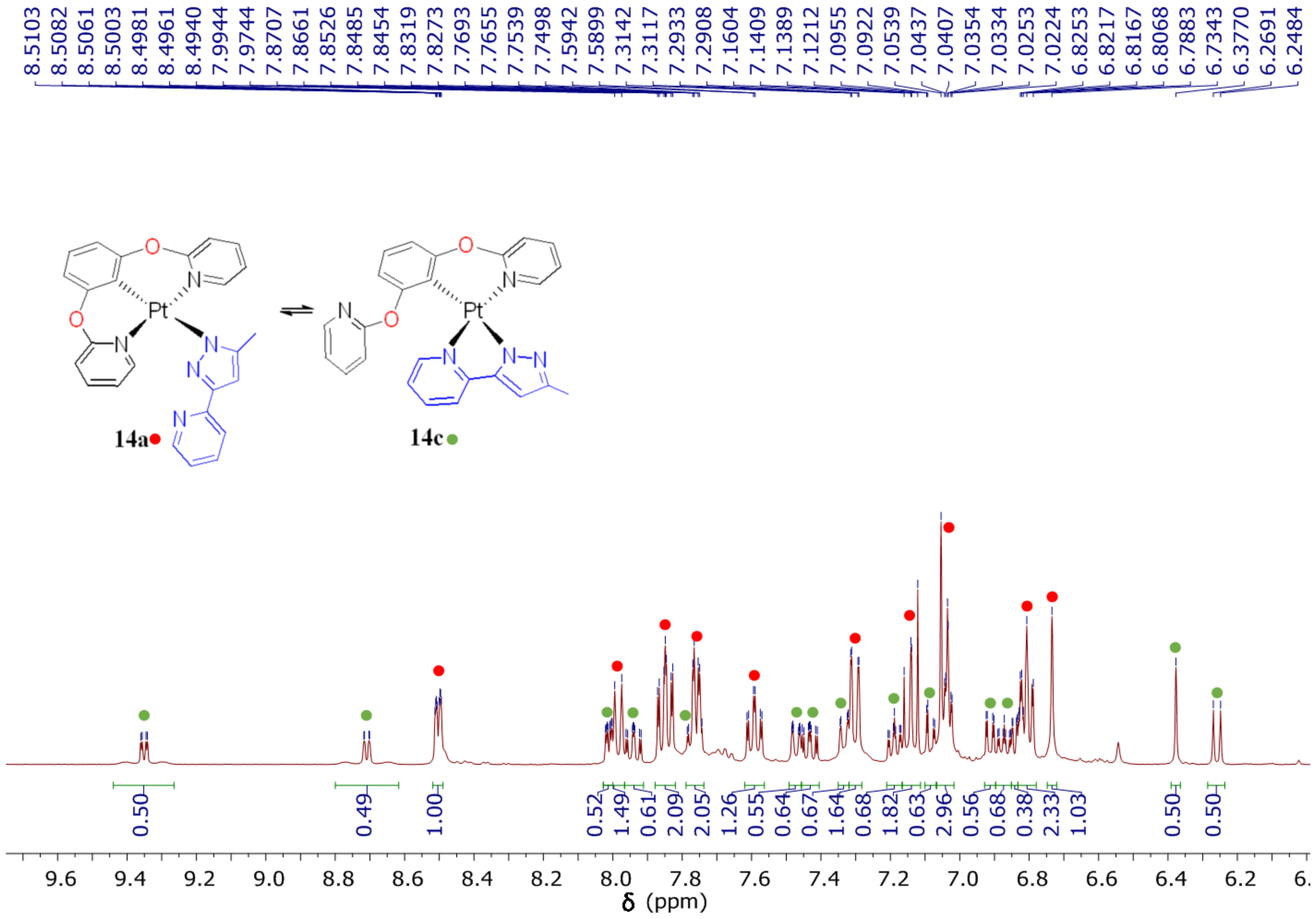

Figure S33. Aromatic region of the ^{1}H NMR spectrum (400.1 MHz, CD_2Cl_2, 298 K) of the isomeric mixture of complexes **14a** and **14c**.

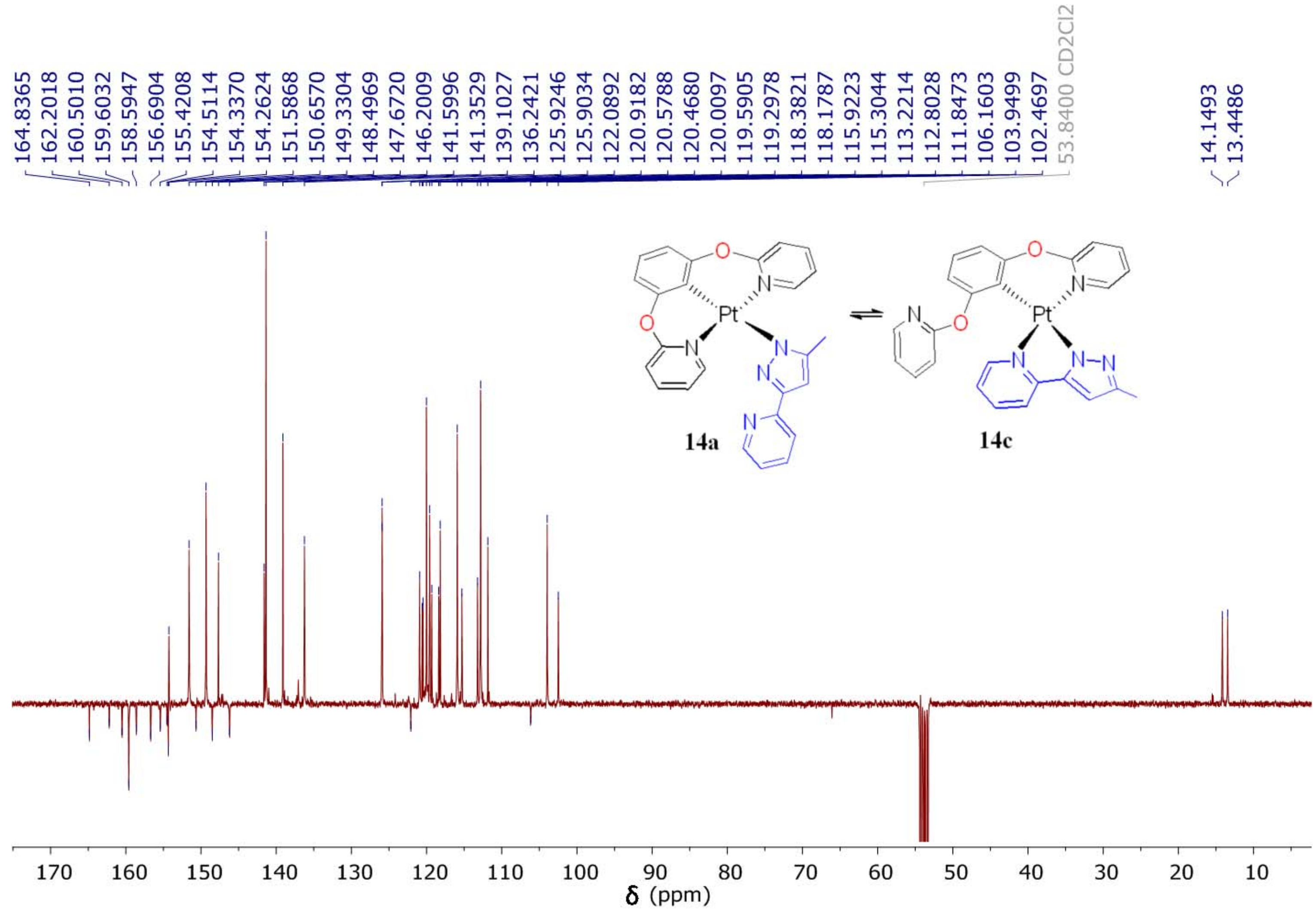

Figure S34. $^{13}C\{^1H\}$-apt NMR spectrum (100.63 MHz, CD_2Cl_2, 298 K) of the isomeric mixture of complexes **14a** and **14c**.

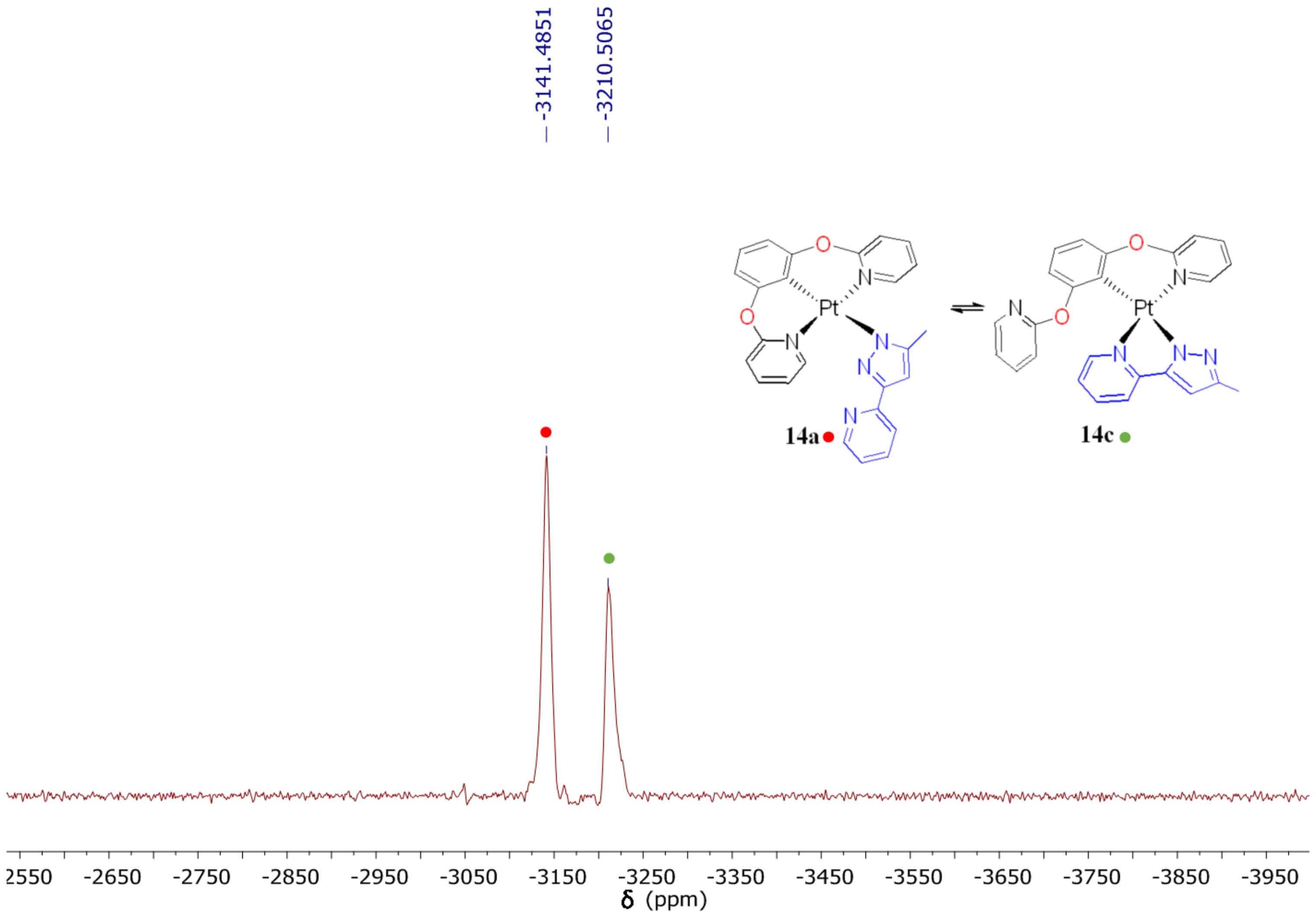

Figure S35. $^{195}Pt\{^1H\}$ NMR spectrum (85.6 MHz, CD_2Cl_2, 298 K) of the isomeric mixture of complexes **14a** and **14c**.

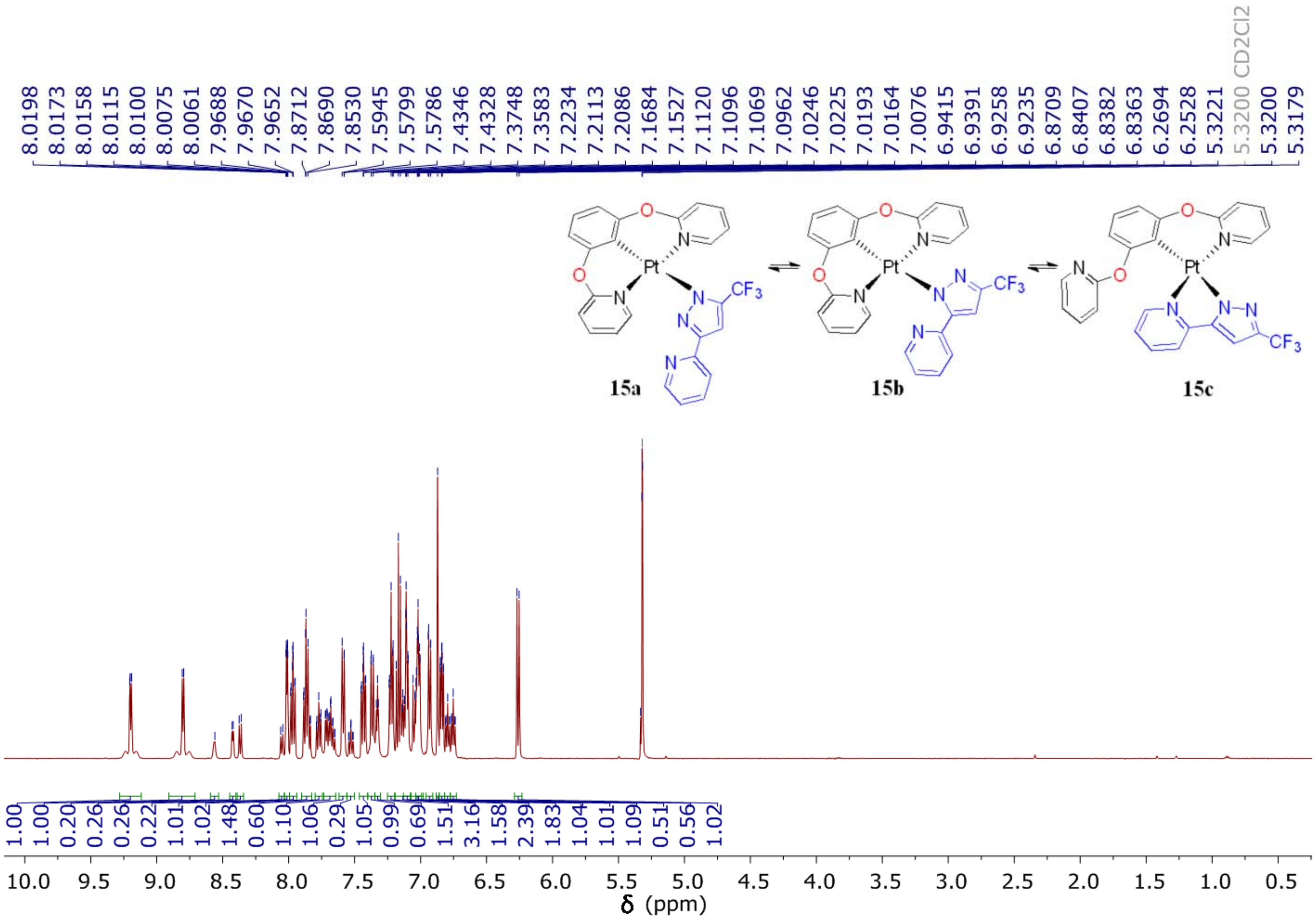

Figure S36. ^{1}H NMR spectrum (500 MHz, CD_2Cl_2, 298 K) of the isomeric mixture of complexes **15a**, **15b** and **15c**.

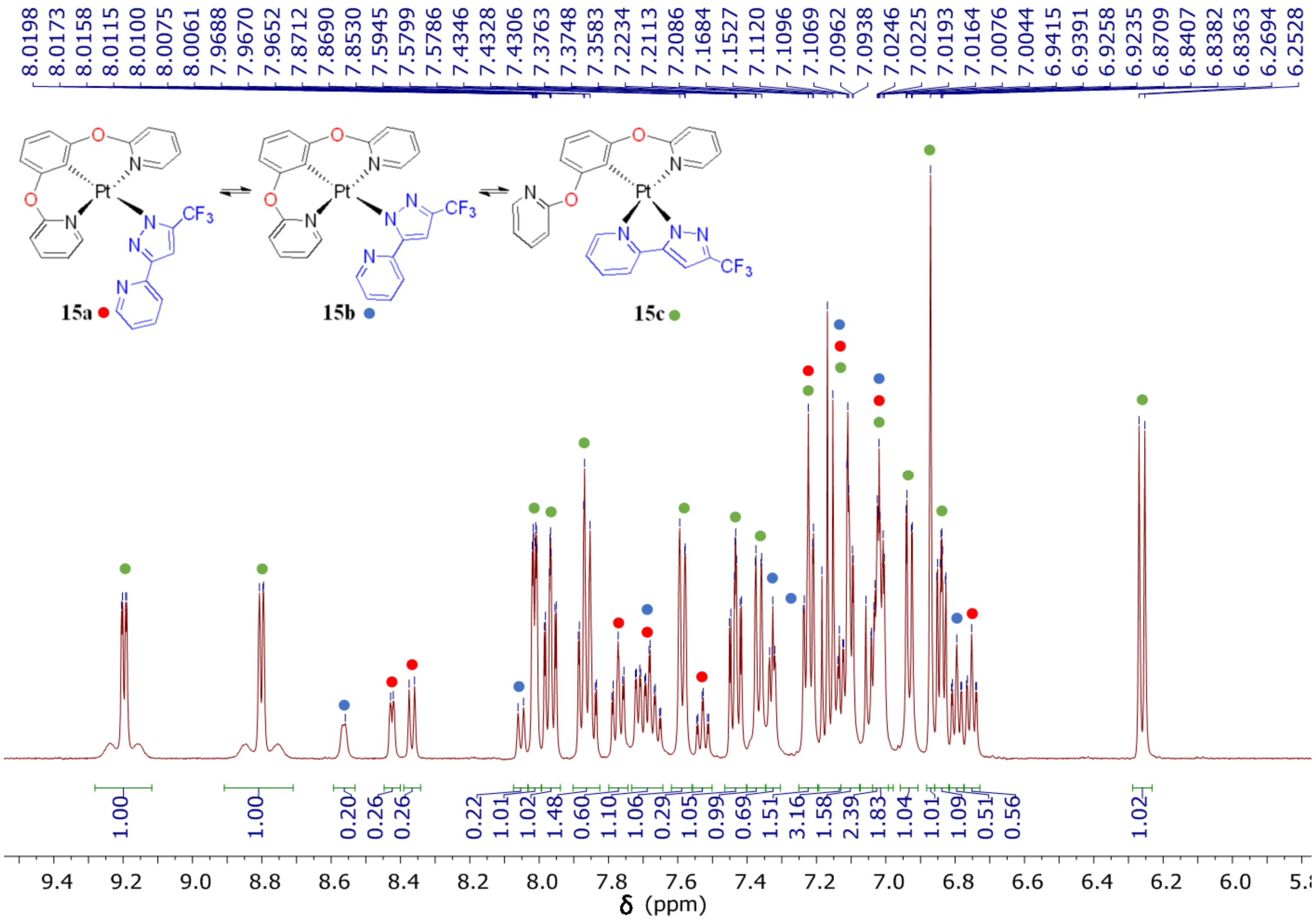

Figure S37. Aromatic region of the ^1H NMR spectrum (500 MHz, CD_2Cl_2, 298 K) of the isomeric mixture of complexes **15a**, **15b** and **15c**.

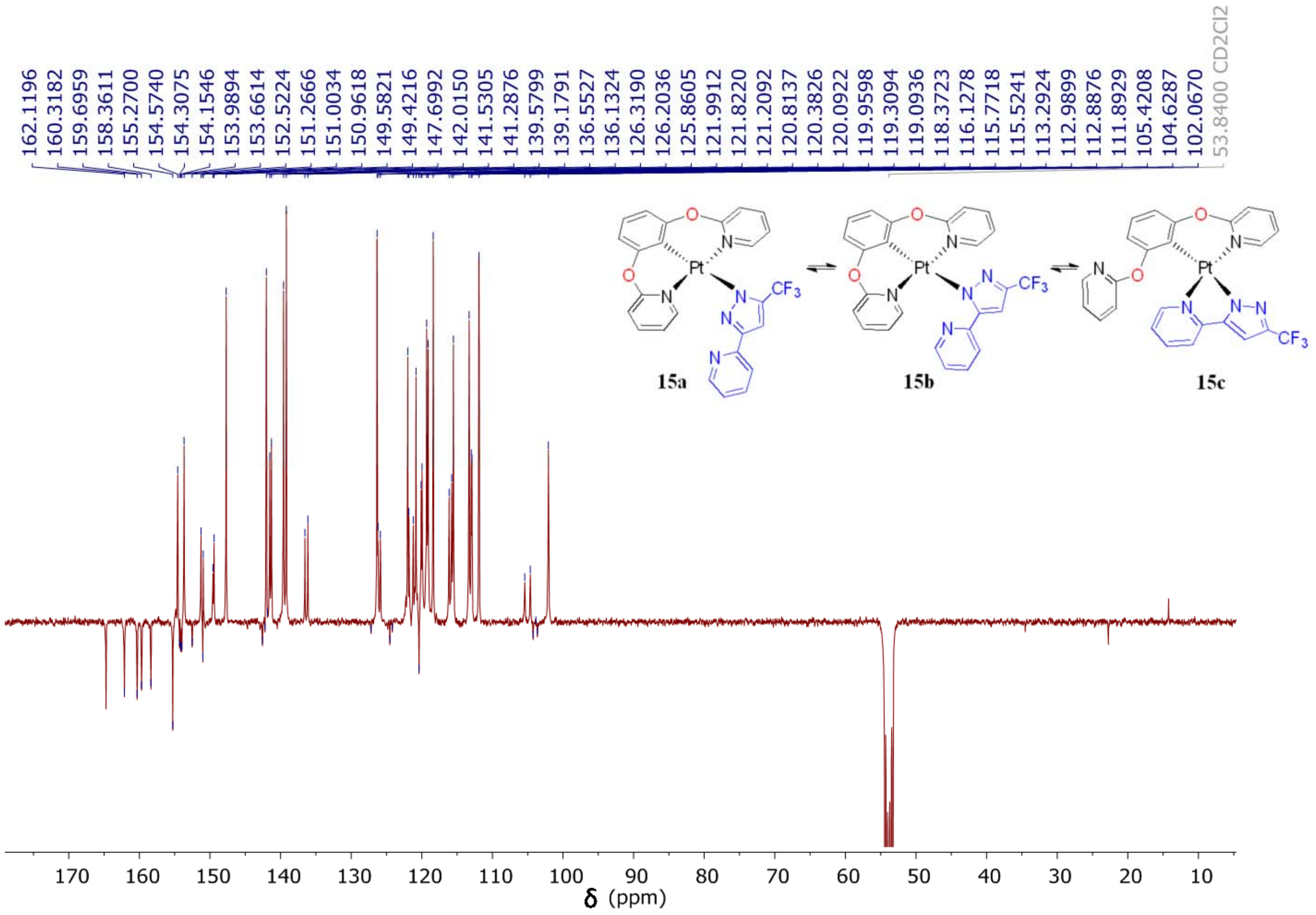

Figure S38. $^{13}C\{^{1}H\}$-apt NMR spectrum (100.63 MHz, CD_2Cl_2, 298 K) of the isomeric mixture of complexes **15a**, **15b** and **15c**.

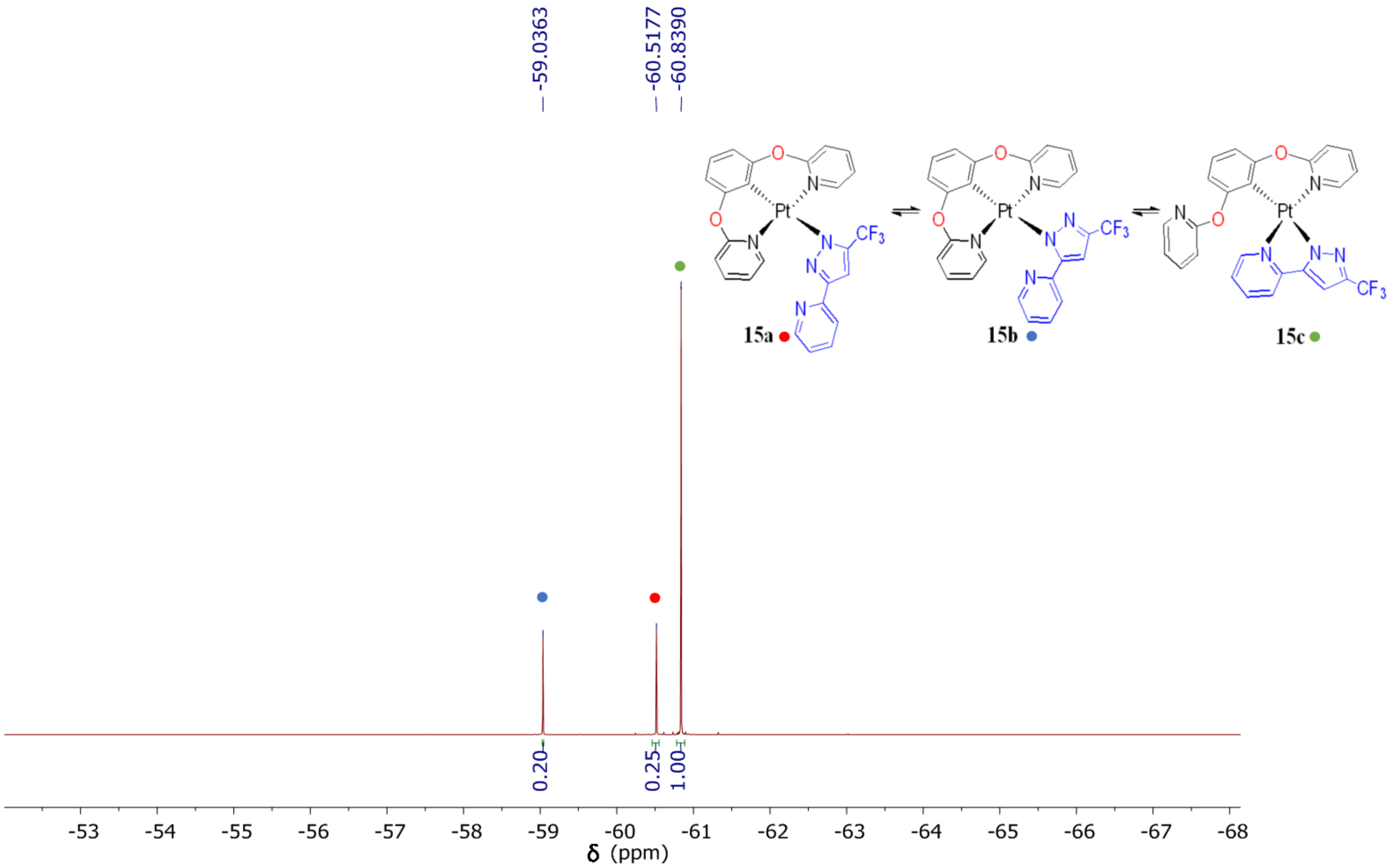

Figure S39. $^{19}F\{^1H\}$ NMR spectrum (282.40 MHz, CD_2Cl_2, 298 K) of the isomeric mixture of complexes **15a**, **15b** and **15c**.

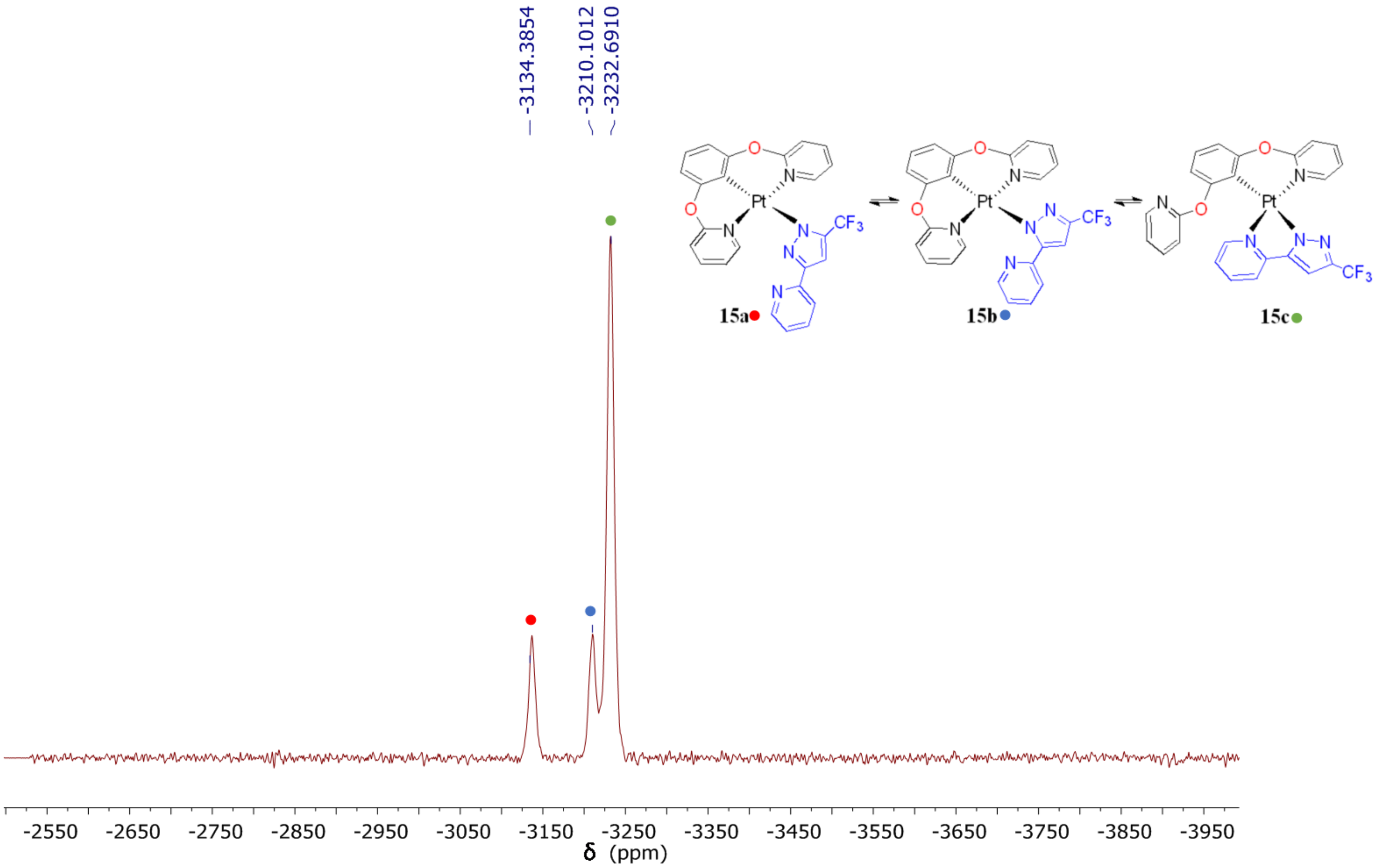

Figure S40. $^{195}Pt\{^1H\}$ NMR spectrum (85.6 MHz, CD_2Cl_2, 298 K) of the isomeric mixture of complexes **15a**, **15b** and **15c**.

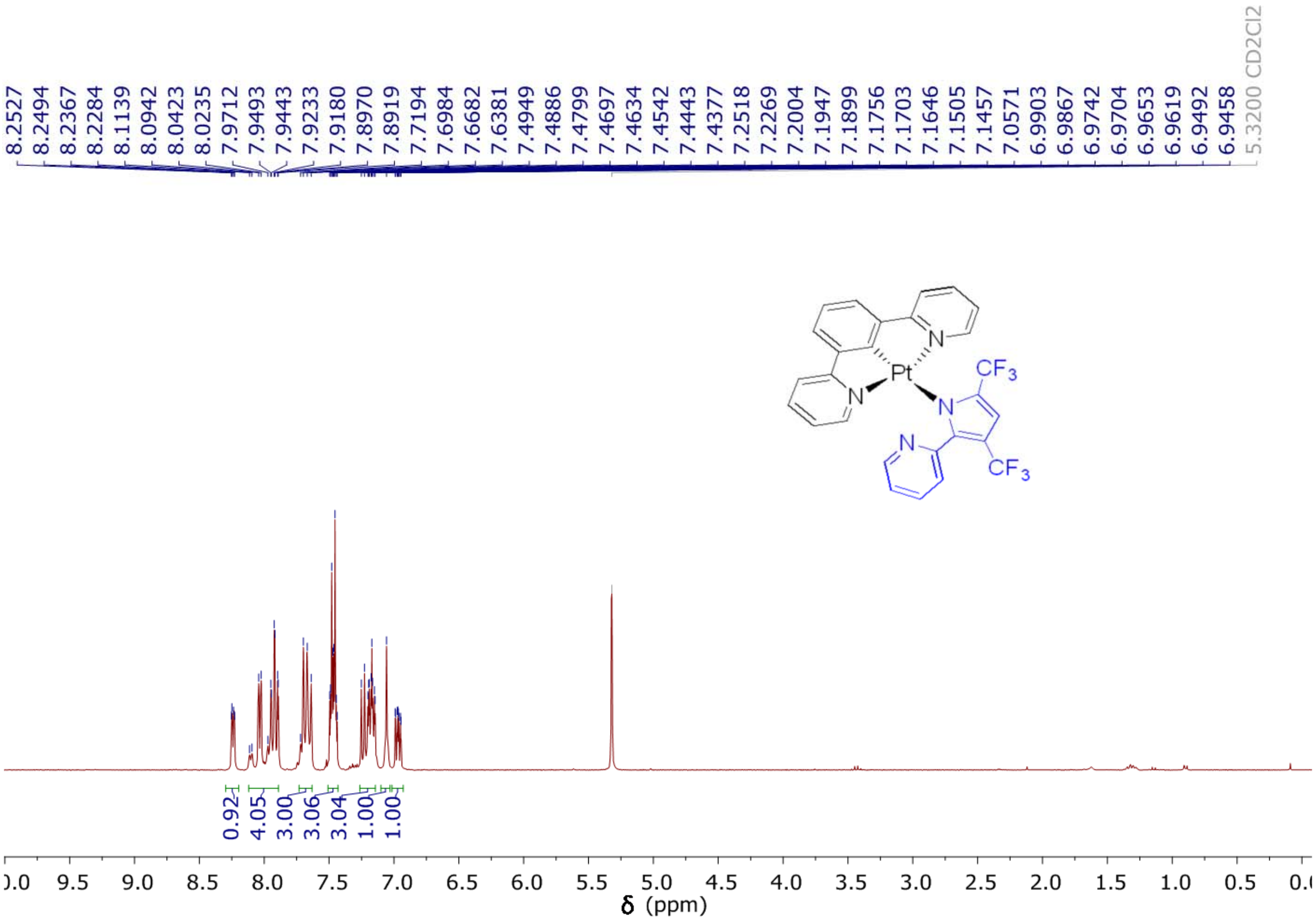

Figure S41. ^{1}H NMR spectrum (300.13 MHz, CD_2Cl_2, 298 K) of complex **16**.

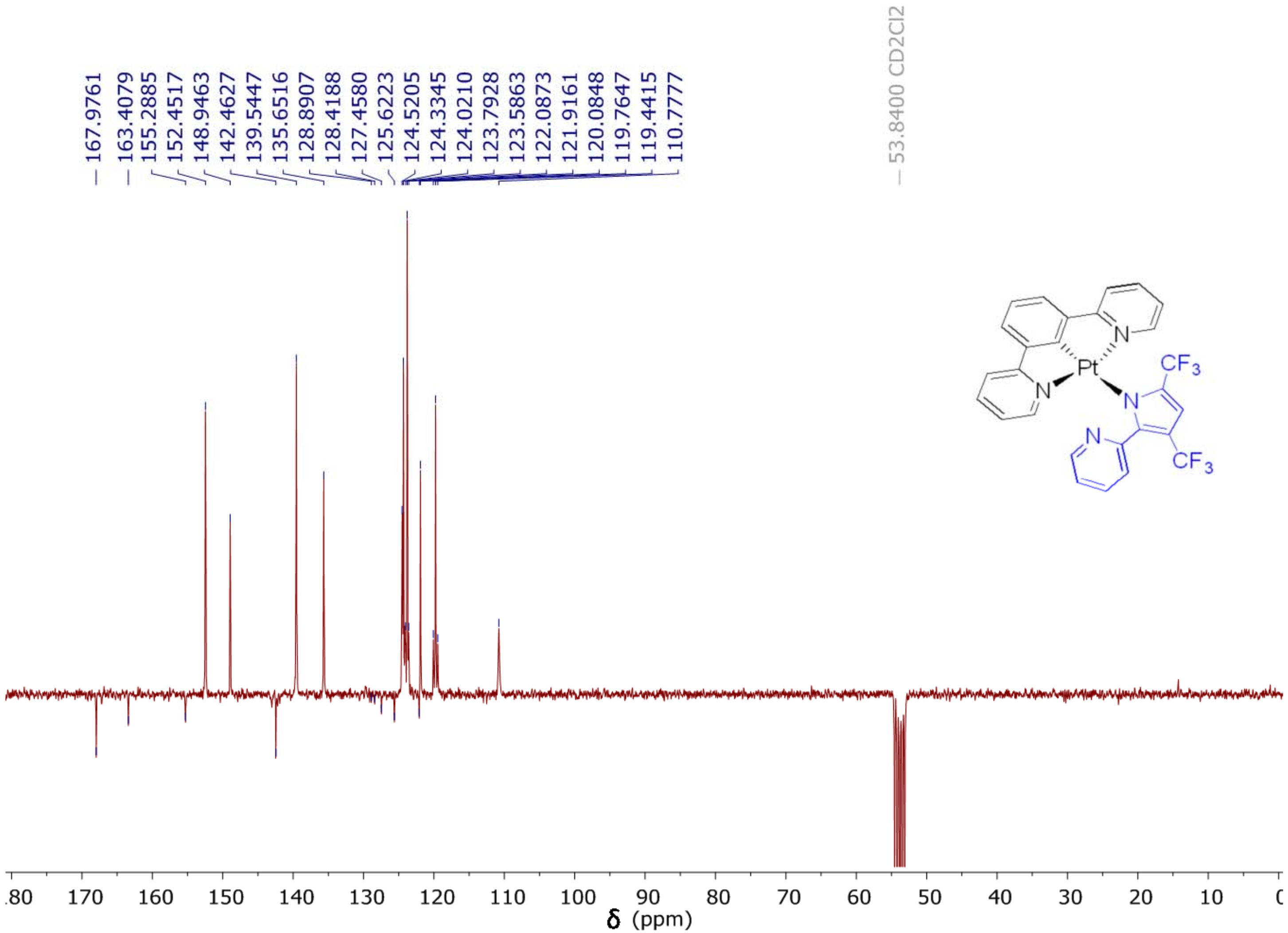

Figure S42. $^{13}C\{^{1}H\}$-apt NMR spectrum (75.48 MHz, CD_2Cl_2, 298 K) of complex **16**.

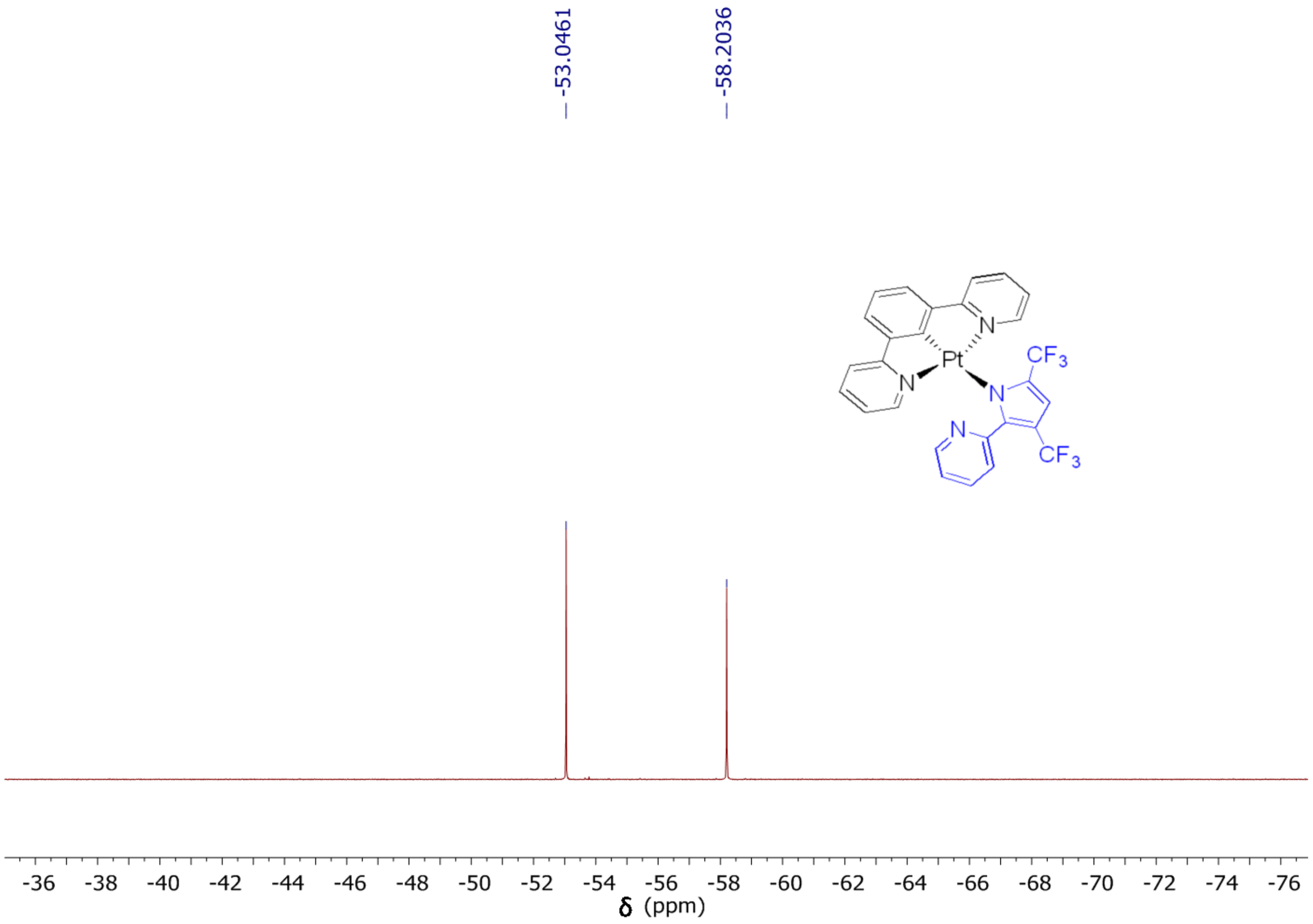

Figure S43. $^{19}F\{^{1}H\}$ NMR spectrum (282.40 MHz, CD_2Cl_2, 298 K) of complex **16**.

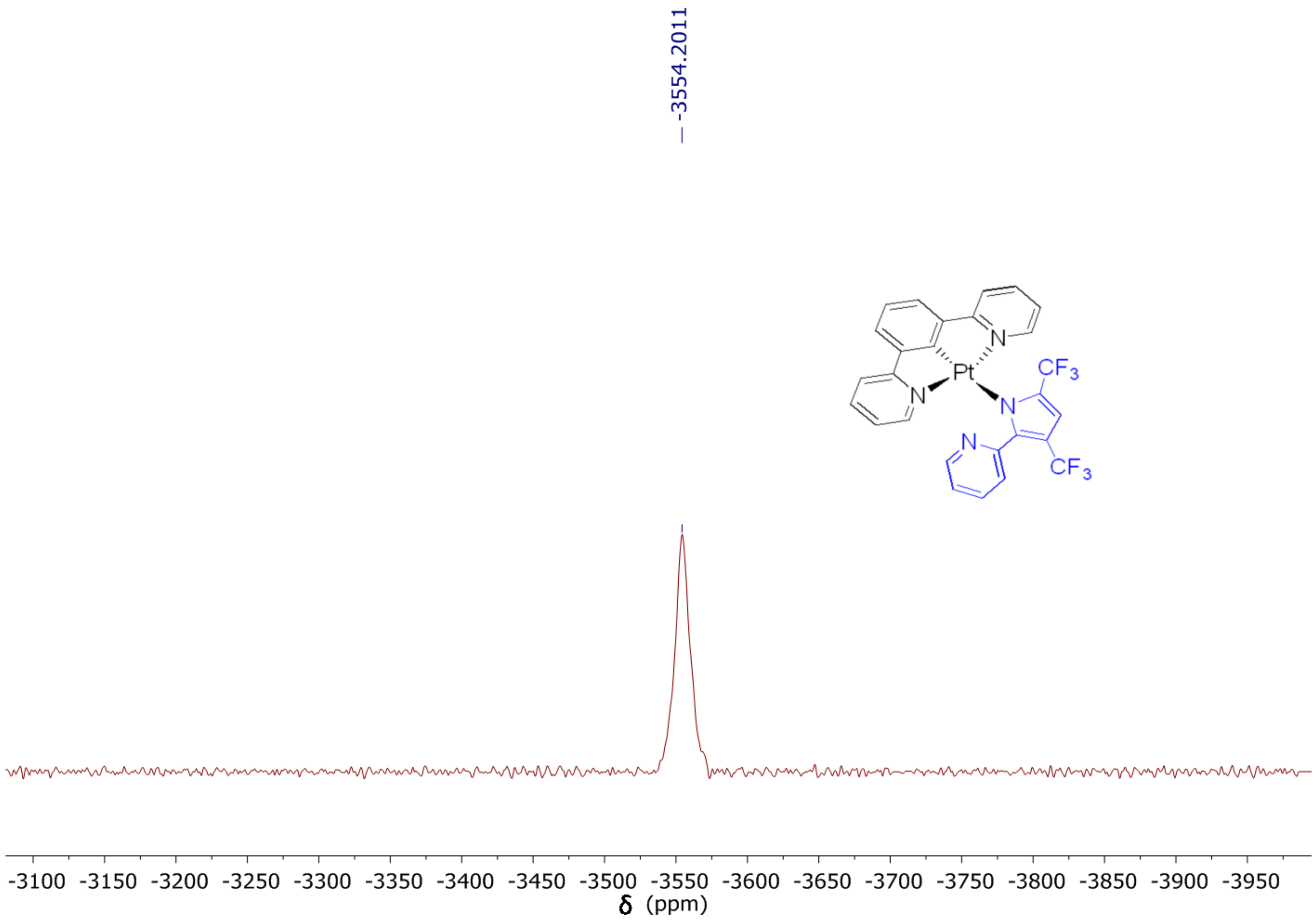

Figure S44. $^{195}Pt\{^1H\}$ NMR spectrum (85.6 MHz, CD_2Cl_2, 298 K) of complex **16**.

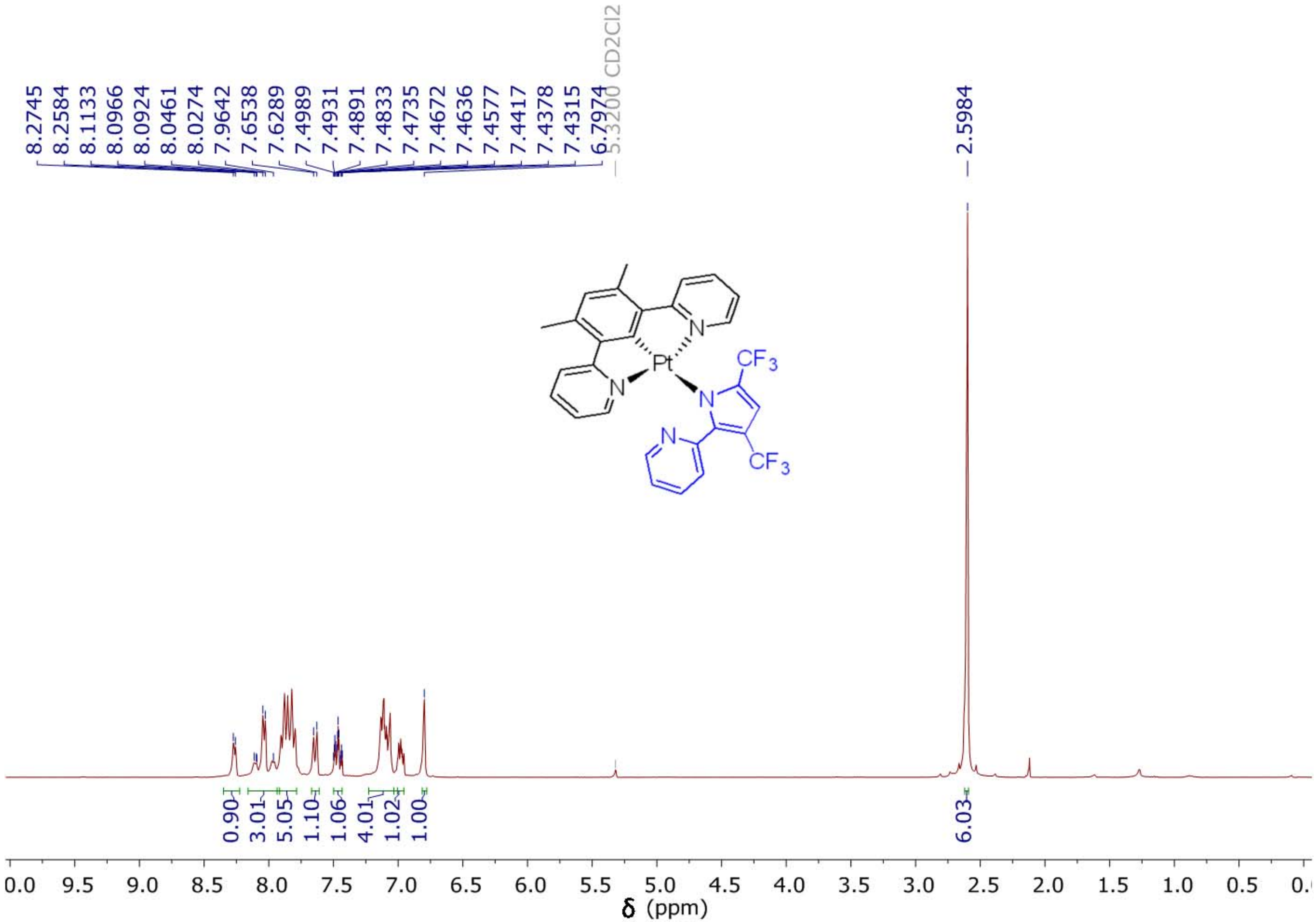

Figure S45. ^{1}H NMR spectrum (300.13 MHz, CD_2Cl_2, 298 K) of complex **17**.

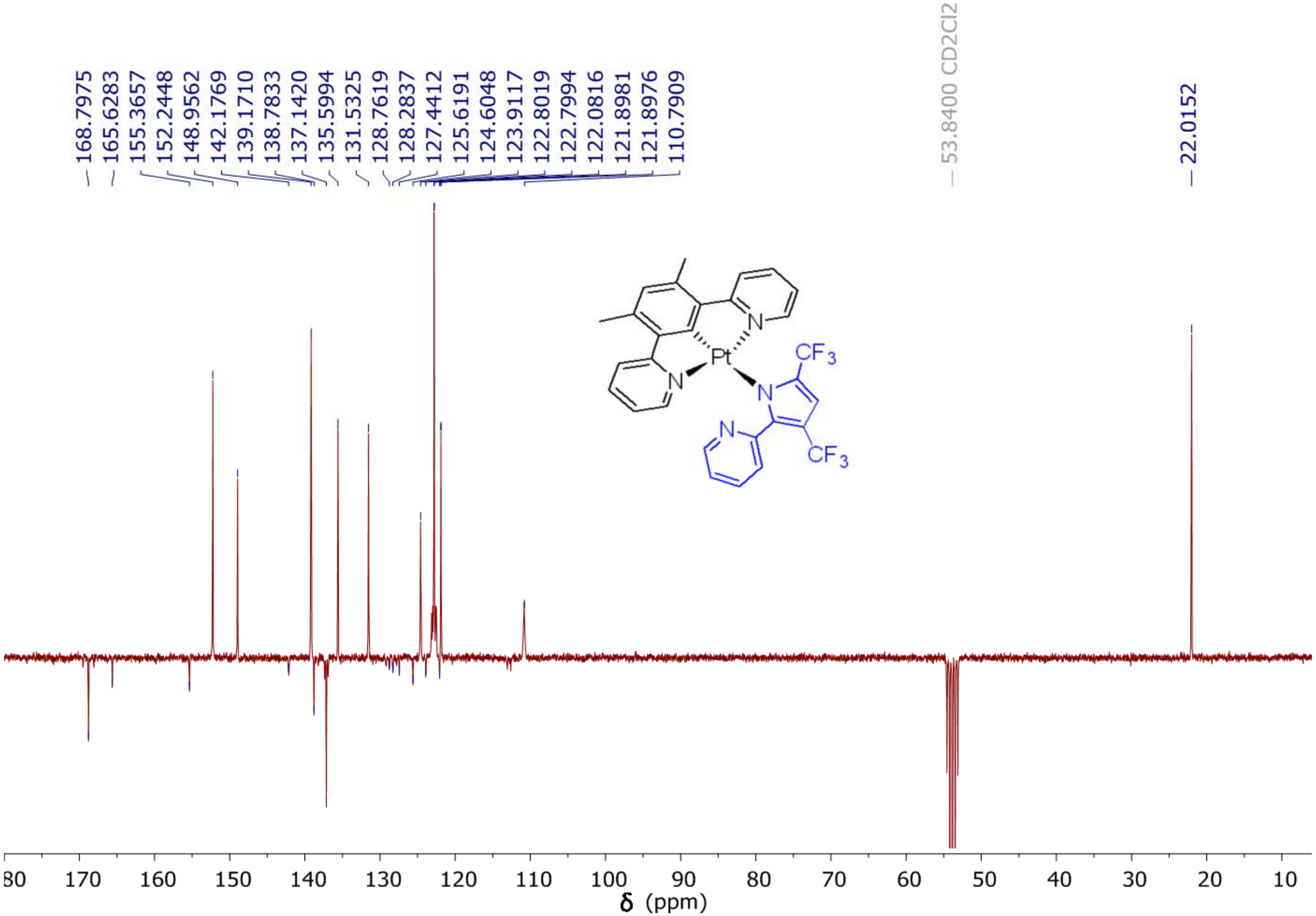

Figure S46. $^{13}C\{^{1}H\}$-apt NMR spectrum (75.48 MHz, CD_2Cl_2, 298 K) of complex **17**.

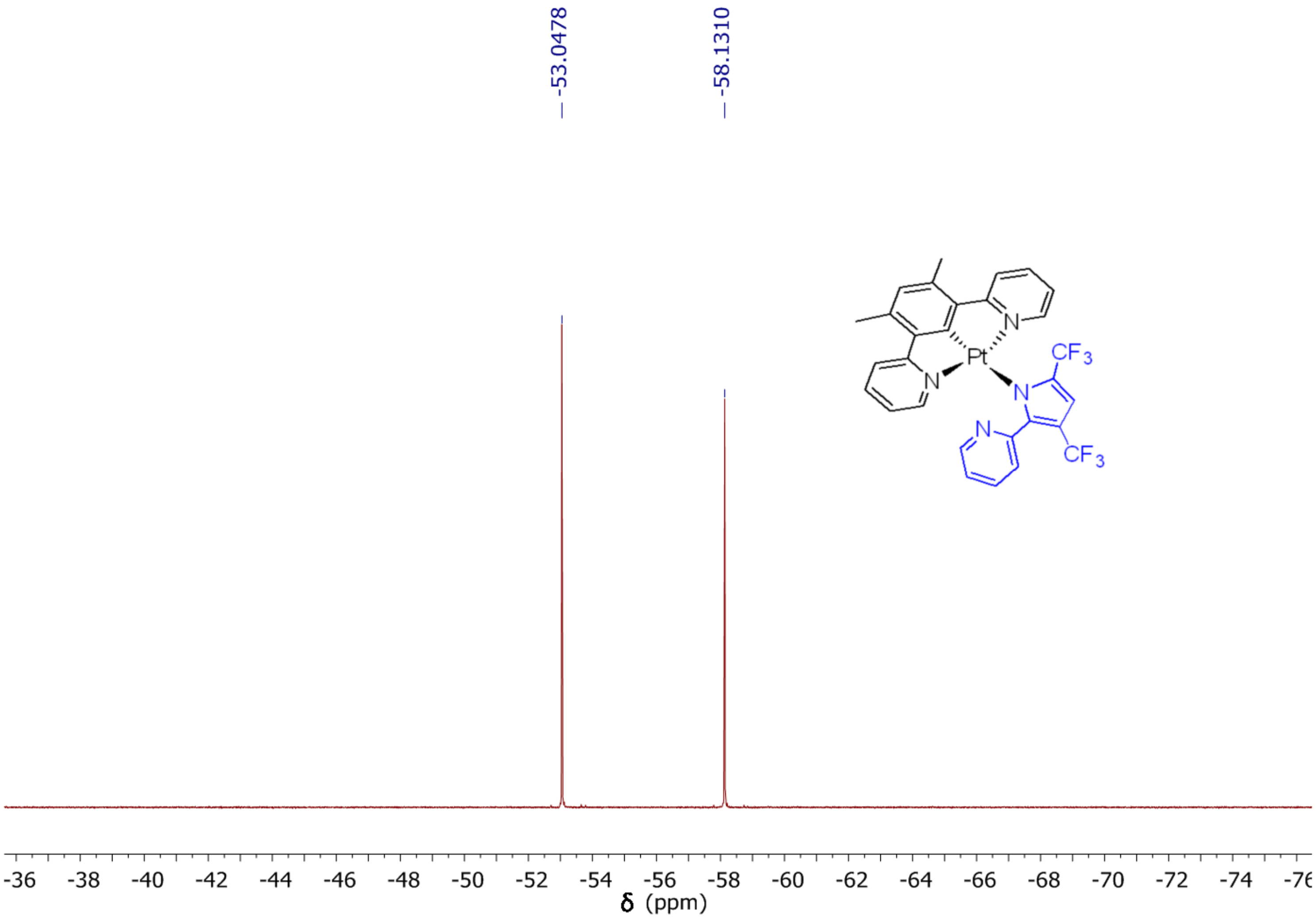

Figure S47. $^{19}F\{^{1}H\}$ NMR spectrum (282.40 MHz, CD_2Cl_2, 298 K) of complex **17**.

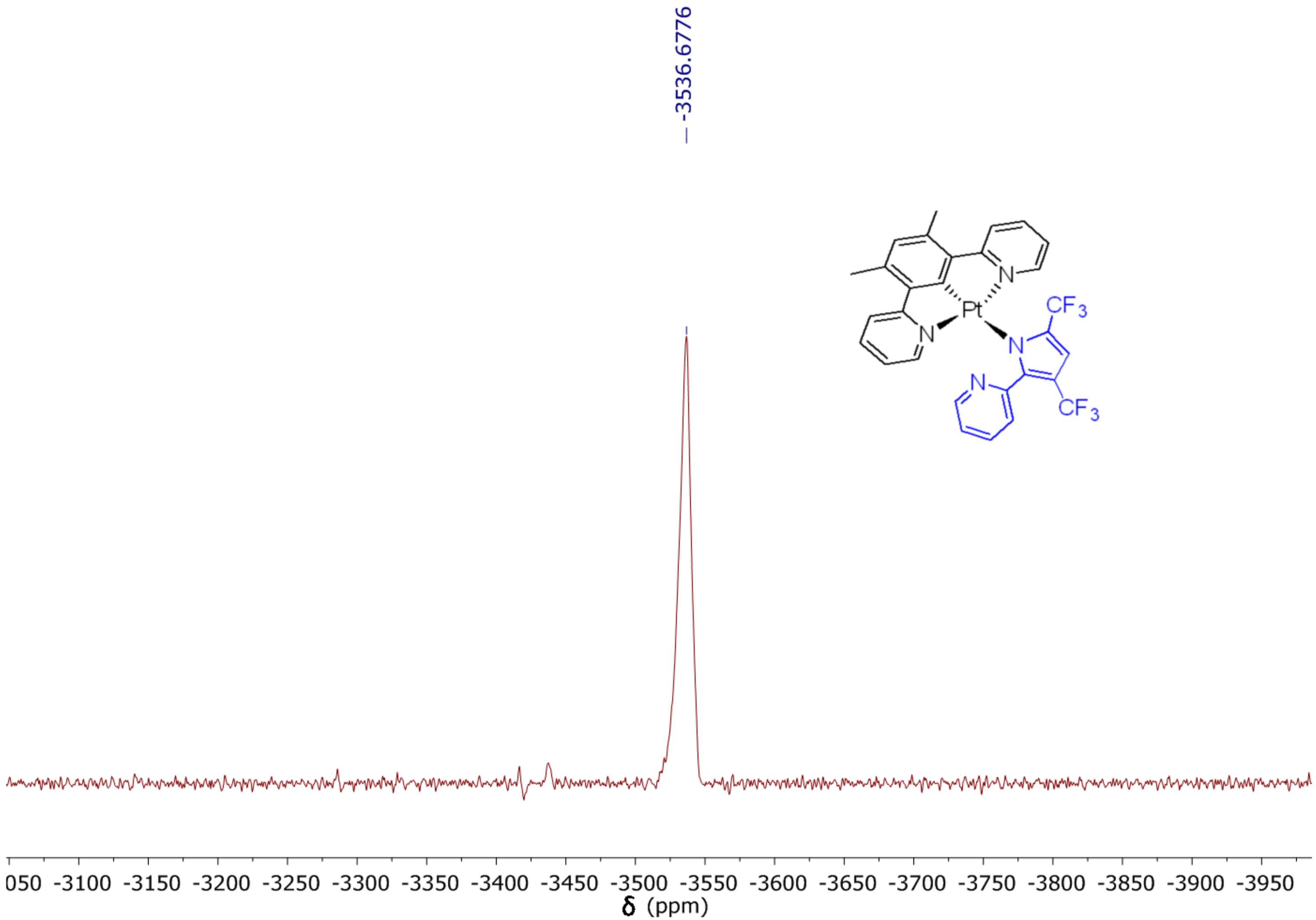

Figure S48. $^{195}Pt\{^1H\}$ NMR spectrum (85.6 MHz, CD_2Cl_2, 298 K) of complex **17**.

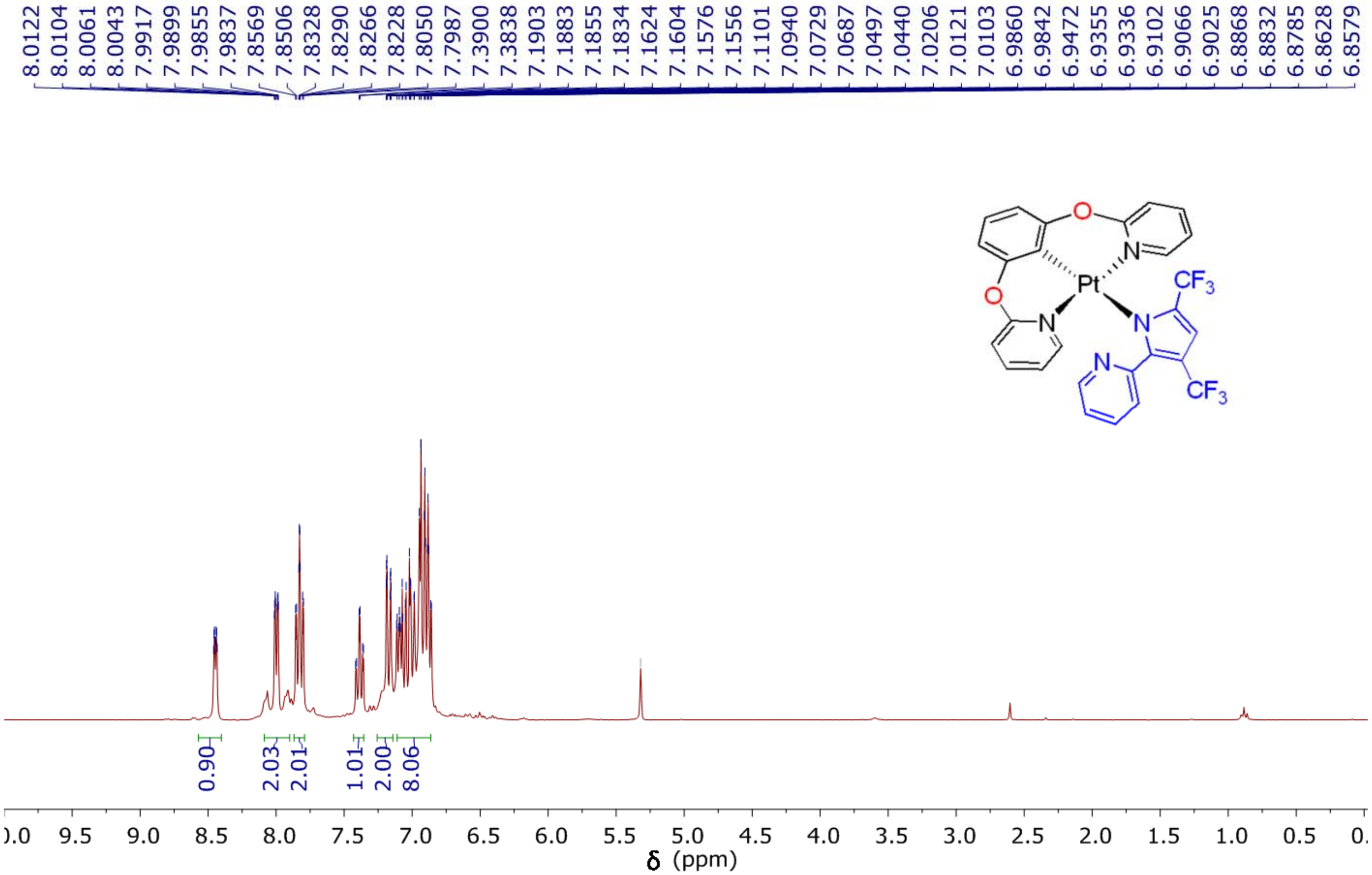

Figure S49. ^{1}H NMR spectrum (300.13 MHz, CD_2Cl_2, 298 K) of complex **18**.

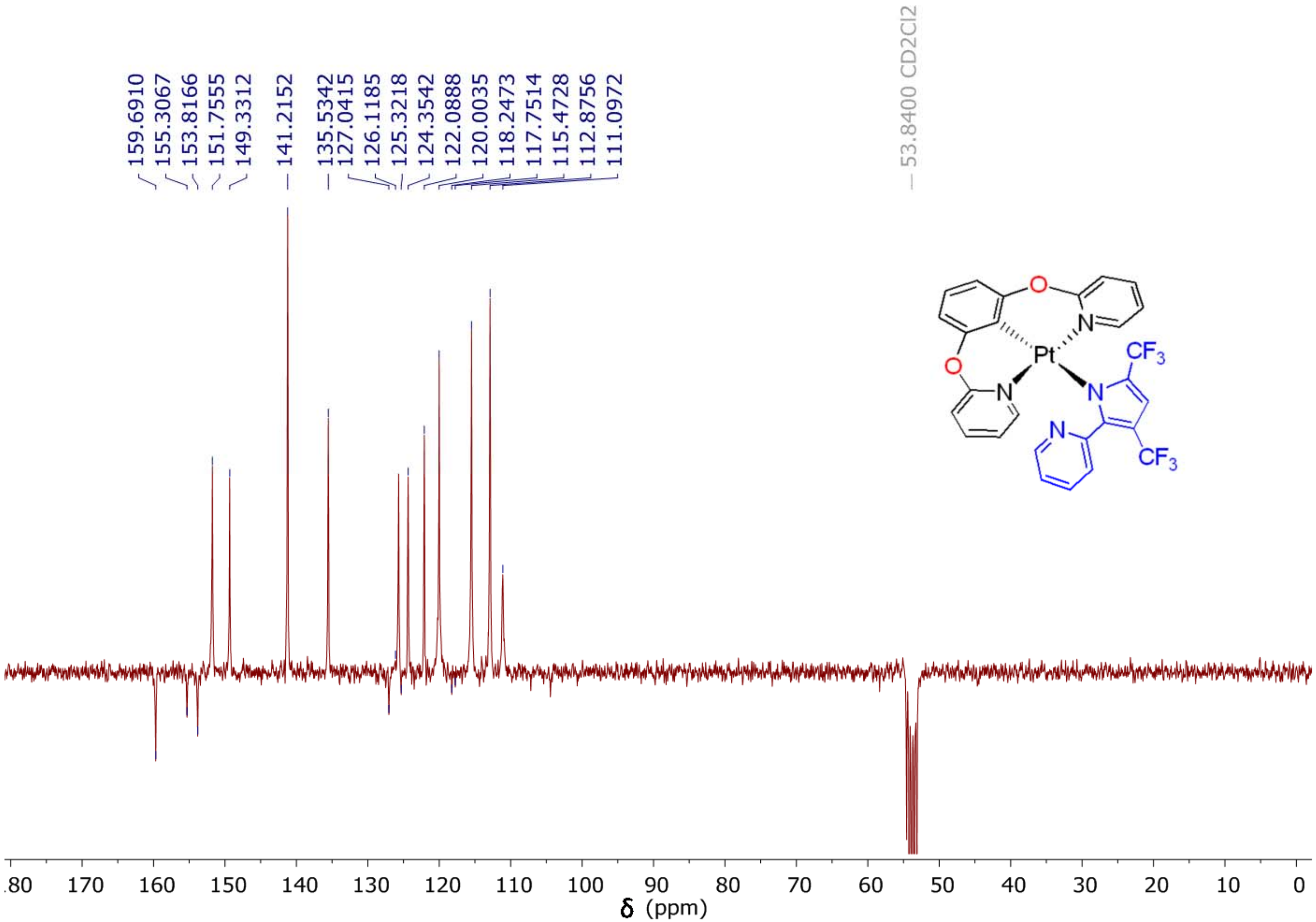

Figure S50. $^{13}C\{^1H\}$-apt NMR spectrum (100.62 MHz, CD_2Cl_2, 298 K) of complex **18**.

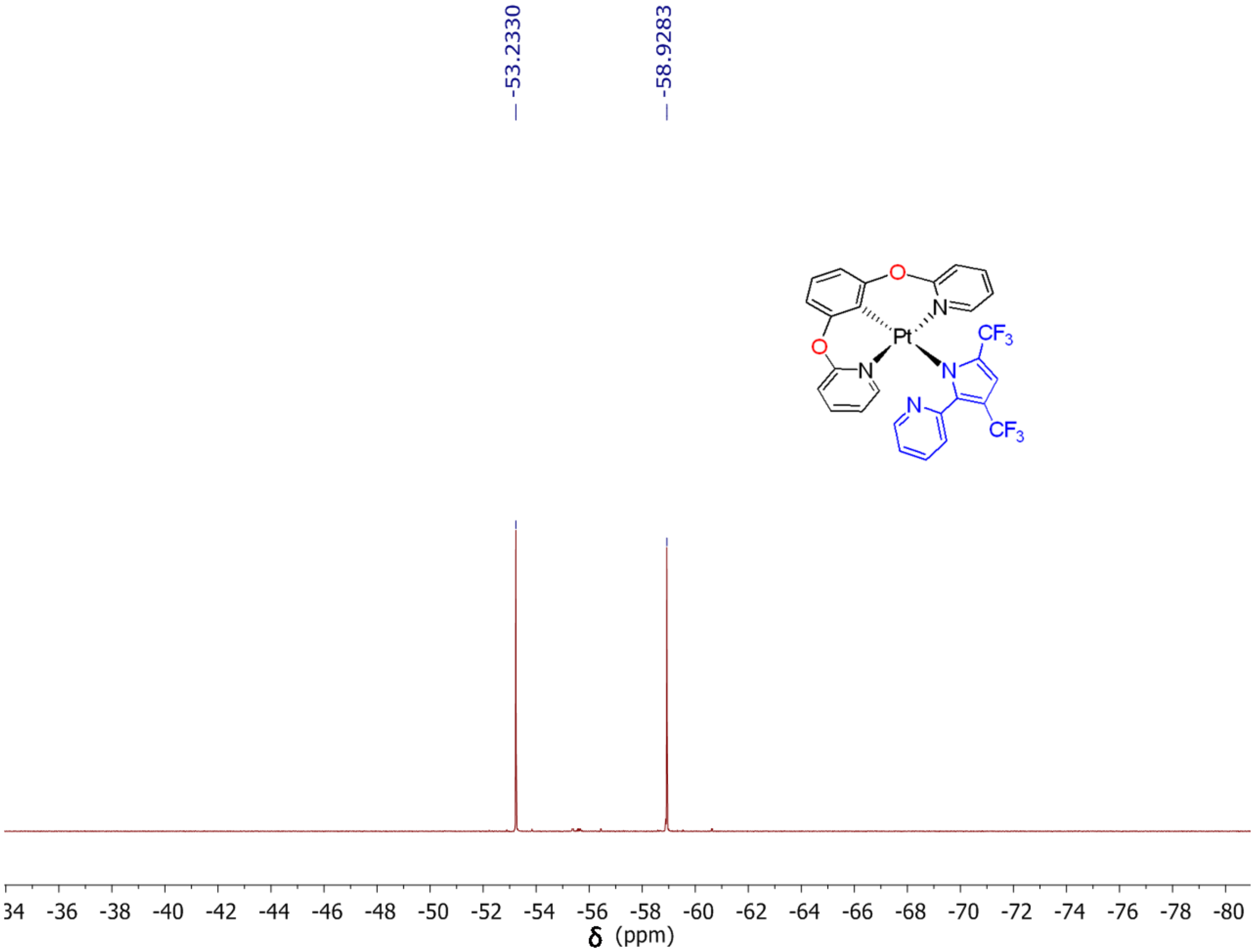

Figure S51. $^{19}F\{^1H\}$ NMR spectrum (282.40 MHz, CD_2Cl_2, 298 K) of complex **18**.

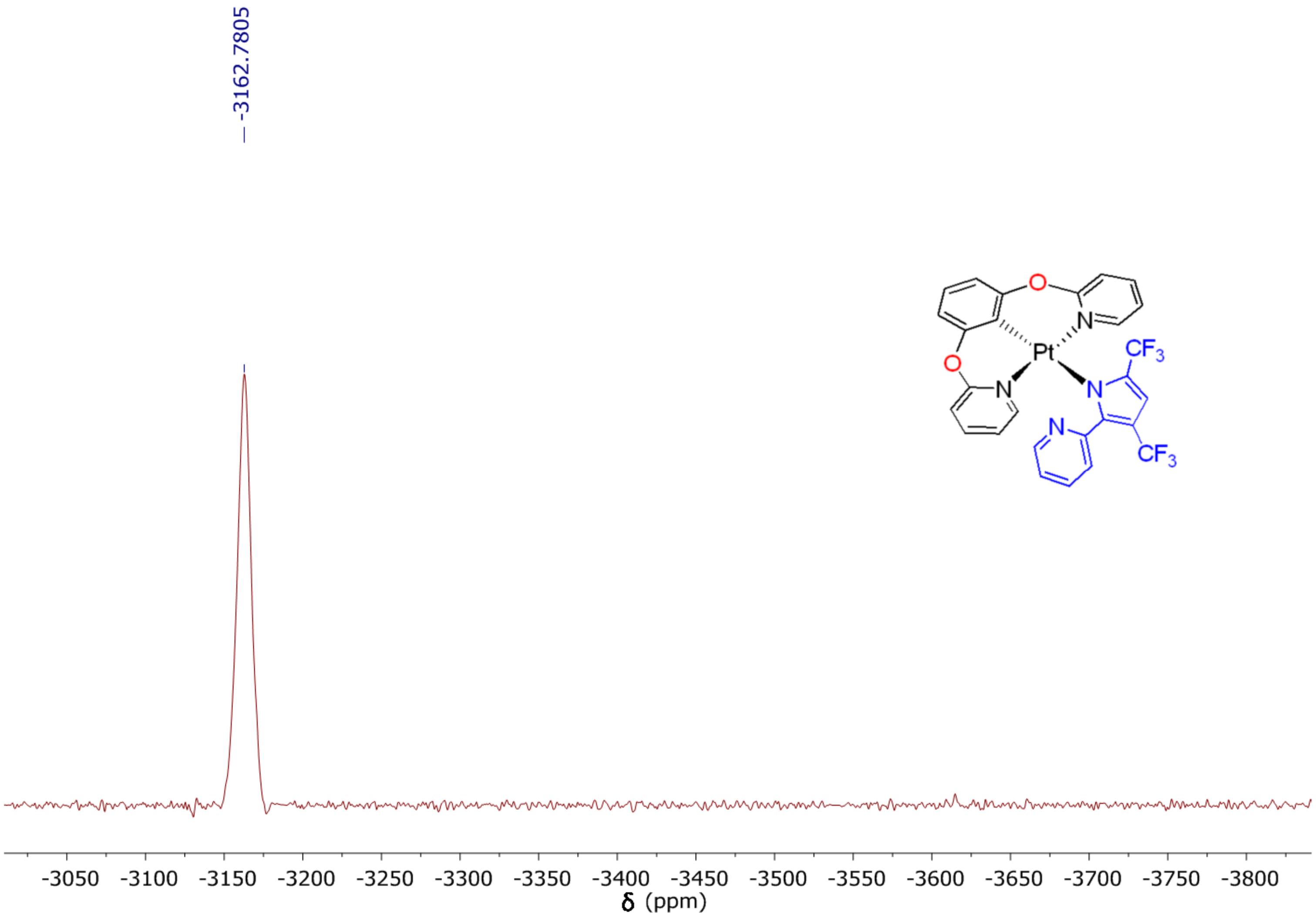

Figure S52. $^{195}Pt\{^{1}H\}$ NMR spectrum (85.6 MHz, CD_2Cl_2, 298 K) of complex **18**.

• Equilibrium and Kinetic Analysis for Complexes 11 and 12

Equilibrium Analysis. Integration of the $^{19}F\{^1H\}$ NMR spectra (376 MHz, CD_2Cl_2) of complexes **11** and **12** in the temperature range 298 to 223 K provided the equilibrium constants at each temperature (K_{11} = [**11a**]/[**11b**]; K_{12} = [**12a**]/[**12b**]). The values of $\Delta H°$ and $\Delta S°$ were derived from the Van't Hoff plot of ln K vs 1/T. Error analysis assumed a 10% error in the rate constant and 1 K in the temperature. Errors were computed by published methods.[4]

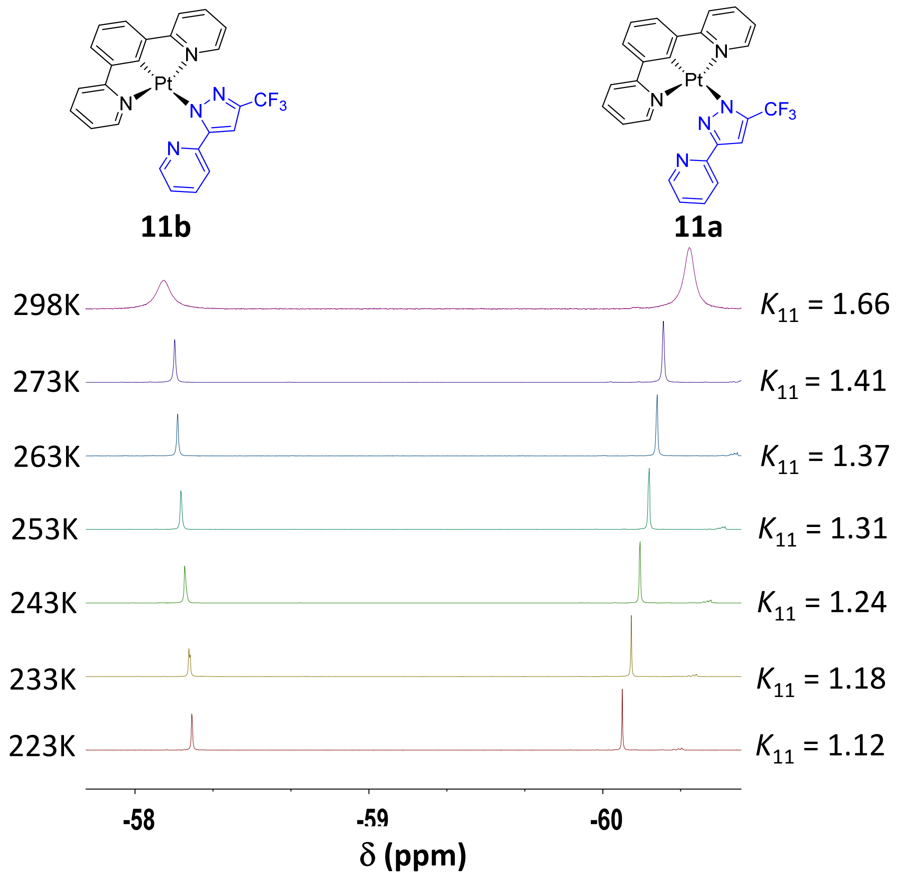

Figure S53. Variable temperature $^{19}F\{^1H\}$ NMR spectra (376 MHz) of a 10 mg sample of **11** in 0.4 mL of CD_2Cl_2.

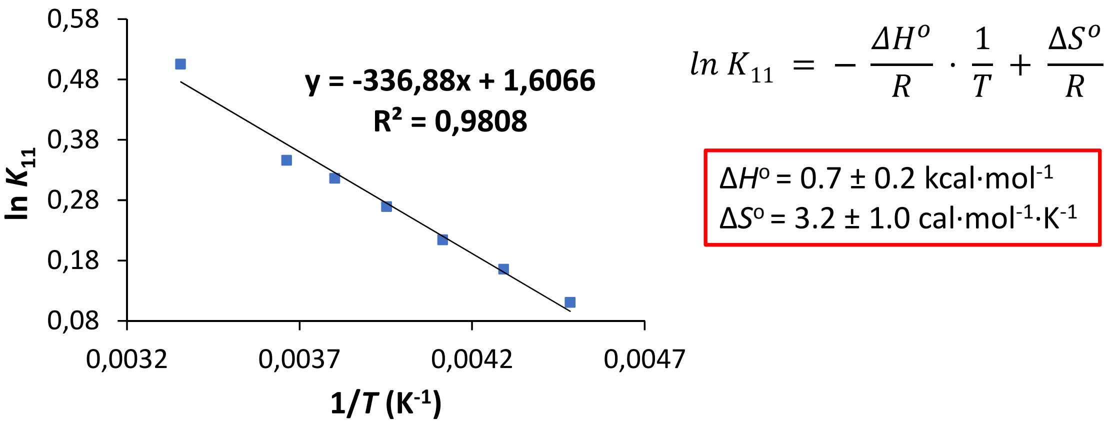

Figure S54. Van't Hoff plot of the equilibrium between **11a** and **11b**.

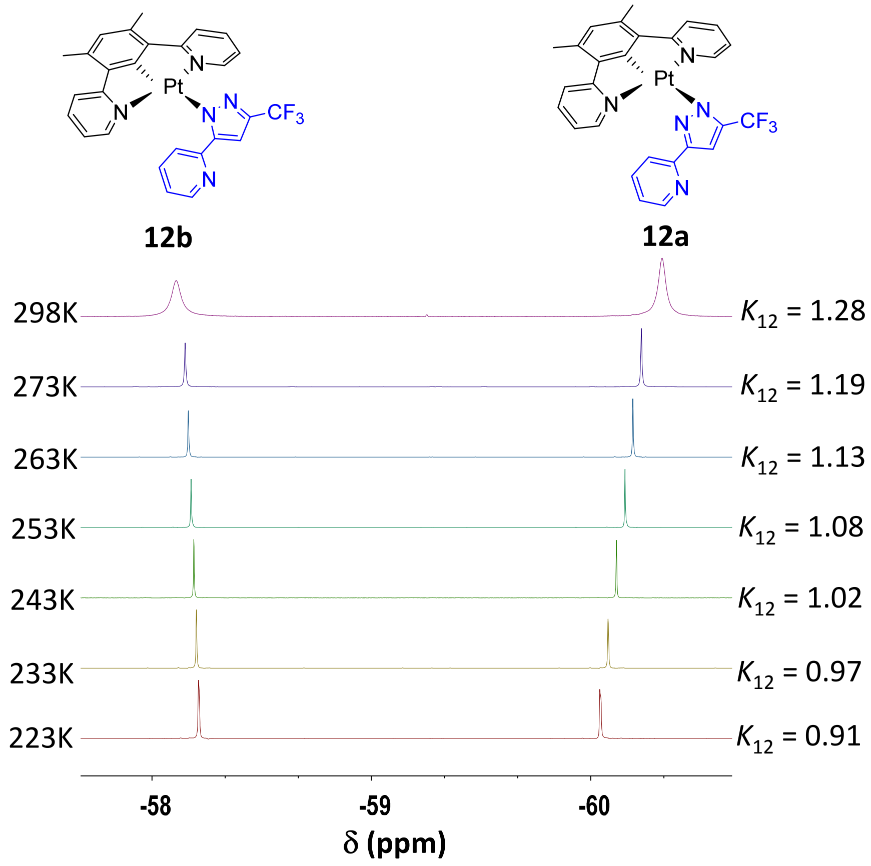

Figure S55. Variable temperature $^{19}F\{^1H\}$ NMR spectra (376 MHz) of a 10 mg sample of **12** in 0.4 mL of CD_2Cl_2.

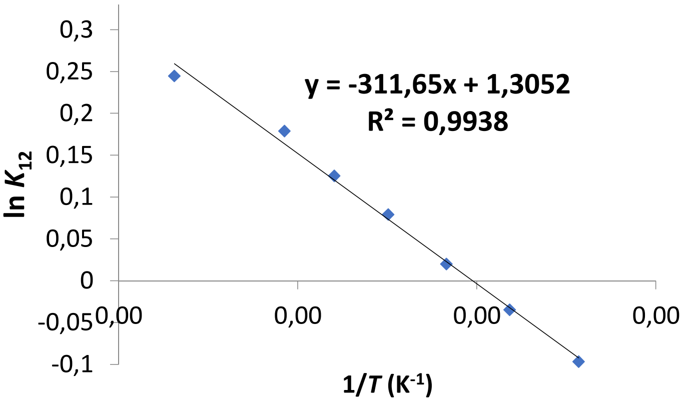

$$ln\,K_{12} = -\frac{\Delta H^{o}}{R}\cdot\frac{1}{T} + \frac{\Delta S^{o}}{R}$$

ΔH^{o} = 0.6 ± 0.2 kcal·mol^{-1}
ΔS^{o} = 2.6 ± 1.0 cal·mol^{-1}·K^{-1}

Figure S56. Van't Hoff plot of the equilibrium between **12a** and **12b**.

Kinetic Analysis. Line-shape analysis of the $^{19}F\{^{1}H\}$ NMR spectra of complexes **11** and **12** was achieved using the gNMR 4.1 (Cherwell Scientific Limited) simulation program. The rate constants at each temperature were obtained by fitting calculated to experimental spectra by full line shape iterations. The activation parameters $\Delta H^{\ddagger}$ and $\Delta S^{\ddagger}$ were calculated by least-squares fit of ln(k/T) *vs* 1/T (Eyring equation). Error analysis assumed a 10% error in the rate constant and 1 K in the temperature. Error were computed by published methods.[4]

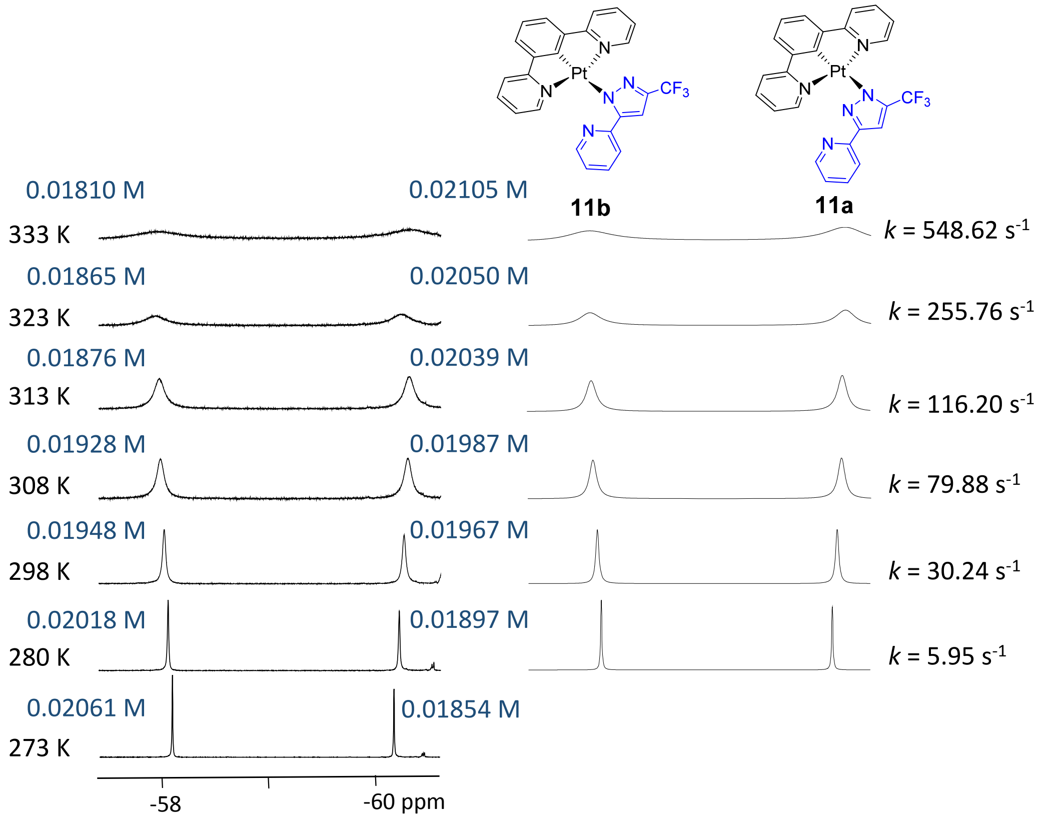

Figure S57. (left) Observed variable temperature $^{19}F\{^{1}H\}$ NMR spectra (376 MHz) of a 10 mg sample of **11** in 0.4 mL of $CDCl_3$. (right) Simulated spectra. The concentration of each species was derived from the integral; temperatures and rate constants are also provided.

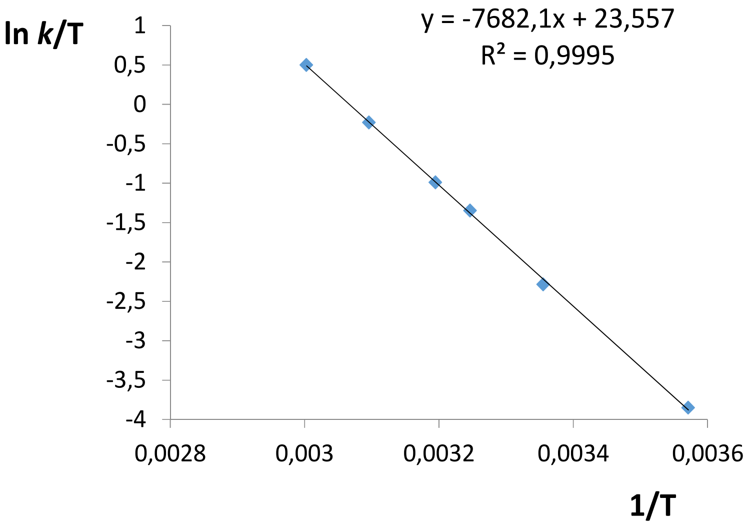

Figure S58. Eyring plot for **11**.

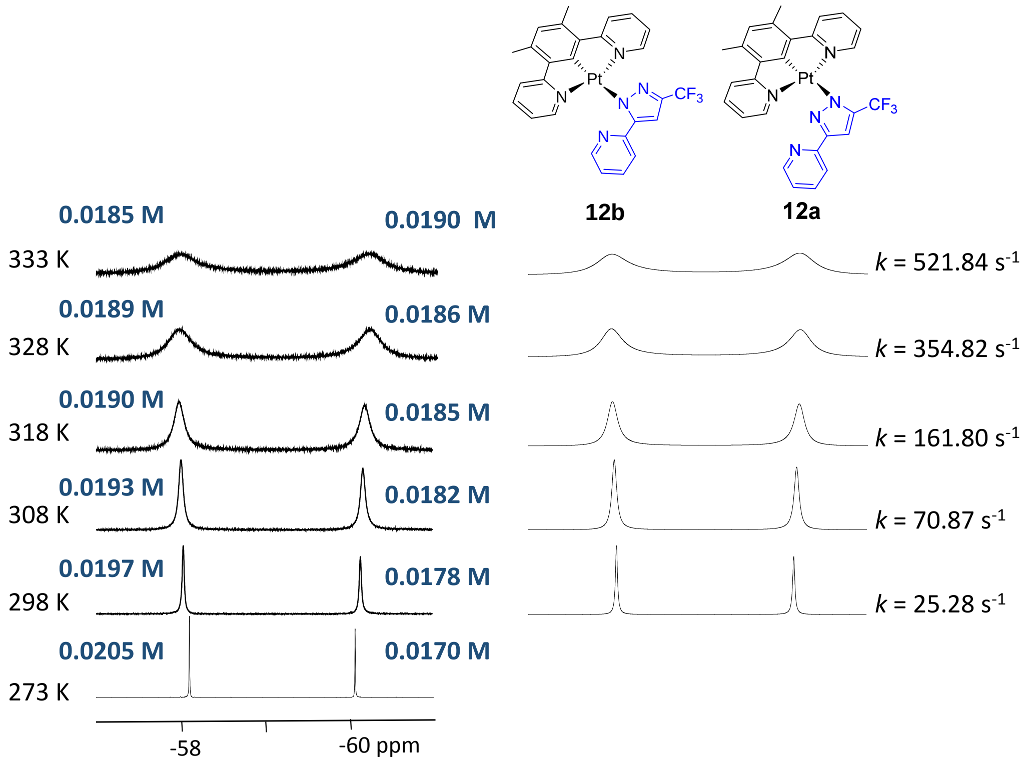

Figure S59. (left) Observed variable temperature $^{19}F\{^{1}H\}$ NMR spectra (376 MHz) of a 10 mg sample of **12** in 0.4 mL of $CDCl_3$. (right) Simulated spectra. The concentration of each species was derived from the integral; temperatures and rate constants are also provided.

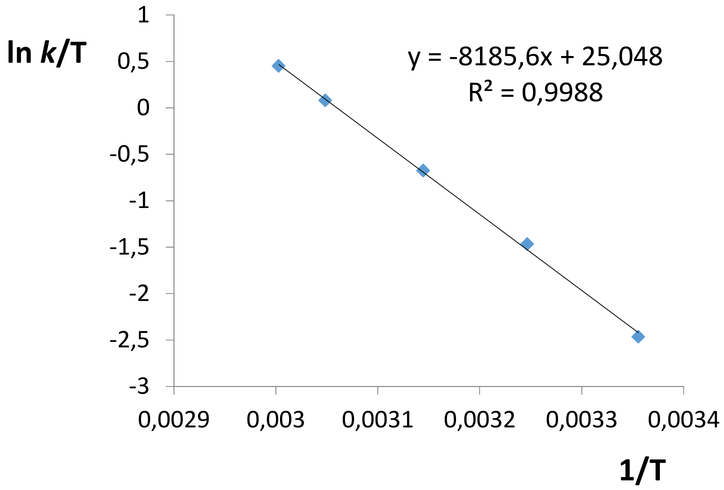

Figure S60. Eyring plot for **12**.

• Structural Analysis of Complexes 9, 12b, 15c, 17, and 18.

X-ray data were collected on a APEX CCD (**9**), DUO CCD (**15c**, **17**), and D8 Venture Bruker diffractometers (**12b**, **18**) (Mo radiation, λ = 0.71073 Å). The crystals were cooled with a nitrogen flow with an Oxford Cryosystems system. Data were corrected for absorption by using a multiscan method applied with the SADABS program.[5] The structures were solved by Patterson or direct methods and refined by full-matrix least squares on F^2 with SHELXL2019,[6] including isotropic and subsequently anisotropic displacement parameters. The hydrogen atoms were observed in the last Fourier Maps or calculated, and refined freely or using a restricted riding model.

Crystal data for **9** (CCDC 2243712): $C_{26}H_{21}N_5Pt$, CH_2Cl_2, M_W 683.49, yellow, irregular block (0.346 x 0.100 x 0.070 mm^3), monoclinic, space group C2/c, *a*: 21.4115(12) Å, *b*: 10.9645(6) Å, *c*: 20.5787(12) Å, *β*: 90.7310(10)°, *V* = 4830.8(5) $Å^3$, *Z* = 8, *Z'* = 1, D_{calc}: 1.880 g cm^{-3}, F(000): 2656, T = 100(2) K, μ 6.058 mm^{-1}. 27250 measured reflections (2θ: 3-57°, ω and φ scans 0.5°), 5883 unique (R_{int} = 0.0451); min./max. transm. factors 0.631/0.862. Final agreement factors were R^1 = 0.0259 (4843 observed reflections, I > 2σ(I)) and wR^2 = 0.0567; data/restraints/parameters 5883/0/318; GoF = 1.010. Largest peak and hole 1.247 (close to Pt atoms) and -1.064 e/ $Å^3$.

Crystal data for **12b** (CCDC 2243710): $C_{27}H_{20}F_3N_5Pt$, M_W 666.57, yellow, irregular block (0.300 x 0.039 x 0.025 mm^3), monoclinic, space group P21/n, *a*: 14.2188(4) Å, *b*: 7.46787(17) Å, *c*: 22.1503(6) Å, *β*: 105.8387(9)°, *V* = 2262.71(10) $Å^3$, *Z* = 4, *Z'* = 1, D_{calc}: 1.957 g cm^{-3}, F(000): 1288, T = 100(2) K, μ 6.254 mm^{-1}. 100901 measured reflections (2θ: 3-57°, ω and φ scans 0.5°), 6919 unique (R_{int} = 0.0345); min./max. transm. factors 0.671/0.862. Final agreement factors were R^1 = 0.0247 (6513 observed reflections, I > 2σ(I)) and wR^2 = 0.0641; data/restraints/parameters 6919/0/327; GoF = 1.040. Largest peak and hole 2.034 (close to Pt atoms) and -2.171 e/ $Å^3$.

Crystal data for **15c** (CCDC 2243711): $C_{25}H_{16}F_3N_5O_2Pt$, M_W 670.52, colourless, irregular block, (0.143 x 0.121 x 0.039 mm^3), monoclinic, space group $P2_1/c$, *a*: 11.5710(12) Å, *b*: 14.3012(14) Å, *c*: 14.3964(15) Å, *β*: 111.542(2)°, *V* = 2215.9(4) $Å^3$, *Z* = 4, *Z'* = 2, D_{calc}: 2.010 g cm^{-3}, F(000): 1288, T = 120(2) K, μ 6.393 mm^{-1}. 58524 measured reflections (2θ: 3-51°, ω and φ scans 0.5°), 6158 unique (R_{int} = 0.0810); min./max. transm. factors 0.689/0.862. Final agreement factors were R^1 = 0.0312 (4475 observed reflections, I > 2σ(I)) and wR^2 = 0.0631; data/restraints/parameters

6158/18/327; GoF = 1.063. Largest peak and hole 1.278 (close to Pt atoms) and -1.016 e/ Å^3.

Crystal data for **17** (CCDC 2243709): $C_{29}H_{20}F_6N_4Pt$, M_W 733.58, yellow, irregular block, (0.300 x 0.082 x 0.061 mm^3), orthorhombic, space group Pbca, *a*: 15.1474(17) Å, *b*: 9.6599(11) Å, *c*: 34.128(4) Å, V = 4993.7(10) Å^3, Z = 8, Z' = 1, D_{calc}: 1.951 g cm^{-3}, F(000): 2832, T = 120(2) K, μ 5.692 mm^{-1}. 37122 measured reflections (2θ: 3-57°, ω and φ scans 0.5^o), 7347 unique (R_{int} = 0.0629); min./max. transm. factors 0.569/0.862. Final agreement factors were R^1 = 0.0348 (4988 observed reflections, I > 2σ(I)) and wR^2 = 0.0753; data/restraints/parameters 7347/0/363; GoF = 1.007. Largest peak and hole 2.267 (close to Pt atoms) and -2.422 e/ Å^3.

Crystal data for **18** (CCDC 2243708): $C_{27}H_{16}F_6N_4O_2Pt$, M_W 737.53, yellow, irregular block (0.400 x 0.200 x 0.020 mm^3), orthorhombic, space group $P2_12_12_1$, *a*: 8.6432(15)Å , *b*: 16.542(2) Å, *c*: 17.644(3) Å, V = 2522.6(7) Å^3, Z = 4, Z' = 1, D_{calc}: 1.942 g cm^{-3}, F(000): 1416, T = 100(2) K, μ 5.641 mm^{-1}. 19789 measured reflections (2θ: 3-57°, ω and φ scans 0.5^o), 6181 unique (R_{int} = 0.0251); min./max. transm. factors 0.692/0.862. Final agreement factors were R^1 = 0.0148 (6063 observed reflections, I > 2σ(I)) and wR^2 = 0.0331; Flack parameter: -0.020(5); data/restraints/parameters 6181/0/362; GoF = 1.038. Largest peak and hole 0.496 (close to Pt atoms) and -0.335 e/ Å^3.

• **Computational Details and Energies of Calculated Complexes**

Computational Details. All calculations were performed at the DFT level using the B3LYP functional[8] supplemented with the Grimme's dispersion correction D3[9] as implemented in Gaussian09.[10] Pt atoms were described by means of an effective core potential SDD for the inner electron[11] and its associated double-ζ basis set for the outer ones, complemented with a set of f-polarization functions for platinum.[12] The 6-31G** basis set was used for the H, C, O, N, and F atoms.[13] All minima were verified to have no negative frequencies. The geometries were fully optimized in $CHCl_3$ (ε = 4.7113) or CH_2Cl_2 (ε = 8.93) solvent using the continuum SMD model.[14] We performed TD-DFT calculations at the same level of theory calculating the lowest 50 singlet-singlet excitations at the ground state S0. It should be noted that the singlet-triplet excitations are set to zero due to the neglect of spin-orbit coupling in the TDDFT calculations as implemented in G09. The UV/vis absorption spectra were obtained by using the GaussSum 3 software.[15] The phosphorescence emission compares well with the 0-0 transition calculated taking into account the zero point energies (zpe) of the geometries of both the optimized T_1 and S_0 states. The AIM calculations were performed with the AIMAII program.[16]

Energies of Calculated Complexes

TS-12a-12b ($CHCl_3$)

```
Zero-point correction=                          0.419492 (Hartree/Particle)
Thermal correction to Energy=                   0.449026
Thermal correction to Enthalpy=                 0.449970
Thermal correction to Gibbs Free Energy=        0.354957
Sum of electronic and zero-point Energies=      -1733.386196
Sum of electronic and thermal Energies=         -1733.356662
Sum of electronic and thermal Enthalpies=       -1733.355718
Sum of electronic and thermal Free Energies=    -1733.450730
```

12a ($CHCl_3$)

```
Zero-point correction=                          0.420398 (Hartree/Particle)
Thermal correction to Energy=                   0.450389
Thermal correction to Enthalpy=                 0.451333
Thermal correction to Gibbs Free Energy=        0.356208
Sum of electronic and zero-point Energies=      -1733.413576
Sum of electronic and thermal Energies=         -1733.383585
Sum of electronic and thermal Enthalpies=       -1733.382641
Sum of electronic and thermal Free Energies=    -1733.477765
```

12b ($CHCl_3$)

Zero-point correction= 0.420518 (Hartree/Particle)
Thermal correction to Energy= 0.450466
Thermal correction to Enthalpy= 0.451410
Thermal correction to Gibbs Free Energy= 0.356385
Sum of electronic and zero-point Energies= -1733.407919
Sum of electronic and thermal Energies= -1733.377971
Sum of electronic and thermal Enthalpies= -1733.377027
Sum of electronic and thermal Free Energies= -1733.472052

7-S_0 (CH_2Cl_2)

Zero-point correction= 0.359969 (Hartree/Particle)
Thermal correction to Energy= 0.383179
Thermal correction to Enthalpy= 0.384124
Thermal correction to Gibbs Free Energy= 0.304814
Sum of electronic and zero-point Energies= -1317.784410
Sum of electronic and thermal Energies= -1317.761200
Sum of electronic and thermal Enthalpies= -1317.760255
Sum of electronic and thermal Free Energies= -1317.839565

7-T_1 (CH_2Cl_2)

Zero-point correction= 0.356050 (Hartree/Particle)
Thermal correction to Energy= 0.379757
Thermal correction to Enthalpy= 0.380702
Thermal correction to Gibbs Free Energy= 0.299736
Sum of electronic and zero-point Energies= -1317.693096
Sum of electronic and thermal Energies= -1317.669388
Sum of electronic and thermal Enthalpies= -1317.668444
Sum of electronic and thermal Free Energies= -1317.749409

8-S_0 (CH_2Cl_2)

Zero-point correction= 0.416093 (Hartree/Particle)
Thermal correction to Energy= 0.442256
Thermal correction to Enthalpy= 0.443200
Thermal correction to Gibbs Free Energy= 0.357828
Sum of electronic and zero-point Energies= -1396.371530
Sum of electronic and thermal Energies= -1396.345367
Sum of electronic and thermal Enthalpies= -1396.344423
Sum of electronic and thermal Free Energies= -1396.429794

8-T_1 (CH_2Cl_2)

Zero-point correction= 0.411952 (Hartree/Particle)
Thermal correction to Energy= 0.438734
Thermal correction to Enthalpy= 0.439679
Thermal correction to Gibbs Free Energy= 0.351800

Sum of electronic and zero-point Energies= -1396.280629
Sum of electronic and thermal Energies= -1396.253846
Sum of electronic and thermal Enthalpies= -1396.252902
Sum of electronic and thermal Free Energies= -1396.340780

9-S_0 (CH_2Cl_2)

Zero-point correction= 0.387189 (Hartree/Particle)
Thermal correction to Energy= 0.411422
Thermal correction to Enthalpy= 0.412366
Thermal correction to Gibbs Free Energy= 0.331311
Sum of electronic and zero-point Energies= -1357.084383
Sum of electronic and thermal Energies= -1357.060150
Sum of electronic and thermal Enthalpies= -1357.059206
Sum of electronic and thermal Free Energies= -1357.140261

9-T_1 (CH_2Cl_2)

Zero-point correction= 0.383252 (Hartree/Particle)
Thermal correction to Energy= 0.408780
Thermal correction to Enthalpy= 0.409724
Thermal correction to Gibbs Free Energy= 0.324602
Sum of electronic and zero-point Energies= -1356.992795
Sum of electronic and thermal Energies= -1356.967268
Sum of electronic and thermal Enthalpies= -1356.966324
Sum of electronic and thermal Free Energies= -1357.051446

10-S_0 (CH_2Cl_2)

Zero-point correction= 0.443563 (Hartree/Particle)
Thermal correction to Energy= 0.471496
Thermal correction to Enthalpy= 0.472440
Thermal correction to Gibbs Free Energy= 0.383503
Sum of electronic and zero-point Energies= -1435.671661
Sum of electronic and thermal Energies= -1435.643728
Sum of electronic and thermal Enthalpies= -1435.642784
Sum of electronic and thermal Free Energies= -1435.731720

10-T_1 (CH_2Cl_2)

Zero-point correction= 0.439288 (Hartree/Particle)
Thermal correction to Energy= 0.467869
Thermal correction to Enthalpy= 0.468814
Thermal correction to Gibbs Free Energy= 0.376971
Sum of electronic and zero-point Energies= -1435.580698
Sum of electronic and thermal Energies= -1435.552116
Sum of electronic and thermal Enthalpies= -1435.551172
Sum of electronic and thermal Free Energies= -1435.643014

16-S_0 (CH_2Cl_2)

Zero-point correction= 0.380385 (Hartree/Particle)
Thermal correction to Energy= 0.411279
Thermal correction to Enthalpy= 0.412223
Thermal correction to Gibbs Free Energy= 0.314888
Sum of electronic and zero-point Energies= -1975.834108
Sum of electronic and thermal Energies= -1975.803214
Sum of electronic and thermal Enthalpies= -1975.802270
Sum of electronic and thermal Free Energies= -1975.899606

16-T_1 (CH_2Cl_2)

Zero-point correction= 0.376798 (Hartree/Particle)
Thermal correction to Energy= 0.408032
Thermal correction to Enthalpy= 0.408976
Thermal correction to Gibbs Free Energy= 0.310113
Sum of electronic and zero-point Energies= -1975.755709
Sum of electronic and thermal Energies= -1975.724476
Sum of electronic and thermal Enthalpies= -1975.723532
Sum of electronic and thermal Free Energies= -1975.822394

16-T_2 (CH_2Cl_2)

Zero-point correction= 0.375907 (Hartree/Particle)
Thermal correction to Energy= 0.407412
Thermal correction to Enthalpy= 0.408356
Thermal correction to Gibbs Free Energy= 0.308946
Sum of electronic and zero-point Energies= -1975.741586
Sum of electronic and thermal Energies= -1975.710082
Sum of electronic and thermal Enthalpies= -1975.709138
Sum of electronic and thermal Free Energies= -1975.808548

17-S_0 (CH_2Cl_2)

Zero-point correction= 0.436418 (Hartree/Particle)
Thermal correction to Energy= 0.470218
Thermal correction to Enthalpy= 0.471162
Thermal correction to Gibbs Free Energy= 0.368582
Sum of electronic and zero-point Energies= -2054.421712
Sum of electronic and thermal Energies= -2054.387911
Sum of electronic and thermal Enthalpies= -2054.386967
Sum of electronic and thermal Free Energies= -2054.489547

17-T_1 (CH_2Cl_2)

Zero-point correction= 0.433170 (Hartree/Particle)
Thermal correction to Energy= 0.467335
Thermal correction to Enthalpy= 0.468280

Thermal correction to Gibbs Free Energy= 0.363555
Sum of electronic and zero-point Energies= -2054.341204
Sum of electronic and thermal Energies= -2054.307039
Sum of electronic and thermal Enthalpies= -2054.306095
Sum of electronic and thermal Free Energies= -2054.410819

17-T_2 (CH_2Cl_2)

Zero-point correction= 0.432317 (Hartree/Particle)
Thermal correction to Energy= 0.466757
Thermal correction to Enthalpy= 0.467701
Thermal correction to Gibbs Free Energy= 0.362338
Sum of electronic and zero-point Energies= -2054.329932
Sum of electronic and thermal Energies= -2054.295493
Sum of electronic and thermal Enthalpies= -2054.294549
Sum of electronic and thermal Free Energies= -2054.399912

18-S_0 (CH_2Cl_2)

Zero-point correction= 0.388343 (Hartree/Particle)
Thermal correction to Energy= 0.421028
Thermal correction to Enthalpy= 0.421972
Thermal correction to Gibbs Free Energy= 0.320924
Sum of electronic and zero-point Energies= -2126.255922
Sum of electronic and thermal Energies= -2126.223236
Sum of electronic and thermal Enthalpies= -2126.222292
Sum of electronic and thermal Free Energies= -2126.323340

18-T_1 (CH_2Cl_2)

Zero-point correction= 0.385985 (Hartree/Particle)
Thermal correction to Energy= 0.419576
Thermal correction to Enthalpy= 0.420520
Thermal correction to Gibbs Free Energy= 0.315117
Sum of electronic and zero-point Energies= -2126.179041
Sum of electronic and thermal Energies= -2126.145451
Sum of electronic and thermal Enthalpies= -2126.144506
Sum of electronic and thermal Free Energies= -2126.249910

18-T_2 (CH_2Cl_2)

Zero-point correction= 0.383789 (Hartree/Particle)
Thermal correction to Energy= 0.417080
Thermal correction to Enthalpy= 0.418024
Thermal correction to Gibbs Free Energy= 0.314707
Sum of electronic and zero-point Energies= -2126.152196
Sum of electronic and thermal Energies= -2126.118906
Sum of electronic and thermal Enthalpies= -2126.117962
Sum of electronic and thermal Free Energies= -2126.221278

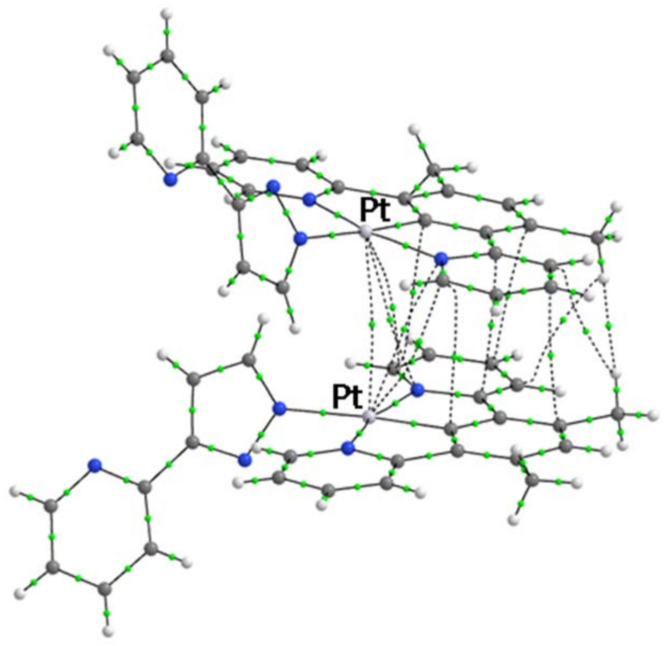

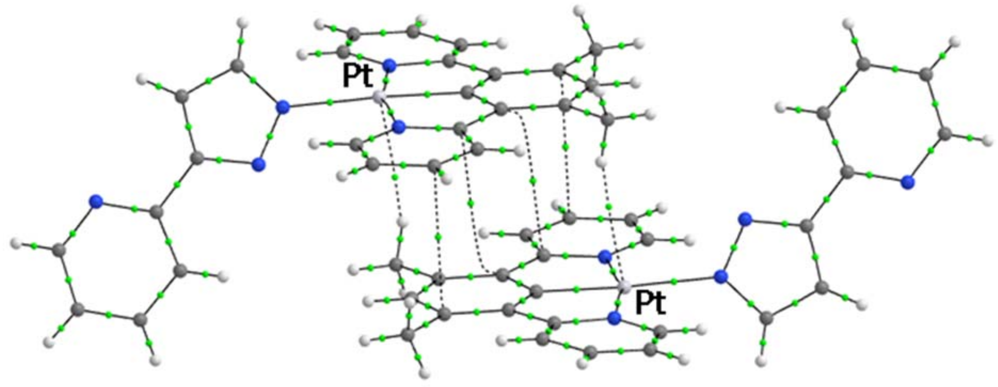

Figure S61. Molecular graph showing the bond paths and the bond critical points for the two π-π stacking moieties taken from the crystal data of complex **9**. Only bond paths between the Pt-tridentate ligands are depicted (intermolecular bond paths are shown as dashed lines). The bond critical points (BCP) are represented by the small green circles.

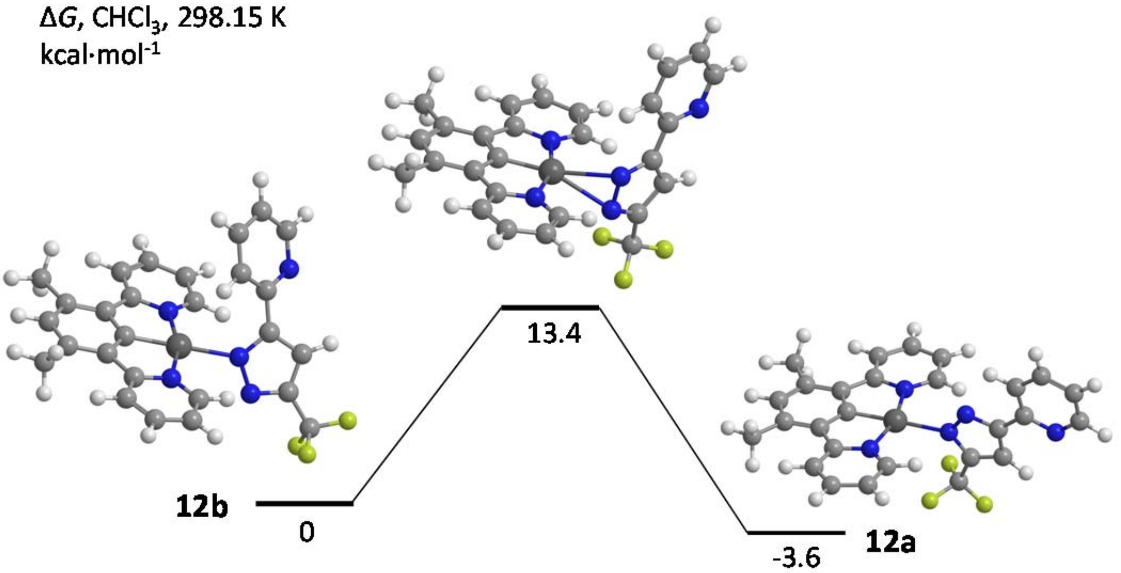

Figure S62. DFT-computed energy profile for the isomerization **12a-12b**. Relative free energies (ΔG at 298.15 K) are given in kcal·mol^{-1} and were computed at the B3LYP(G)//SDD(f)6-31Gg** level.

• UV-vis Spectra (Observed and Calculated) and Selected Excited Electronic Configurations for the Calculated Spectra of Complexes 7-10 and 16-18

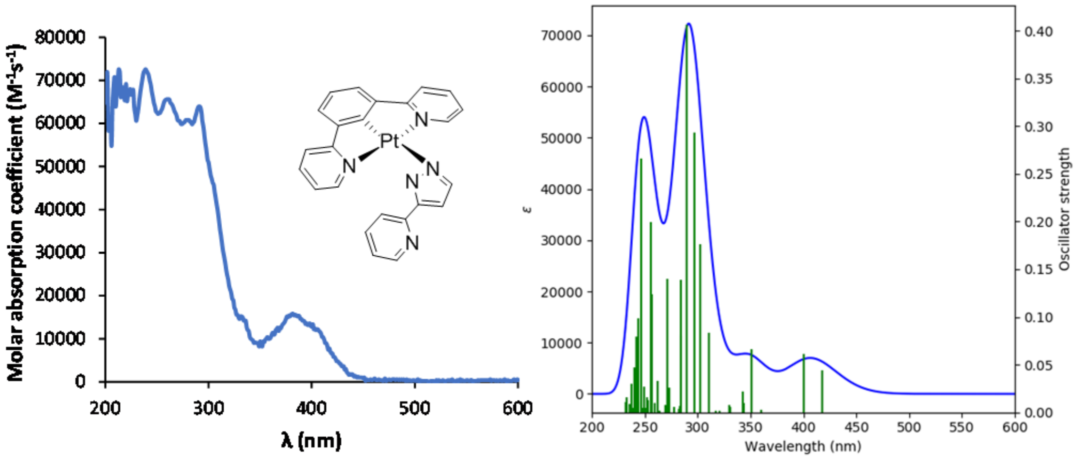

Figure S63. Observed UV-vis spectrum of complex **7** in dichloromethane (1.0 x 10^{-5} M) and calculated (B3LYP(GD3)//SDD(f)/6-31G**) in dichloromethane.

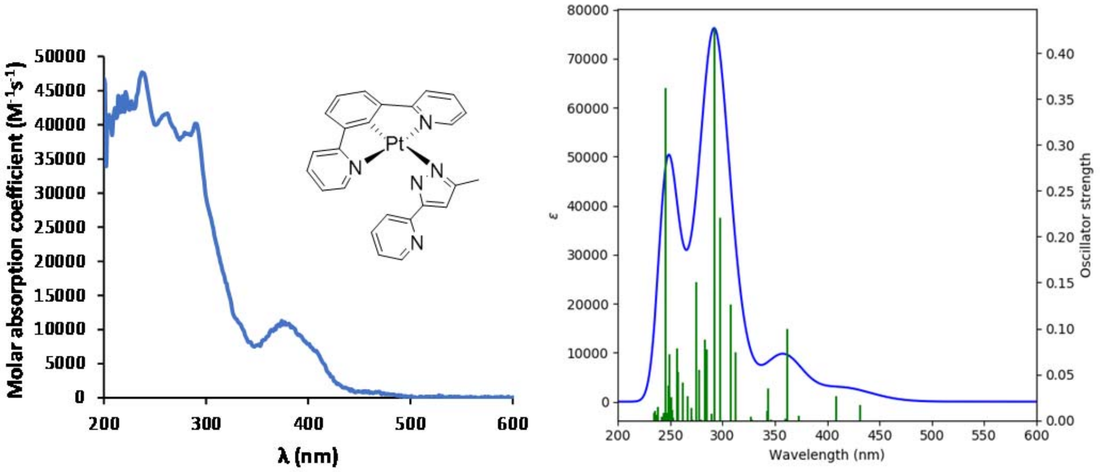

Figure S64. Observed UV-vis spectrum of complex **8** in dichloromethane (1.0 x 10^{-5} M) and calculated (B3LYP(GD3)//SDD(f)/6-31G**) in dichloromethane.

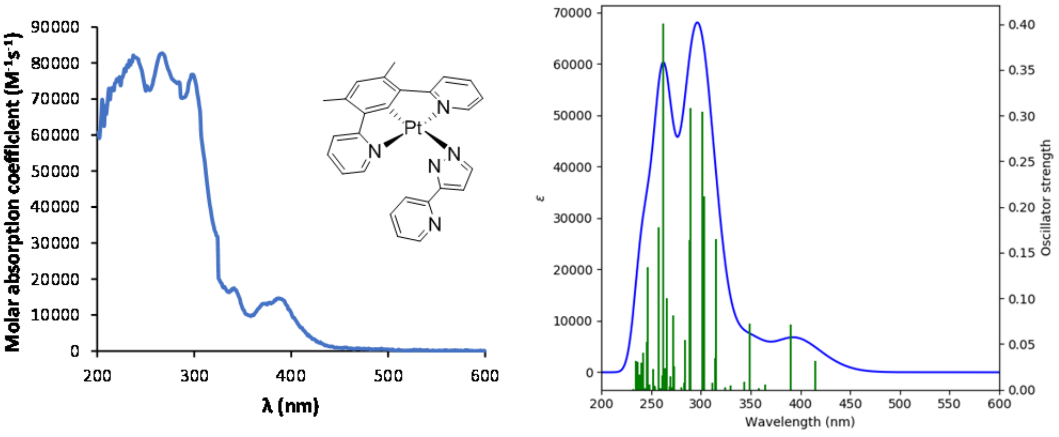

Figure S65. Observed UV-vis spectrum of complex **9** in dichloromethane (1.0 x 10^{-5} M) and calculated (B3LYP(GD3)//SDD(f)/6-31G**) in dichloromethane.

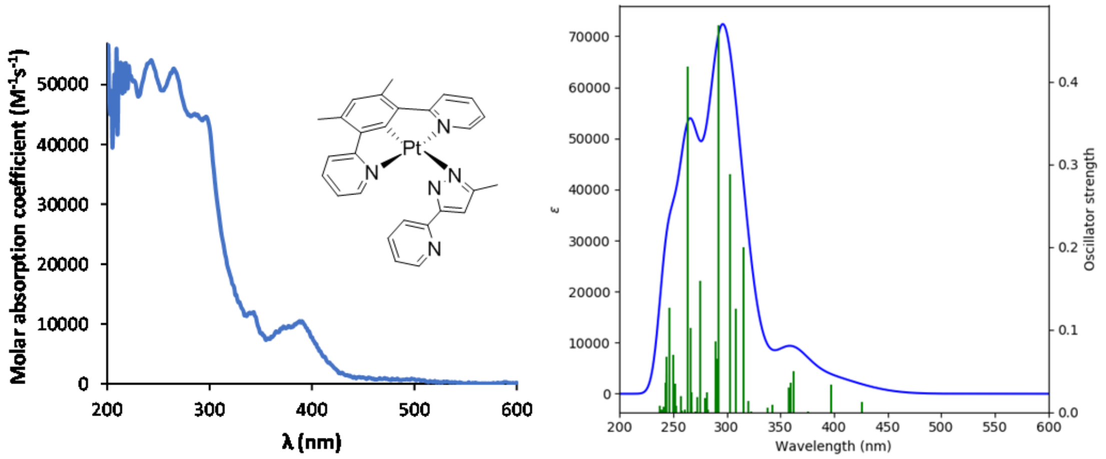

Figure S66. Observed UV-vis spectrum of complex **10** in dichloromethane (1.0 x 10^{-5} M) and calculated (B3LYP(GD3)//SDD(f)/6-31G**) in dichloromethane.

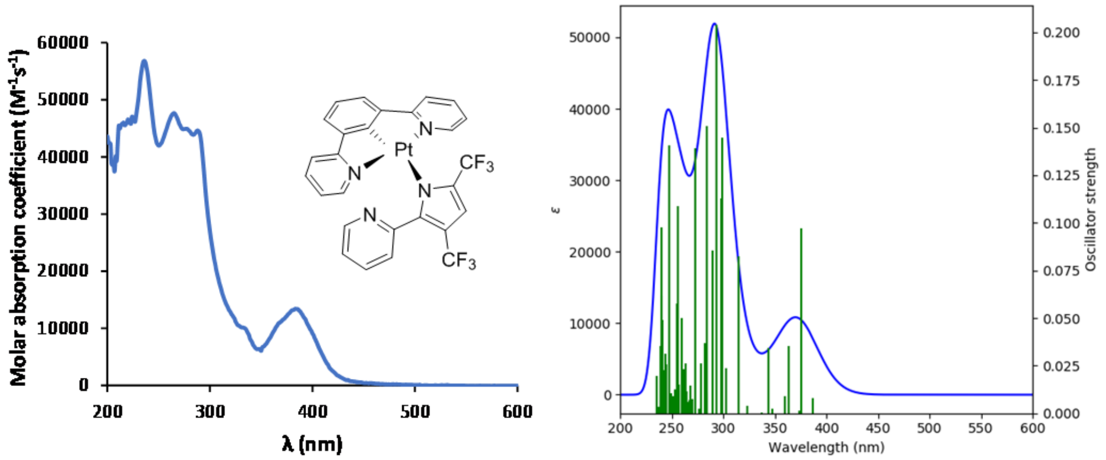

Figure S67. Observed UV-vis spectrum of complex **16** in dichloromethane (1.0 x 10^{-5} M) and calculated (B3LYP(GD3)//SDD(f)/6-31G**) in dichloromethane.

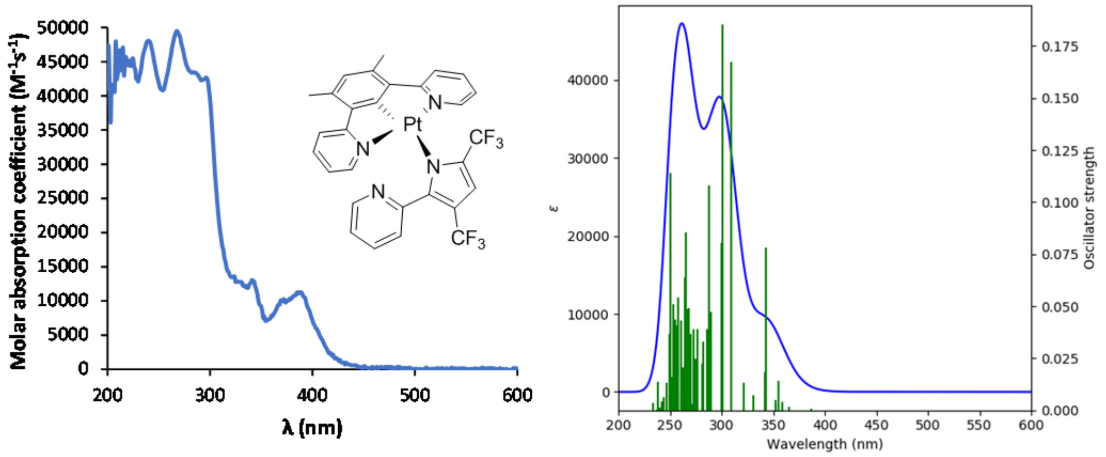

Figure S68. Observed UV-vis spectrum of complex **17** in dichloromethane (1.0 x 10^{-5} M) and calculated (B3LYP(GD3)//SDD(f)/6-31G**) in dichloromethane.

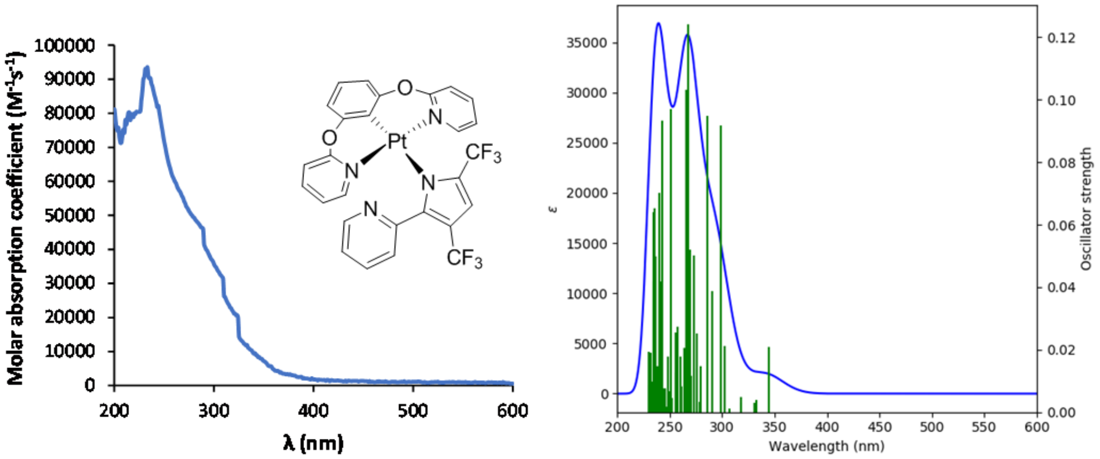

Figure S69. Observed UV-vis spectrum of complex **18** in dichloromethane (1.0 x 10^{-5} M) and calculated (B3LYP(GD3)//SDD(f)/6-31G**) in dichloromethane.

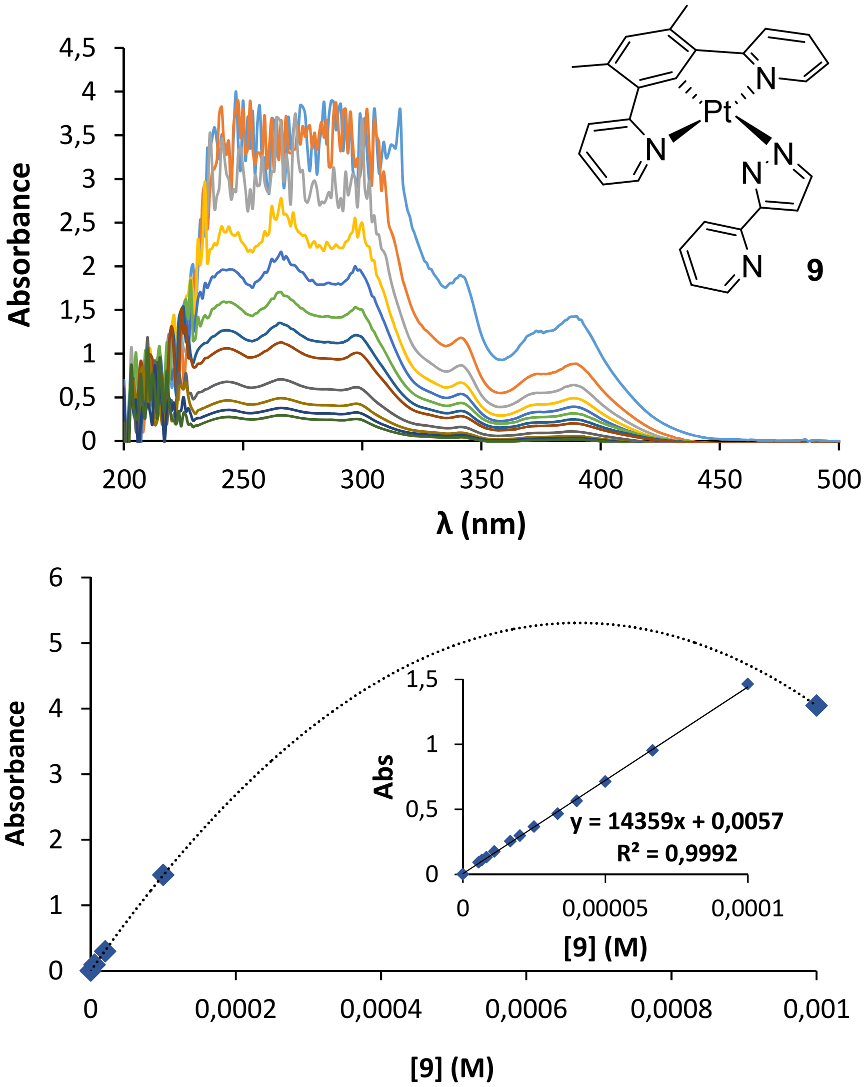

Figure S70. (top) Concentration dependent UV-vis spectra of **9**. (bottom) Beer-Lambert correlation of the absorption intensity at 390 nm.

Table S1. Selected excited electronic configurations for the calculated UV spectrum of complex 7 in CH_2Cl_2

λ (nm)	Osc. Strength	Symmetry	Major contributions	Minor contributions
471	0	Triplet-A	HOMO-3 → LUMO+1 (25%)<br>HOMO-1 → LUMO (22%)<br>HOMO → LUMO (34%)	HOMO-5 → LUMO+1 (3%)<br>HOMO-2 → LUMO (4%)
400	0.0613	Singlet-A	HOMO → LUMO+1 (93%)	HOMO-1 → LUMO+1 (3%)
351	0.0662	Singlet-A	HOMO-1 → LUMO+1 (78%)	HOMO-5 → LUMO (3%)<br>HOMO-3 → LUMO (9%)<br>HOMO-2 → LUMO+1 (4%)<br>HOMO → LUMO+1 (4%)
302	0.1762	Singlet-A	HOMO → LUMO+3 (91%)	HOMO-3 → LUMO+1 (4%)
289	0.4064	Singlet-A	HOMO → LUMO+4 (89%)	HOMO → LUMO+5 (4%)
256	0.1992	Singlet-A	HOMO-3 → LUMO+3 (63%)	HOMO-10 → LUMO+1 (4%)<br>HOMO-5 → LUMO (2%)<br>HOMO-5 → LUMO+3 (4%)<br>HOMO-3 → LUMO+4 (3%)
247	0.2664	Singlet-A	HOMO-9 → LUMO+1 (49%)<br>HOMO-3 → LUMO+3 (15%)<br>HOMO → LUMO+8 (10%)	HOMO-10 → LUMO+1 (7%)<br>HOMO-8 → LUMO+1 (4%)<br>HOMO → LUMO+6 (7%)

Table S2. Selected excited electronic configurations for the calculated UV spectrum of complex 8 in CH_2Cl_2

λ (nm)	Osc. Strength	Symmetry	Major contributions	Minor contributions
470	0	Triplet-A	HOMO-3 → LUMO+1 (26%)<br>HOMO-2 → LUMO (25%)<br>HOMO → LUMO (25%)	HOMO-5 → LUMO+1 (3%)
409	0.0266	Singlet-A	HOMO → LUMO+1 (96%)	-
313		Singlet-A	HOMO-5 → LUMO+1 (15%)<br>HOMO-3 → LUMO+1 (65%)<br>HOMO → LUMO+3 (11%)	-
298	0.2209	Singlet-A	HOMO-5 → LUMO (65%)<br>HOMO-2 → LUMO+2 (13%)<br>HOMO-1 → LUMO+2 (11%)	HOMO-3 → LUMO-3 (2%)
293	0.4262	Singlet-A	HOMO-1 → LUMO+2 (20%)<br>HOMO → LUMO+4 (61%)	HOMO-5 → LUMO (8%)<br>HOMO → LUMO+5 (4%)
262	0.0407	Singlet-A	HOMO-3 → LUMO+2 (61%)	HOMO-4 → LUMO+6 (4%)<br>HOMO-2 → LUMO+3 (7%)
246	0.3616	Singlet-A	HOMO-10 → LUMO+1 (14%)<br>HOMO-9 → LUMO+1 (45%)<br>HOMO-3 → LUMO+3 (13%)<br>HOMO → LUMO+8 (14%)	HOMO-2 → LUMO+6 (2%)

Table S3. Selected excited electronic configurations for the calculated UV spectrum of complex 9 in CH_2Cl_2

λ (nm)	Osc. Strength	Symmetry	Major contributions	Minor contributions
475	0	Triplet-A	HOMO-2 → LUMO+1 (30%)<br>HOMO-1 → LUMO (26%)<br>HOMO → LUMO (28%)	HOMO-3 → LUMO+1 (3%)
391	0.0716	Singlet-A	HOMO → LUMO+1 (92%)	HOMO-1 → LUMO+1 (5%)
349	0.0725	Singlet-A	HOMO-1 → LUMO+1 (87%)	HOMO-2 → LUMO (3%)<br>HOMO → LUMO+1 (5%)
303	0.2118	Singlet-A	HOMO → LUMO+3 (87%)	HOMO-5 → LUMO (6%)<br>HOMO-2 → LUMO+1 (4%)
289	0.3078	Singlet-A	HOMO → LUMO+4 (86%)	HOMO-5 → LUMO+1 (3%)<br>HOMO → LUMO+5 (4%)
258	0.1783	Singlet-A	HOMO-1 → LUMO+4 (66%)	HOMO-4 → LUMO+3 (2%)<br>HOMO-1 → LUMO+3 (5%)<br>HOMO → LUMO+5 (5%)
246	0.1337	Singlet-A	HOMO-9 → LUMO+1 (57%)	HOMO-10 → LUMO+1 (5%)<br>HOMO-8 → LUMO+1 (4%)<br>HOMO-2 → LUMO+3 (7%)<br>HOMO-2 → LUMO+4 (9%)

Table S4. Selected excited electronic configurations for the calculated UV spectrum of complex 10 in CH_2Cl_2

λ (nm)	Osc. Strength	Symmetry	Major contributions	Minor contributions
474	0	Triplet-A	HOMO-3 → LUMO+1 (34%)<br>HOMO-1 → LUMO (25%)<br>HOMO → LUMO (17%)	-
397	0.0329	Singlet-A	HOMO → LUMO+1 (95%)	HOMO-1 → LUMO+1 (2%)
308	0.1253	Singlet-A	HOMO → LUMO+3 (93%)	HOMO-3 → LUMO+1 (4%)
303	0.2879	Singlet-A	HOMO-5 → LUMO (79%)	HOMO-3 → LUMO+3 (4%)<br>HOMO-2 → LUMO+2 (3%)<br>HOMO-1 → LUMO+2 (5%)
293	0.4687	Singlet-A	HOMO → LUMO+4 (84%)	HOMO-1 → LUMO+2 (3%)<br>HOMO → LUMO+5 (6%)
264	0.4185	Singlet-A	HOMO-3 → LUMO+3 (78%)	HOMO-9 → LUMO+1 (3%)<br>HOMO-8 → LUMO+1 (2%)
247	0.1264	Singlet-A	HOMO-9 → LUMO+1 (61%)<br>HOMO → LUMO+8 (10%)	HOMO-5 → LUMO (4%)<br>HOMO-5 → LUMO-3 (3%)

Table S5. Selected excited electronic configurations for the calculated UV spectrum of complex 16 in CH_2Cl_2

λ (nm)	Osc. Strength	Symmetry	Major contributions	Minor contributions
464	0	Triplet-A	HOMO → LUMO+1 (34%)<br>HOMO-4 → LUMO+1 (19%)<br>HOMO-1 → LUMO (41%)	HOMO-5 → LUMO+1 (5%)<br>HOMO-4 → LUMO (3%)<br>HOMO-1 → LUMO+1 (3%)<br>HOMO → LUMO (2%)
375	0.0973	Singlet-A	HOMO-1 → LUMO+1 (52%)	HOMO-1 → LUMO (7%)
343	0.0343	Singlet-A	HOMO-4 → LUMO (32%)<br>HOMO-3 → LUMO (50%)<br>HOMO-2 → LUMO+1 (10%)	HOMO-1 → LUMO+1 (2%)
315	0.0824	Singlet-A	HOMO-5 → LUMO+1 (16%)<br>HOMO-4 → LUMO+1 (54%)<br>HOMO-3 → LUMO+1 (17%)	-
299	0.1448	Singlet-A	HOMO-5 → LUMO (11%)<br>HOMO → LUMO+3 (59%)	HOMO-2 → LUMO+3 (2%)<br>HOMO-1 → LUMO+2 (8%)<br>HOMO-1 → LUMO+3 (7%)<br>HOMO → LUMO+2 (6%)
272	0.1391	Singlet-A	HOMO → LUMO+5 (73%)	HOMO-2 → LUMO+3 (8%)<br>HOMO-2 → LUMO+4 (3%)<br>HOMO → LUMO+3 (3%)
256	0.1088	Singlet-A	HOMO-3 → LUMO+4 (73%)	HOMO-8 → LUMO+1 (6%)<br>HOMO-6 → LUMO (3%)<br>HOMO-4 → LUMO+3 (3%)

Table S6. Selected excited electronic configurations for the calculated UV spectrum of complex 17 in CH_2Cl_2

λ (nm)	Osc. Strength	Symmetry	Major contributions	Minor contributions
452	0	Triplet-A	HOMO-3 → LUMO+1 (41%)<br>HOMO-2 → LUMO (20%)	HOMO-3 → LUMO (5%)<br>HOMO-1 → LUMO (5%)<br>HOMO-1 → LUMO+1 (5%)<br>HOMO → LUMO (6%)
355	0.0142	Singlet-A	HOMO → LUMO+1 (70%)	HOMO-4 → LUMO (7%)<br>HOMO-3 → LUMO (3%)<br>HOMO-2 → LUMO+1 (2%)<br>HOMO-1 → LUMO (3%)
343	0.0782	Singlet-A	HOMO-4 → LUMO (15%)<br>HOMO-2 → LUMO+1 (42%)<br>HOMO-1 → LUMO+1 (20%)	HOMO-3 → LUMO (2%)<br>HOMO-2 → LUMO (8%)<br>HOMO-1 → LUMO (5%)<br>HOMO → LUMO (2%)
309	0.1674	Singlet-A	HOMO-3 → LUMO+1 (80%)	HOMO-5 → LUMO (3%)<br>HOMO-5 → LUMO+1 (8%)
301	0.1855	Singlet-A	HOMO-5 → LUMO (57%)<br>HOMO → LUMO+2 (28%)	HOMO-2 → LUMO+2 (3%)
288	0.1081	Singlet-A	HOMO-5 → LUMO+1 (37%)<br>HOMO-3 → LUMO+2 (11%)<br>HOMO-1 → LUMO+2 (32%)	HOMO-3 → LUMO+1 (4%)<br>HOMO → LUMO+2 (4%)<br>HOMO → LUMO+3 (5%)
255	0.0434	Singlet-A	HOMO-1 → LUMO+5 (63%)	HOMO-4 → LUMO+6 (4%)<br>HOMO-4 → LUMO+7 (5%)<br>HOMO-3 → LUMO+4 (6%)<br>HOMO-2 → LUMO+5 (4%)

Table S7. Selected excited electronic configurations for the calculated UV spectrum of complex 18 in CH_2Cl_2

**λ (nm)**	**Osc. Strength**	**Symmetry**	**Major contributions**	**Minor contributions**
371	0	Triplet-A	HOMO-1 → LUMO+2 (11%)<br>HOMO → LUMO+2 (56%)	HOMO-11 → LUMO+2 (4%)<br>HOMO-8 → LUMO+5 (3%)<br>HOMO-1 → LUMO+5 (2%)<br>HOMO → LUMO+5 (7%)
345	0.0209	Singlet-A	HOMO → LUMO (85%)	-
302	0.0212	Singlet-A	HOMO-3 → LUMO (88%)	HOMO-5 → LUMO (2%)<br>HOMO-2 → LUMO (3%)<br>HOMO-1 → LUMO+1 (2%)
299	0.0916	Singlet-A	HOMO → LUMO+2 (87%)	HOMO-2 → LUMO+2 (4%)
290	0.0386	Singlet-A	HOMO-3 → LUMO+1 (81%)	HOMO-2 → LUMO+1 (5%)
286	0.0949	Singlet-A	HOMO-1 → LUMO+2 (85%)	HOMO-2 → LUMO+2 (4%)<br>HOMO → LUMO+2 (2%)<br>HOMO → LUMO+4 (3%)
242	0.0933	Singlet-A	HOMO-7 → LUMO (20%)<br>HOMO-6 → LUMO+1 (12%)<br>HOMO-3 → LUMO+3 (10%)<br>HOMO → LUMO+7 (25%)	HOMO-8 → LUMO (2%)<br>HOMO-7 → LUMO+2 (2%)<br>HOMO-6 → LUMO (7%)<br>HOMO-2 → LUMO+7 (2%)

• Theoretical Analysis of Molecular Orbitals of Complexes 7-10 and 16-18.

Energies and population analysis (%) of molecular orbitals are given in Tables S8-S14 whereas Figures S71–S77 collect the molecular orbitals.

Table S8. Composition of molecular orbitals of 7

MO	eV	% (Pt)	% (NCN)	% (NN)
L+9	0.86	74	24	2
L+8	0.42	70	27	3
L+7	0.29	21	78	1
L+6	-0.04	83	16	1
L+5	-0.22	1	1	98
L+4	-0.57	1	11	88
L+3	-0.70	3	86	11
L+2	-0.93	3	97	0
L+1	-1.73	2	98	0
LUMO	-1.81	10	89	1
HOMO	-5.38	14	12	74
H-1	-5.91	20	45	35
H-2	-6.08	9	16	75
H-3	-6.23	23	76	1
H-4	-6.42	88	4	8
H-5	-6.70	47	51	2
H-6	-6.77	1	0	99
H-7	-6.91	5	16	80
H-8	-7.05	43	14	43
H-9	-7.37	32	43	25

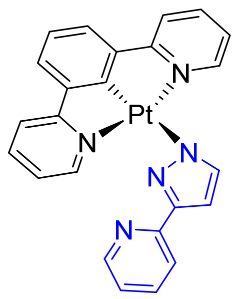

7

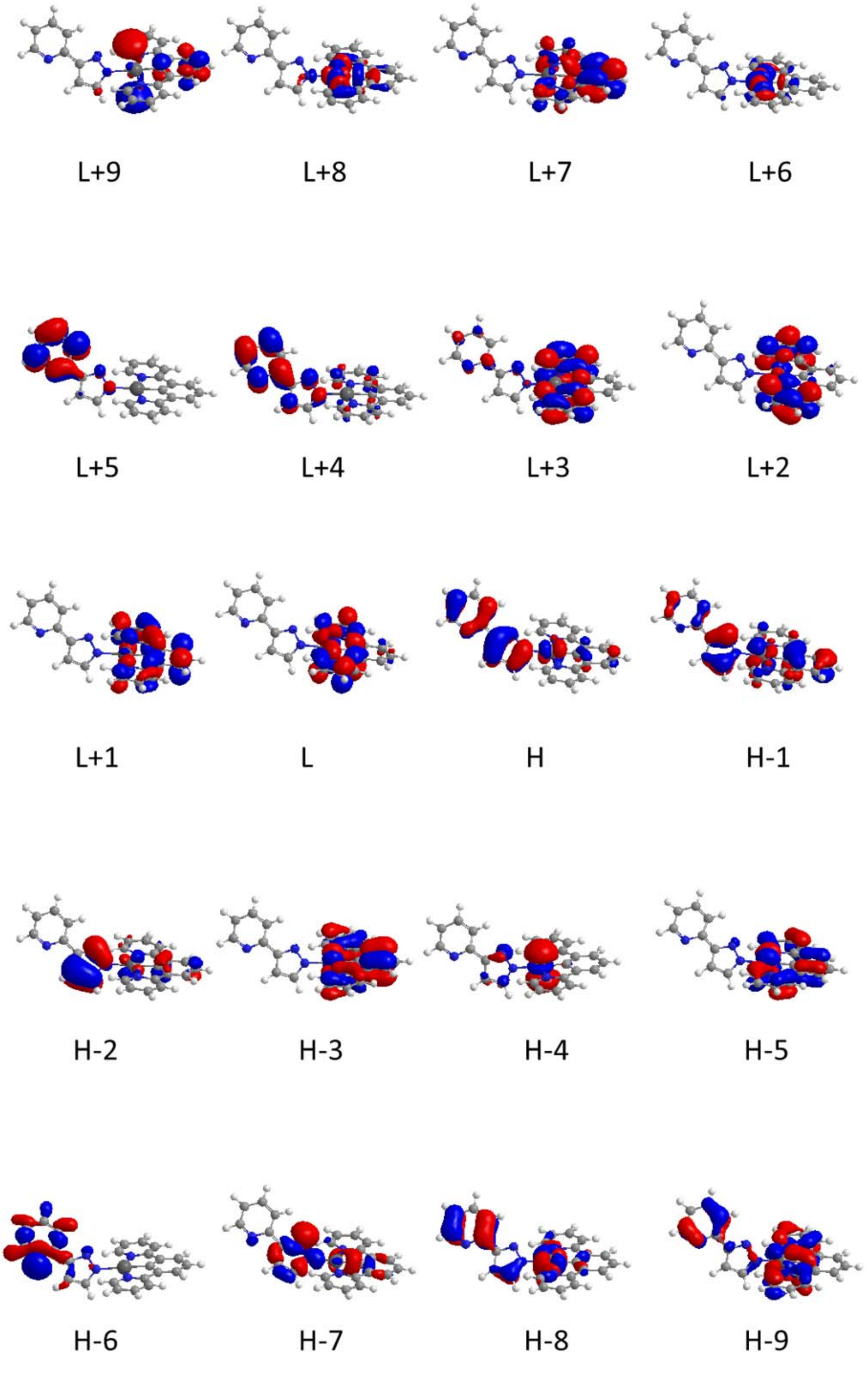

Figure S71. Molecular orbitals calculated for the optimized geometry of the ground state of complex **7** (isovalue 0.03 a.u.).

Table S9. Composition of molecular orbitals of 8

MO	eV	% (Pt)	% (NCN)	% (NN)
L+9	0.94	66	32	2
L+8	0.59	62	35	4
L+7	0.38	30	69	1
L+6	0.01	89	10	1
L+5	-0.22	1	1	99
L+4	-0.56	1	12	88
L+3	-0.69	3	86	11
L+2	-0.84	2	97	1
L+1	-1.64	2	98	0
LUMO	-1.77	11	89	1
HOMO	-5.37	14	13	72
H-1	-5.83	20	54	26
H-2	-6.02	15	77	8
H-3	-6.05	9	15	76
H-4	-6.38	87	4	9
H-5	-6.61	54	46	1
H-6	-6.76	0	0	99
H-7	-6.87	4	17	79
H-8	-7.03	43	13	44
H-9	-7.34	38	46	15

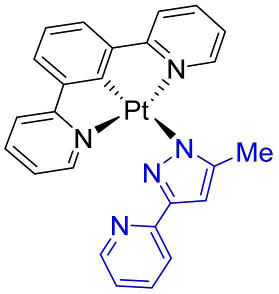

8

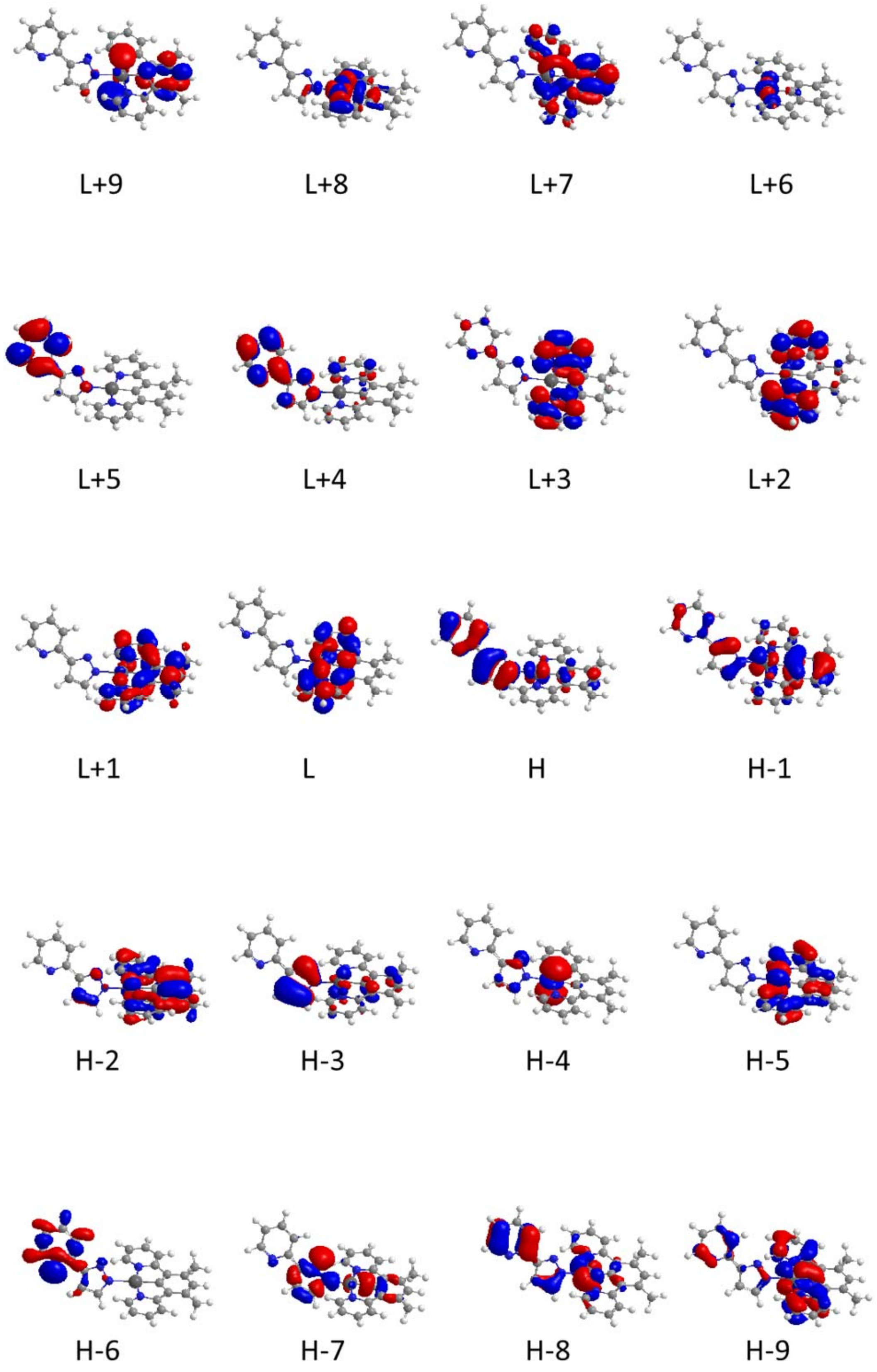

Figure S72. Molecular orbitals calculated for the optimized geometry of the ground state of complex **8** (isovalue 0.03 a.u.).

Table S10. Composition of molecular orbitals of 9

MO	eV	% (Pt)	% (NCN)	% (NN)
L+9	0.87	72	25	3
L+8	0.46	67	30	3
L+7	0.29	20	78	2
L+6	-0.04	85	14	1
L+5	-0.19	1	1	99
L+4	-0.53	1	5	95
L+3	-0.70	4	92	5
L+2	-0.93	2	96	1
L+1	-1.73	2	98	0
LUMO	-1.81	10	89	1
HOMO	-5.29	10	5	85
H-1	-5.77	9	15	76
H-2	-5.90	22	51	28
H-3	-6.23	26	74	1
H-4	-6.35	90	4	6
H-5	-6.69	44	52	4
H-6	-6.72	2	1	97
H-7	-6.81	7	15	78
H-8	-6.97	37	11	52
H-9	-7.39	29	39	32

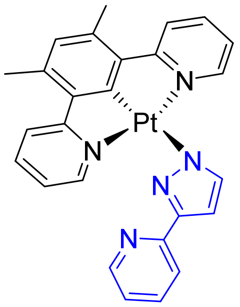

9

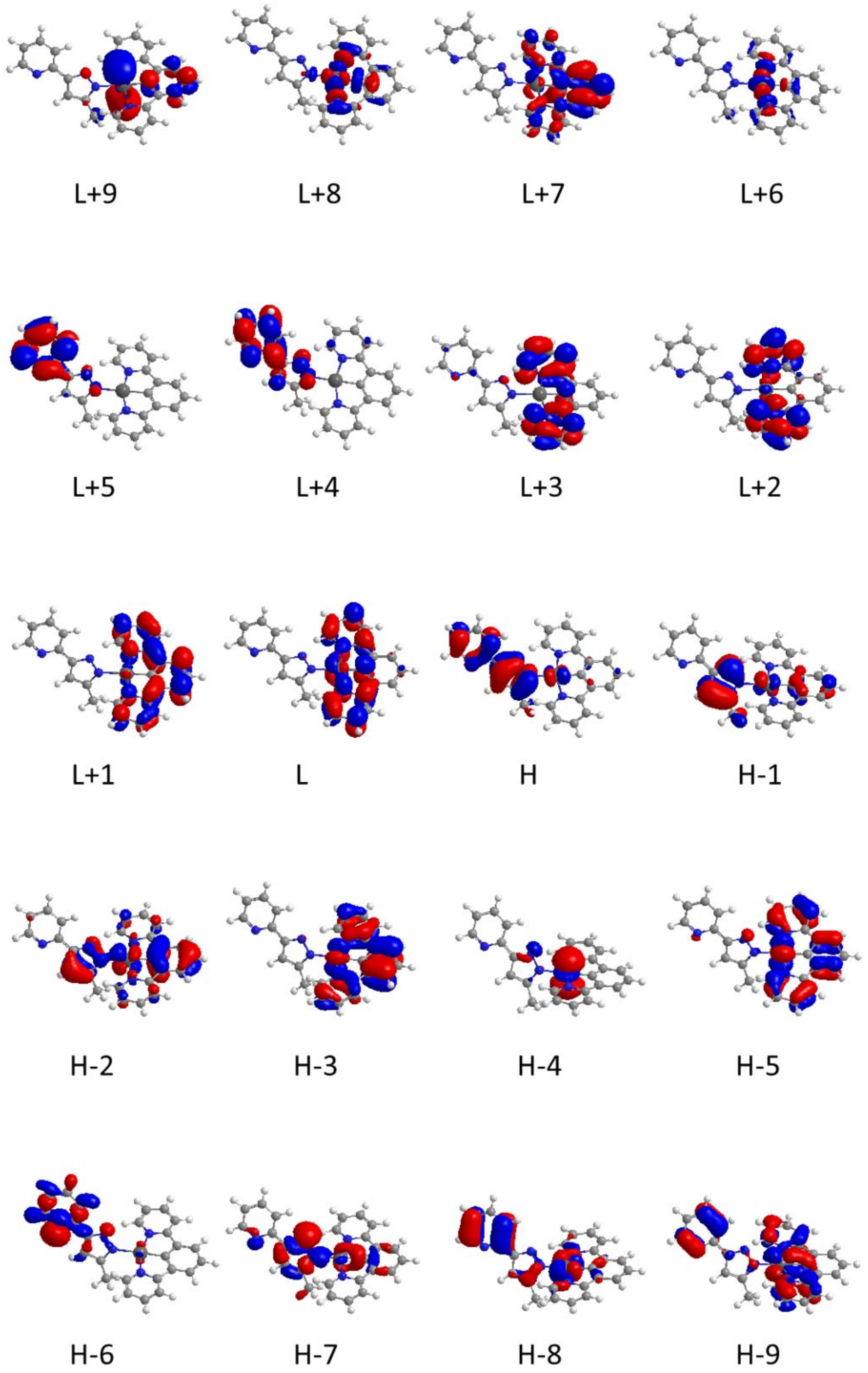

Figure S73. Molecular orbitals calculated for the optimized geometry of the ground state of complex **9** (isovalue 0.03 a.u.).

Table S11. Composition of molecular orbitals of 10

MO	eV	% (Pt)	% (NCN)	% (NN)
L+9	0.96	64	33	2
L+8	0.63	61	36	3
L+7	0.38	28	70	2
L+6	-0.01	90	9	1
L+5	-0.18	1	0	99
L+4	-0.52	1	5	94
L+3	-0.69	4	92	5
L+2	-0.84	2	96	2
L+1	-1.63	2	98	0
LUMO	-1.77	10	89	1
HOMO	-5.29	10	6	85
H-1	-5.73	17	40	43
H-2	-5.84	11	28	61
H-3	-6.02	17	83	0
H-4	-6.31	91	4	5
H-5	-6.59	52	47	1
H-6	-6.72	1	0	99
H-7	-6.77	5	16	78
H-8	-6.96	38	11	51
H-9	-7.35	38	48	14

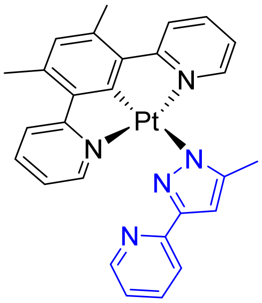

10

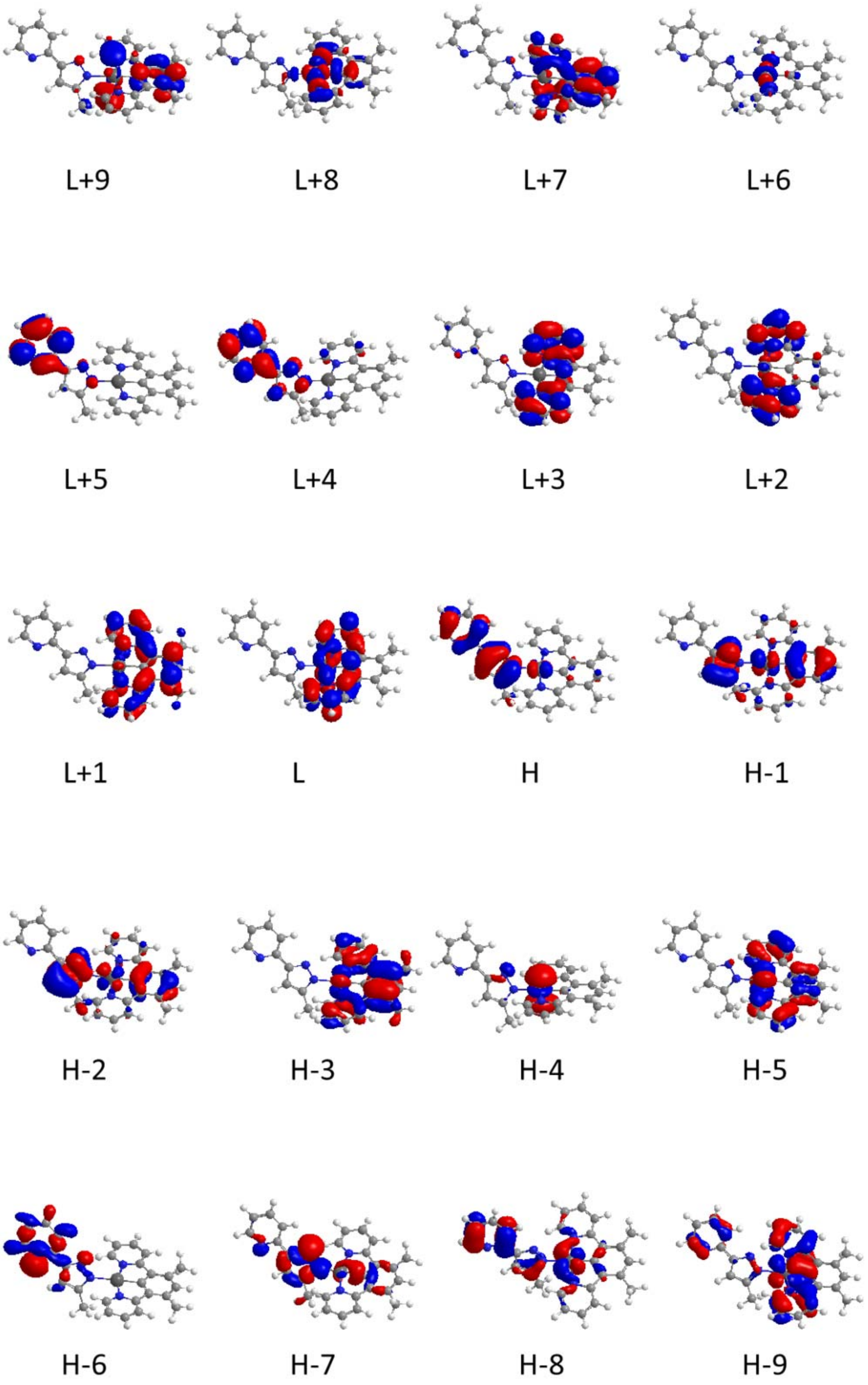

Figure S74. Molecular orbitals calculated for the optimized geometry of the ground state of complex **10** (isovalue 0.03 a.u.)

Table S12. Composition of molecular orbitals of 16

MO	eV	% (Pt)	% (NCN)	% (NN)
L+9	0.93	17	7	76
L+8	0.44	66	30	4
L+7	0.37	10	89	1
L+6	0.01	83	13	4
L+5	-0.41	5	1	94
L+4	-0.66	3	93	5
L+3	-0.82	2	13	85
L+2	-0.90	2	90	8
L+1	-1.71	2	98	0
LUMO	-1.76	8	90	2
HOMO	-5.61	5	3	92
H-1	-5.76	32	57	11
H-2	-6.07	69	11	20
H-3	-6.18	32	32	36
H-4	-6.21	27	45	28
H-5	-6.65	42	57	1
H-6	-7.09	18	11	72
H-7	-7.29	1	3	96
H-8	-7.40	34	54	12
H-9	-7.52	37	27	35

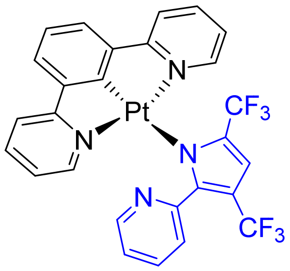

16

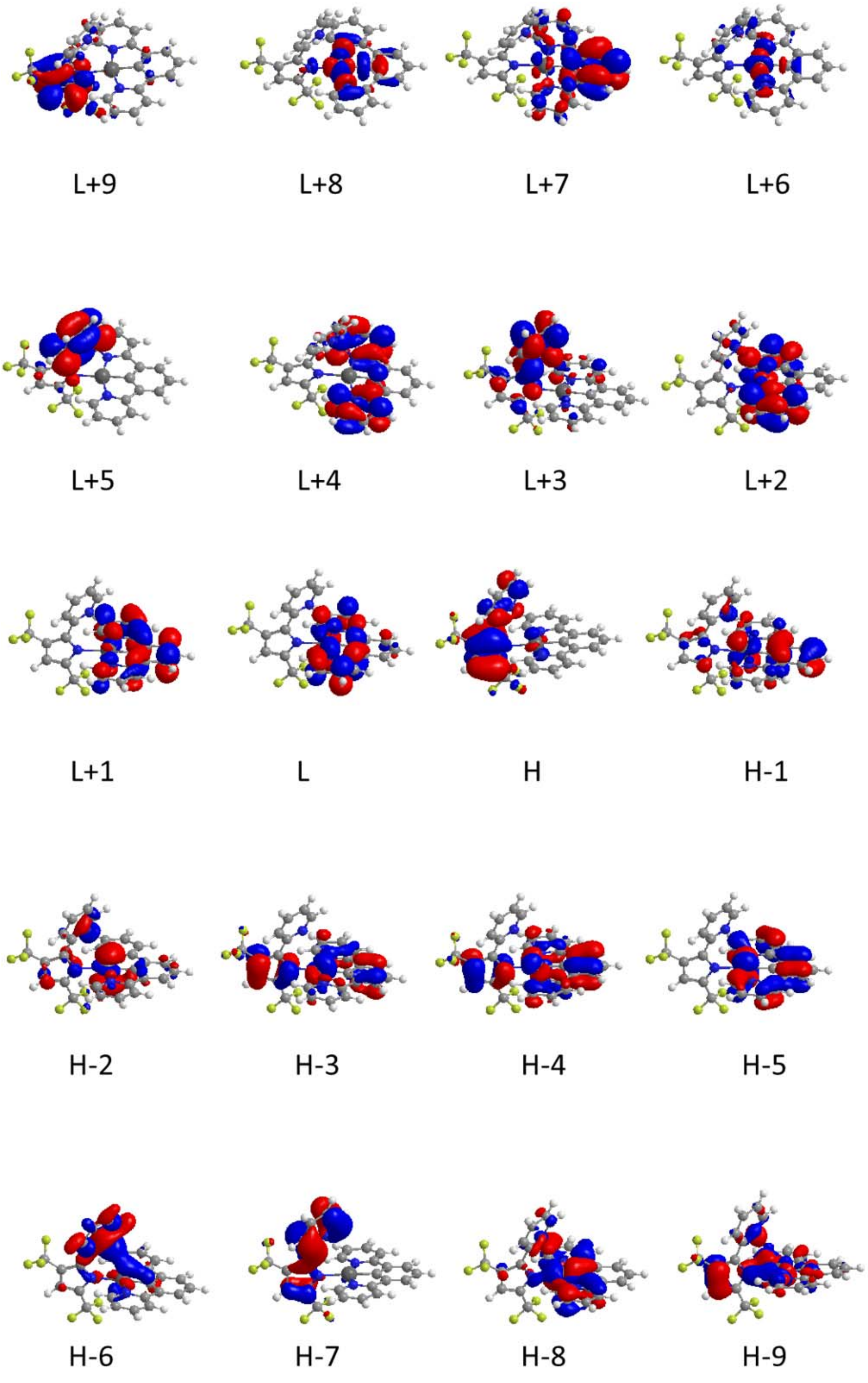

Figure S75. Molecular orbitals calculated for the optimized geometry of the ground state of complex **16** (isovalue 0.03 a.u.)

Table S13. Composition of molecular orbitals of 17

MO	eV	% (Pt)	% (NCN)	% (NN)
L+9	0.98	26	4	71
L+8	0.56	23	76	1
L+7	0.46	55	32	13
L+6	0.04	85	12	3
L+5	-0.27	6	1	93
L+4	-0.53	3	80	17
L+3	-0.63	2	27	71
L+2	-0.81	2	86	11
L+1	-1.48	2	97	0
LUMO	-1.66	9	90	1
HOMO	-5.64	22	12	66
H-1	-5.80	26	18	56
H-2	-5.84	24	59	17
H-3	-5.95	17	71	12
H-4	-6.13	86	5	10
H-5	-6.50	50	48	2
H-6	-6.96	6	8	86
H-7	-7.11	43	38	19
H-8	-7.18	12	12	76
H-9	-7.24	16	51	32

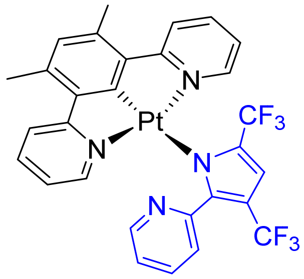

17

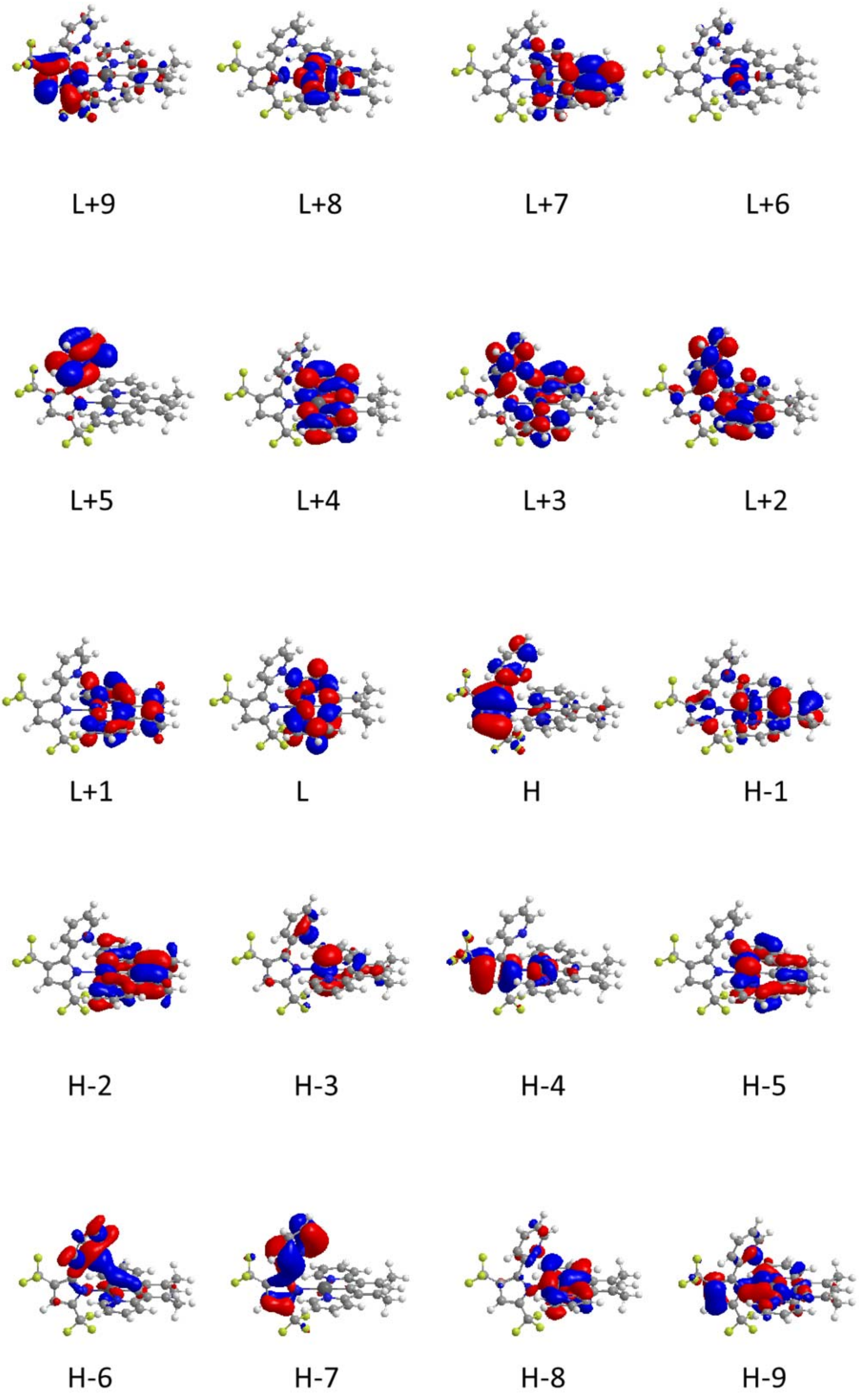

Figure S76. Molecular orbitals calculated for the optimized geometry of the ground state of complex **17** (isovalue 0.03 a.u.)

Table S14. Composition of molecular orbitals of 18

MO	eV	% (Pt)	% (NCN)	% (NN)
L+9	0.33	69	26	5
L+8	0.24	18	78	4
L+7	0.01	21	76	3
L+6	-0.07	59	37	4
L+5	-0.43	4	30	66
L+4	-0.50	2	69	29
L+3	-0.53	2	96	2
L+2	-0.86	2	4	95
L+1	-1.25	4	95	1
LUMO	-1.41	4	95	1
HOMO	-5.69	16	15	69
H-1	-5.82	24	37	40
H-2	-6.12	72	10	18
H-3	-6.16	13	84	3
H-4	-6.54	21	14	66
H-5	-6.73	65	34	1
H-6	-7.05	10	67	23
H-7	-7.22	9	28	63
H-8	-7.28	1	27	72
H-9	-7.44	58	30	12

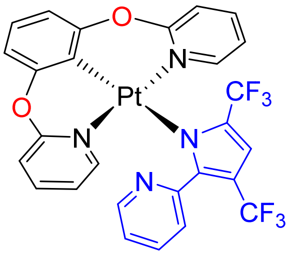

18

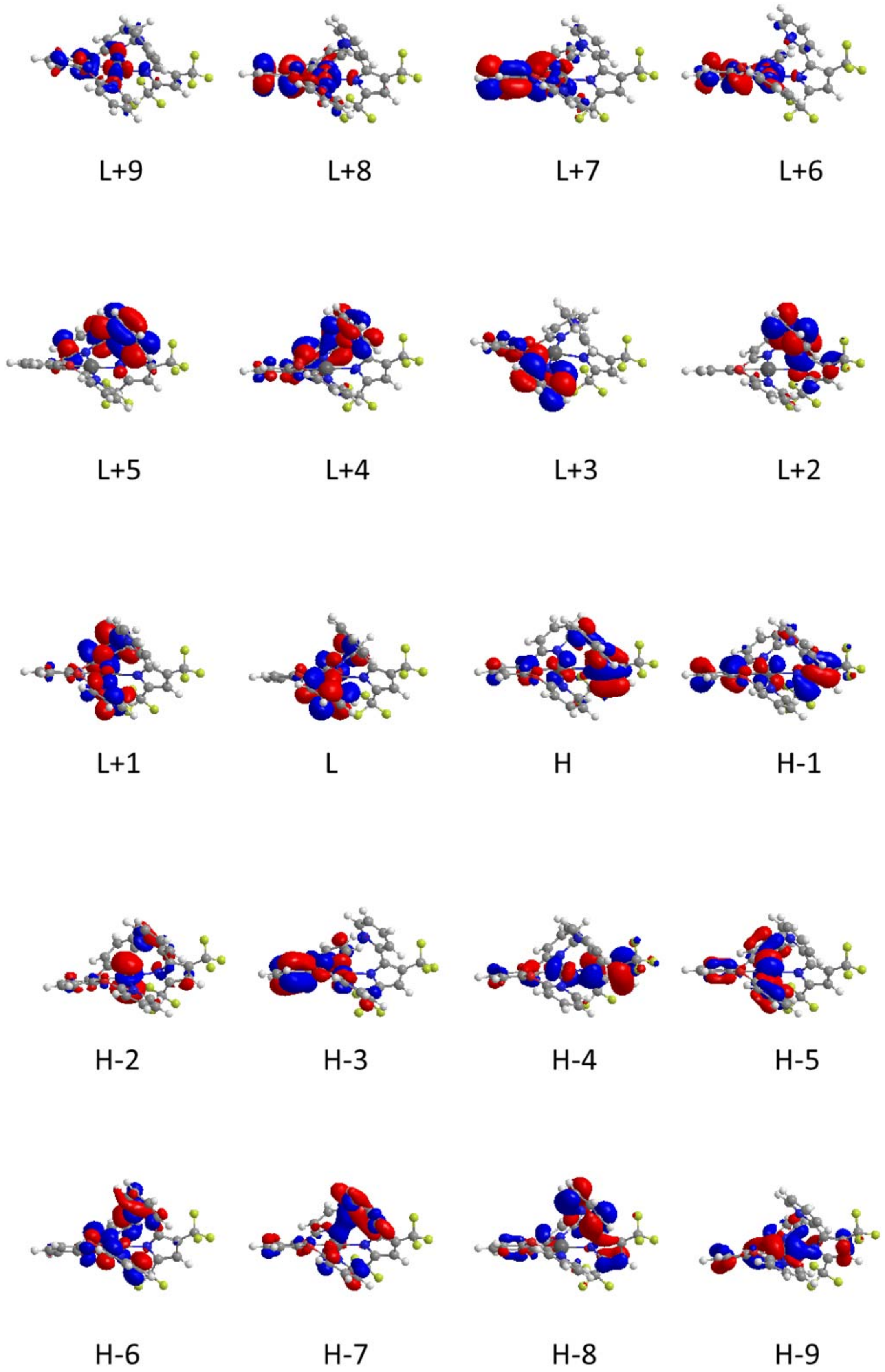

Figure S77. Molecular orbitals calculated for the optimized geometry of the ground state of complex **18** (isovalue 0.03 a.u.)

• **Cyclic Voltammograms**

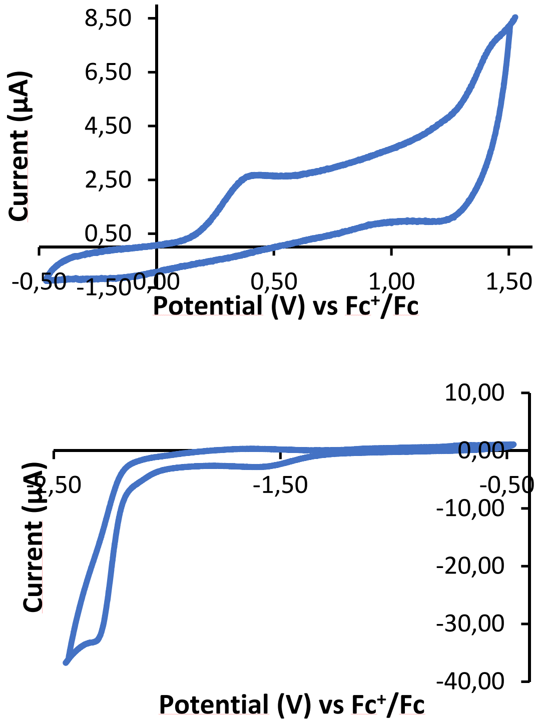

Figure S78. Cyclic voltammogram of complex **7** in dichloromethane 10^{-3} M solution with $[Bu_4N]PF_6$ as supporting electrolyte (0.1 M) at a scan rate of 100 mV s^{-1}. The potentials were referenced to the Fc/Fc^+ couple.

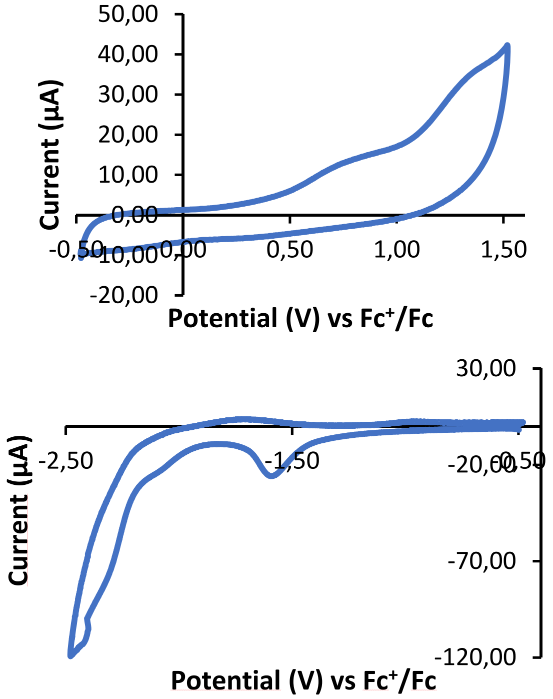

Figure S79. Cyclic voltammogram of complex **8** in dichloromethane 10^{-3} M solution with $[Bu_4N]PF_6$ as supporting electrolyte (0.1 M) at a scan rate of 100 mV s^{-1}. The potentials were referenced to the Fc/Fc^+ couple.

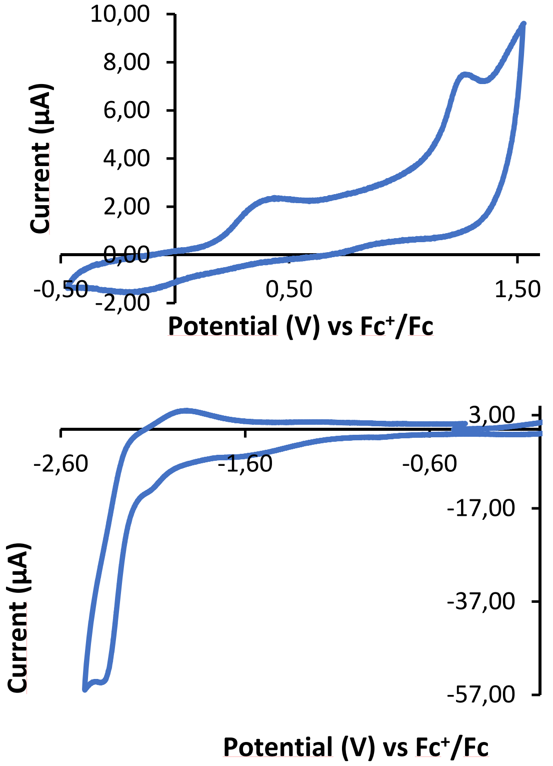

Figure S80. Cyclic voltammogram of complex **9** in dichloromethane 10^{-3} M solution with $[Bu_4N]PF_6$ as supporting electrolyte (0.1 M) at a scan rate of 100 mV s^{-1}. The potentials were referenced to the Fc/Fc^+ couple.

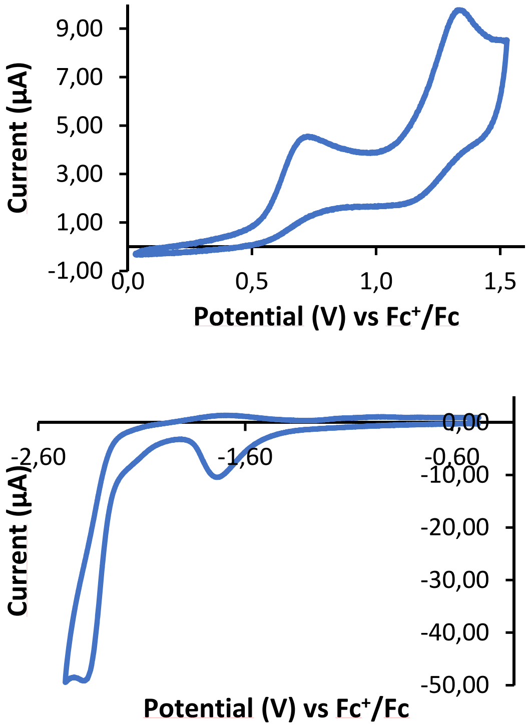

Figure S81. Cyclic voltammogram of complex **10** in dichloromethane 10^{-3} M solution with $[Bu_4N]PF_6$ as supporting electrolyte (0.1 M) at a scan rate of 100 mV s^{-1}. The potentials were referenced to the Fc/Fc^+ couple.

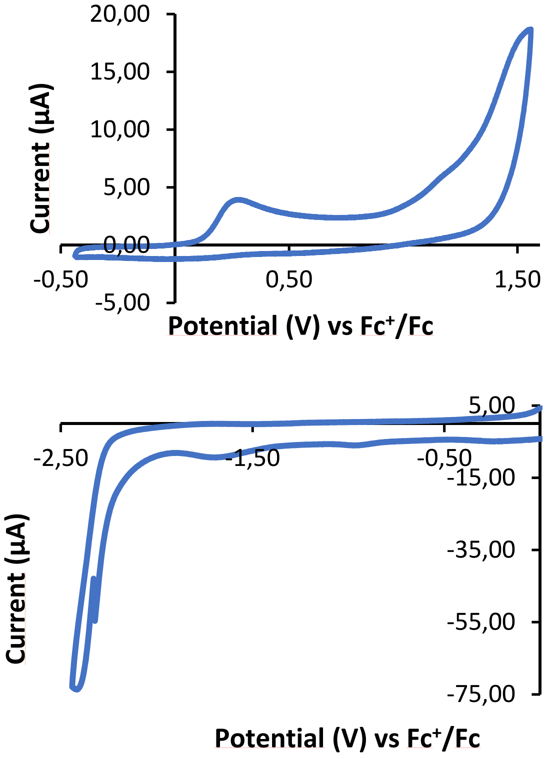

Figure S82. Cyclic voltammogram of complex **16** in dichloromethane 10^{-3} M solution with $[Bu_4N]PF_6$ as supporting electrolyte (0.1 M) at a scan rate of 100 mV s^{-1}. The potentials were referenced to the Fc/Fc^{+} couple.

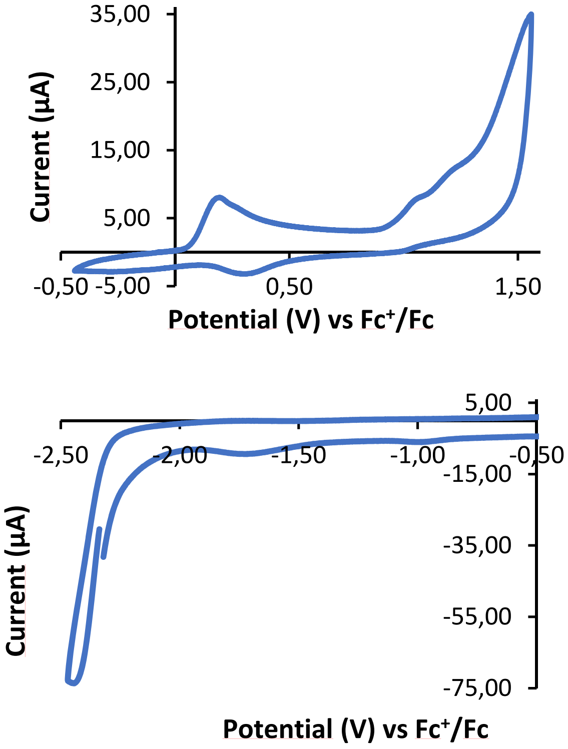

Figure S83. Cyclic voltammogram of complex **17** in dichloromethane 10^{-3} M solution with $[Bu_4N]PF_6$ as supporting electrolyte (0.1 M) at a scan rate of 100 mV s^{-1}. The potentials were referenced to the Fc/Fc^{+} couple.

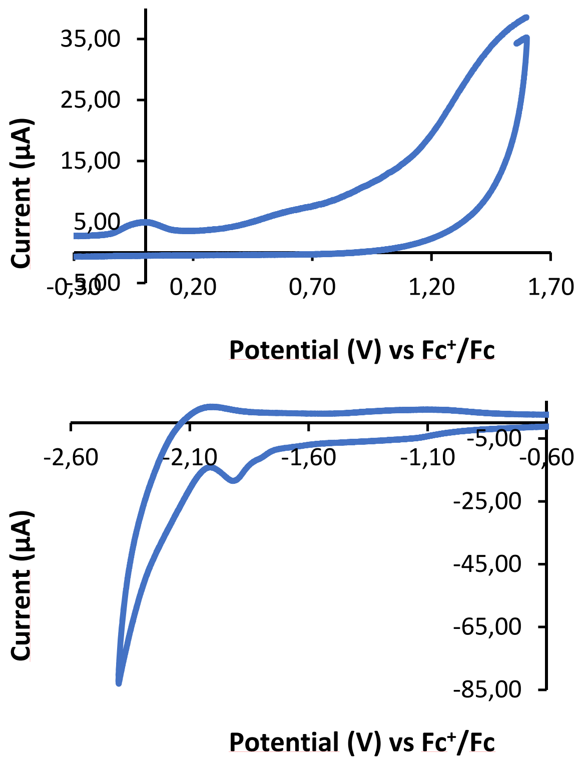

Figure S84. Cyclic voltammogram of complex **18** in dichloromethane 10^{-3} M solution with $[Bu_4N]PF_6$ as supporting electrolyte (0.1 M) at a scan rate of 100 mV s^{-1}. The potentials were referenced to the Fc/Fc^+ couple.

• Photophysical studies

Table S15. Photophysical Data of Complexes 1, 2, 7-10, and 16-18

Complex	Medium/concentration/T (K)	λ_{em} (nm)[a]	τ (μs) green-shifted band[b]	τ (μs) red-shifted band[b]	Φ_L[c]
**1**	PMMA / 2% weight / 298	**492**, 526, 568, 674	5.4 (86.7%), 2.9 (13.3%)	4.9 (16.2%), 1.5 (83.8%)	0.67
	PMMA / 5% weight / 298	**494**, 528, 572, 674	5.1 (79.6%), 2.5 (20.4%)	4.3 (16.4%), 1.6 (83.6%)	0.55
	CH_2Cl_2 / 1 x 10^{-3} M/ 298	490, 524, 560, **690**	0.2	0.8	0.25
	CH_2Cl_2 / 1 x 10^{-4} M / 298	**490**, 526, 566, 684	1.6	-	0.40
	CH_2Cl_2 / 1 x 10^{-5} M / 298	**490**, 524,562	4.1	-	0.60
	CH_2Cl_2 / 1 x 10^{-6} M / 298	**490**, 526, 564	4.8	-	-
	CH_2Cl_2 / 1 x 10^{-3} M / 77	490,500, 526, 566, **668**	5.5 (96.6%), 2.2 (3.4%)	8.4 (1.8%), 3.0 (98.2%)	-
	CH_2Cl_2 / 1 x 10^{-4} M / 77	488, 526, 568, **662**	5.7 (83.7%), 2.8 (16.3%)	9.3 (2.3%), 3.1 (97.7%)	-
	CH_2Cl_2 / 1 x 10^{-5} M / 77	490, 524, 568, **656**	8.3 (26.9%), 4.8 (73.1%)	7.5 (5.1%), 3.2 (94.9%)	-
	CH_2Cl_2 / 1 x 10^{-6} M / 77	**490,** 520, 562, 652	8.6 (40.1%), 4.9 (59.9%)	-	-
**2**	PMMA / 2% weight / 298	**500**, 532, 574, 678	5.5 (75.8%), 2.7 (24.2%)	4.9 (22.5%), 1.9 (77.5%)	0.63
	PMMA / 5% weight / 298	**504**, 534, 576, 670	5.6 (64.3%), 2.6 (35.7%)	5.1 (13.5%), 2.0 (86.5%)	0.43
	CH_2Cl_2 / 1 x 10^{-3} M/ 298	496, 528, **692**	0.2	0.9	0.25
	CH_2Cl_2 / 1 x 10^{-4} M / 298	**494**, 530, 570, 684	1.4	-	0.40
	CH_2Cl_2 / 1 x 10^{-5} M / 298	**494**, 528, 570, 674	4.2	-	0.60
	CH_2Cl_2 / 1 x 10^{-6} M / 298	**494**, 530, 568	5.6	-	-
	CH_2Cl_2 / 1 x 10^{-3} M / 77	**506**, 544, 588, 656	8.6 (66.5%), 4.1 (33.5%)	10.0 (11.0%), 4.5 (89.0%)	-
	CH_2Cl_2 / 1 x 10^{-4} M / 77	**506**, 544, 582, 660	9.7 (63.7%), 4.5 (36.3%)	10.5 (16.8%), 4.5 (83.2%)	-
	CH_2Cl_2 / 1 x 10^{-5} M / 77	**504**, 540, 580, 654	9.1 (66.4%), 3.9 (33.6%)	8.8 (29.6%), 4.1 (70.4%)	-
	CH_2Cl_2 / 1 x 10^{-6} M / 77	**496**, 528, 568, 654	9.4 (54.0%), 4.4 (46.0%)	-	-
**7**	PMMA / 2% weight / 298	**490**, 524, 564, 638	5.1 (76.9%), 2.8 (23.1%)	4.9 (45.0%), 2.0 (55.0%)	0.60
	PMMA / 5% weight / 298	**492**, 528, 570, 674	4.5 (69.9%), 1.9 (30.1%)	4.1 (38.7%), 1.7 (61.3%)	0.30
	CH_2Cl_2 / 1 x 10^{-3} M/ 298	**490**, 524, 558, 664	0.3 (77.8%), 0.2 (22.2%)	0.8 (12.0%), 0.3 (88.0%)	0.05
	CH_2Cl_2 / 1 x 10^{-4} M / 298	**490**, 522, 562	1.5	-	0.26
	CH_2Cl_2 / 1 x 10^{-5} M / 298	**490**, 524, 564	4.2	-	0.60
	CH_2Cl_2 / 1 x 10^{-6} M / 298	**490**, 526, 562	5.4	-	-
	CH_2Cl_2 / 1 x 10^{-3} M / 77	486, 522, **646**	6.6 (62.9%), 4.3 (37.1%)	4.4 (32.0%), 1.8 (68.0%)	-
	CH_2Cl_2 / 1 x 10^{-4} M / 77	486, 524, **638**	5.9	3.3 (65.0%), 1.4 (35.0%)	-
	CH_2Cl_2 / 1 x 10^{-5} M / 77	**484**, 522, 636	7.2 (42.3%), 4.8 (57.7%)	3.8 (54.7%), 2.3 (45.3%)	-
	CH_2Cl_2 / 1 x 10^{-6} M / 77	**490,** 526, 564, 646	9.9 (21.5%), 5.3 (78.5%)	14.6 (3.3%), 3.5 (96.7%)	-
**8**	PMMA / 2% weight / 298	**488**, 526, 560	5.1 (78.1%), 3.0 (21.9%)	-	0.72
	PMMA / 5% weight / 298	**494**, 524, 558, 640	4.0 (58.0%), 1.8 (42.0%)	14.6 (20.3%), 2.9 (79.7%)	0.50
	CH_2Cl_2 / 1 x 10^{-3} M/ 298	**490**, 522, 556	0.9	-	0.10
	CH_2Cl_2 / 1 x 10^{-4} M / 298	**488**, 522, 564	2.8	-	0.28
	CH_2Cl_2 / 1 x 10^{-5} M / 298	**488**, 522,562	4.2	-	0.56
	CH_2Cl_2 / 1 x 10^{-6} M / 298	**490**, 526, 564	5.1	-	-
	CH_2Cl_2 / 1 x 10^{-4} M / 77	490, 524, 558, **652**	6.2 (76.2%), 3.6 (23.8%)	23.0 (7.9%), 3.4 (92.1%)	-
	CH_2Cl_2 / 1 x 10^{-5} M / 77	**490**, 526, 556, 652	7.3 (51.1%), 4.2 (48.9%)	35.0 (34.2%), 5.3 (65.8%)	-

	CH_2Cl_2 / 1 x 10^{-6} M / 77	**490,** 526, 570, 654	8.3 (52.0%), 4.2 (48.0%)	-	-
**9**	PMMA / 2% weight / 298	**496**, 532, 574, 640	5.4 (71.0%), 2.4 (29.0%)	4.9 (30.6%), 2.2 (69.4%)	0.57
	PMMA / 5% weight / 298	**498**, 530, 572, 640	4.3 (60.2%), 1.5 (39.8%)	3.6 (27.8%), 1.8 (72.2%)	0.33
	CH_2Cl_2 / 1 x 10^{-3} M/ 298	**494**, 528, 567, 663	0.4	0.3	0.05
	CH_2Cl_2 / 1 x 10^{-4} M / 298	**494**, 528, 566	2.5	-	0.25
	CH_2Cl_2 / 1 x 10^{-5} M / 298	**494**, 530, 570	4.7	-	0.62
	CH_2Cl_2 / 1 x 10^{-6} M / 298	**495**, 530, 569	6.7	-	-
	CH_2Cl_2 / 1 x 10^{-3} M / 77	489, 496, 530, **627**	6.0 (77.4%), 3.3 (22.6%)	4.2 (44.2%), 2.0 (55.8%)	-
	CH_2Cl_2 / 1 x 10^{-4} M / 77	487, 499, 527, **641**	6.1 (65.4%), 3.4 (34.6%)	3.8 (54.3%), 1.4 (45.7%)	-
	CH_2Cl_2 / 1 x 10^{-5} M / 77	486, **496**, 526, 625	6.5 (70.3%), 3.0 (29.7%)	4.4 (42.4%), 2.3 (57.6%)	-
	CH_2Cl_2 / 1 x 10^{-6} M / 77	**498**, 532, 579, 662	13.8 (15.4%), 6.4 (84.6%)	4.0 (77.0%), 2.3 (23.0%)	-
**10**	PMMA / 2% weight / 298	**494**, 528, 566, 646	5.6 (78.4%), 2.7 (21.6%)	4.9 (45.0%), 2.0 (55.0%)	
	PMMA / 5% weight / 298	**498**, 530, 576, 640	4.5 (65.9%), 1.4 (34.1%)	3.6 (30.2%), 2.0 (69.8%)	0.75
	CH_2Cl_2 / 1 x 10^{-3} M/ 298	**494**, 528, 564	2.3	-	0.50
	CH_2Cl_2 / 1 x 10^{-4} M / 298	**492**, 526, 566	4.7	-	0.22
	CH_2Cl_2 / 1 x 10^{-5} M / 298	**494**, 526, 560	5.6	-	0.47
	CH_2Cl_2 / 1 x 10^{-6} M / 298	**494**, 528, 568	12.0 (4.0%), 6.1 (96.0%)	-	0.60
	CH_2Cl_2 / 1 x 10^{-3} M / 77	484, 500, 540, **640**	5.2 (45.0%), 1.8 (55.0%)	32 (0.7%), 4.4 (99.3%)	-
	CH_2Cl_2 / 1 x 10^{-4} M / 77	484, 498, 520, **634**	7.4 (22.0%), 4.9 (78.0%)	4.1	-
	CH_2Cl_2 / 1 x 10^{-5} M / 77	**498**, 530, 648	8.0 (64.0%), 3.7 (36.0%)	3.8 (73.1%), 1.4 (26.9%)	-
	CH_2Cl_2 / 1 x 10^{-6} M / 77	**493**, 526, 571, 638	8.8 (61.5%), 4.1 (38.5%)	-	-
**16**	PMMA / 5% weight / 298	**490**, 523, 561	1.5 (36.9%), 4.2 (63.1%)	-	0.01
	CH_2Cl_2 / 1 x 10^{-3} M/ 298	**490**, 525, 570, 681	0.5	-	0.03
	CH_2Cl_2 / 1 x 10^{-4} M / 298	**490**, 526, 567, 647	1.1	-	0.08
	CH_2Cl_2 / 1 x 10^{-5} M / 298	**491**, 525, 563	3.6	-	0.03
	CH_2Cl_2 / 1 x 10^{-6} M / 298	**491**, 526, 563	5.0	-	-
	CH_2Cl_2 / 1 x 10^{-3} M / 77	485, **497,** 524, 571, 654	11.2 (54.6%), 4.7 (45.4%)	-	-
	CH_2Cl_2 / 1 x 10^{-4} M / 77	**485,** 525, 569, 647	9.7 (52.1%), 4.3 (47.9%)	-	-
	CH_2Cl_2 / 1 x 10^{-5} M / 77	**482**, 519, 556, 650	10.4 (48.3%), 6.5 (51.7%)	-	-
	CH_2Cl_2 / 1 x 10^{-6} M / 77	**481**, 519, 553, 652	9.3 (56.2%), 5.3 (43.8%)	-	-
**17**	PMMA / 5% weight / 298	**495**, 527, 571	0.5 (5.4%), 3.6 (94.6%)	-	0.03
	CH_2Cl_2 / 1 x 10^{-4} M / 298	**494**, 528, 569	1.1	-	0.008
	CH_2Cl_2 / 1 x 10^{-5} M / 298	**494**, 527, 569	3.6	-	0.03
	CH_2Cl_2 / 1 x 10^{-6} M / 298	**494**, 527, 572	5.0	-	0.06
	CH_2Cl_2 / 1 x 10^{-4} M / 77	**498**, 534, 573	9.8 (62.1%), 4.5 (37.9%)	-	-
	CH_2Cl_2 / 1 x 10^{-5} M / 77	**496**, 530, 572	8.8 (62.8%), 3.4 (37.2%)	-	-
	CH_2Cl_2 / 1 x 10^{-6} M / 77	**484**, 521, 557	9.5 (53.0%), 5.5 (47.0%)	-	-
**18**	PMMA / 5% weight / 298	483, **511**	0.4 (2.5%), 5.6 (97.5%)	-	0.12
	CH_2Cl_2 / 1 x 10^{-3} M/ 298	493, **520**	9.9 (91.7%), 3.7 (8.3%)	-	0.03
	CH_2Cl_2 / 1 x 10^{-4} M / 298	494, **518**	36.7 (0.7%), 13.4 (99.3%)	-	0.03
	CH_2Cl_2 / 1 x 10^{-5} M / 298	**495**, 525, 570	23.4 (12.6%), 12.2 (87.4%)		0.10
	CH_2Cl_2 / 1 x 10^{-6} M / 298	**489**, 519, 563	-	-	-
	CH_2Cl_2 / 1 x 10^{-3} M / 77	475, **509,** 531	53.6 (52.3%), 27.1 (47.7%)	-	-
	CH_2Cl_2 / 1 x 10^{-4} M / 77	475, **508,** 532	54.9 (50.6%), 28.7 (49.4%)	-	-
	CH_2Cl_2 / 1 x 10^{-5} M / 77	**492**, 525, 562	51.0 (44.9%), 10.1 (55.1%)	-	-

CH_2Cl_2 / 1 x 10^{-6} M / 77	475, **490**, 511	-	-	-

(a) The most intense peak is in bold. (b) Relative amplitudes (%) are given in parentheses for biexponential decays. (c) Absolute quantum yield.

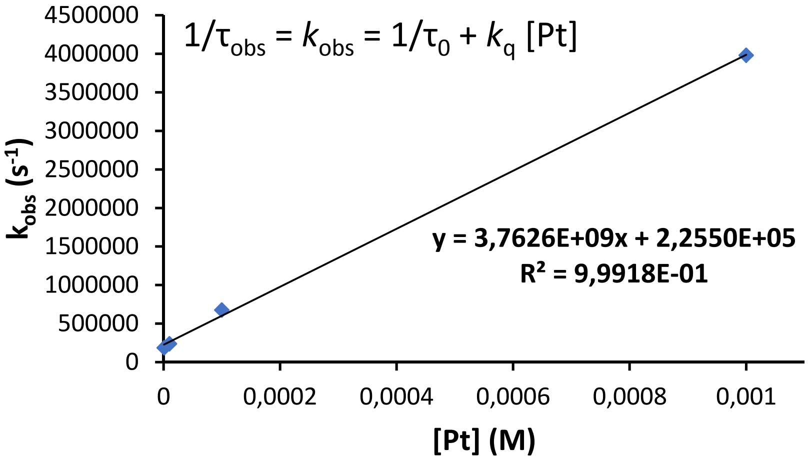

Figure S85. Stern-Volmer plot (λ_{em} = 490 nm) for **7** in dichloromethane solution at 298 K. The slope is k_q and the y intercept is $1/\tau_0$ (amplitude-weighted average lifetime time has been used for the 1 x 10^{-3} M concentration).

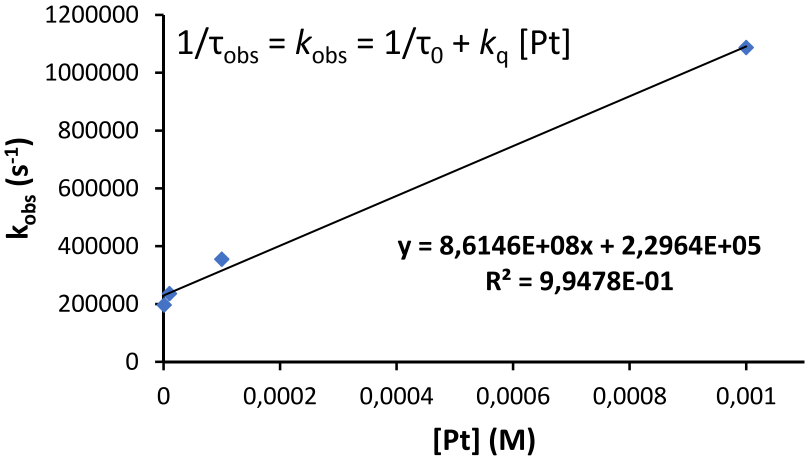

Figure S86. Stern-Volmer plot (λ_{em} = 489 nm) for **8** in dichloromethane solution at 298 K. The slope is k_q and the y intercept is $1/\tau_0$.

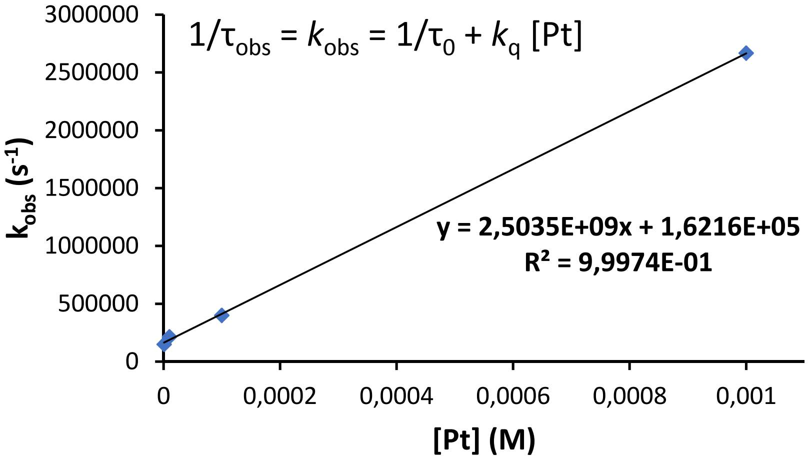

Figure S87. Stern-Volmer plot (λ_{em} = 494 nm) for **9** in dichloromethane solution at 298 K. The slope is k_q and the y intercept is $1/\tau_0$.

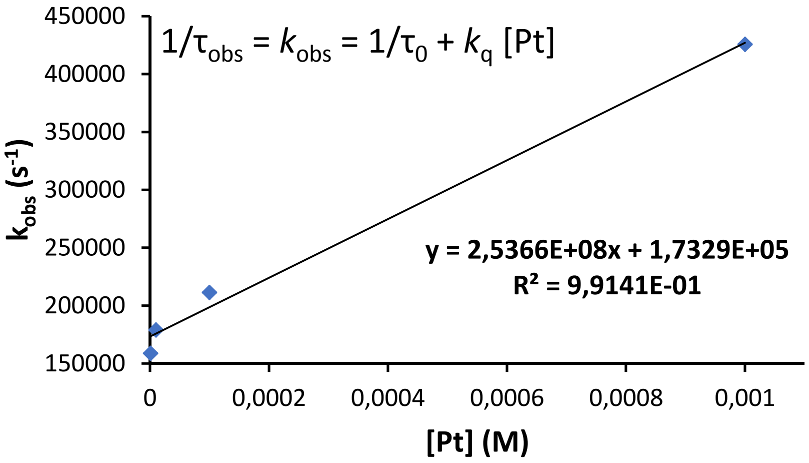

Figure S88. Stern-Volmer plot (λ_{em} = 494 nm) for **10** in dichloromethane solution at 298 K. The slope is k_q and the y intercept is $1/\tau_0$ (amplitude-weighted average lifetime time has been used for the 1 x 10^{-6} M concentration).

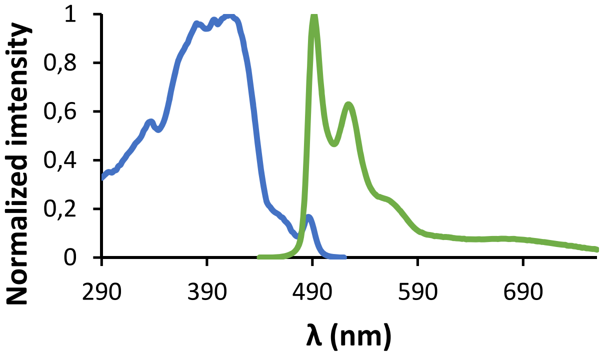

Figure S89. Normalized excitation (blue line; λ_{em} = 560 nm) and emission (green line; λ_{exc} = 420 nm) spectra of **1** in PMMA film (2 wt%) at 298 K.

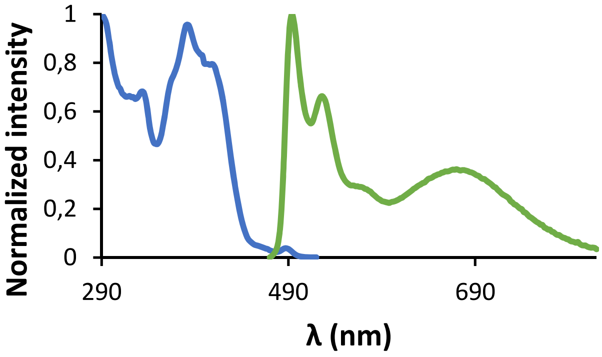

Figure S90. Normalized excitation (blue line; λ_{em} = 560 nm) and emission (green line; λ_{exc} = 420 nm) spectra of **1** in PMMA film (5 wt%) at 298 K.

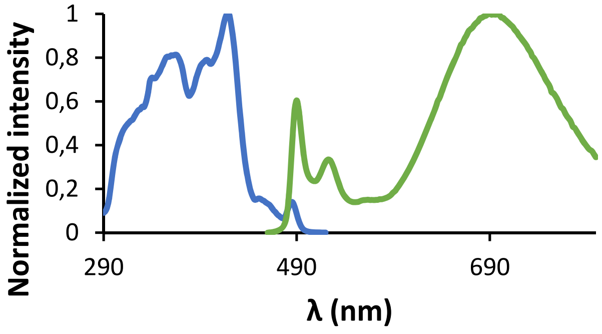

Figure S91. Normalized excitation (blue line; λ_{em} = 560 nm) and emission (green line; λ_{exc} = 420 nm) spectra of **1** in a 1.0 x 10^{-3} M solution in dichloromethane at 298 K.

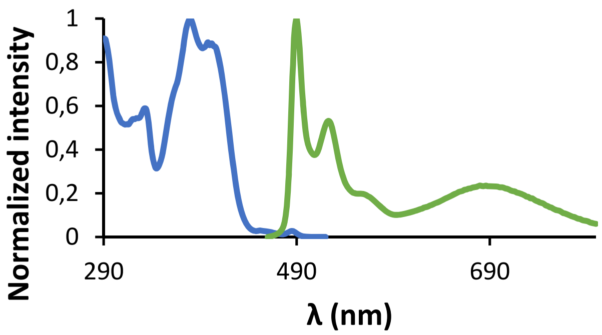

Figure S92. Normalized excitation (blue line; λ_{em} = 560 nm) and emission (green line; λ_{exc} = 420 nm) spectra of **1** in a 1.0 x 10^{-4} M solution in dichloromethane at 298 K.

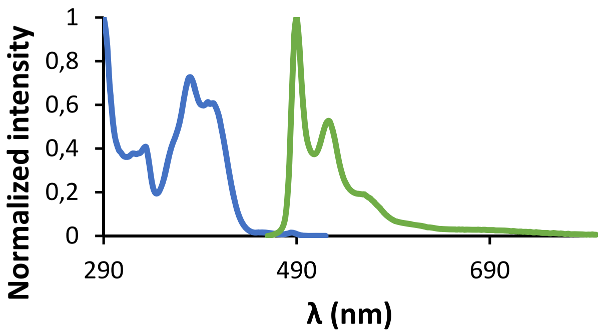

Figure S93. Normalized excitation (blue line; λ_{em} = 560 nm) and emission (green line; λ_{exc} = 420 nm) spectra of **1** in a 1.0 x 10^{-5} M solution in dichloromethane at 298 K.

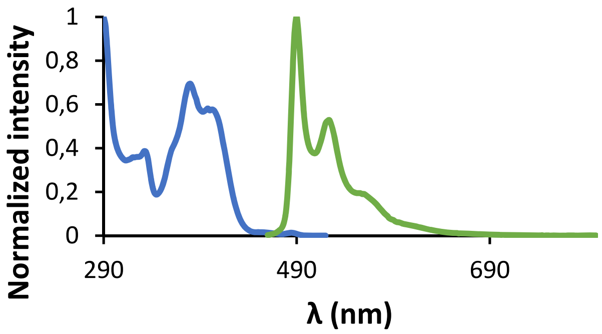

Figure S94. Normalized excitation (blue line; λ_{em} = 560 nm) and emission (green line; λ_{exc} = 420 nm) spectra of **1** in a 1.0 x 10^{-6} M solution in dichloromethane at 298 K.

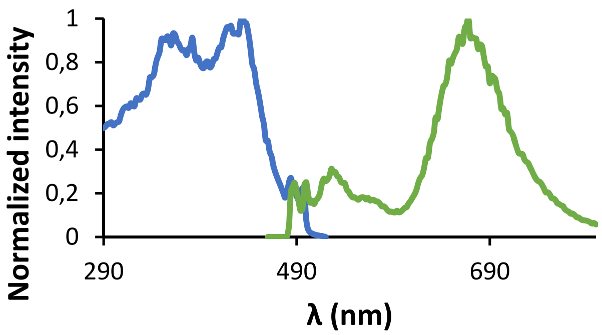

Figure S95. Normalized excitation (blue line; λ_{em} = 560 nm) and emission (green line; λ_{exc} = 420 nm) spectra of **1** in a 1.0 x 10^{-3} M solution in dichloromethane at 77 K.

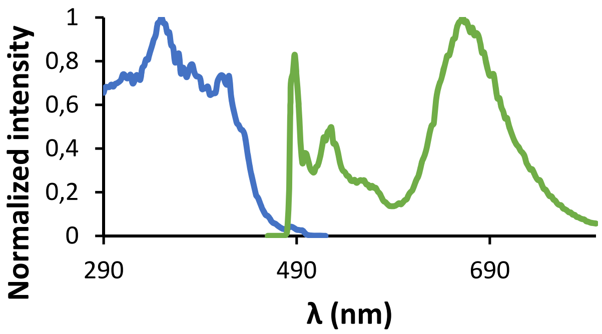

Figure S96. Normalized excitation (blue line; λ_{em} = 560 nm) and emission (green line; λ_{exc} = 420 nm) spectra of **1** in a 1.0 x 10^{-4} M solution in dichloromethane at 77 K.

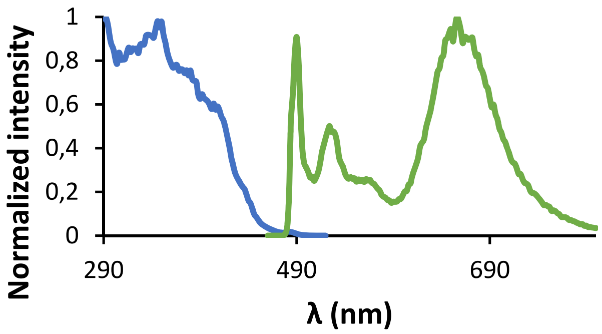

Figure S97. Normalized excitation (blue line; λ_{em} = 560 nm) and emission (green line; λ_{exc} = 420 nm) spectra of **1** in a 1.0 x 10^{-5} M solution in dichloromethane at 77 K.

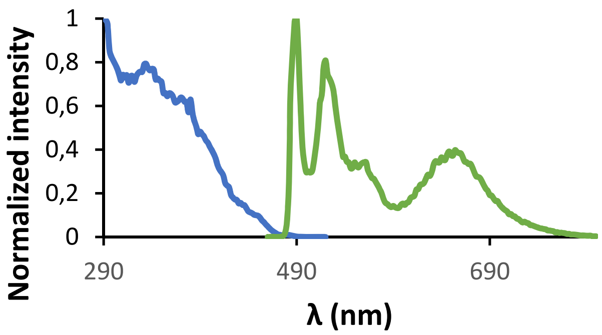

Figure S98. Normalized excitation (blue line; λ_{em} = 560 nm) and emission (green line; λ_{exc} = 420 nm) spectra of **1** in a 1.0 x 10^{-6} M solution in dichloromethane at 77 K.

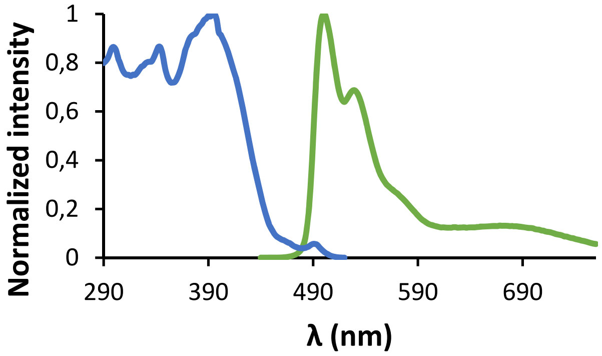

Figure S99. Normalized excitation (blue line; λ_{em} = 560 nm) and emission (green line; λ_{exc} = 420 nm) spectra of **2** in PMMA film (2 wt%) at 298 K.

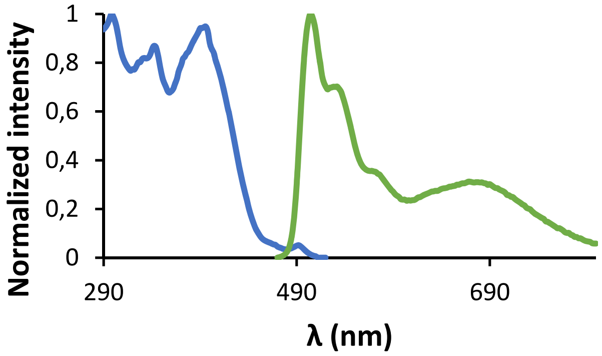

Figure S100. Normalized excitation (blue line; λ_{em} = 560 nm) and emission (green line; λ_{exc} = 420 nm) spectra of **2** in PMMA film (5 wt%) at 298 K.

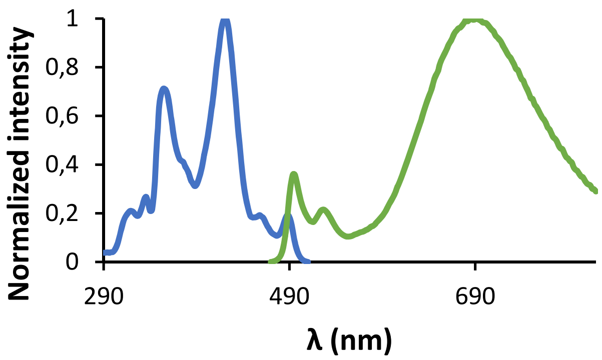

Figure S101. Normalized excitation (blue line; λ_{em} = 560 nm) and emission (green line; λ_{exc} = 420 nm) spectra of **2** in a 1.0 x 10^{-3} M solution in dichloromethane at 298 K.

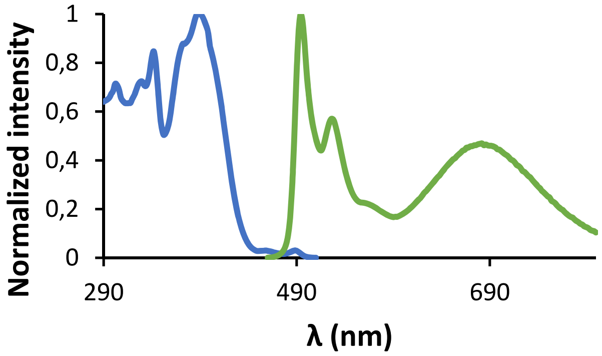

Figure S102. Normalized excitation (blue line; λ_{em} = 560 nm) and emission (green line; λ_{exc} = 420 nm) spectra of **2** in a 1.0 x 10^{-4} M solution in dichloromethane at 298 K.

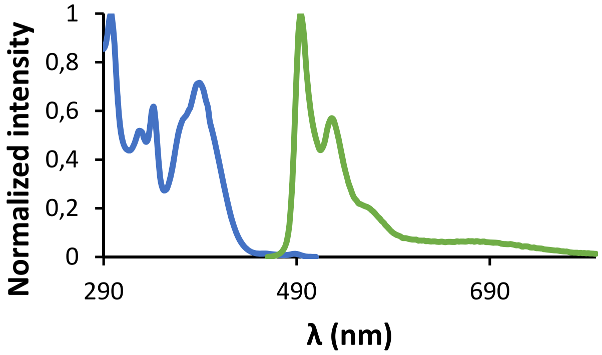

Figure S103. Normalized excitation (blue line; λ_{em} = 560 nm) and emission (green line; λ_{exc} = 420 nm) spectra of **2** in a 1.0 x 10^{-5} M solution in dichloromethane at 298 K.

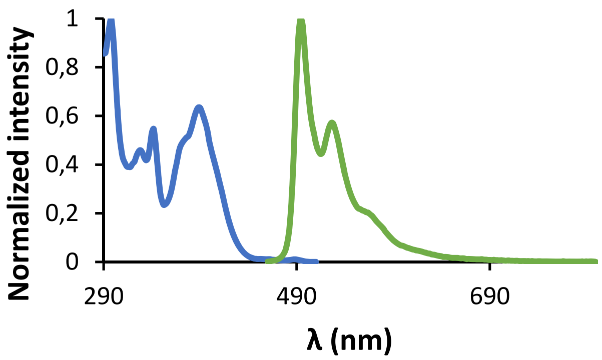

Figure S104. Normalized excitation (blue line; λ_{em} = 560 nm) and emission (green line; λ_{exc} = 420 nm) spectra of **2** in a 1.0 x 10^{-6} M solution in dichloromethane at 298 K.

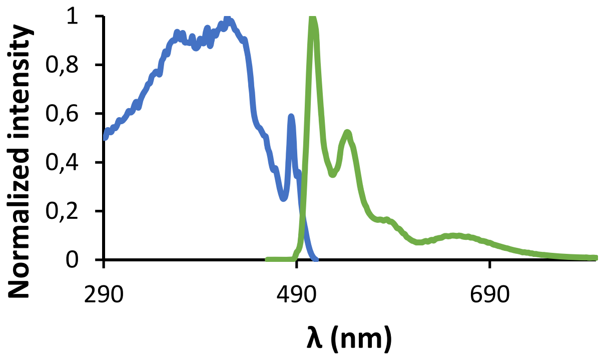

Figure S105. Normalized excitation (blue line; λ_{em} = 560 nm) and emission (green line; λ_{exc} = 420 nm) spectra of **2** in a 1.0 x 10^{-3} M solution in dichloromethane at 77 K.

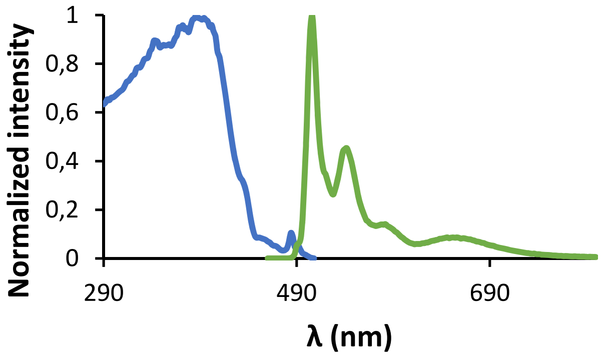

Figure S106. Normalized excitation (blue line; λ_{em} = 560 nm) and emission (green line; λ_{exc} = 420 nm) spectra of **2** in a 1.0 x 10^{-4} M solution in dichloromethane at 77 K.

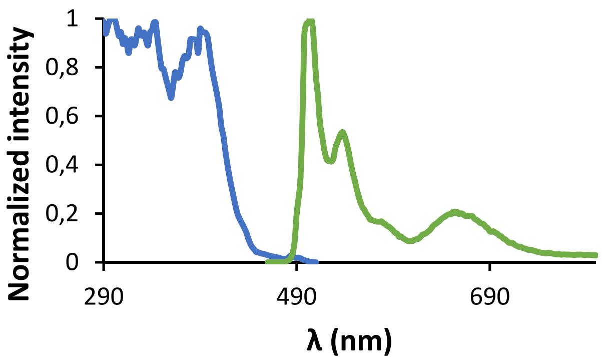

Figure S107. Normalized excitation (blue line; λ_{em} = 560 nm) and emission (green line; λ_{exc} = 420 nm) spectra of **2** in a 1.0 x 10^{-5} M solution in dichloromethane at 77 K.

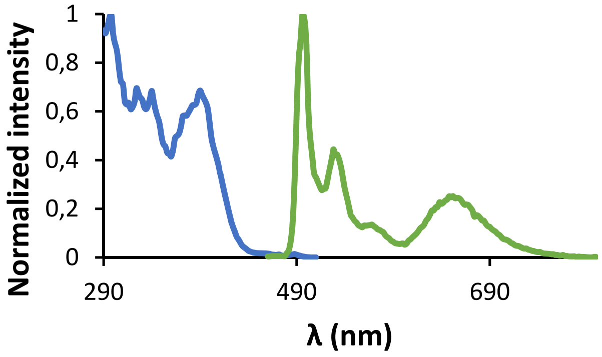

Figure S108. Normalized excitation (blue line; λ_{em} = 560 nm) and emission (green line; λ_{exc} = 420 nm) spectra of **2** in a 1.0 x 10^{-6} M solution in dichloromethane at 77 K.

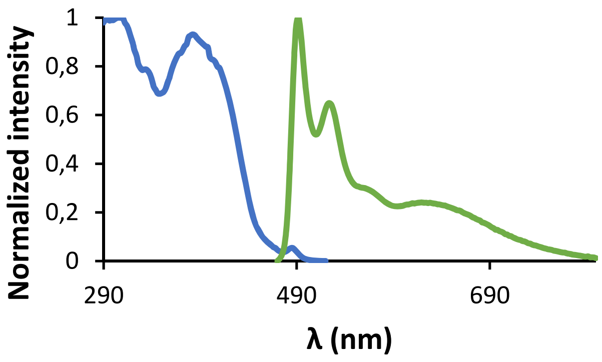

Figure S109. Normalized excitation (blue line; λ_{em} = 560 nm) and emission (green line; λ_{exc} = 420 nm) spectra of **7** in PMMA film (2 wt%) at 298 K.

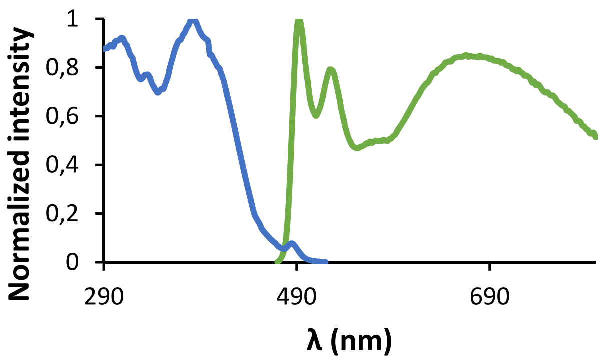

Figure S110. Normalized excitation (blue line; λ_{em} = 560 nm) and emission (green line; λ_{exc} = 420 nm) spectra of **7** in PMMA film (5 wt%) at 298 K.

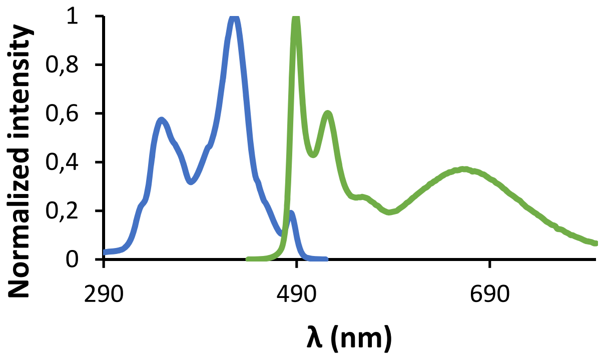

Figure S111. Normalized excitation (blue line; λ_{em} = 560 nm) and emission (green line; λ_{exc} = 420 nm) spectra of **7** in a 1.0 x 10^{-3} M solution in dichloromethane at 298 K.

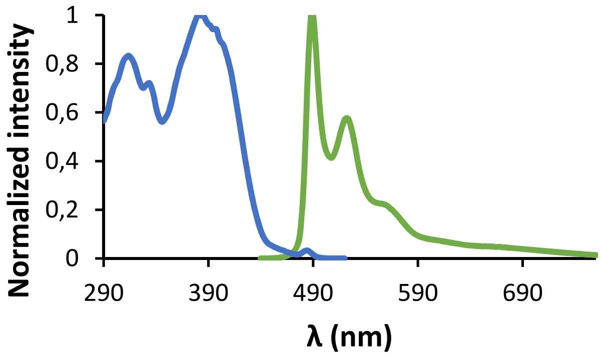

Figure S112. Normalized excitation (blue line; λ_{em} = 560 nm) and emission (green line; λ_{exc} = 420 nm) spectra of **7** in a 1.0 x 10^{-4} M solution in dichloromethane at 298 K.

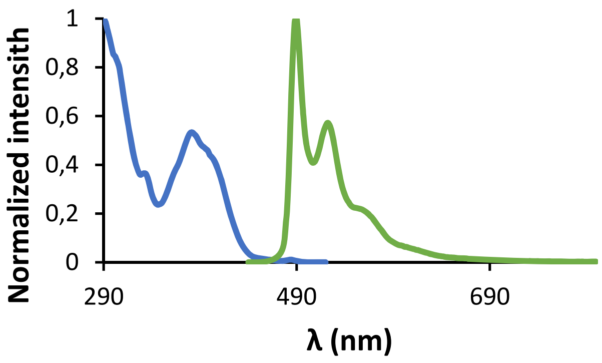

Figure S113. Normalized excitation (blue line; λ_{em} = 560 nm) and emission (green line; λ_{exc} = 420 nm) spectra of **7** in a 1.0 x 10^{-5} M solution in dichloromethane at 298 K.

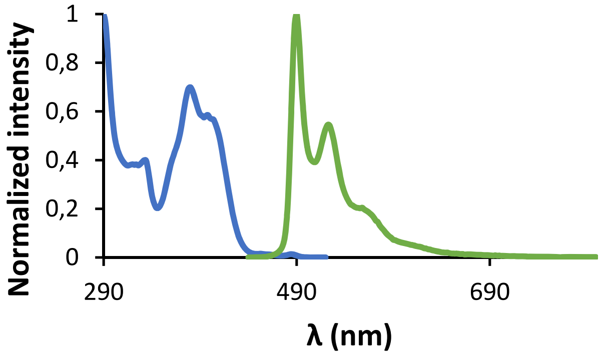

Figure S114. Normalized excitation (blue line; λ_{em} = 560 nm) and emission (green line; λ_{exc} = 420 nm) spectra of **7** in a 1.0 x 10^{-6} M solution in dichloromethane at 298 K.

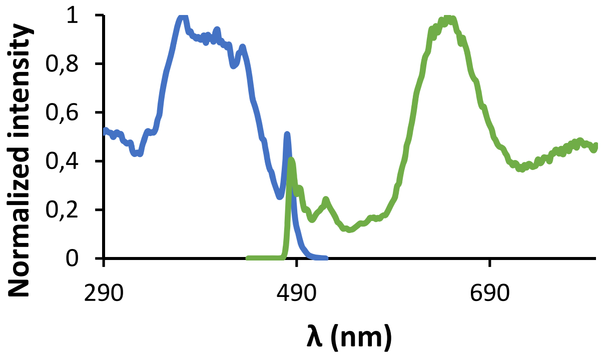

Figure S115. Normalized excitation (blue line; λ_{em} = 560 nm) and emission (green line; λ_{exc} = 420 nm) spectra of **7** in a 1.0 x 10^{-3} M solution in dichloromethane at 77 K.

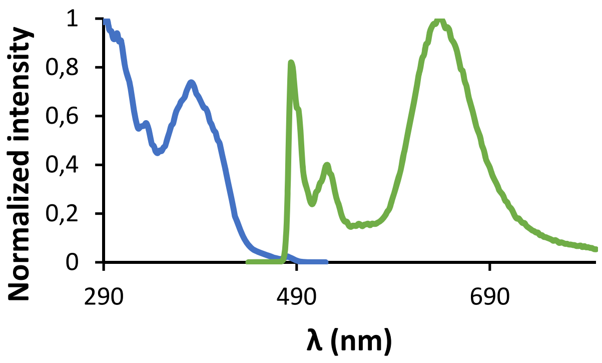

Figure S116. Normalized excitation (blue line; λ_{em} = 560 nm) and emission (green line; λ_{exc} = 420 nm) spectra of **7** in a 1.0 x 10^{-4} M solution in dichloromethane at 77 K.

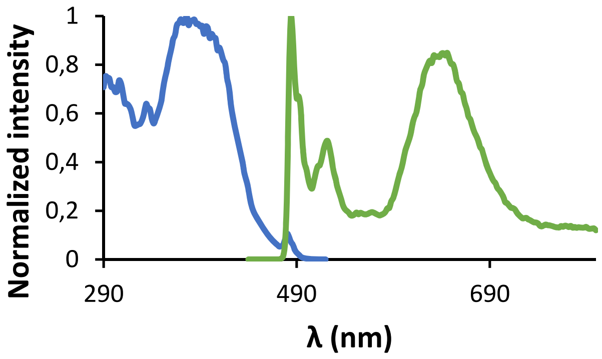

Figure S117. Normalized excitation (blue line; λ_{em} = 560 nm) and emission (green line; λ_{exc} = 420 nm) spectra of **7** in a 1.0 x 10^{-5} M solution in dichloromethane at 77 K.

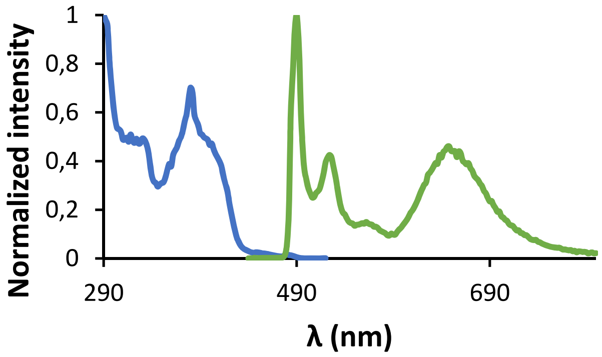

Figure S118. Normalized excitation (blue line; λ_{em} = 560 nm) and emission (green line; λ_{exc} = 420 nm) spectra of **7** in a 1.0 x 10^{-6} M solution in dichloromethane at 77 K.

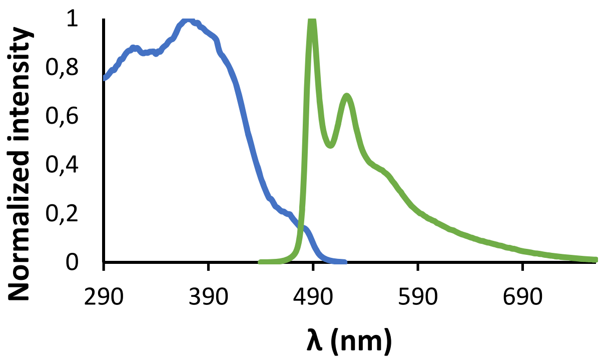

Figure S119. Normalized excitation (blue line; λ_{em} = 560 nm) and emission (green line; λ_{exc} = 420 nm) spectra of **8** in PMMA film (2 wt%) at 298 K.

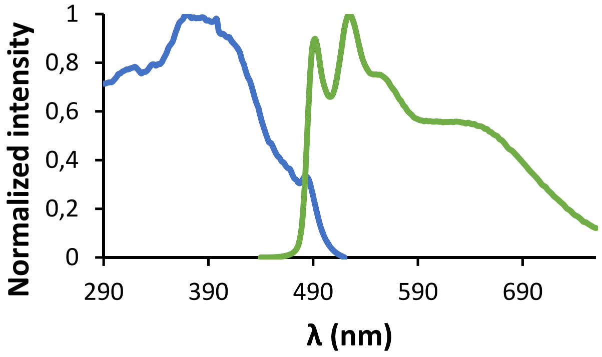

Figure S120. Normalized excitation (blue line; λ_{em} = 560 nm) and emission (green line; λ_{exc} = 420 nm) spectra of **8** in PMMA film (5 wt%) at 298 K.

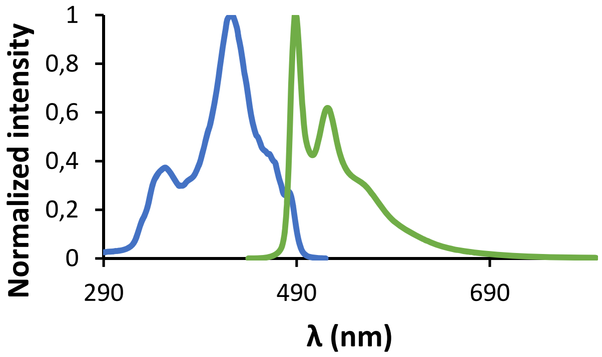

Figure S121. Normalized excitation (blue line; λ_{em} = 560 nm) and emission (green line; λ_{exc} = 420 nm) spectra of **8** in a 1.0 x 10^{-3} M solution in dichloromethane at 298 K.

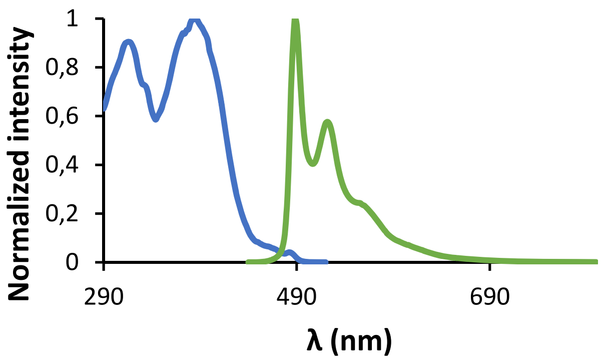

Figure S122. Normalized excitation (blue line; λ_{em} = 560 nm) and emission (green line; λ_{exc} = 420 nm) spectra of **8** in a 1.0 x 10^{-4} M solution in dichloromethane at 298 K.

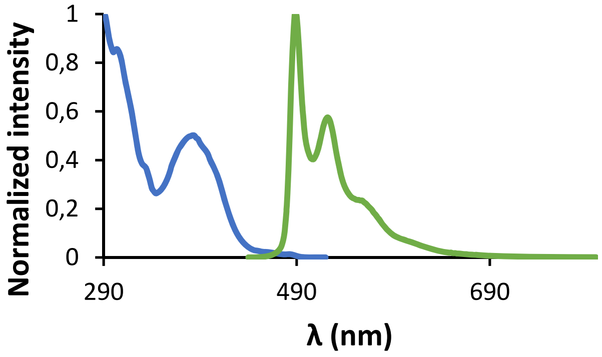

Figure S123. Normalized excitation (blue line; λ_{em} = 560 nm) and emission (green line; λ_{exc} = 420 nm) spectra of **8** in a 1.0 x 10^{-5} M solution in dichloromethane at 298 K.

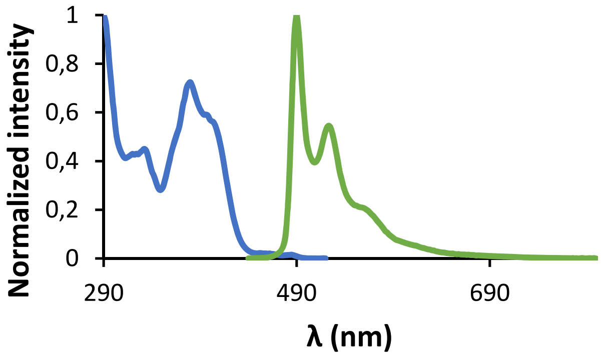

Figure S124. Normalized excitation (blue line; λ_{em} = 560 nm) and emission (green line; λ_{exc} = 420 nm) spectra of **8** in a 1.0 x 10^{-6} M solution in dichloromethane at 298 K.

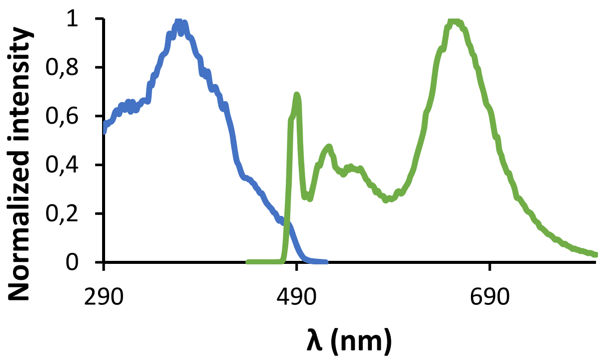

Figure S125. Normalized excitation (blue line; λ_{em} = 560 nm) and emission (green line; λ_{exc} = 420 nm) spectra of **8** in a 1.0 x 10^{-4} M solution in dichloromethane at 77 K.

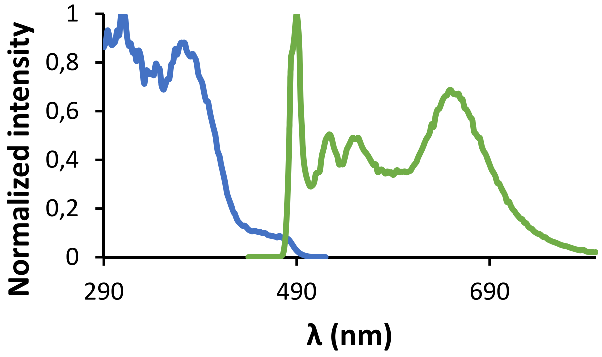

Figure S126. Normalized excitation (blue line; λ_{em} = 560 nm) and emission (green line; λ_{exc} = 420 nm) spectra of **8** in a 1.0 x 10^{-5} M solution in dichloromethane at 77 K.

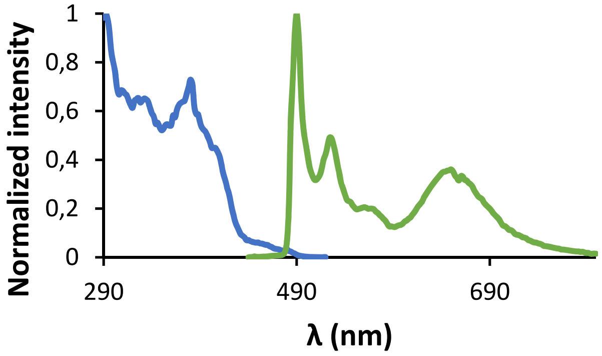

Figure S127. Normalized excitation (blue line; λ_{em} = 560 nm) and emission (green line; λ_{exc} = 420 nm) spectra of **8** in a 1.0 x 10^{-6} M solution in dichloromethane at 77 K.

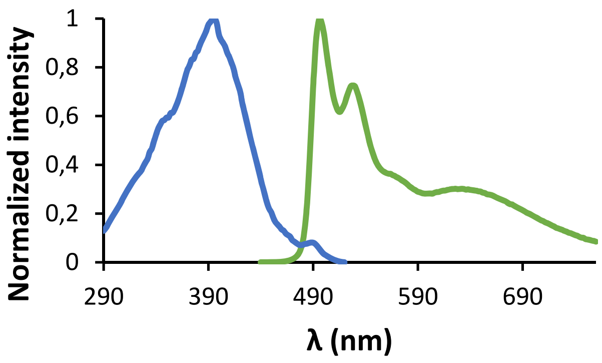

Figure S128. Normalized excitation (blue line; λ_{em} = 560 nm) and emission (green line; λ_{exc} = 420 nm) spectra of **9** in PMMA film (2 wt%) at 298 K.

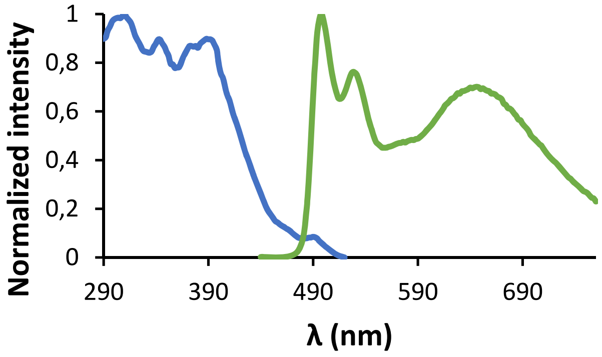

Figure S129. Normalized excitation (blue line; λ_{em} = 560 nm) and emission (green line; λ_{exc} = 420 nm) spectra of **9** in PMMA film (5 wt%) at 298 K.

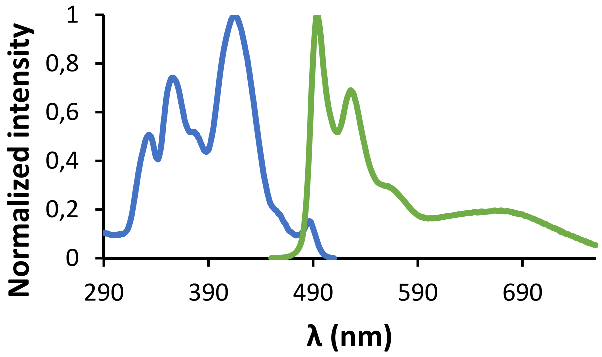

Figure S130. Normalized excitation (blue line; λ_{em} = 560 nm) and emission (green line; λ_{exc} = 420 nm) spectra of **9** in a 1.0 x 10^{-3} M solution in dichloromethane at 298 K.

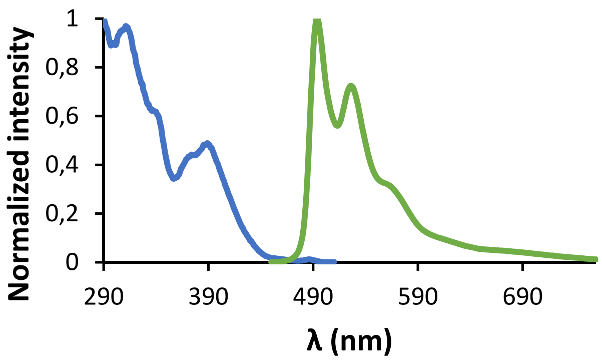

Figure S131. Normalized excitation (blue line; λ_{em} = 560 nm) and emission (green line; λ_{exc} = 420 nm) spectra of **9** in a 1.0 x 10^{-4} M solution in dichloromethane at 298 K.

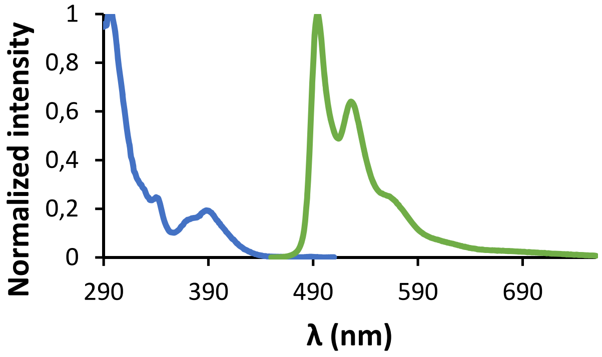

Figure S132. Normalized excitation (blue line; λ_{em} = 560 nm) and emission (green line; λ_{exc} = 420 nm) spectra of **9** in a 1.0 x 10^{-5} M solution in dichloromethane at 298 K.

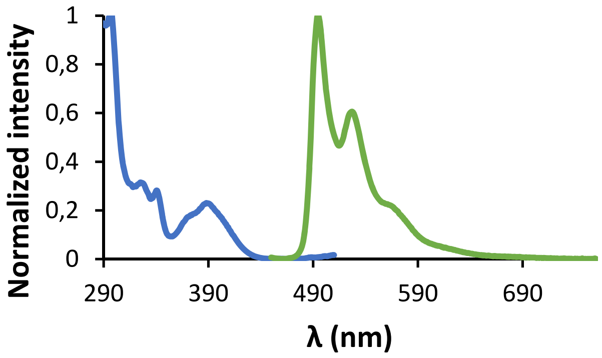

Figure S133. Normalized excitation (blue line; λ_{em} = 560 nm) and emission (green line; λ_{exc} = 420 nm) spectra of **9** in a 1.0 x 10^{-6} M solution in dichloromethane at 298 K.

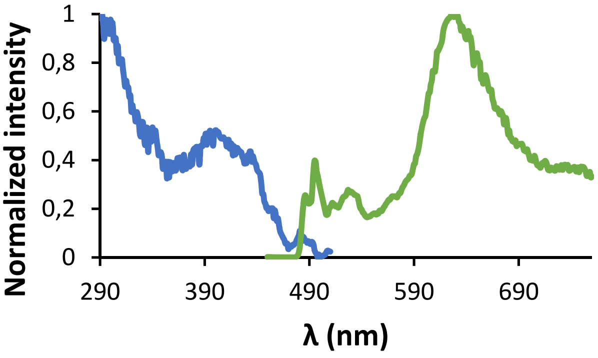

Figure S134. Normalized excitation (blue line; λ_{em} = 560 nm) and emission (green line; λ_{exc} = 420 nm) spectra of **9** in a 1.0 x 10^{-3} M solution in dichloromethane at 77 K.

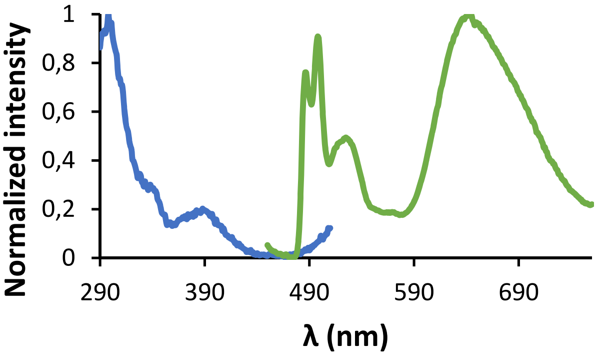

Figure S135. Normalized excitation (blue line; λ_{em} = 560 nm) and emission (green line; λ_{exc} = 420 nm) spectra of **9** in a 1.0 x 10^{-4} M solution in dichloromethane at 77 K.

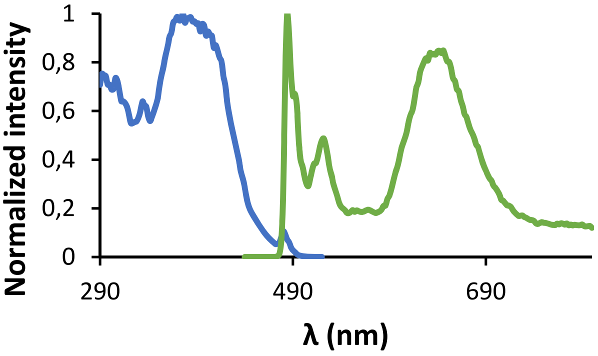

Figure S136. Normalized excitation (blue line; λ_{em} = 560 nm) and emission (green line; λ_{exc} = 420 nm) spectra of **9** in a 1.0 x 10^{-5} M solution in dichloromethane at 77 K.

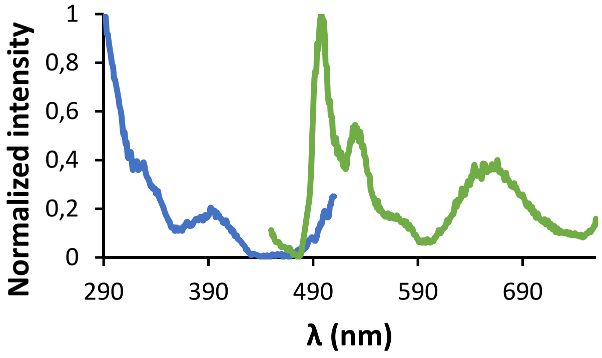

Figure S137. Normalized excitation (blue line; λ_{em} = 560 nm) and emission (green line; λ_{exc} = 420 nm) spectra of **9** in a 1.0 x 10^{-6} M solution in dichloromethane at 77 K.

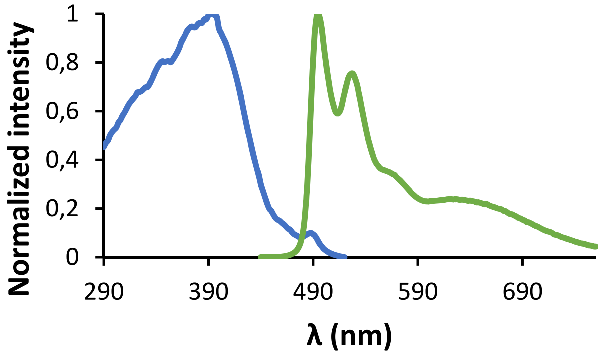

Figure S138. Normalized excitation (blue line; λ_{em} = 560 nm) and emission (green line; λ_{exc} = 420 nm) spectra of **10** in PMMA film (2 wt%) at 298 K.

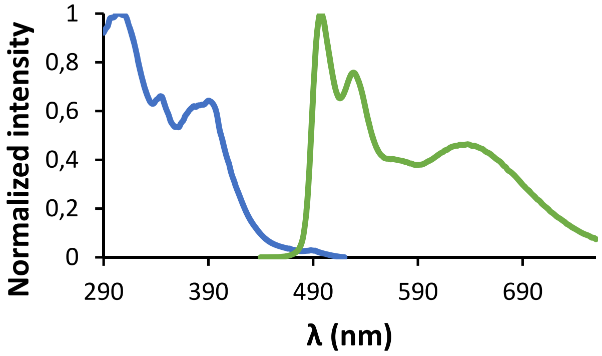

Figure S139. Normalized excitation (blue line; λ_{em} = 560 nm) and emission (green line; λ_{exc} = 420 nm) spectra of **10** in PMMA film (5 wt%) at 298 K.

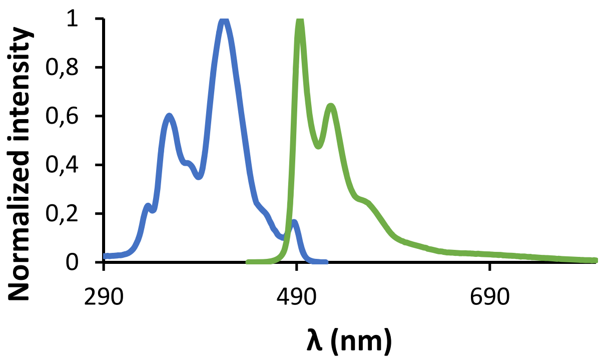

Figure S140. Normalized excitation (blue line; λ_{em} = 560 nm) and emission (green line; λ_{exc} = 420 nm) spectra of **10** in a 1.0 x 10^{-3} M solution in dichloromethane at 298 K.

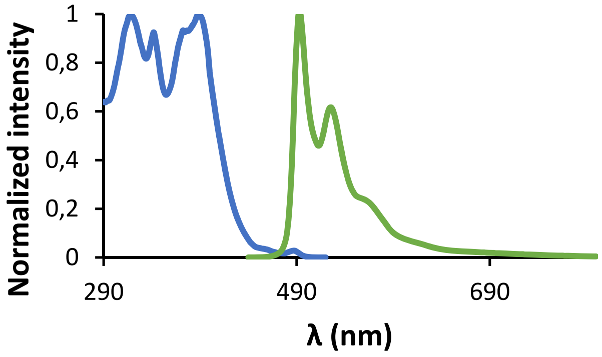

Figure S141. Normalized excitation (blue line; λ_{em} = 560 nm) and emission (green line; λ_{exc} = 420 nm) spectra of **10** in a 1.0 x 10^{-4} M solution in dichloromethane at 298 K.

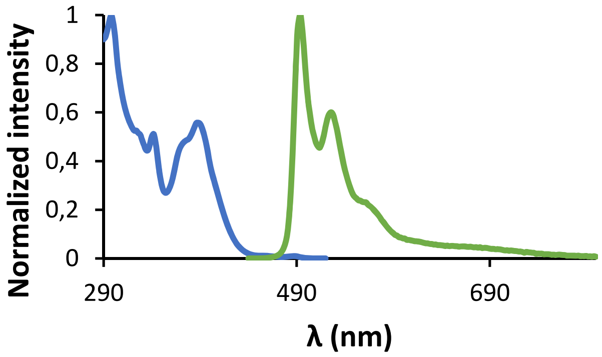

Figure S142. Normalized excitation (blue line; λ_{em} = 560 nm) and emission (green line; λ_{exc} = 420 nm) spectra of **10** in a 1.0 x 10^{-5} M solution in dichloromethane at 298 K.

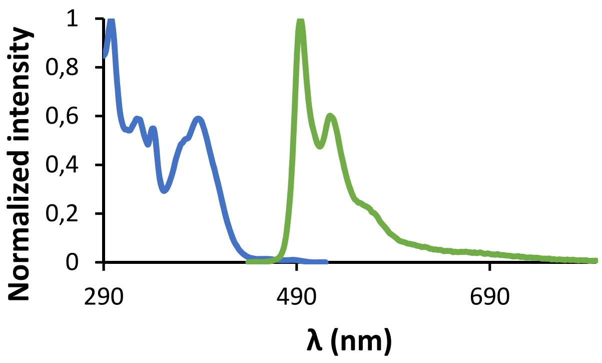

Figure S143. Normalized excitation (blue line; λ_{em} = 560 nm) and emission (green line; λ_{exc} = 420 nm) spectra of **10** in a 1.0 x 10^{-6} M solution in dichloromethane at 298 K.

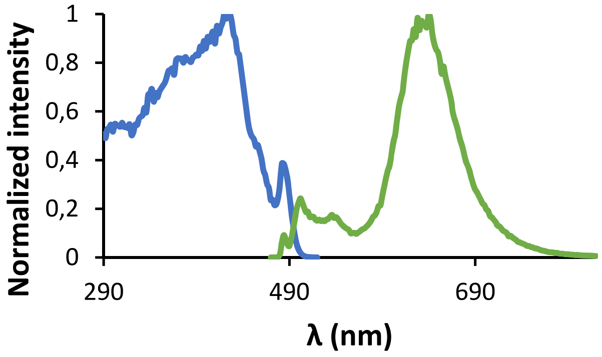

Figure S144. Normalized excitation (blue line; λ_{em} = 560 nm) and emission (green line; λ_{exc} = 420 nm) spectra of **10** in a 1.0 x 10^{-3} M solution in dichloromethane at 77 K.

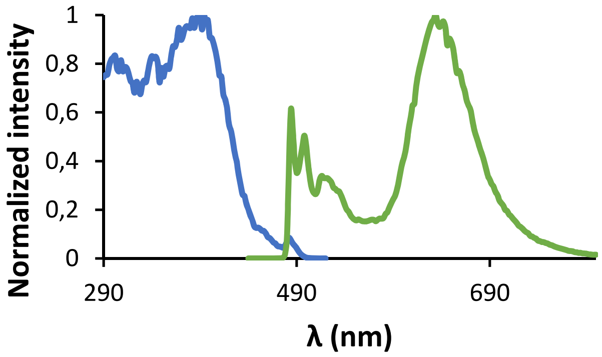

Figure S145. Normalized excitation (blue line; λ_{em} = 560 nm) and emission (green line; λ_{exc} = 420 nm) spectra of **10** in a 1.0 x 10^{-4} M solution in dichloromethane at 77 K.

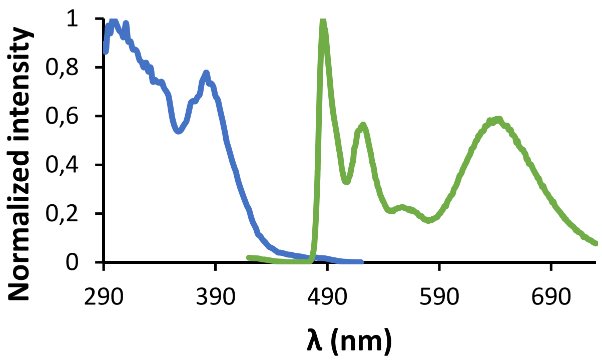

Figure S146. Normalized excitation (blue line; λ_{em} = 560 nm) and emission (green line; λ_{exc} = 420 nm) spectra of **10** in a 1.0 x 10^{-5} M solution in dichloromethane at 77 K.

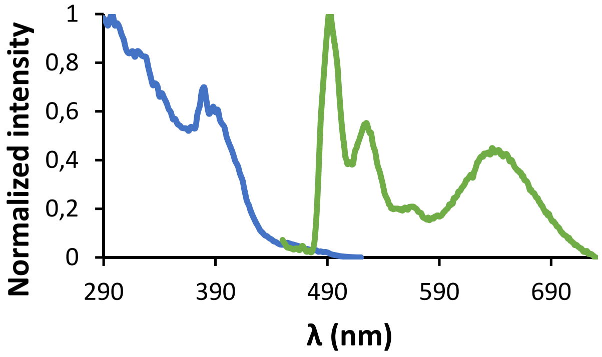

Figure S147. Normalized excitation (blue line; λ_{em} = 560 nm) and emission (green line; λ_{exc} = 420 nm) spectra of **10** in a 1.0 x 10^{-6} M solution in dichloromethane at 77 K.

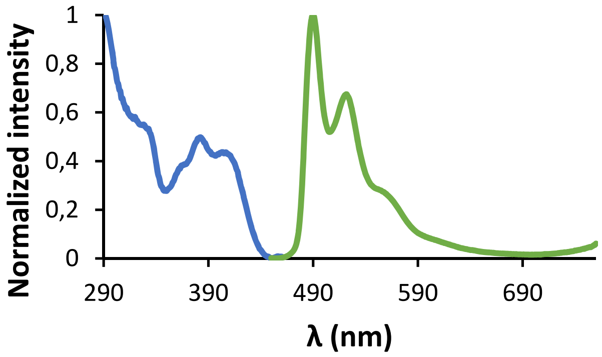

Figure S148. Normalized excitation (blue line; λ_{em} = 500 nm) and emission (green line; λ_{exc} = 400 nm) spectra of **16** in PMMA film (5 wt%) at 298 K.

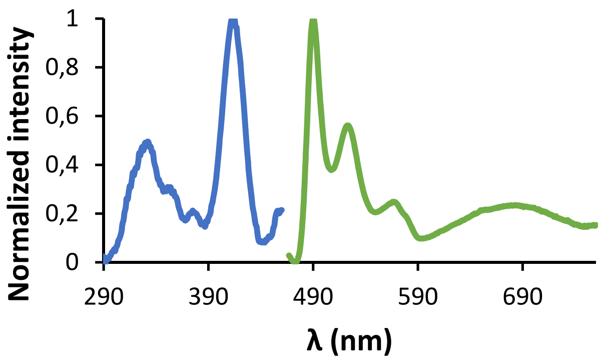

Figure S149. Normalized excitation (blue line; λ_{em} = 500 nm) and emission (green line; λ_{exc} = 400 nm) spectra of **16** in a 1.0 x 10^{-3} M solution in dichloromethane at 298 K.

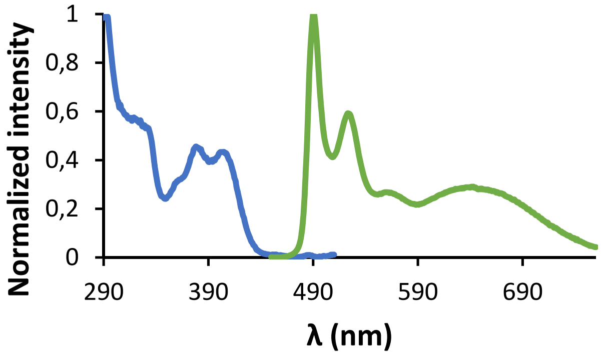

Figure S150. Normalized excitation (blue line; λ_{em} = 550 nm) and emission (green line; λ_{exc} = 400 nm) spectra of **16** in a 1.0 x 10^{-4} M solution in dichloromethane at 298 K.

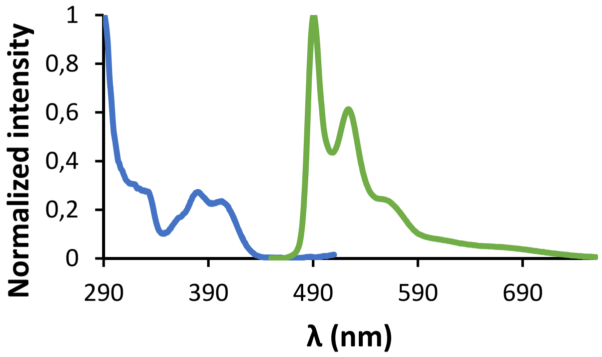

Figure S151. Normalized excitation (blue line; λ_{em} = 550 nm) and emission (green line; λ_{exc} = 400 nm) spectra of **16** in a 1.0 x 10^{-5} M solution in dichloromethane at 298 K.

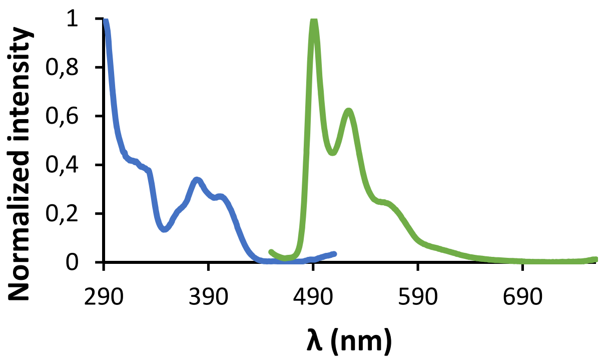

Figure S152. Normalized excitation (blue line; λ_{em} = 550 nm) and emission (green line; λ_{exc} = 400 nm) spectra of **16** in a 1.0 x 10^{-6} M solution in dichloromethane at 298 K.

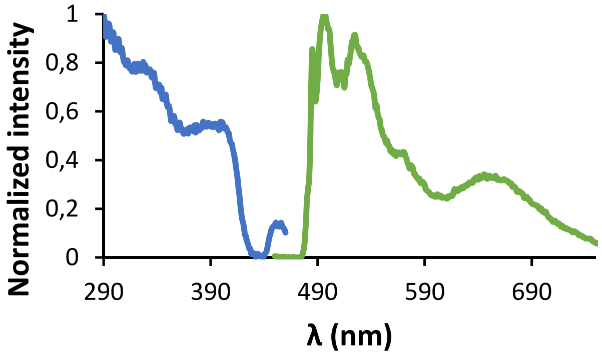

Figure S153. Normalized excitation (blue line; λ_{em} = 500 nm) and emission (green line; λ_{exc} = 400 nm) spectra of **16** in a 1.0 x 10^{-3} M solution in dichloromethane at 77 K.

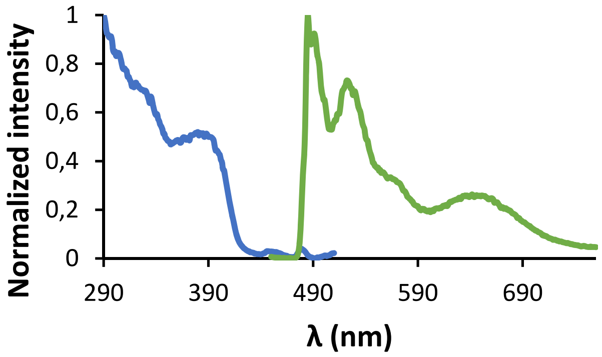

Figure S154. Normalized excitation (blue line; λ_{em} = 550 nm) and emission (green line; λ_{exc} = 400 nm) spectra of **16** in a 1.0 x 10^{-4} M solution in dichloromethane at 77 K.

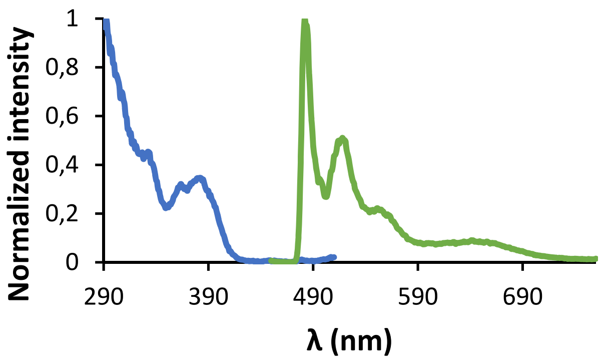

Figure S155. Normalized excitation (blue line; λ_{em} = 550 nm) and emission (green line; λ_{exc} = 400 nm) spectra of **16** in a 1.0 x 10^{-5} M solution in dichloromethane at 77 K.

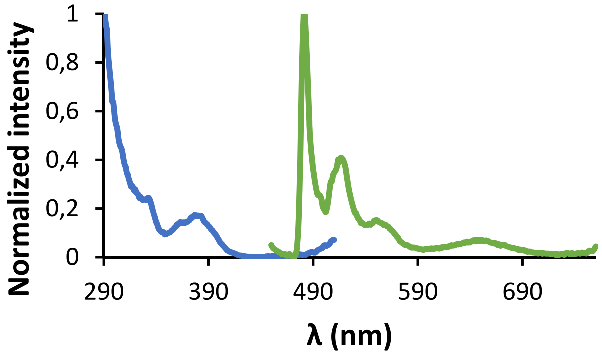

Figure S156. Normalized excitation (blue line; λ_{em} = 550 nm) and emission (green line; λ_{exc} = 400 nm) spectra of **16** in a 1.0 x 10^{-6} M solution in dichloromethane at 77 K.

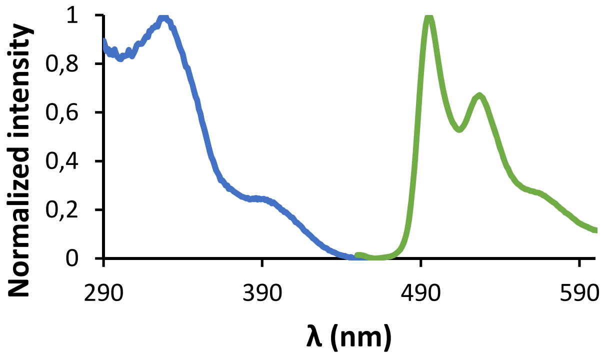

Figure S157. Normalized excitation (blue line; λ_{em} = 550 nm) and emission (green line; λ_{exc} = 350 nm) spectra of **17** in PMMA film (5 wt%) at 298 K.

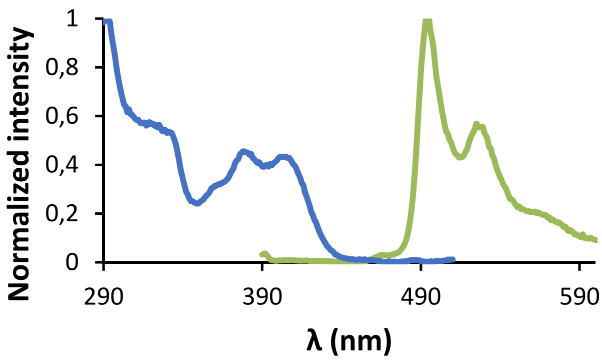

Figure S158. Normalized excitation (blue line; λ_{em} = 550 nm) and emission (green line; λ_{exc} = 350 nm) spectra of **17** in a 1.0 x 10^{-4} M solution in dichloromethane at 298 K.

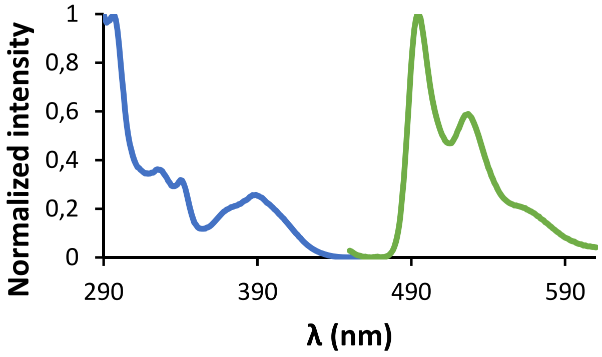

Figure S159. Normalized excitation (blue line; λ_{em} = 550 nm) and emission (green line; λ_{exc} = 350 nm) spectra of **17** in a 1.0 x 10^{-5} M solution in dichloromethane at 298 K.

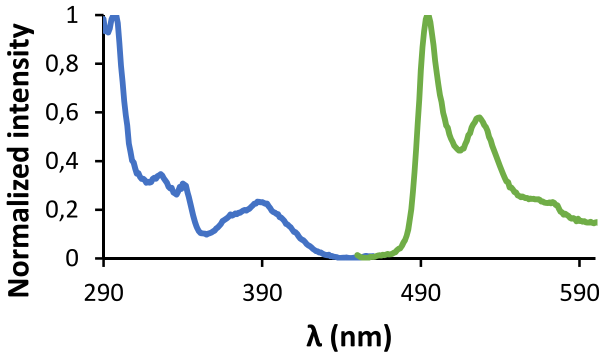

Figure S160. Normalized excitation (blue line; λ_{em} = 550 nm) and emission (green line; λ_{exc} = 350 nm) spectra of **17** in a 1.0 x 10^{-6} M solution in dichloromethane at 298 K.

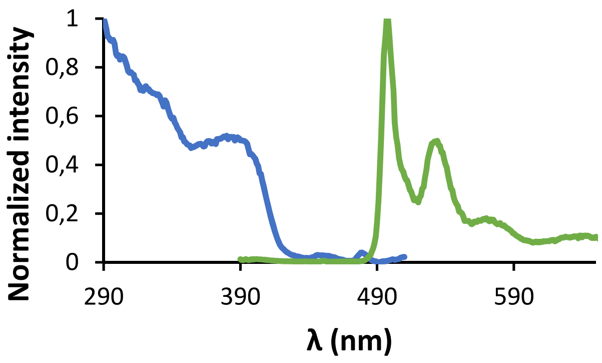

Figure S161. Normalized excitation (blue line; λ_{em} = 550 nm) and emission (green line; λ_{exc} = 350 nm) spectra of **17** in a 1.0 x 10^{-4} M solution in dichloromethane at 77 K.

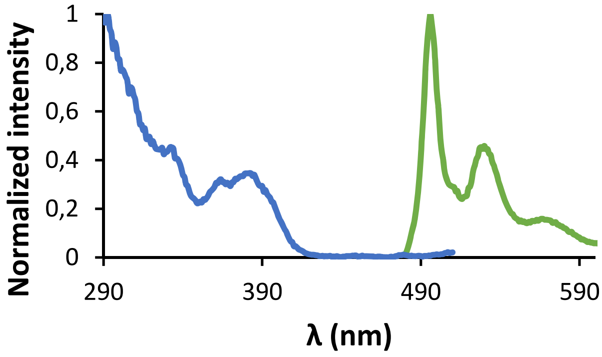

Figure S162. Normalized excitation (blue line; λ_{em} = 550 nm) and emission (green line; λ_{exc} = 350 nm) spectra of **17** in a 1.0 x 10^{-5} M solution in dichloromethane at 77 K.

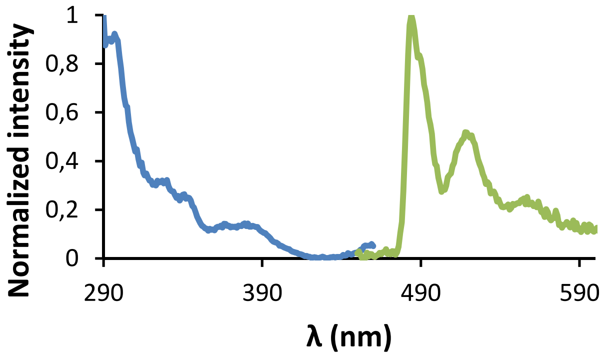

Figure S163. Normalized excitation (blue line; λ_{em} = 550 nm) and emission (green line; λ_{exc} = 350 nm) spectra of **17** in a 1.0 x 10^{-6} M solution in dichloromethane at 77 K.

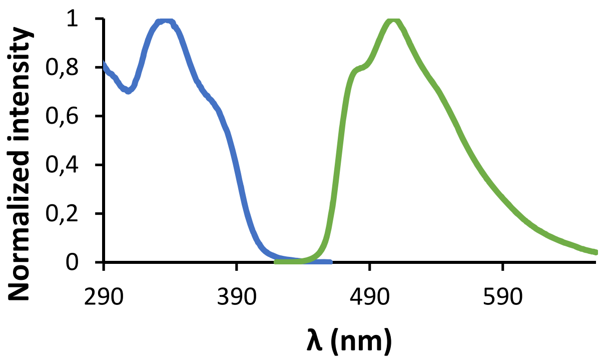

Figure S164. Normalized excitation (blue line; λ_{em} = 500 nm) and emission (green line; λ_{exc} = 350 nm) spectra of **18** in PMMA film (5 wt%) at 298 K.

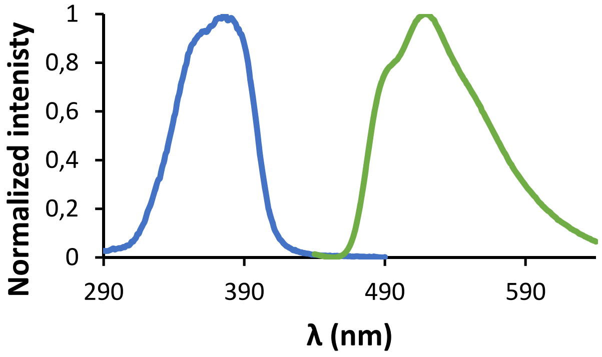

Figure S165. Normalized excitation (blue line; λ_{em} = 500 nm) and emission (green line; λ_{exc} = 350 nm) spectra of **18** in a 1.0 x 10^{-3} M solution in dichloromethane at 298 K.

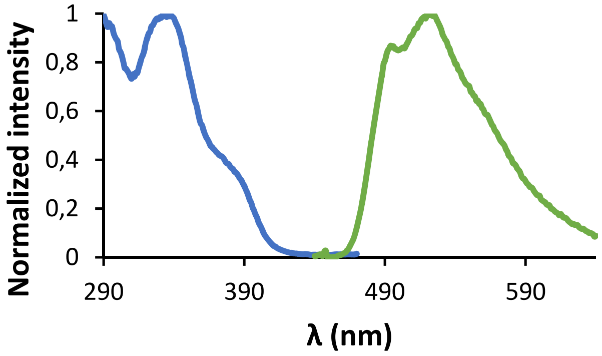

Figure S166. Normalized excitation (blue line; λ_{em} = 500 nm) and emission (green line; λ_{exc} = 350 nm) spectra of **18** in a 1.0 x 10^{-4} M solution in dichloromethane at 298 K.

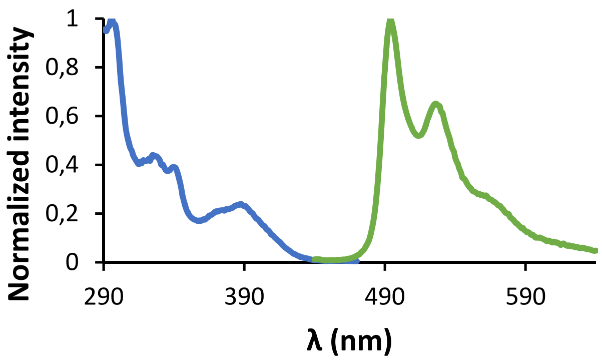

Figure S167. Normalized excitation (blue line; λ_{em} = 500 nm) and emission (green line; λ_{exc} = 350 nm) spectra of **18** in a 1.0 x 10^{-5} M solution in dichloromethane at 298 K.

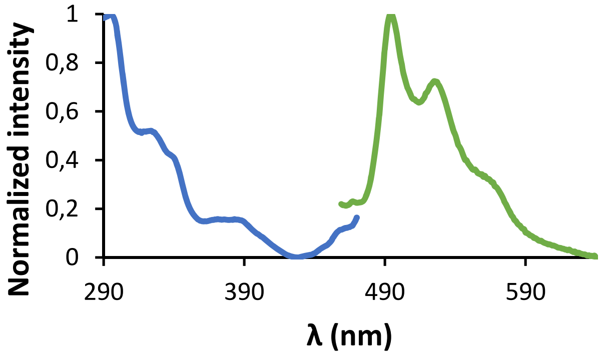

Figure S168. Normalized excitation (blue line; λ_{em} = 500 nm) and emission (green line; λ_{exc} = 350 nm) spectra of **18** in a 1.0 x 10^{-6} M solution in dichloromethane at 298 K.

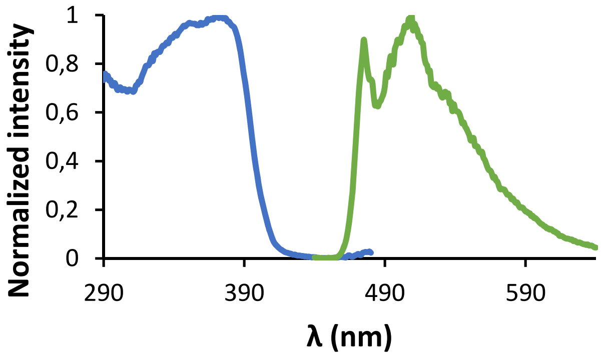

Figure S169. Normalized excitation (blue line; λ_{em} = 500 nm) and emission (green line; λ_{exc} = 350 nm) spectra of **18** in a 1.0 x 10^{-3} M solution in dichloromethane at 77 K.

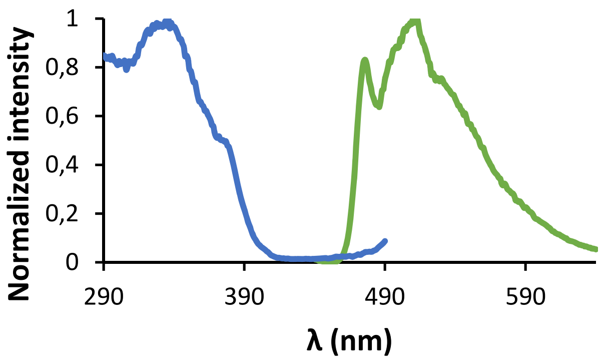

Figure S170. Normalized excitation (blue line; λ_{em} = 500 nm) and emission (green line; λ_{exc} = 350 nm) spectra of **18** in a 1.0 x 10^{-4} M solution in dichloromethane at 77 K.

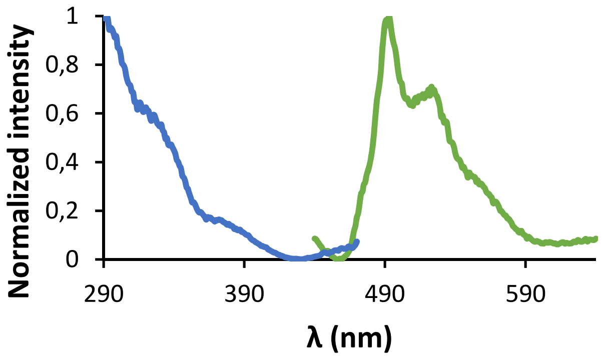

Figure S171. Normalized excitation (blue line; λ_{em} = 500 nm) and emission (green line; λ_{exc} = 350 nm) spectra of **18** in a 1.0 x 10^{-5} M solution in dichloromethane at 77 K.

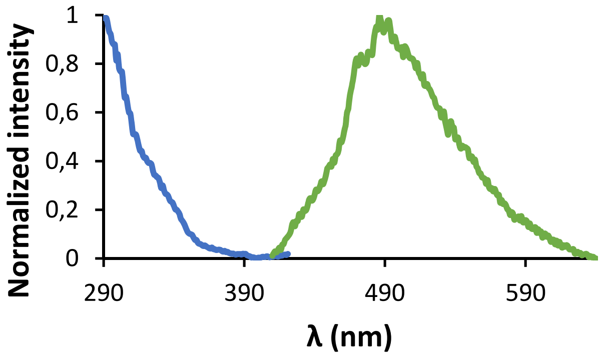

Figure S172. Normalized excitation (blue line; λ_{em} = 500 nm) and emission (green line; λ_{exc} = 350 nm) spectra of **18** in a 1.0 x 10^{-6} M solution in dichloromethane at 77 K.

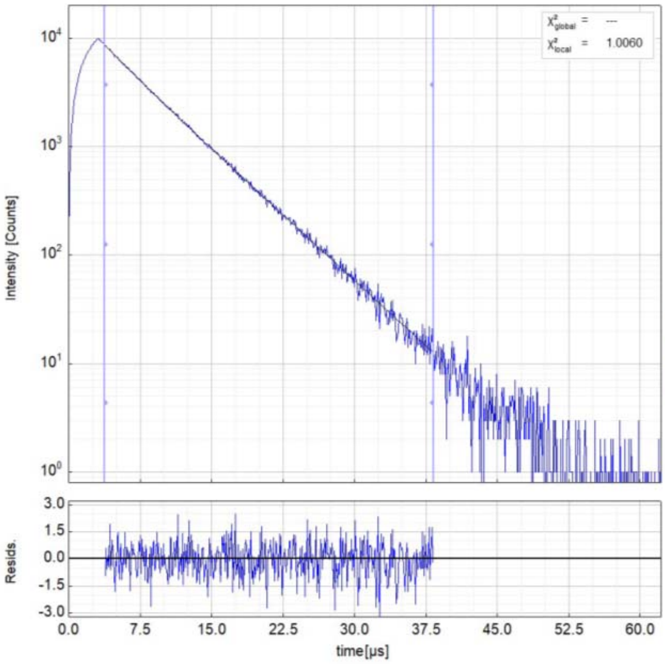

Parameter	Value	Δ
A_1[kCnts/Chnl]	7.42	±0.40
τ_1[ns]	5 392	±89
I_1[kCnts]	500	±19
A_2[kCnts/Chnl]	1.14	±0.44
τ_2[ns]	2 850	±510
I_2[kCnts]	41	±21
$Bkgr_{Dec}$[kCnts]	0.0003	±0.0006
τ_{AvInt}[ns]	5 200.7	±9.8

Figure S173. Left: Raw (experimental) time-resolved photoluminescence decay of **1** in PMMA film (2 wt%) at 298 K (λ_{exc} = 405 nm, λ_{em} = 492 nm). Right: Fitting parameters and confidence limits.

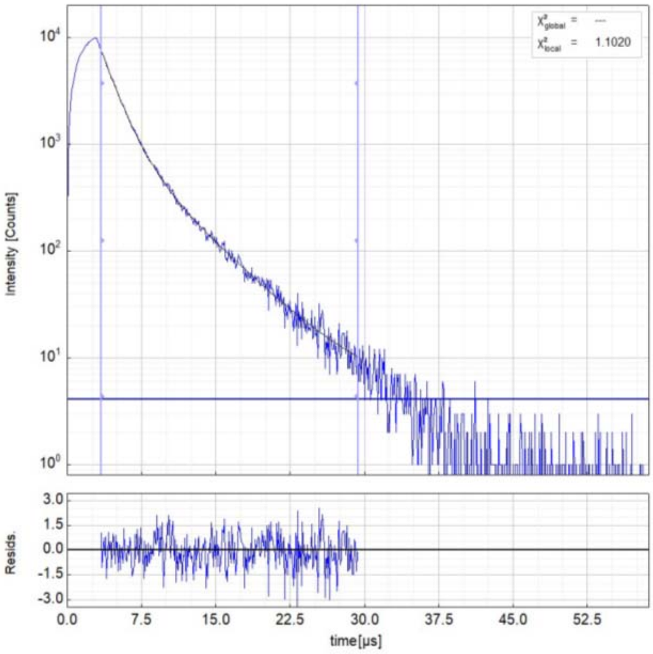

Parameter	Value	Δ
A_1[kCnts/Chnl]	1.217	±0.053
τ_1[ns]	4 890	±110
I_1[kCnts]	74.3	±2.2
A_2[kCnts/Chnl]	6.278	±0.065
τ_2[ns]	1 534	±19
I_2[kCnts]	120.4	±2.2
$Bkgr_{Dec}$[kCnts]	0.0042	±0.0009
τ_{AvInt}[ns]	2 812	±23

Figure S174. Left: Raw (experimental) time-resolved photoluminescence decay of **1** in PMMA film (2 wt%) at 298 K (λ_{exc} = 405 nm, λ_{em} = 674 nm). Right: Fitting parameters and confidence limits.

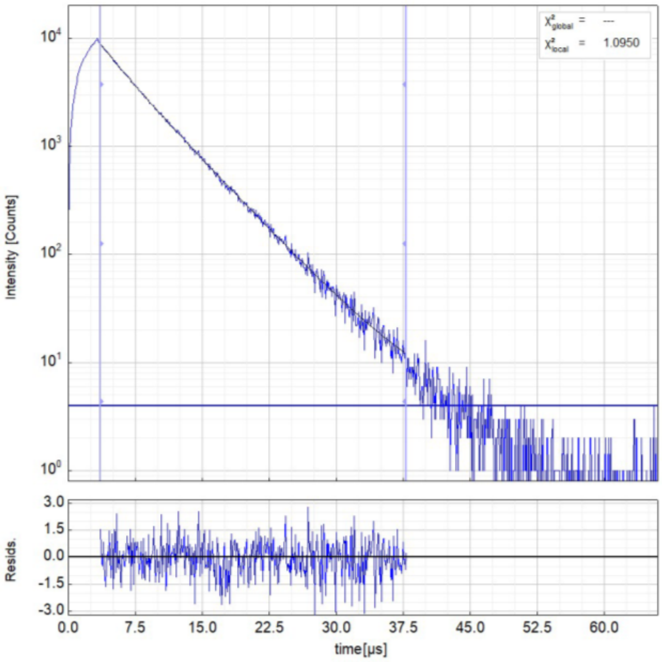

Parameter	Value	Δ
A_1[kCnts/Chnl]	7.01	±0.37
τ_1[ns]	5 061	±70
I_1[kCnts]	444	±17
A_2[kCnts/Chnl]	1.80	±0.33
τ_2[ns]	2 500	±380
I_2[kCnts]	57	±17
$Bkgr_{Dec}$[kCnts]	0.0040	±0.0006
τ_{AvInt}[ns]	4 773	±11

Figure S175. Left: Raw (experimental) time-resolved photoluminescence decay of **1** in PMMA film (5 wt%) at 298 K (λ_{exc} = 405 nm, λ_{em} = 494 nm). Right: Fitting parameters and confidence limits.

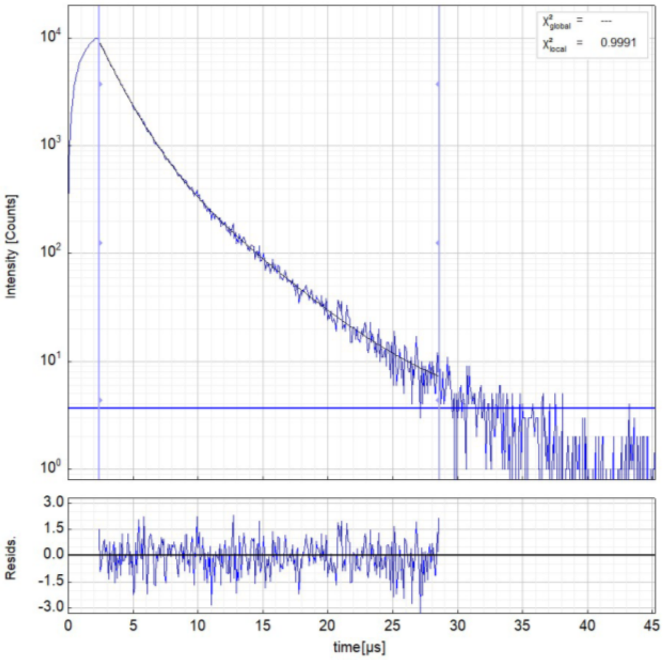

Parameter	Value	Δ
A_1[kCnts/Chnl]	1.517	±0.069
τ_1[ns]	4 332	±78
I_1[kCnts]	82.2	±1.9
A_2[kCnts/Chnl]	7.733	±0.043
τ_2[ns]	1 606	±20
I_2[kCnts]	155.2	±2.1
$Bkgr_{Dec}$[kCnts]	0.0037	±0.0007
τ_{AvInt}[ns]	2 549.2	±8.2

Figure S176. Left: Raw (experimental) time-resolved photoluminescence decay of **1** in PMMA film (5 wt%) at 298 K (λ_{exc} = 405 nm, λ_{em} = 674 nm). Right: Fitting parameters and confidence limits.

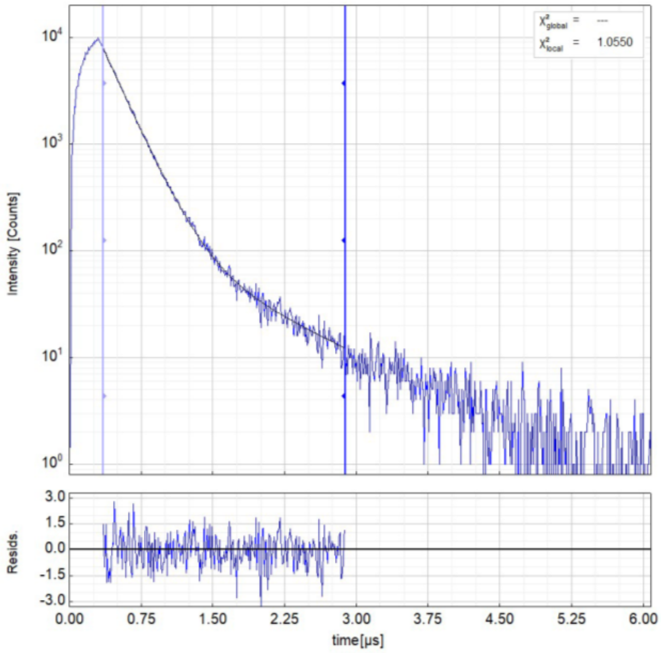

Parameter	Value	Δ
A_1[kCnts/Chnl]	0.153	±0.030
$τ_1$[ns]	1 010	±230
I_1[kCnts]	15.5	±2.4
A_2[kCnts/Chnl]	7.872	±0.035
$τ_2$[ns]	217.3	±1.8
I_2[kCnts]	171.1	±1.4
$Bkgr_{Dec}$[kCnts]	-0.0003	±0.0071
$τ_{AvInt}$[ns]	283	±28

Figure S177. Left: Raw (experimental) time-resolved photoluminescence decay of **1** (10^{-3} M) in DCM at 298 K ($λ_{exc}$ = 405 nm, $λ_{em}$ = 490 nm). Right: Fitting parameters and confidence limits.

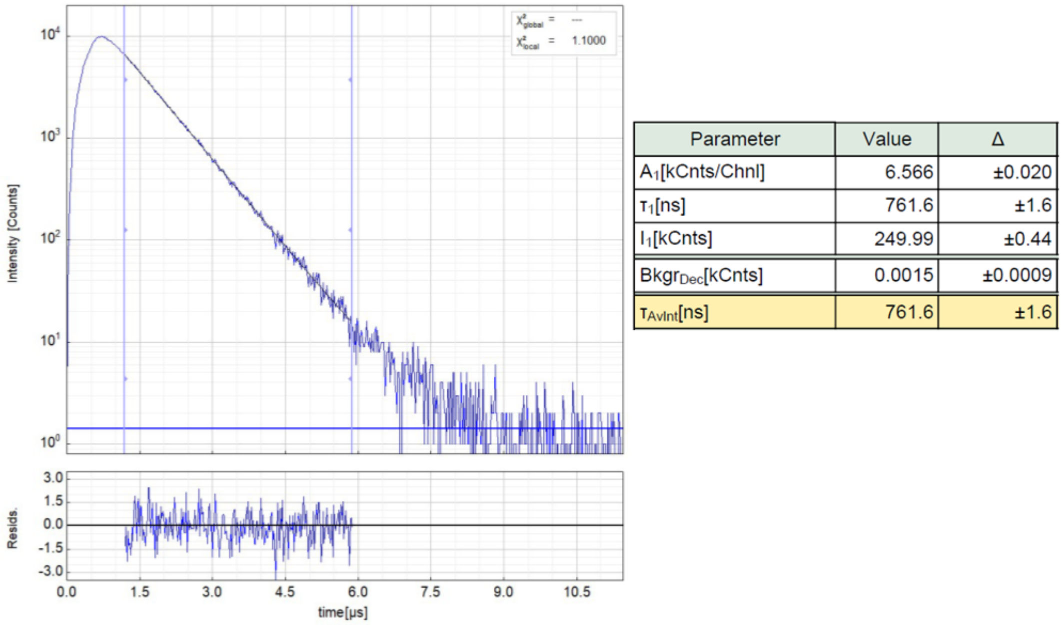

Parameter	Value	Δ
A_1[kCnts/Chnl]	6.566	±0.020
$τ_1$[ns]	761.6	±1.6
I_1[kCnts]	249.99	±0.44
$Bkgr_{Dec}$[kCnts]	0.0015	±0.0009
$τ_{AvInt}$[ns]	761.6	±1.6

Figure S178. Left: Raw (experimental) time-resolved photoluminescence decay of **1** (10^{-3} M) in DCM at 298 K ($λ_{exc}$ = 405 nm, $λ_{em}$ = 690 nm). Right: Fitting parameters and confidence limits.

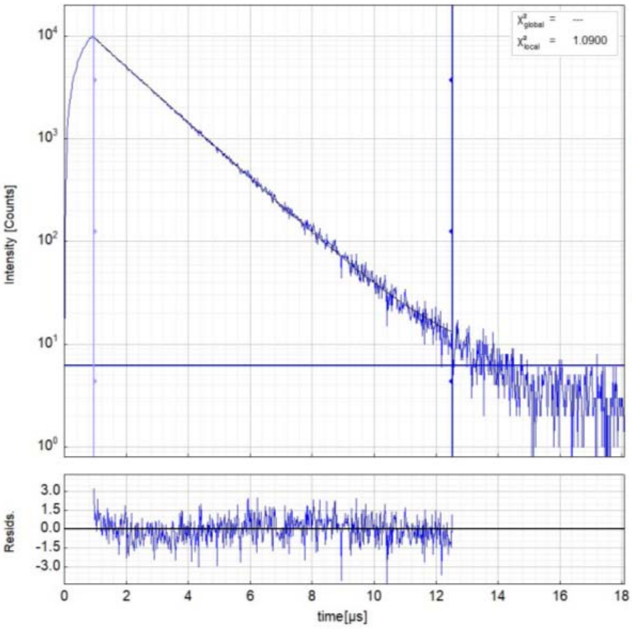

Parameter	Value	Δ
A_1[kCnts/Chnl]	9.583	±0.022
τ_1[ns]	1 598.5	±2.1
I_1[kCnts]	765.91	±0.75
$Bkgr_{Dec}$[kCnts]	0.0062	±0.0006
τ_{AvInt}[ns]	1 598.5	±2.1

Figure S179. Left: Raw (experimental) time-resolved photoluminescence decay of **1** (10^{-4} M) in DCM at 298 K (λ_{exc} = 378 nm, λ_{em} = 490 nm). Right: Fitting parameters and confidence limits.

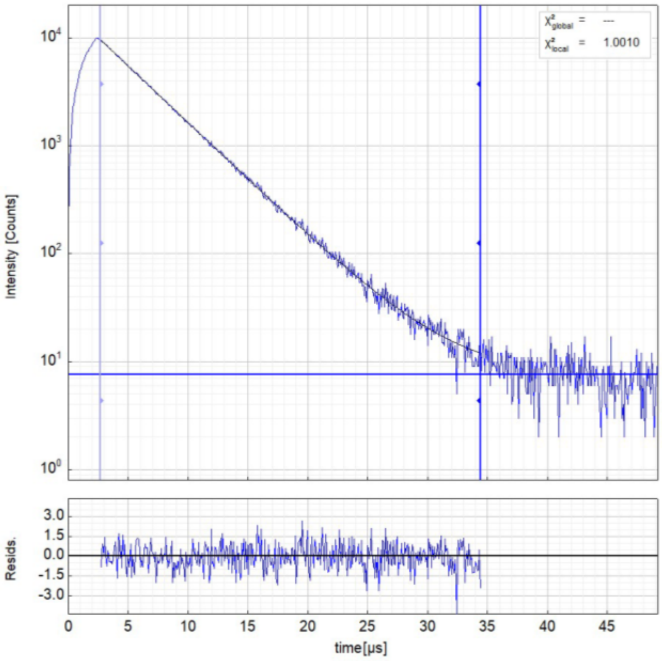

Parameter	Value	Δ
A_1[kCnts/Chnl]	9.475	±0.016
τ_1[ns]	4 129.2	±4.9
I_1[kCnts]	489.1	±1.1
$Bkgr_{Dec}$[kCnts]	0.0077	±0.0005
τ_{AvInt}[ns]	4 129.2	±4.9

Figure S180. Left: Raw (experimental) time-resolved photoluminescence decay of **1** (10^{-5} M) in DCM at 298 K (λ_{exc} = 378 nm, λ_{em} = 490 nm). Right: Fitting parameters and confidence limits.

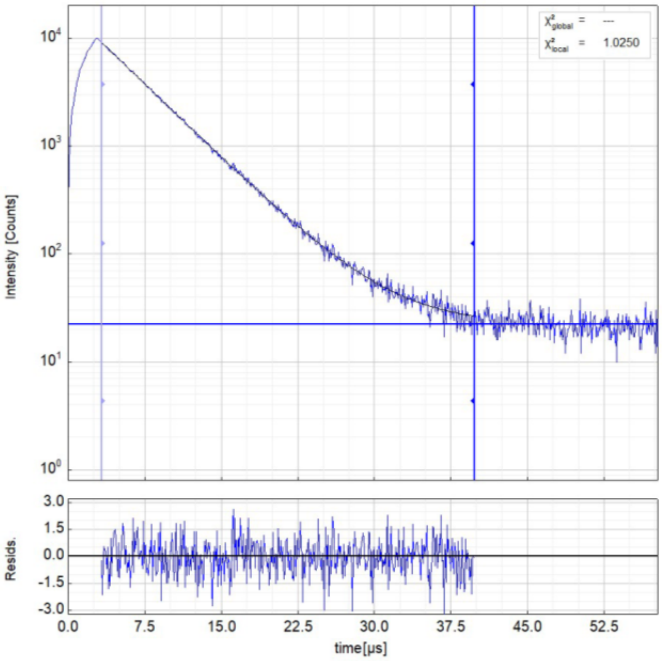

Parameter	Value	Δ
A_1[kCnts/Chnl]	9.055	±0.018
τ_1[ns]	4 754	±11
I_1[kCnts]	537.99	±0.47
$Bkgr_{Dec}$[kCnts]	0.0224	±0.0007
τ_{AvInt}[ns]	4 754	±11

Figure S181. Left: Raw (experimental) time-resolved photoluminescence decay of **1** (10^{-6} M) in DCM at 298 K (λ_{exc} = 378 nm, λ_{em} = 490 nm). Right: Fitting parameters and confidence limits.

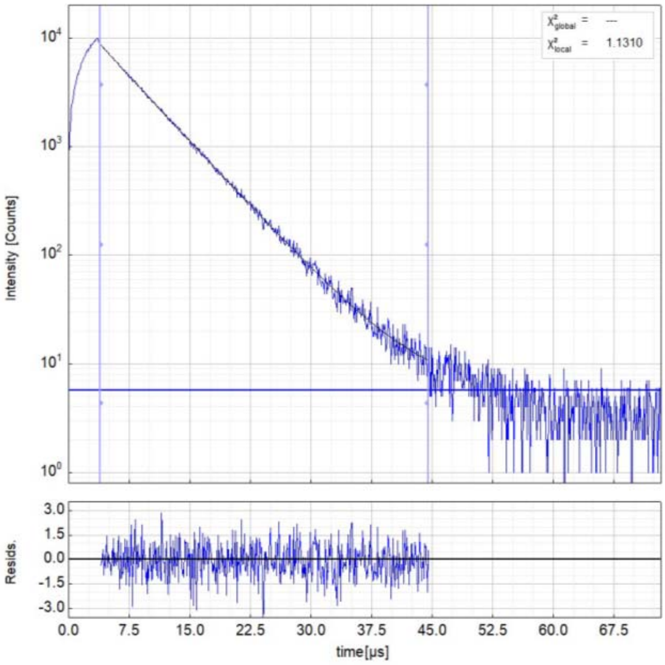

Parameter	Value	Δ
A_1[kCnts/Chnl]	8.33	±0.24
τ_1[ns]	5 475	±47
I_1[kCnts]	570	±12
A_2[kCnts/Chnl]	0.29	±0.24
τ_2[ns]	2 200	±1 300
I_2[kCnts]	8	±12
$Bkgr_{Dec}$[kCnts]	0.0058	±0.0008
τ_{AvInt}[ns]	5 430	±13

Figure S182. Left: Raw (experimental) time-resolved photoluminescence decay of **1** (10^{-3} M) in DCM at 77 K (λ_{exc} = 405 nm, λ_{em} = 490 nm). Right: Fitting parameters and confidence limits.

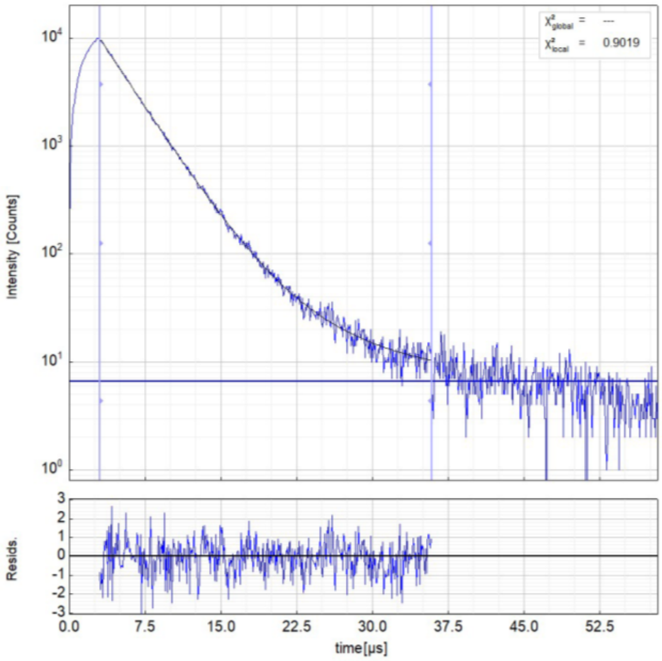

Parameter	Value	Δ
A_1[kCnts/Chnl]	0.178	±0.077
τ_1[ns]	8 400	±2 800
I_1[kCnts]	18.6	±4.0
A_2[kCnts/Chnl]	9.640	±0.094
τ_2[ns]	3 016	±30
I_2[kCnts]	363.4	±5.1
$Bkgr_{Dec}$[kCnts]	0.0067	±0.0027
τ_{AvInt}[ns]	3 277	±64

Figure S183. Left: Raw (experimental) time-resolved photoluminescence decay of **1** (10^{-3} M) in DCM at 77 K (λ_{exc} = 405 nm, λ_{em} = 668 nm). Right: Fitting parameters and confidence limits.

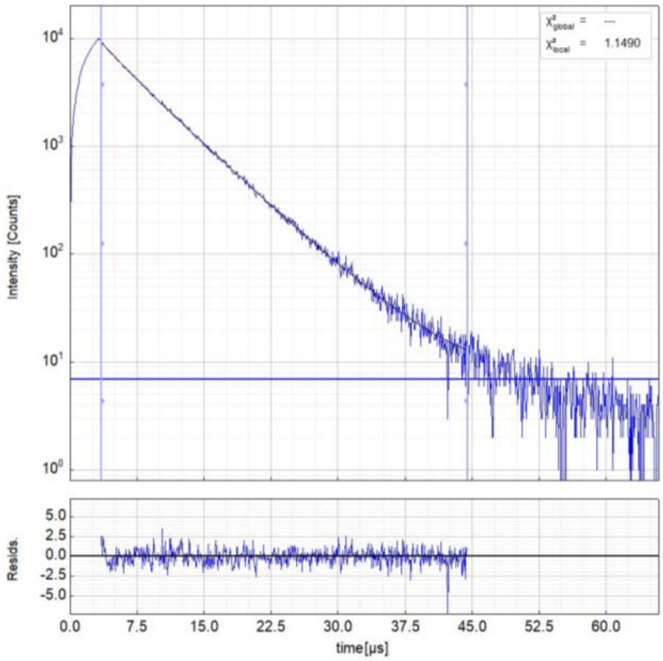

Parameter	Value	Δ
A_1[kCnts/Chnl]	7.62	±0.64
τ_1[ns]	5 730	±150
I_1[kCnts]	545	±31
A_2[kCnts/Chnl]	1.48	±0.51
τ_2[ns]	2 800	±1 200
I_2[kCnts]	51	±32
$Bkgr_{Dec}$[kCnts]	0.0069	±0.0014
τ_{AvInt}[ns]	5 475	±28

Figure S184. Left: Raw (experimental) time-resolved photoluminescence decay of **1** (10^{-4} M) in DCM at 77 K (λ_{exc} = 378 nm, λ_{em} = 488 nm). Right: Fitting parameters and confidence limits.

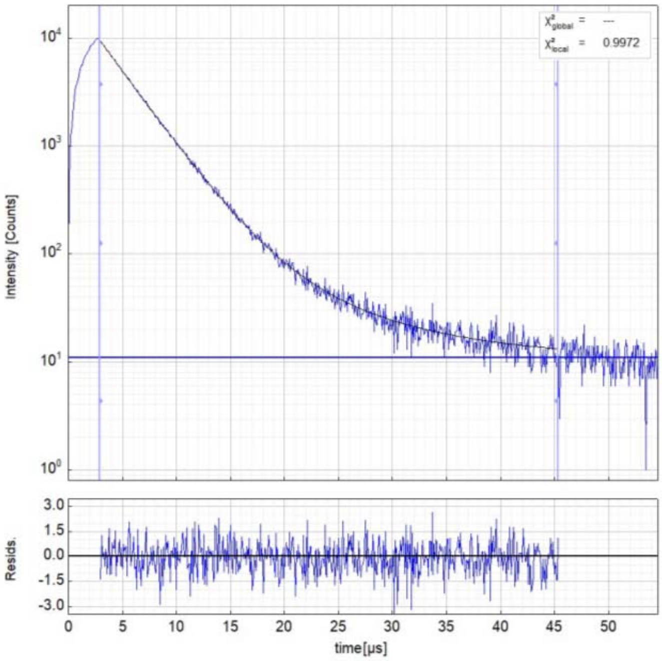

Parameter	Value	Δ
A_1[kCnts/Chnl]	0.215	±0.026
τ_1[ns]	9 310	±610
I_1[kCnts]	25.0	±1.3
A_2[kCnts/Chnl]	9.193	±0.043
τ_2[ns]	3 109.4	±6.4
I_2[kCnts]	357.3	±1.5
$Bkgr_{Dec}$[kCnts]	0.0110	±0.0011
τ_{AvInt}[ns]	3 514	±31

Figure S185. Left: Raw (experimental) time-resolved photoluminescence decay of **1** (10^{-4} M) in DCM at 77 K (λ_{exc} = 378 nm, λ_{em} = 662 nm). Right: Fitting parameters and confidence limits.

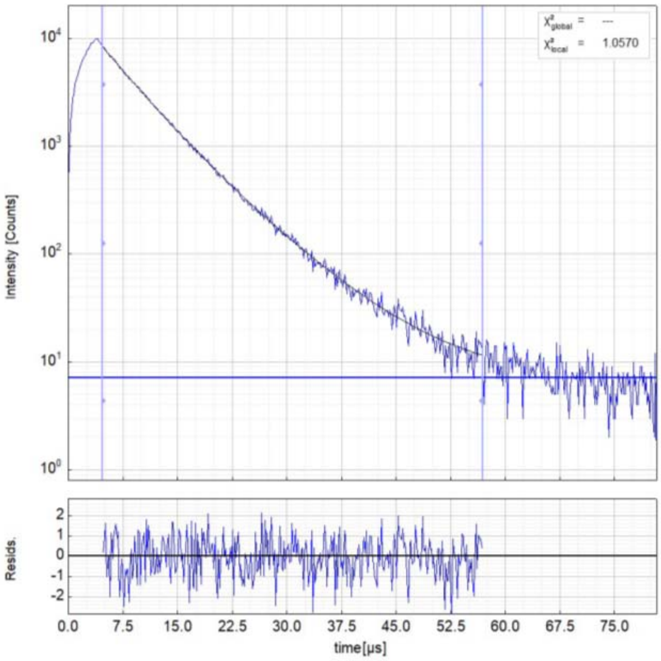

Parameter	Value	Δ
A_1[kCnts/Chnl]	2.21	±0.87
τ_1[ns]	8 340	±580
I_1[kCnts]	115	±34
A_2[kCnts/Chnl]	6.01	±0.89
τ_2[ns]	4 820	±260
I_2[kCnts]	181	±35
$Bkgr_{Dec}$[kCnts]	0.0072	±0.0015
τ_{AvInt}[ns]	6 184	±48

Figure S186. Left: Raw (experimental) time-resolved photoluminescence decay of **1** (10^{-5} M) in DCM at 77 K (λ_{exc} = 378 nm, λ_{em} = 490 nm). Right: Fitting parameters and confidence limits.

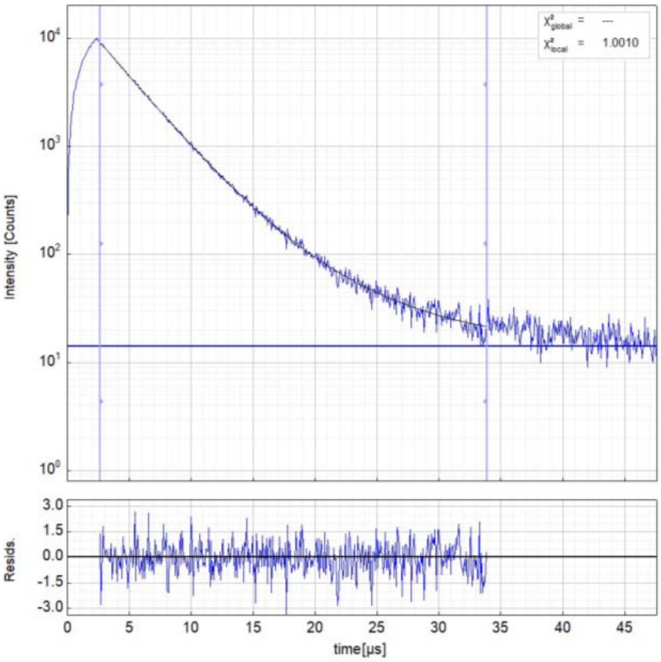

Parameter	Value	Δ
A_1[kCnts/Chnl]	0.47	±0.39
τ_1[ns]	7 500	±1 600
I_1[kCnts]	44	±22
A_2[kCnts/Chnl]	8.66	±0.36
τ_2[ns]	3 153	±83
I_2[kCnts]	342	±23
$Bkgr_{Dec}$[kCnts]	0.0141	±0.0034
τ_{AvInt}[ns]	3 633	±72

Figure S187. Left: Raw (experimental) time-resolved photoluminescence decay of **1** (10^{-5} M) in DCM at 77 K (λ_{exc} = 378 nm, λ_{em} = 656 nm). Right: Fitting parameters and confidence limits.

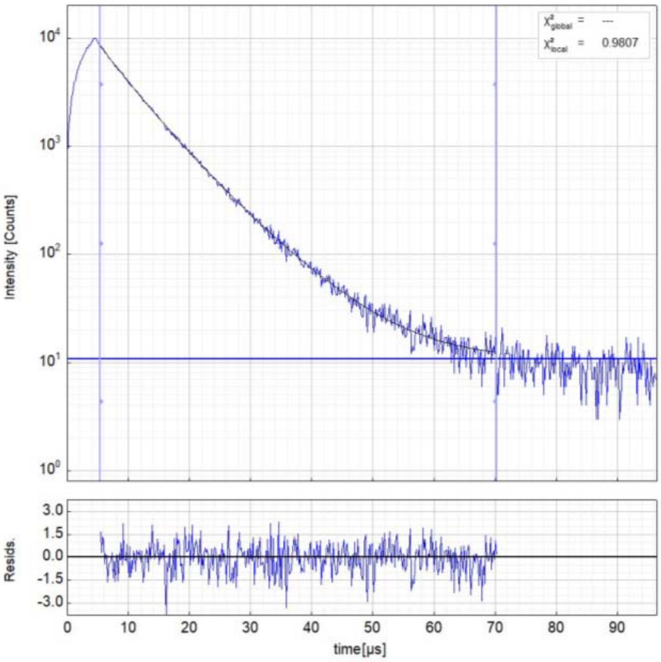

Parameter	Value	Δ
A_1[kCnts/Chnl]	3.35	±0.80
τ_1[ns]	8 550	±440
I_1[kCnts]	179	±35
A_2[kCnts/Chnl]	5.01	±0.82
τ_2[ns]	4 910	±300
I_2[kCnts]	154	±36
$Bkgr_{Dec}$[kCnts]	0.0108	±0.0007
τ_{AvInt}[ns]	6 864	±26

Figure S188. Left: Raw (experimental) time-resolved photoluminescence decay of **1** (10^{-6} M) in DCM at 77 K (λ_{exc} = 378 nm, λ_{em} = 490 nm). Right: Fitting parameters and confidence limits.

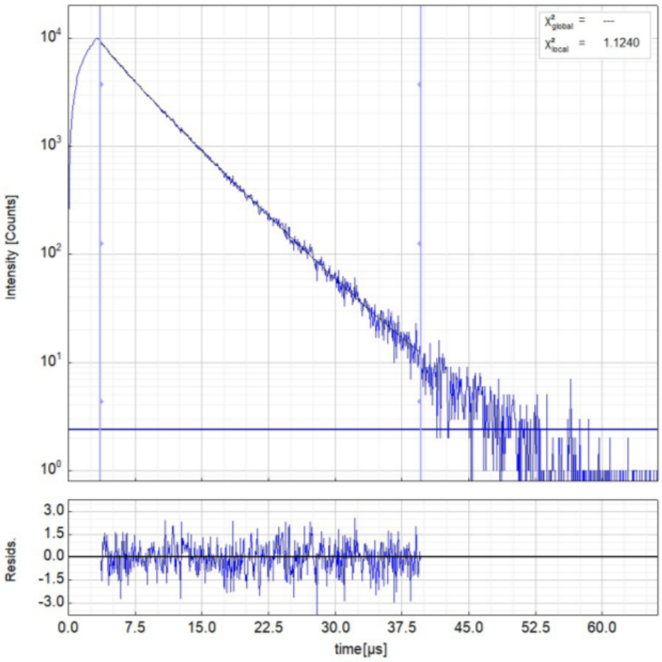

Parameter	Value	Δ
A_1[kCnts/Chnl]	6.86	±0.28
τ_1[ns]	5 492	±62
I_1[kCnts]	471	±13
A_2[kCnts/Chnl]	2.19	±0.26
τ_2[ns]	2 720	±210
I_2[kCnts]	74	±13
$Bkgr_{Dec}$[kCnts]	0.0024	±0.0010
τ_{AvInt}[ns]	5 115	±19

Figure S189. Left: Raw (experimental) time-resolved photoluminescence decay of **2** in PMMA film (2 wt%) at 298 K (λ_{exc} = 405 nm, λ_{em} = 500 nm). Right: Fitting parameters and confidence limits.

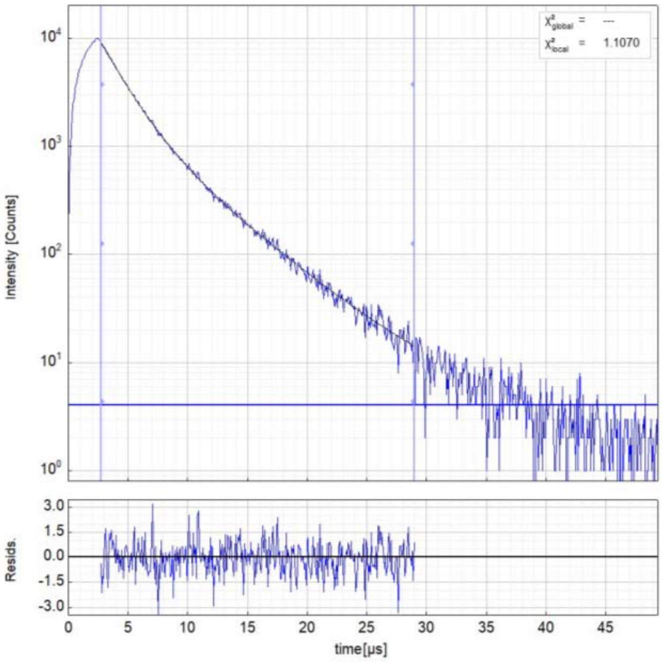

Parameter	Value	Δ
A_1[kCnts/Chnl]	2.08	±0.14
τ_1[ns]	4 940	±120
I_1[kCnts]	128.1	±5.5
A_2[kCnts/Chnl]	7.17	±0.12
τ_2[ns]	1 918	±33
I_2[kCnts]	171.9	±5.9
$Bkgr_{Dec}$[kCnts]	0.0040	±0.0016
τ_{AvInt}[ns]	3 208	±26

Figure S190. Left: Raw (experimental) time-resolved photoluminescence decay of **2** in PMMA film (2 wt%) at 298 K (λ_{exc} = 405 nm, λ_{em} = 678 nm). Right: Fitting parameters and confidence limits.

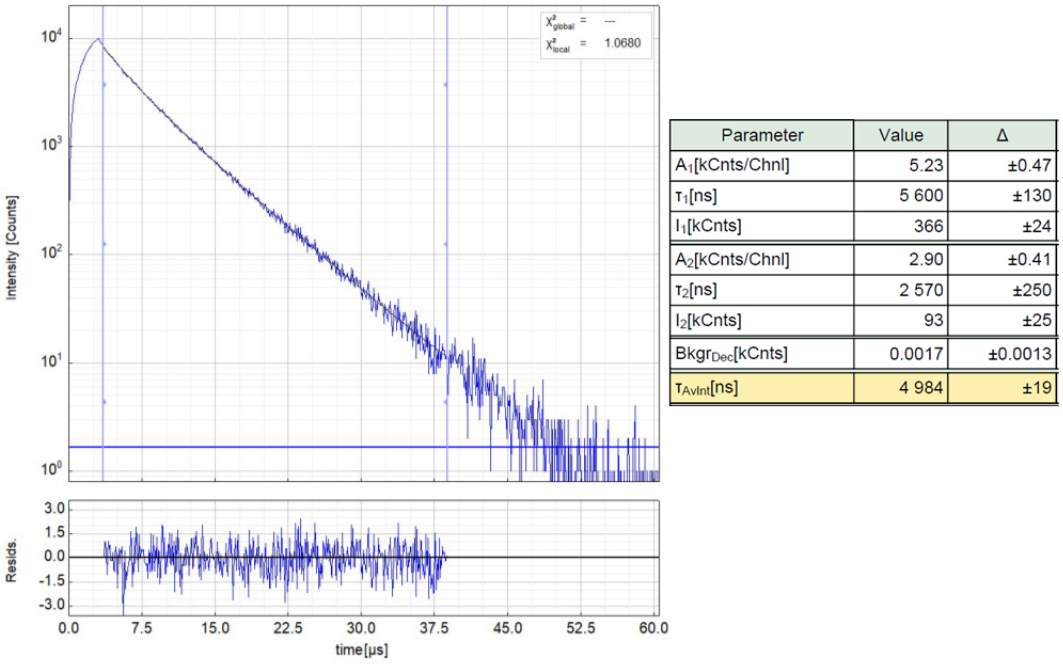

Parameter	Value	Δ
A_1[kCnts/Chnl]	5.23	±0.47
τ_1[ns]	5 600	±130
I_1[kCnts]	366	±24
A_2[kCnts/Chnl]	2.90	±0.41
τ_2[ns]	2 570	±250
I_2[kCnts]	93	±25
$Bkgr_{Dec}$[kCnts]	0.0017	±0.0013
τ_{AvInt}[ns]	4 984	±19

Figure S191. Left: Raw (experimental) time-resolved photoluminescence decay of **2** in PMMA film (5 wt%) at 298 K (λ_{exc} = 405 nm, λ_{em} = 504 nm). Right: Fitting parameters and confidence limits.

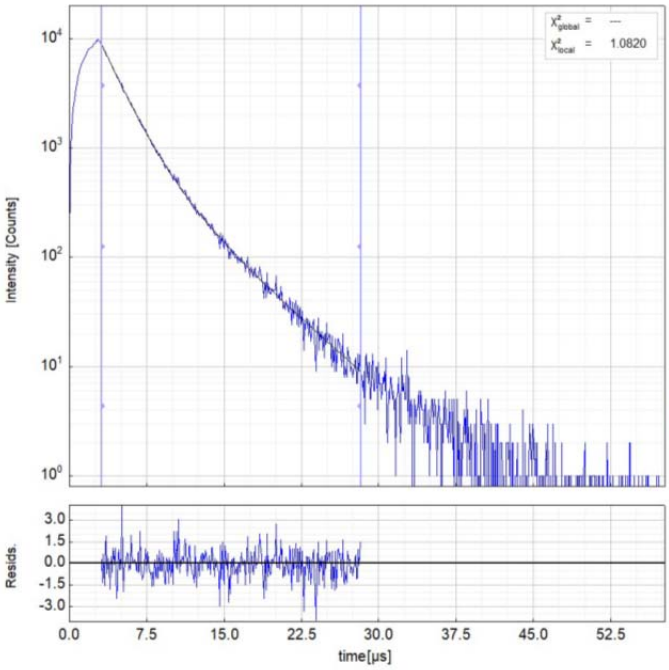

Parameter	Value	Δ
A_1[kCnts/Chnl]	1.21	±0.12
τ_1[ns]	5 120	±190
I_1[kCnts]	76.7	±4.4
A_2[kCnts/Chnl]	7.76	±0.15
τ_2[ns]	1 984	±24
I_2[kCnts]	192.2	±5.1
$Bkgr_{Dec}$[kCnts]	0.0003	±0.0013
τ_{AvInt}[ns]	2 876	±18

Figure S192. Left: Raw (experimental) time-resolved photoluminescence decay of **2** in PMMA film (5 wt%) at 298 K (λ_{exc} = 405 nm, λ_{em} = 670 nm). Right: Fitting parameters and confidence limits.

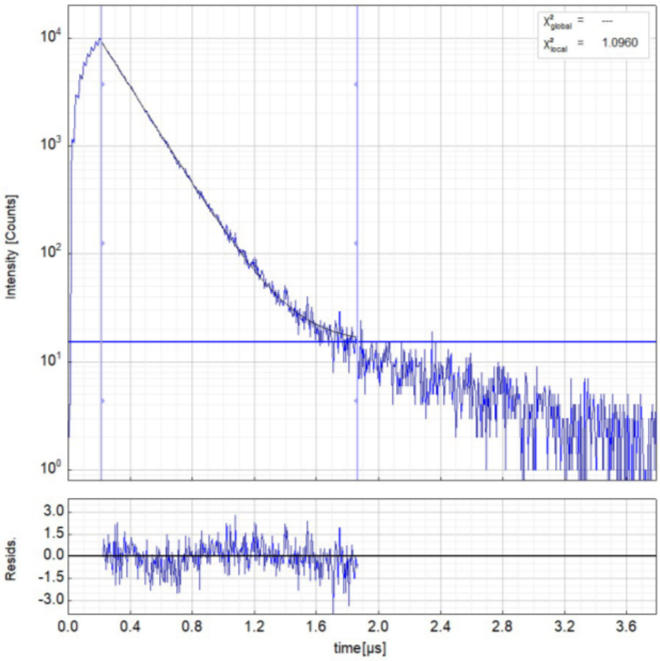

Parameter	Value	Δ
A_1[kCnts/Chnl]	8.978	±0.021
τ_1[ns]	192.59	±0.35
I_1[kCnts]	345.79	±0.45
$Bkgr_{Dec}$[kCnts]	0.0152	±0.0008
τ_{AvInt}[ns]	192.59	±0.35

Figure S193. Left: Raw (experimental) time-resolved photoluminescence decay of **2** (10^{-3} M) in DCM at 298 K (λ_{exc} = 405 nm, λ_{em} = 496 nm). Right: Fitting parameters and confidence limits.

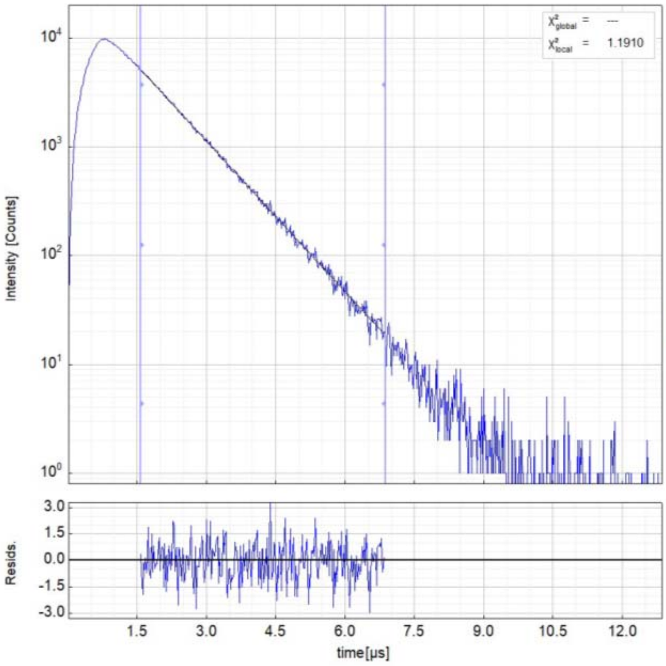

Parameter	Value	Δ
A_1[kCnts/Chnl]	5.123	±0.016
τ_1[ns]	939.4	±2.0
I_1[kCnts]	240.61	±0.40
$Bkgr_{Dec}$[kCnts]	0.0004	±0.0005
τ_{AvInt}[ns]	939.4	±2.0

Figure S194. Left: Raw (experimental) time-resolved photoluminescence decay of **2** (10^{-3} M) in DCM at 298 K (λ_{exc} = 405 nm, λ_{em} = 692 nm). Right: Fitting parameters and confidence limits.

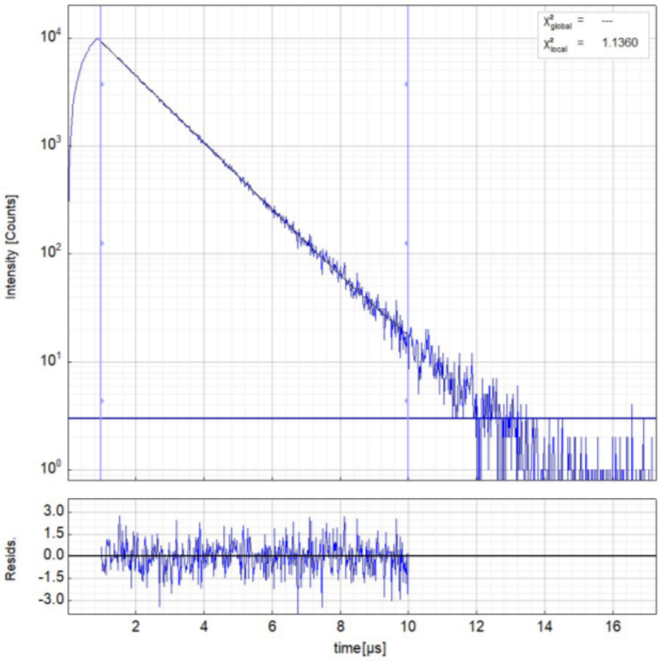

Parameter	Value	Δ
A_1[kCnts/Chnl]	9.161	±0.017
τ_1[ns]	1 396.1	±2.4
I_1[kCnts]	639.43	±0.92
$Bkgr_{Dec}$[kCnts]	0.0030	±0.0007
τ_{AvInt}[ns]	1 396.1	±2.4

Figure S195. Left: Raw (experimental) time-resolved photoluminescence decay of **2** (10^{-4} M) in DCM at 298 K (λ_{exc} = 378 nm, λ_{em} = 494 nm). Right: Fitting parameters and confidence limits.

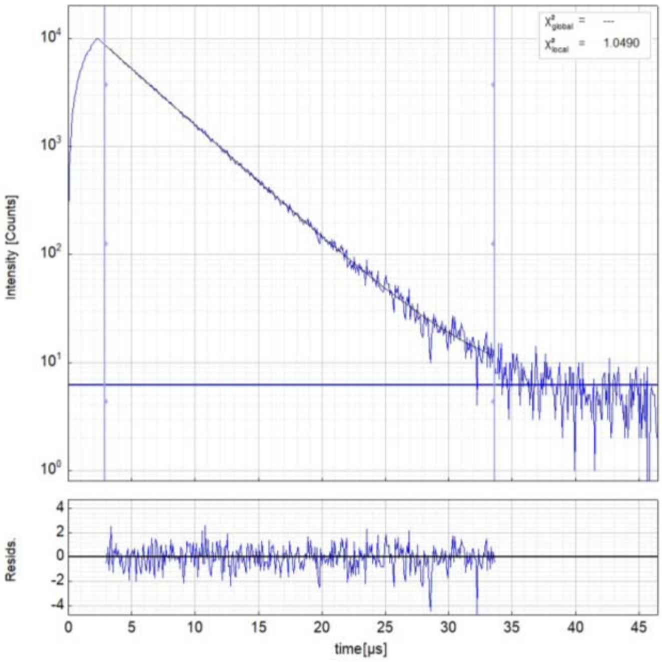

Parameter	Value	Δ
A_1[kCnts/Chnl]	8.500	±0.017
τ_1[ns]	4 153.6	±6.2
I_1[kCnts]	441.30	±0.41
$Bkgr_{Dec}$[kCnts]	0.0062	±0.0004
τ_{AvInt}[ns]	4 153.6	±6.2

Figure S196. Left: Raw (experimental) time-resolved photoluminescence decay of **2** (10^{-5} M) in DCM at 298 K (λ_{exc} = 378 nm, λ_{em} = 494 nm). Right: Fitting parameters and confidence limits.

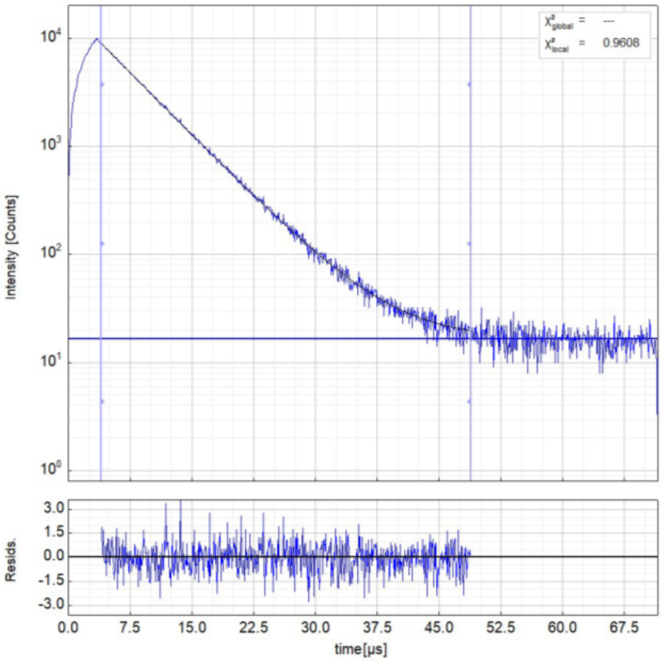

Parameter	Value	Δ
A_1[kCnts/Chnl]	8.739	±0.014
τ_1[ns]	5 649	±11
I_1[kCnts]	617.0	±1.2
$Bkgr_{Dec}$[kCnts]	0.0165	±0.0003
τ_{AvInt}[ns]	5 649	±11

Figure S197. Left: Raw (experimental) time-resolved photoluminescence decay of **2** (10^{-6} M) in DCM at 298 K (λ_{exc} = 378 nm, λ_{em} = 494 nm). Right: Fitting parameters and confidence limits.

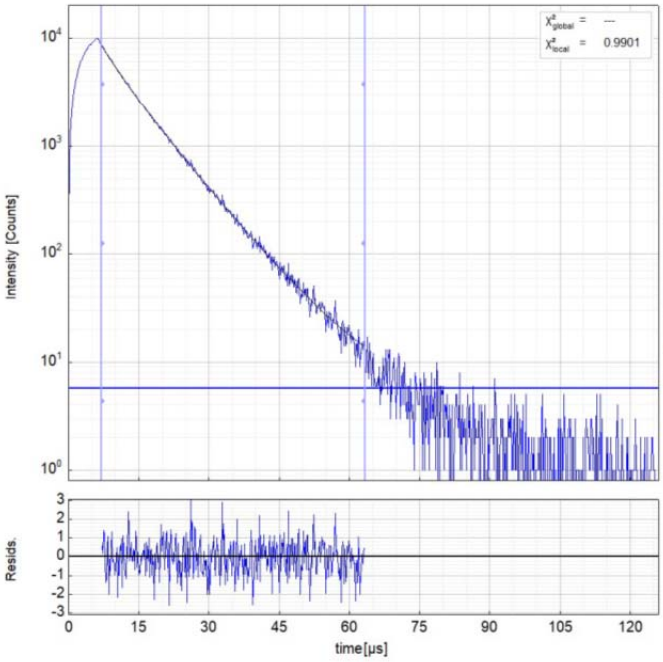

Parameter	Value	Δ
A_1[kCnts/Chnl]	5.57	±0.26
τ_1[ns]	8 610	±150
I_1[kCnts]	300	±11
A_2[kCnts/Chnl]	2.80	±0.28
τ_2[ns]	4 090	±280
I_2[kCnts]	72	±11
$Bkgr_{Dec}$[kCnts]	0.0058	±0.0009
τ_{AvInt}[ns]	7 731	±22

Figure S198. Left: Raw (experimental) time-resolved photoluminescence decay of **2** (10^{-3} M) in DCM at 77 K (λ_{exc} = 405 nm, λ_{em} = 506 nm). Right: Fitting parameters and confidence limits.

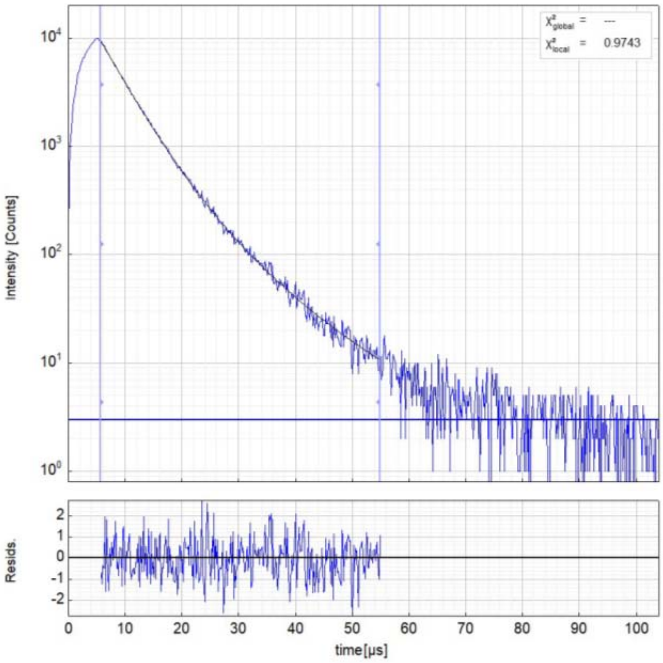

Parameter	Value	Δ
A_1[kCnts/Chnl]	1.03	±0.21
τ_1[ns]	10 010	±810
I_1[kCnts]	64.4	±8.5
A_2[kCnts/Chnl]	8.30	±0.20
τ_2[ns]	4 456	±74
I_2[kCnts]	231.1	±9.4
$Bkgr_{Dec}$[kCnts]	0.0030	±0.0015
τ_{AvInt}[ns]	5 666	±54

Figure S199. Left: Raw (experimental) time-resolved photoluminescence decay of **2** (10^{-3} M) in DCM at 77 K (λ_{exc} = 405 nm, λ_{em} = 656 nm). Right: Fitting parameters and confidence limits.

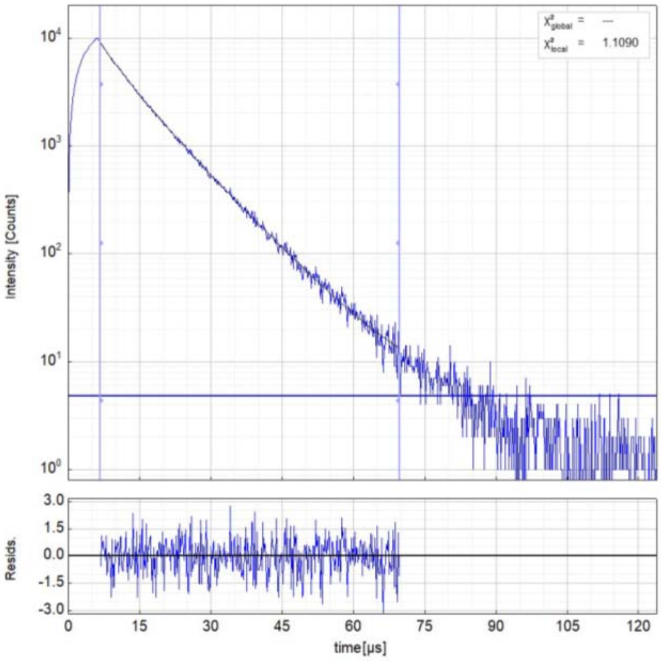

Parameter	Value	Δ
A_1[kCnts/Chnl]	5.66	±0.36
τ_1[ns]	9 660	±200
I_1[kCnts]	342	±14
A_2[kCnts/Chnl]	3.23	±0.33
τ_2[ns]	4 470	±250
I_2[kCnts]	91	±14
$Bkgr_{Dec}$[kCnts]	0.0048	±0.0010
τ_{AvInt}[ns]	8 573	±26

Figure S200. Left: Raw (experimental) time-resolved photoluminescence decay of **2** (10^{-4} M) in DCM at 77 K (λ_{exc} = 405 nm, λ_{em} = 506 nm). Right: Fitting parameters and confidence limits.

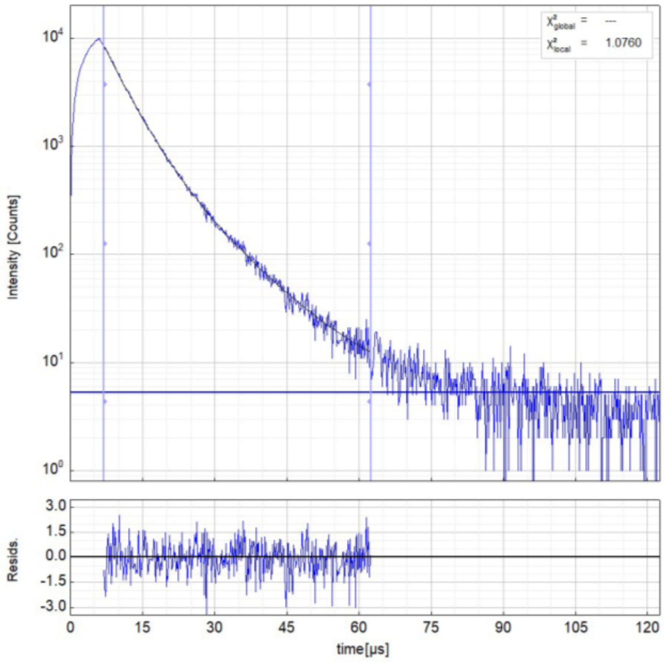

Parameter	Value	Δ
A_1[kCnts/Chnl]	1.41	±0.18
τ_1[ns]	10 490	±480
I_1[kCnts]	92.1	±6.7
A_2[kCnts/Chnl]	6.98	±0.15
τ_2[ns]	4 464	±79
I_2[kCnts]	194.6	±7.2
$Bkgr_{Dec}$[kCnts]	0.0053	±0.0017
τ_{AvInt}[ns]	6 396	±75

Figure S201. Left: Raw (experimental) time-resolved photoluminescence decay of **2** (10^{-4} M) in DCM at 77 K (λ_{exc} = 405 nm, λ_{em} = 660 nm). Right: Fitting parameters and confidence limits.

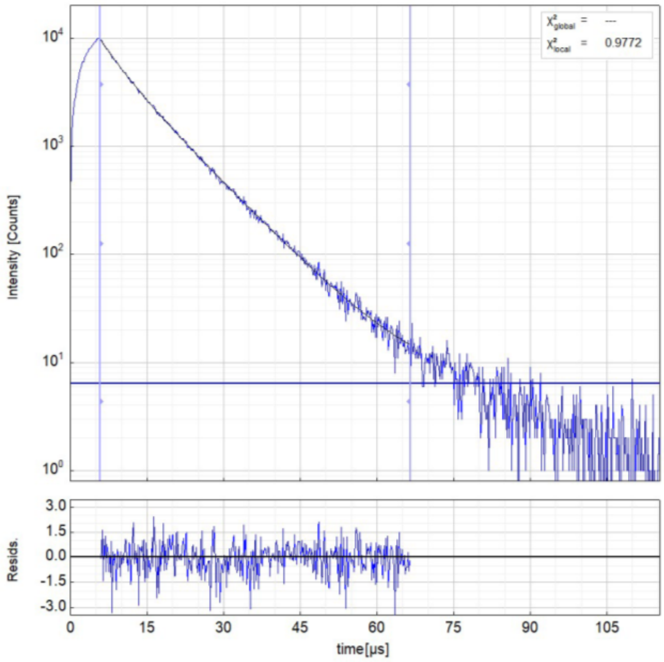

Parameter	Value	Δ
A_1[kCnts/Chnl]	6.39	±0.29
τ_1[ns]	9 100	±130
I_1[kCnts]	364	±11
A_2[kCnts/Chnl]	3.23	±0.27
τ_2[ns]	3 870	±270
I_2[kCnts]	78	±12
$Bkgr_{Dec}$[kCnts]	0.0064	±0.0011
τ_{AvInt}[ns]	8 171	±23

Figure S202. Left: Raw (experimental) time-resolved photoluminescence decay of **2** (10^{-5} M) in DCM at 77 K (λ_{exc} = 405 nm, λ_{em} = 504 nm). Right: Fitting parameters and confidence limits.

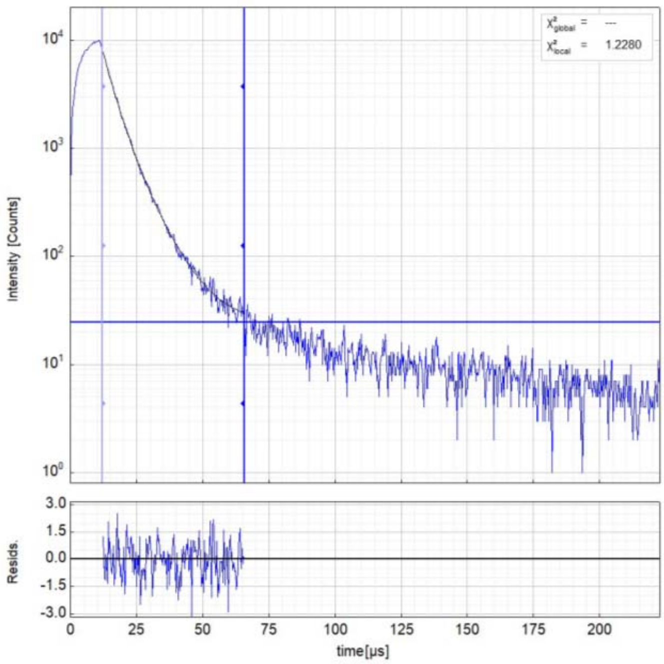

Parameter	Value	Δ
A_1[kCnts/Chnl]	2.23	±0.61
τ_1[ns]	8 840	±870
I_1[kCnts]	62	±12
A_2[kCnts/Chnl]	5.31	±0.53
τ_2[ns]	4 050	±310
I_2[kCnts]	68	±12
$Bkgr_{Dec}$[kCnts]	0.0247	±0.0028
τ_{AvInt}[ns]	6 330	±120

Figure S203. Left: Raw (experimental) time-resolved photoluminescence decay of **2** (10^{-5} M) in DCM at 77 K (λ_{exc} = 405 nm, λ_{em} = 654 nm). Right: Fitting parameters and confidence limits.

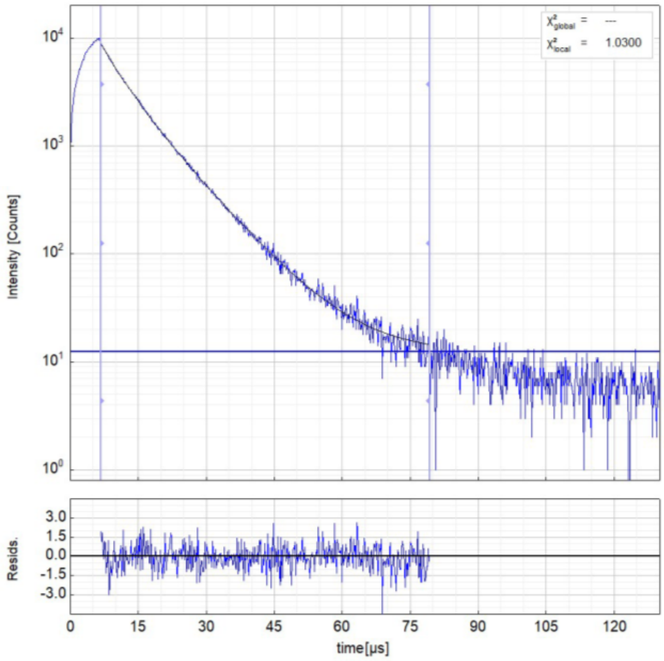

Parameter	Value	Δ
A_1[kCnts/Chnl]	4.77	±0.47
τ_1[ns]	9 420	±250
I_1[kCnts]	281	±19
A_2[kCnts/Chnl]	4.07	±0.39
τ_2[ns]	4 360	±320
I_2[kCnts]	111	±19
$Bkgr_{Dec}$[kCnts]	0.0124	±0.0007
τ_{AvInt}[ns]	7 980	±16

Figure S204. Left: Raw (experimental) time-resolved photoluminescence decay of **2** (10^{-6} M) in DCM at 77 K (λ_{exc} = 405 nm, λ_{em} = 496 nm). Right: Fitting parameters and confidence limits.

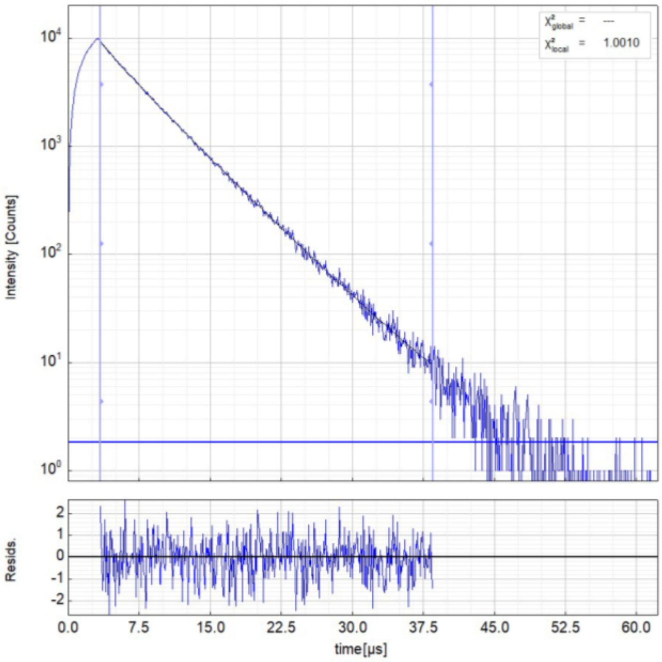

Parameter	Value	Δ
A_1[kCnts/Chnl]	7.11	±1.00
τ_1[ns]	5 140	±190
I_1[kCnts]	457	±50
A_2[kCnts/Chnl]	2.14	±0.91
τ_2[ns]	2 760	±510
I_2[kCnts]	74	±51
$Bkgr_{Dec}$[kCnts]	0.0019	±0.0014
τ_{AvInt}[ns]	4 810	±31

Figure S205. Left: Raw (experimental) time-resolved photoluminescence decay of **7** in PMMA film (2 wt%) at 298 K (λ_{exc} = 405 nm, λ_{em} = 490 nm). Right: Fitting parameters and confidence limits.

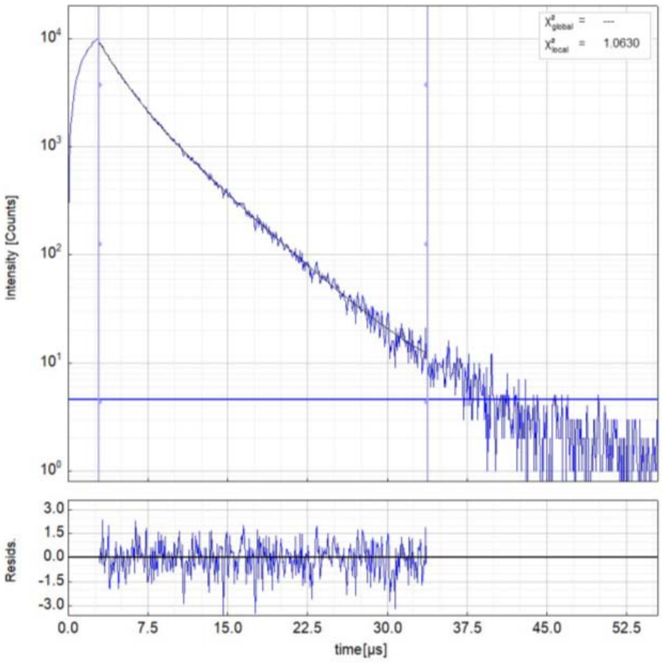

Parameter	Value	Δ
A_1[kCnts/Chnl]	4.08	±0.16
τ_1[ns]	4 896	±84
I_1[kCnts]	249.1	±5.4
A_2[kCnts/Chnl]	4.99	±0.24
τ_2[ns]	1 970	±41
I_2[kCnts]	122.7	±6.3
$Bkgr_{Dec}$[kCnts]	0.0046	±0.0013
τ_{AvInt}[ns]	3 930	±11

Figure S206. Left: Raw (experimental) time-resolved photoluminescence decay of **7** in PMMA film (2 wt%) at 298 K (λ_{exc} = 405 nm, λ_{em} = 638 nm). Right: Fitting parameters and confidence limits.

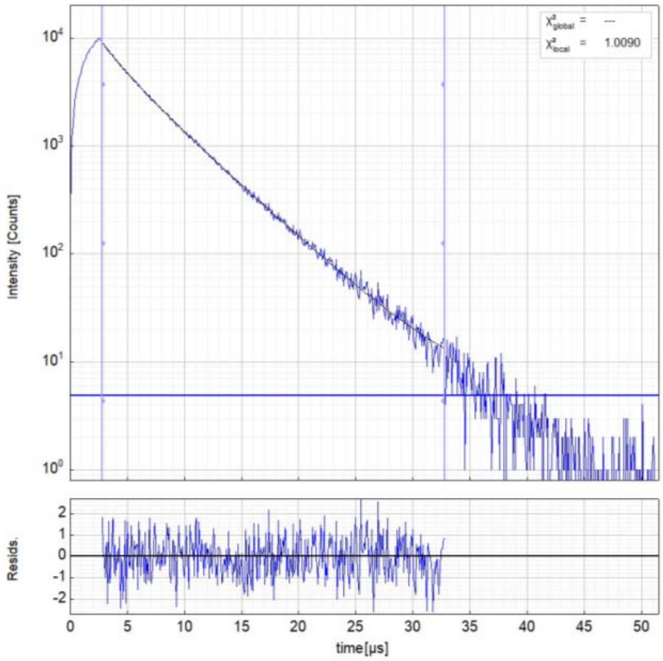

Parameter	Value	Δ
A_1[kCnts/Chnl]	6.33	±0.22
τ_1[ns]	4 525	±56
I_1[kCnts]	357.6	±7.8
A_2[kCnts/Chnl]	2.72	±0.20
τ_2[ns]	1 885	±80
I_2[kCnts]	63.9	±7.4
$Bkgr_{Dec}$[kCnts]	0.0049	±0.0011
τ_{AvInt}[ns]	4 124	±24

Figure S207. Left: Raw (experimental) time-resolved photoluminescence decay of **7** in PMMA film (5 wt%) at 298 K (λ_{exc} = 405 nm, λ_{em} = 492 nm). Right: Fitting parameters and confidence limits.

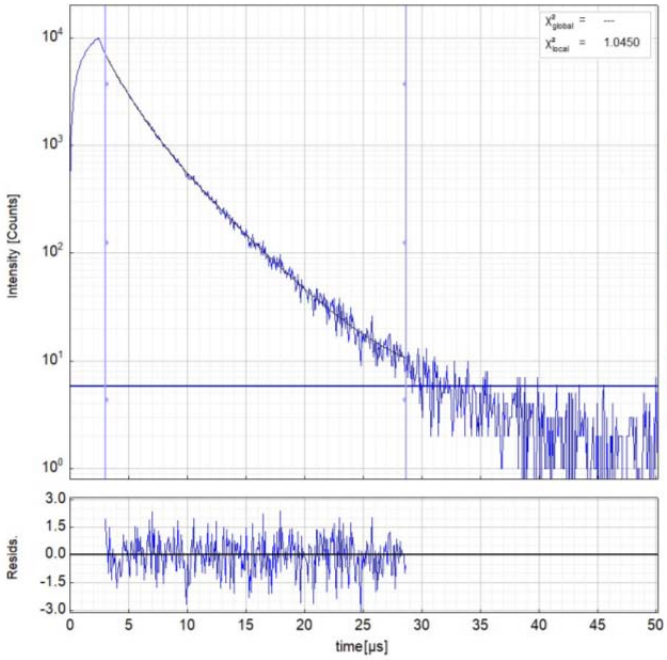

Parameter	Value	Δ
A_1[kCnts/Chnl]	2.674	±0.094
τ_1[ns]	4 056	±62
I_1[kCnts]	135.6	±3.3
A_2[kCnts/Chnl]	4.23	±0.11
τ_2[ns]	1 695	±29
I_2[kCnts]	89.4	±3.5
$Bkgr_{Dec}$[kCnts]	0.0058	±0.0007
τ_{AvInt}[ns]	3 117.4	±9.5

Figure S208. Left: Raw (experimental) time-resolved photoluminescence decay of **7** in PMMA film (5 wt%) at 298 K (λ_{exc} = 405 nm, λ_{em} = 674 nm). Right: Fitting parameters and confidence limits.

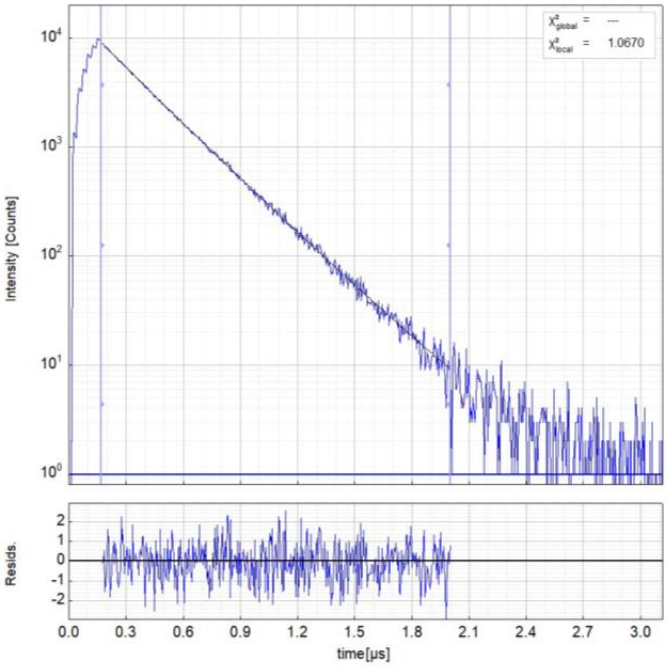

Parameter	Value	Δ
A_1[kCnts/Chnl]	7.0	±1.4
τ_1[ns]	270.4	±9.4
I_1[kCnts]	378	±58
A_2[kCnts/Chnl]	2.0	±1.4
τ_2[ns]	184	±49
I_2[kCnts]	72	±58
$Bkgr_{Dec}$[kCnts]	0.0010	±0.0010
τ_{AvInt}[ns]	256.42	±0.67

Figure S209. Left: Raw (experimental) time-resolved photoluminescence decay of **7** (10^{-3} M) in DCM at 298 K (λ_{exc} = 405 nm, λ_{em} = 490 nm). Right: Fitting parameters and confidence limits.

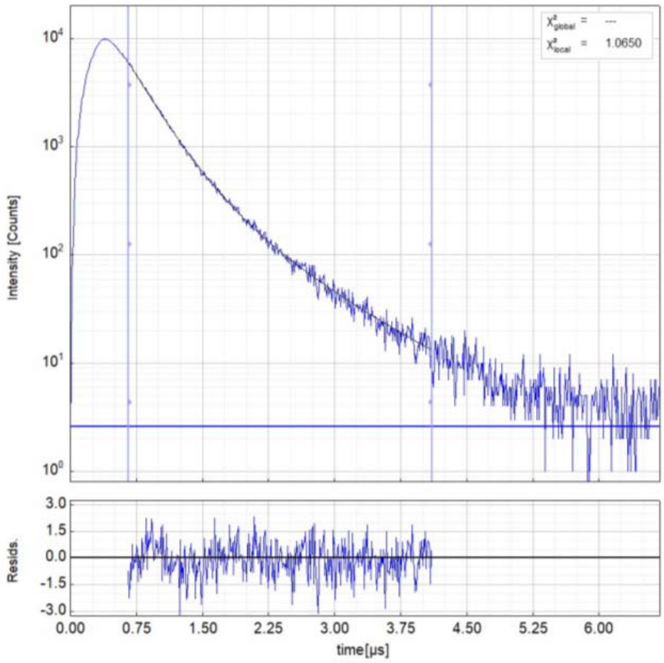

Parameter	Value	Δ
A_1[kCnts/Chnl]	0.724	±0.058
τ_1[ns]	815	±34
I_1[kCnts]	58.9	±2.8
A_2[kCnts/Chnl]	5.325	±0.061
τ_2[ns]	300.7	±3.9
I_2[kCnts]	160.2	±3.6
$Bkgr_{Dec}$[kCnts]	0.0026	±0.0015
τ_{AvInt}[ns]	438.9	±5.6

Figure S210. Left: Raw (experimental) time-resolved photoluminescence decay of **7** (10^{-3} M) in DCM at 298 K (λ_{exc} = 405 nm, λ_{em} = 664 nm). Right: Fitting parameters and confidence limits.

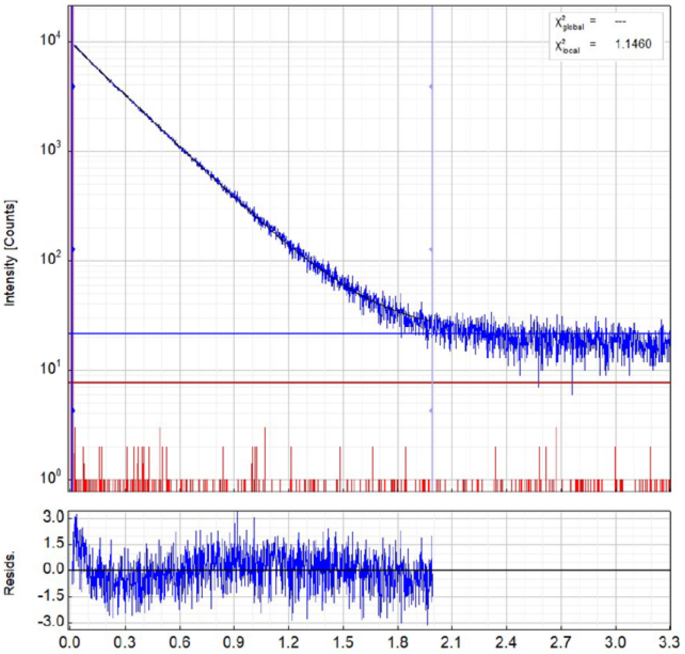

Parameter	Value	Error
A_1[kCnts/Chnl]	26.41	± 0.25
τ_1[ns]	270.57	± 0.33
I_1[kCnts]	4466	± 44
$A_{Rel\,1}$[%]	100.0000	± 0.0000
$I_{Rel\,1}$[%]	100.0000	± 0.0000
$Bkgr_{Dec}$[kCnts]	0.0217	± 0.0004
$Bkgr_{IRF}$[Cnts/Chnl]	7.63	± 0.17
$Shift_{IRF}$[ps]	-1347	± 11
$\tau_{Av\,Int}$[ns]	270.57	± 0.33
$\tau_{Av\,Amp}$[ns]	270.57	± 0.33

Figure S211. Left: Raw (experimental) time-resolved photoluminescence decay of **7** (10^{-3} M) in DCM at 298 K (λ_{exc} = 405 nm, λ_{em} = 490 nm). Right: Fitting parameters and confidence limits.

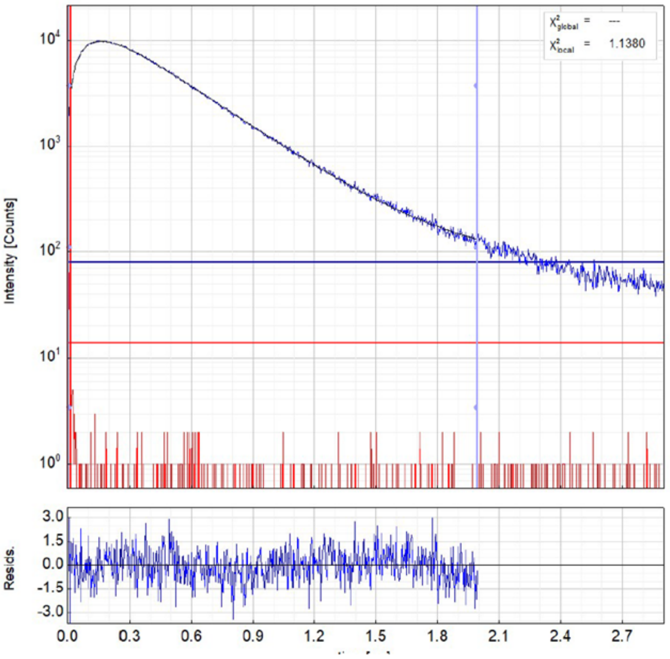

Parameter	Value	Error
A_1[kCnts/Chnl]	125.25	± 0.53
τ_1[ns]	327.81	± 0.53
I_1[kCnts]	12830	± 81
$A_{Rel\,1}$[%]	100.0000	± 0.0000
$I_{Rel\,1}$[%]	100.0000	± 0.0000
A_2[kCnts/Chnl]	-117.39	± 0.75
τ_2[ns]	97.23	± 0.40
I_2[kCnts]	-3567	± 31
$A_{Rel\,2}$[%]	0.0000	± 0.0000
$I_{Rel\,2}$[%]	0.0000	± 0.0000
$Bkgr_{Dec}$[kCnts]	0.0803	± 0.0022
$Bkgr_{IRF}$[Cnts/Chnl]	13.898	± 0.076
$Shift_{IRF}$[ps]	-3199.98	± 0.22
$\tau_{Av\,Int}$[ns]	327.81	± 0.53
$\tau_{Av\,Amp}$[ns]	327.81	± 0.53

Figure S212. Left: Raw (experimental) time-resolved photoluminescence decay of **7** (10^{-3} M) in DCM at 298 K (λ_{exc} = 405 nm, λ_{em} = 660 nm). Right: Fitting parameters and confidence limits. Fitted as biexponential function with a rise-time.

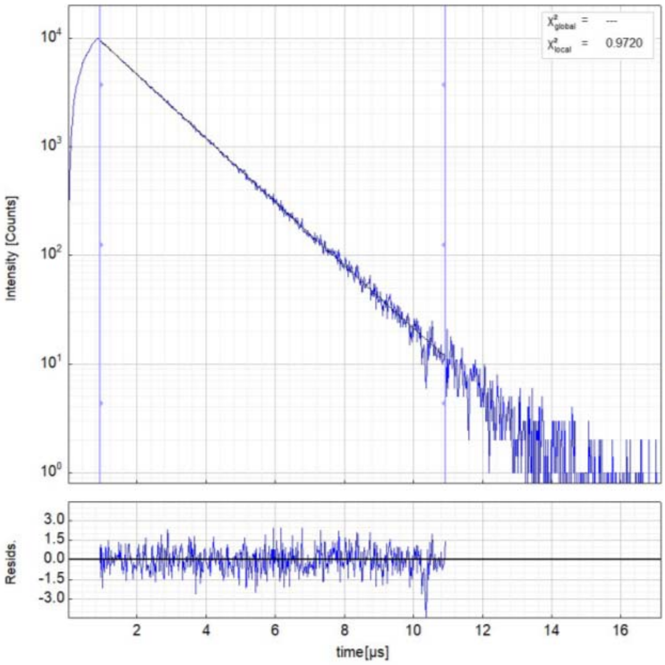

Parameter	Value	Δ
A_1[kCnts/Chnl]	9.617	±0.011
τ_1[ns]	1 477.7	±2.9
I_1[kCnts]	710.47	±0.56
$Bkgr_{Dec}$[kCnts]	0.0008	±0.0009
τ_{AvInt}[ns]	1 477.7	±2.9

Figure S213. Left: Raw (experimental) time-resolved photoluminescence decay of **7** (10^{-4} M) in DCM at 298 K (λ_{exc} = 405 nm, λ_{em} = 490 nm). Right: Fitting parameters and confidence limits.

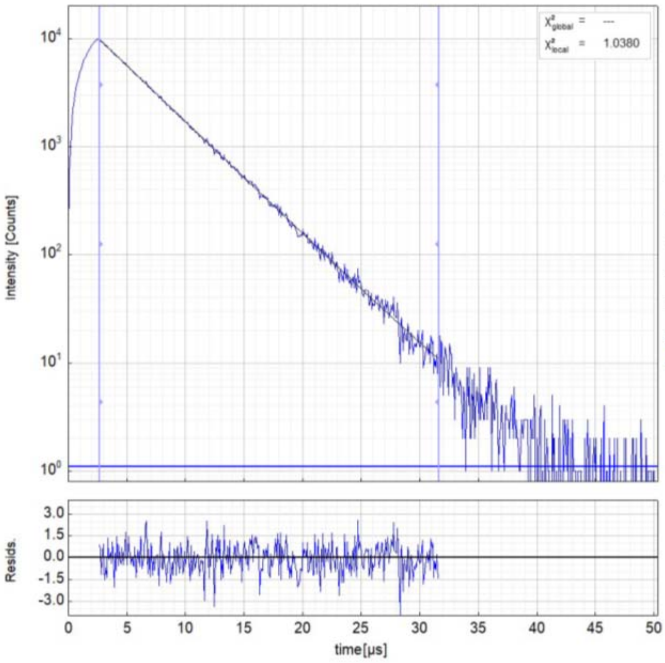

Parameter	Value	Δ
A_1[kCnts/Chnl]	9.830	±0.020
τ_1[ns]	4 190.6	±6.9
I_1[kCnts]	514.90	±0.69
$Bkgr_{Dec}$[kCnts]	0.0012	±0.0003
τ_{AvInt}[ns]	4 190.6	±6.9

Figure S214. Left: Raw (experimental) time-resolved photoluminescence decay of **7** (10^{-5} M) in DCM at 298 K (λ_{exc} = 405 nm, λ_{em} = 490 nm). Right: Fitting parameters and confidence limits.

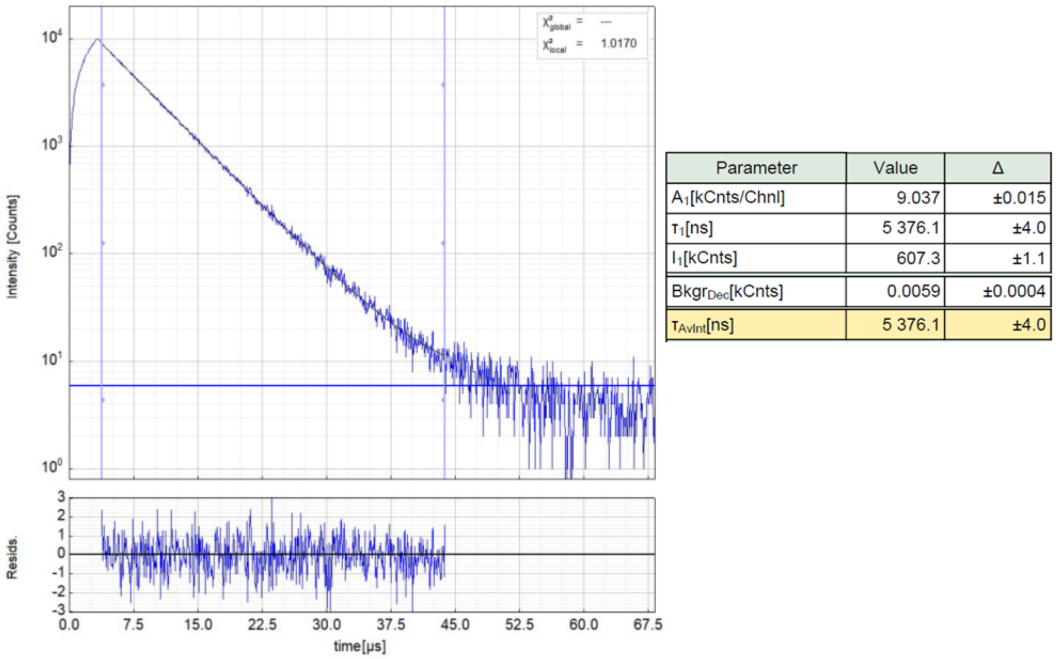

Parameter	Value	Δ
A_1[kCnts/Chnl]	9.037	±0.015
τ_1[ns]	5 376.1	±4.0
I_1[kCnts]	607.3	±1.1
$Bkgr_{Dec}$[kCnts]	0.0059	±0.0004
τ_{AvInt}[ns]	5 376.1	±4.0

Figure S215. Left: Raw (experimental) time-resolved photoluminescence decay of **7** (10^{-6} M) in DCM at 298 K (λ_{exc} = 405 nm, λ_{em} = 490 nm). Right: Fitting parameters and confidence limits.

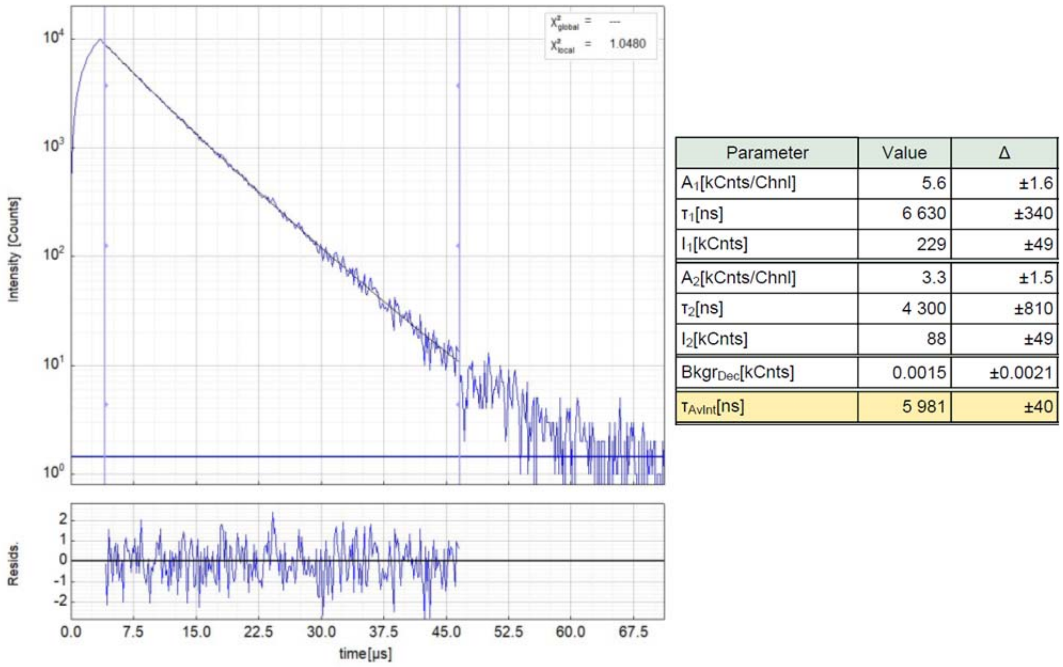

Parameter	Value	Δ
A_1[kCnts/Chnl]	5.6	±1.6
τ_1[ns]	6 630	±340
I_1[kCnts]	229	±49
A_2[kCnts/Chnl]	3.3	±1.5
τ_2[ns]	4 300	±810
I_2[kCnts]	88	±49
$Bkgr_{Dec}$[kCnts]	0.0015	±0.0021
τ_{AvInt}[ns]	5 981	±40

Figure S216. Left: Raw (experimental) time-resolved photoluminescence decay of **7** (10^{-3} M) in DCM at 77 K (λ_{exc} = 405 nm, λ_{em} = 486 nm). Right: Fitting parameters and confidence limits.

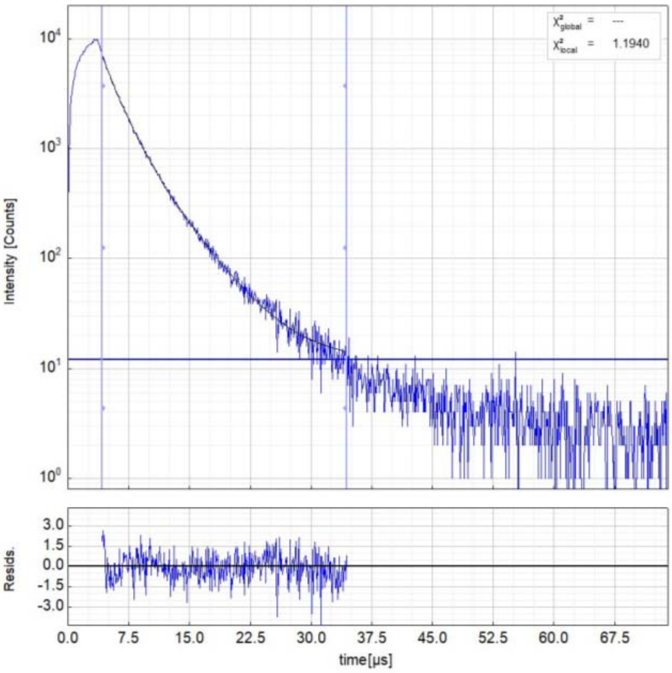

Parameter	Value	Δ
A_1[kCnts/Chnl]	2.23	±0.24
τ_1[ns]	4 380	±190
I_1[kCnts]	121.8	±7.6
A_2[kCnts/Chnl]	4.74	±0.18
τ_2[ns]	1 803	±61
I_2[kCnts]	106.7	±7.7
$Bkgr_{Dec}$[kCnts]	0.0121	±0.0009
τ_{AvInt}[ns]	3 174	±17

Figure S217. Left: Raw (experimental) time-resolved photoluminescence decay of **7** (10^{-3} M) in DCM at 77 K (λ_{exc} = 405 nm, λ_{em} = 646 nm). Right: Fitting parameters and confidence limits.

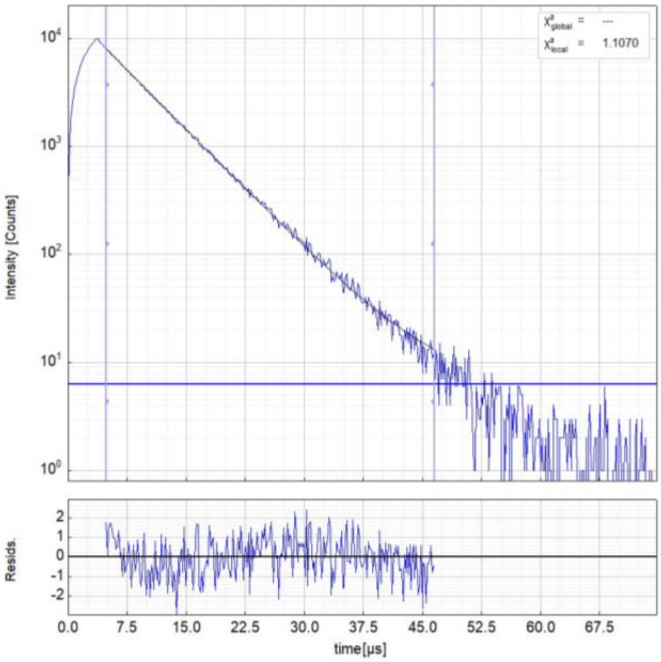

Parameter	Value	Δ
A_1[kCnts/Chnl]	7.966	±0.031
τ_1[ns]	5 915	±21
I_1[kCnts]	294.47	±0.64
$Bkgr_{Dec}$[kCnts]	0.0064	±0.0005
τ_{AvInt}[ns]	5 915	±21

Figure S218. Left: Raw (experimental) time-resolved photoluminescence decay of **7** (10^{-4} M) in DCM at 77 K (λ_{exc} = 405 nm, λ_{em} = 486 nm). Right: Fitting parameters and confidence limits.

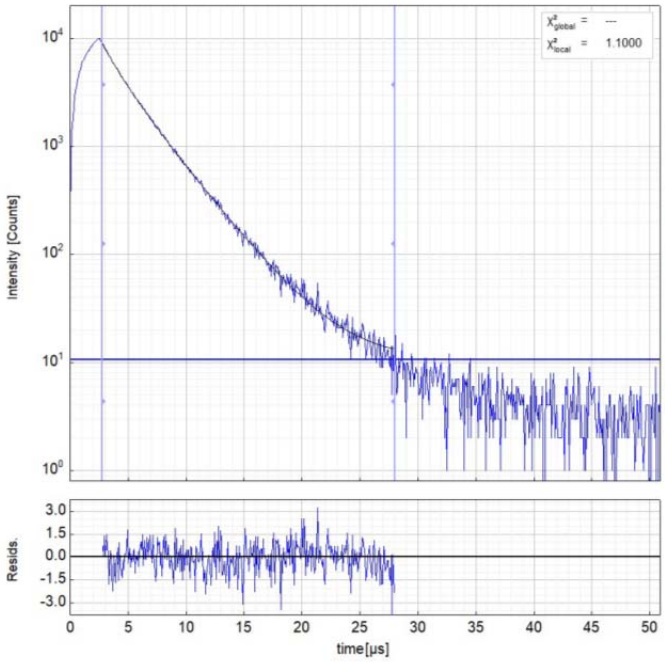

Parameter	Value	Δ
A_1[kCnts/Chnl]	5.71	±0.39
τ_1[ns]	3 273	±76
I_1[kCnts]	234	±11
A_2[kCnts/Chnl]	3.08	±0.40
τ_2[ns]	1 440	±120
I_2[kCnts]	56	±13
$Bkgr_{Dec}$[kCnts]	0.0107	±0.0009
τ_{AvInt}[ns]	2 922	±15

Figure S219. Left: Raw (experimental) time-resolved photoluminescence decay of **7** (10^{-4} M) in DCM at 77 K (λ_{exc} = 405 nm, λ_{em} = 638 nm). Right: Fitting parameters and confidence limits.

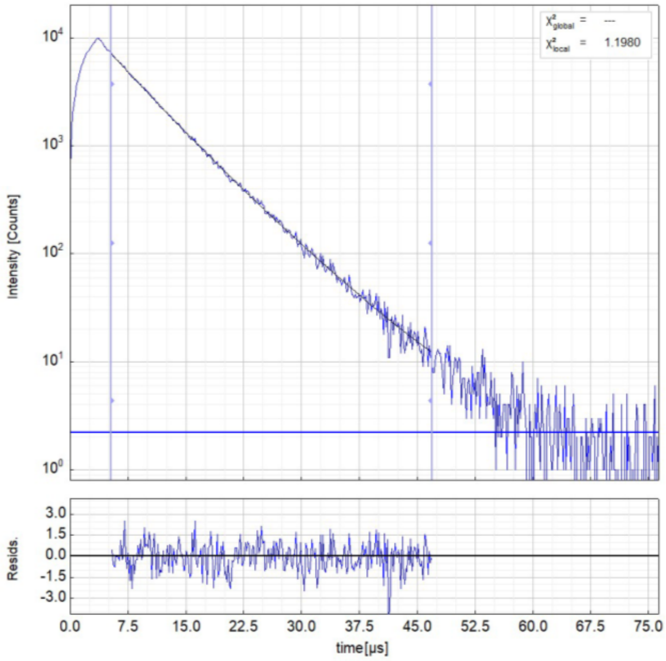

Parameter	Value	Δ
A_1[kCnts/Chnl]	3.0	±1.8
τ_1[ns]	7 210	±570
I_1[kCnts]	133	±64
A_2[kCnts/Chnl]	4.1	±1.8
τ_2[ns]	4 760	±560
I_2[kCnts]	121	±64
$Bkgr_{Dec}$[kCnts]	0.0022	±0.0009
τ_{AvInt}[ns]	6 041	±19

Figure S220. Left: Raw (experimental) time-resolved photoluminescence decay of **7** (10^{-5} M) in DCM at 77 K (λ_{exc} = 405 nm, λ_{em} = 484 nm). Right: Fitting parameters and confidence limits.

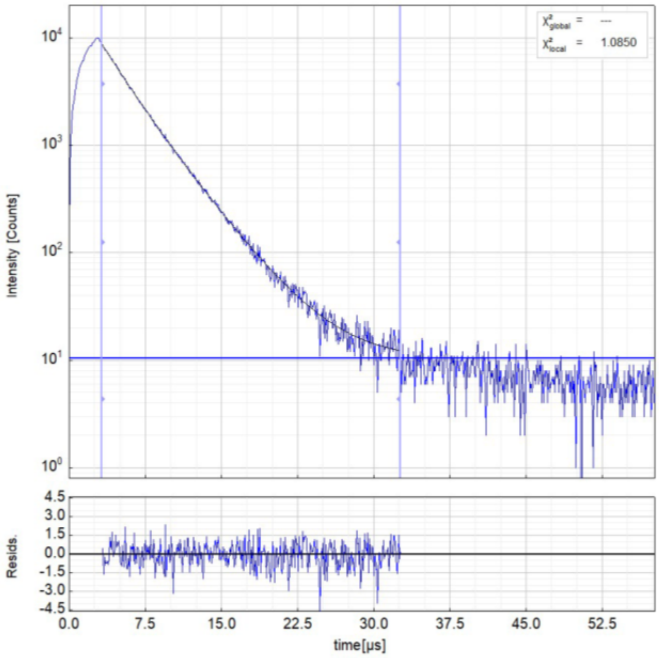

Parameter	Value	Δ
A_1[kCnts/Chnl]	4.71	±0.79
τ_1[ns]	3 760	±120
I_1[kCnts]	221	±30
A_2[kCnts/Chnl]	3.90	±0.75
τ_2[ns]	2 280	±200
I_2[kCnts]	111	±30
$Bkgr_{Dec}$[kCnts]	0.0104	±0.0008
τ_{AvInt}[ns]	3 259.2	±8.3

Figure S221. Left: Raw (experimental) time-resolved photoluminescence decay of **7** (10^{-5} M) in DCM at 77 K (λ_{exc} = 405 nm, λ_{em} = 636 nm). Right: Fitting parameters and confidence limits.

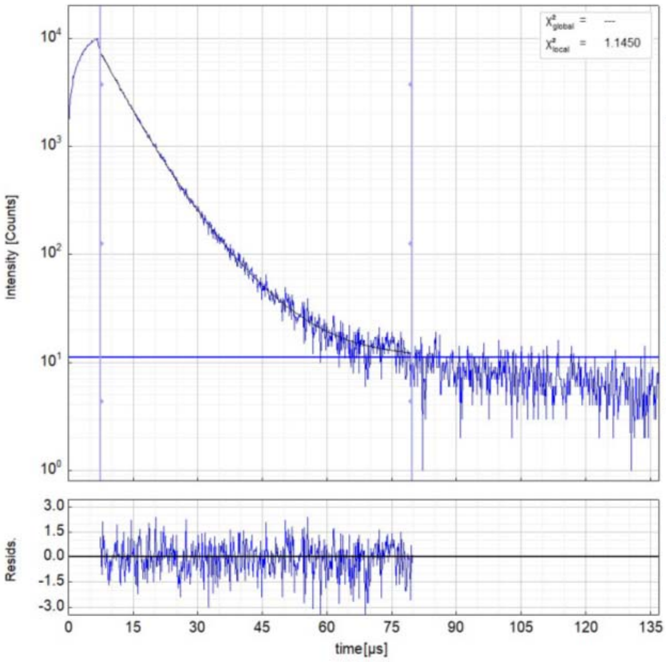

Parameter	Value	Δ
A_1[kCnts/Chnl]	1.57	±0.37
τ_1[ns]	9 920	±670
I_1[kCnts]	98	±18
A_2[kCnts/Chnl]	5.74	±0.38
τ_2[ns]	5 300	±180
I_2[kCnts]	190	±20
$Bkgr_{Dec}$[kCnts]	0.0112	±0.0007
τ_{AvInt}[ns]	6 858	±44

Figure S222. Left: Raw (experimental) time-resolved photoluminescence decay of **7** (10^{-6} M) in DCM at 77 K (λ_{exc} = 405 nm, λ_{em} = 490 nm). Right: Fitting parameters and confidence limits.

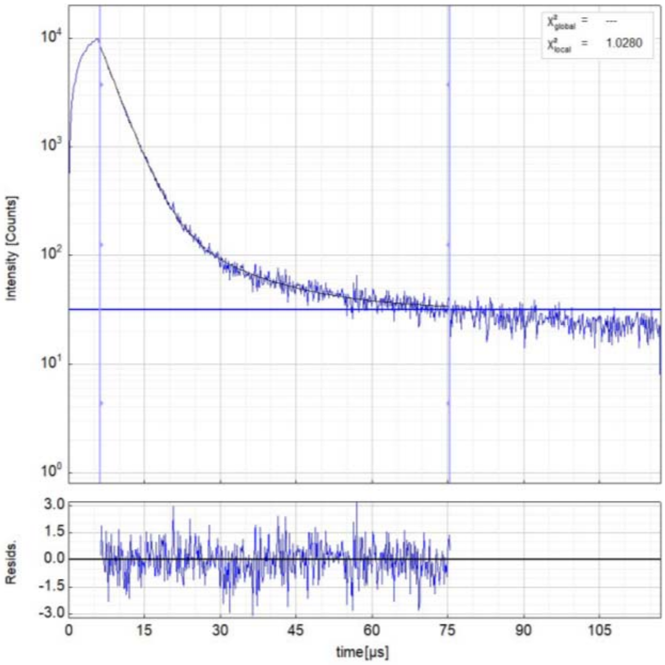

Parameter	Value	Δ
A_1[kCnts/Chnl]	0.264	±0.020
τ_1[ns]	14 630	±910
I_1[kCnts]	24.06	±0.65
A_2[kCnts/Chnl]	7.794	±0.051
τ_2[ns]	3 452	±23
I_2[kCnts]	168.2	±2.0
$Bkgr_{Dec}$[kCnts]	0.0316	±0.0011
τ_{AvInt}[ns]	4 850	±100

Figure S223. Left: Raw (experimental) time-resolved photoluminescence decay of **7** (10^{-6} M) in DCM at 77 K (λ_{exc} = 405 nm, λ_{em} = 646 nm). Right: Fitting parameters and confidence limits.

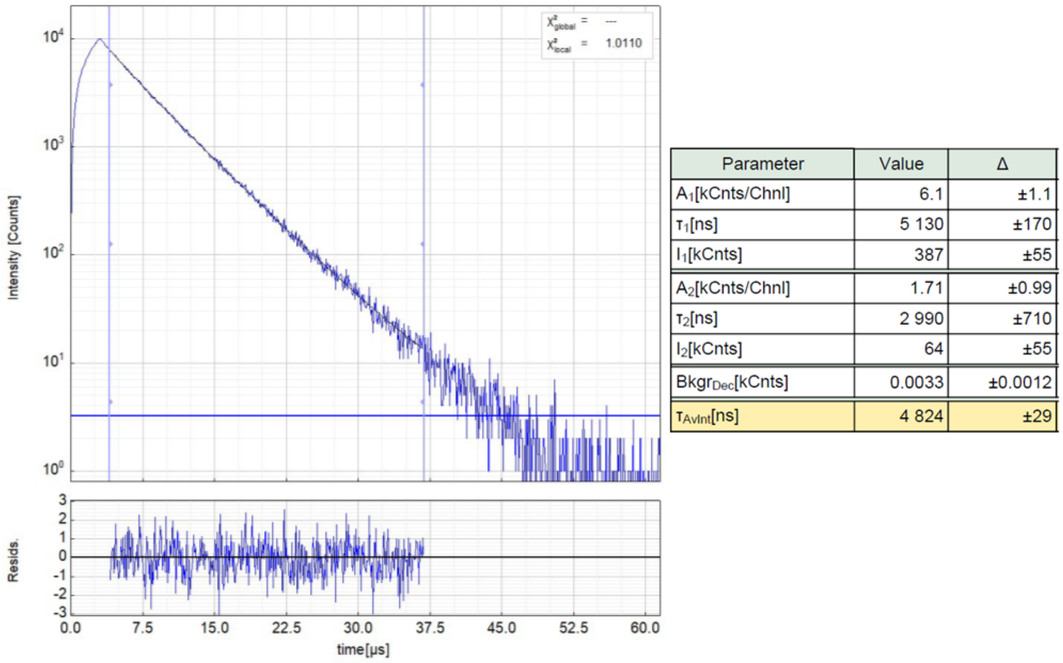

Parameter	Value	Δ
A_1[kCnts/Chnl]	6.1	±1.1
τ_1[ns]	5 130	±170
I_1[kCnts]	387	±55
A_2[kCnts/Chnl]	1.71	±0.99
τ_2[ns]	2 990	±710
I_2[kCnts]	64	±55
$Bkgr_{Dec}$[kCnts]	0.0033	±0.0012
τ_{AvInt}[ns]	4 824	±29

Figure S224. Left: Raw (experimental) time-resolved photoluminescence decay of **8** in PMMA film (2 wt%) at 298 K (λ_{exc} = 405 nm, λ_{em} = 488 nm). Right: Fitting parameters and confidence limits.

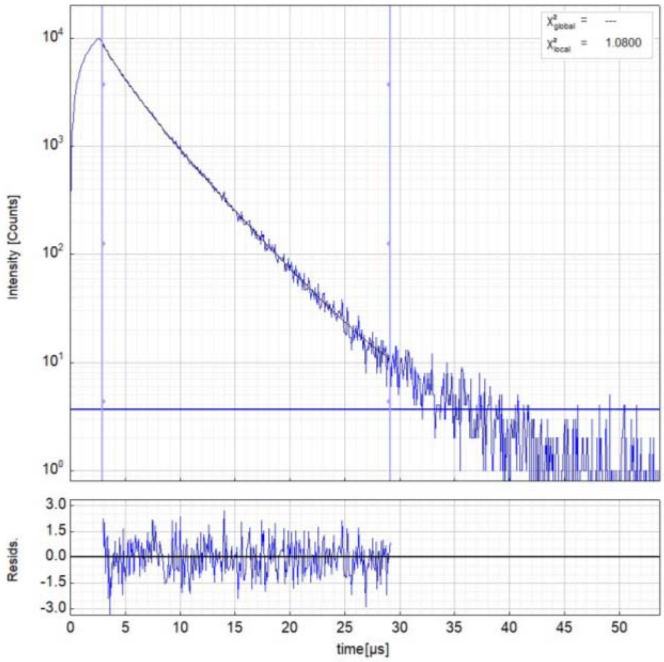

Parameter	Value	Δ
A_1[kCnts/Chnl]	5.03	±0.47
τ_1[ns]	3 990	±130
I_1[kCnts]	251	±17
A_2[kCnts/Chnl]	3.64	±0.44
τ_2[ns]	1 810	±150
I_2[kCnts]	83	±18
$Bkgr_{Dec}$[kCnts]	0.0037	±0.0015
τ_{AvInt}[ns]	3 447	±22

Figure S225. Left: Raw (experimental) time-resolved photoluminescence decay of **8** in PMMA film (5 wt%) at 298 K (λ_{exc} = 405 nm, λ_{em} = 494 nm). Right: Fitting parameters and confidence limits.

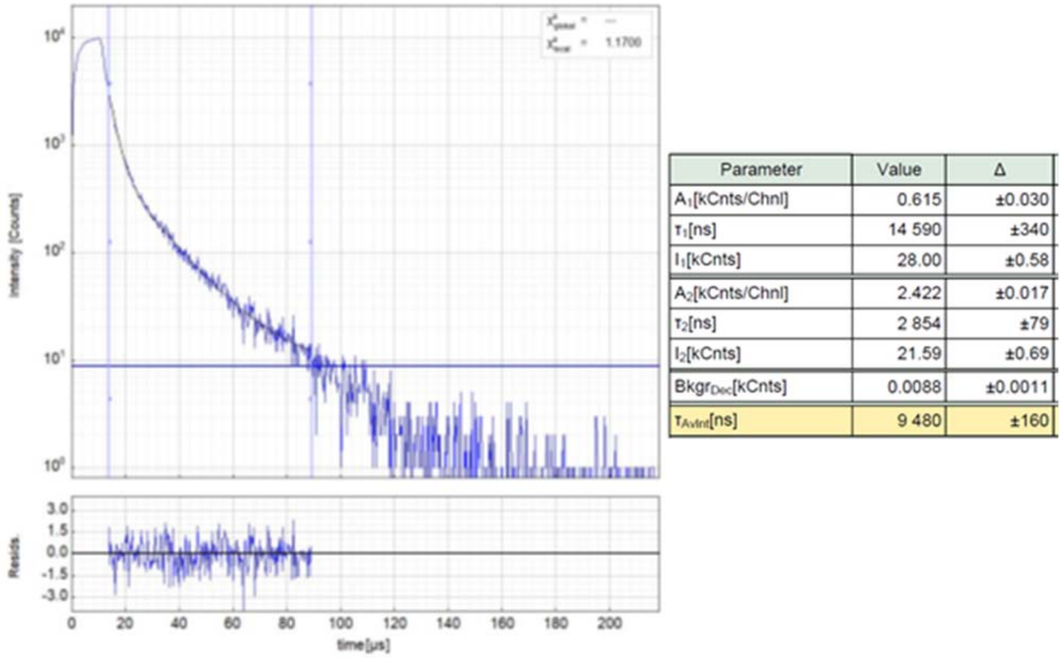

Parameter	Value	Δ
A_1[kCnts/Chnl]	0.615	±0.030
τ_1[ns]	14 590	±340
I_1[kCnts]	28.00	±0.58
A_2[kCnts/Chnl]	2.422	±0.017
τ_2[ns]	2 854	±79
I_2[kCnts]	21.59	±0.69
$Bkgr_{Dec}$[kCnts]	0.0088	±0.0011
τ_{AvInt}[ns]	9 480	±160

Figure S226. Left: Raw (experimental) time-resolved photoluminescence decay of **8** in PMMA film (5 wt%) at 298 K (λ_{exc} = 405 nm, λ_{em} = 640 nm). Right: Fitting parameters and confidence limits.

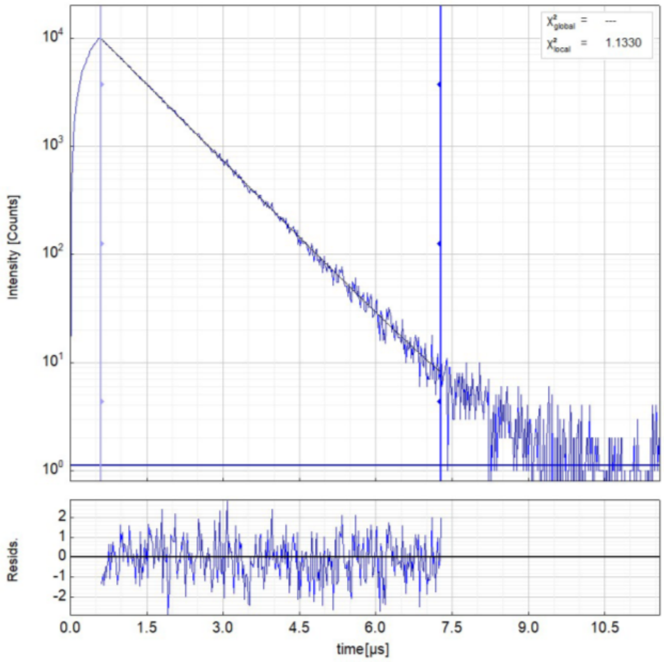

Parameter	Value	Δ
A_1[kCnts/Chnl]	9.722	±0.018
τ_1[ns]	919.8	±1.5
I_1[kCnts]	447.1	±1.1
$Bkgr_{Dec}$[kCnts]	0.0012	±0.0005
τ_{AvInt}[ns]	919.8	±1.5

Figure S227. Left: Raw (experimental) time-resolved photoluminescence decay of **8** (10^{-3} M) in DCM at 298 K (λ_{exc} = 405 nm, λ_{em} = 490 nm). Right: Fitting parameters and confidence limits.

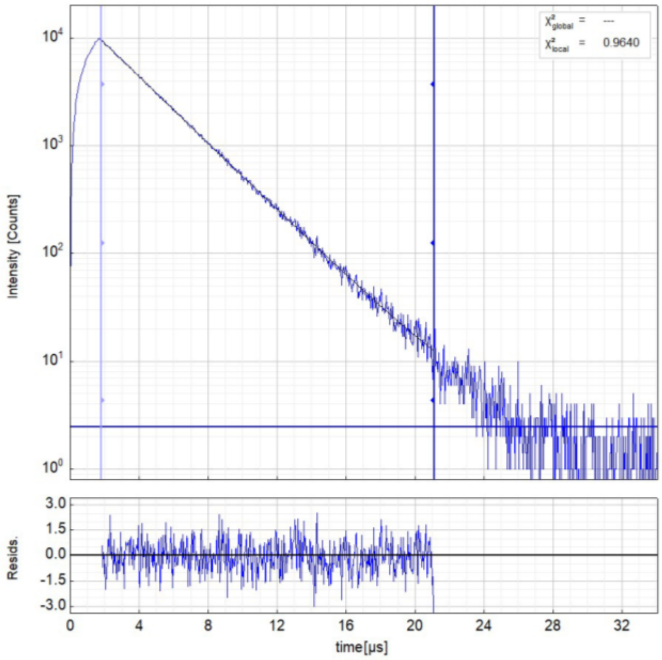

Parameter	Value	Δ
A_1[kCnts/Chnl]	9.454	±0.014
τ_1[ns]	2 813.6	±2.7
I_1[kCnts]	664.98	±0.75
$Bkgr_{Dec}$[kCnts]	0.0025	±0.0005
τ_{AvInt}[ns]	2 813.6	±2.7

Figure S228. Left: Raw (experimental) time-resolved photoluminescence decay of **8** (10^{-4} M) in DCM at 298 K (λ_{exc} = 405 nm, λ_{em} = 488 nm). Right: Fitting parameters and confidence limits.

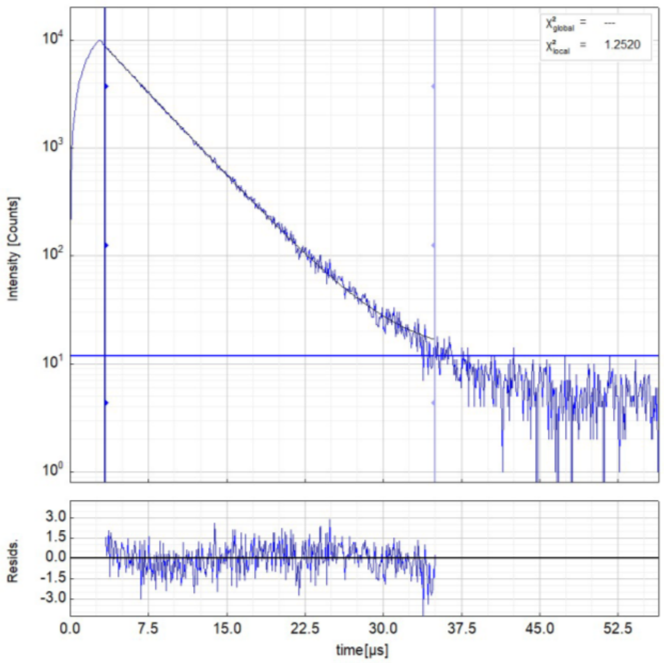

Parameter	Value	Δ
A_1[kCnts/Chnl]	8.467	±0.019
τ_1[ns]	4 231.8	±7.8
I_1[kCnts]	447.9	±1.1
$Bkgr_{Dec}$[kCnts]	0.0119	±0.0006
τ_{AvInt}[ns]	4 231.8	±7.8

Figure S229. Left: Raw (experimental) time-resolved photoluminescence decay of **8** (10^{-5} M) in DCM at 298 K (λ_{exc} = 405 nm, λ_{em} = 488 nm). Right: Fitting parameters and confidence limits.

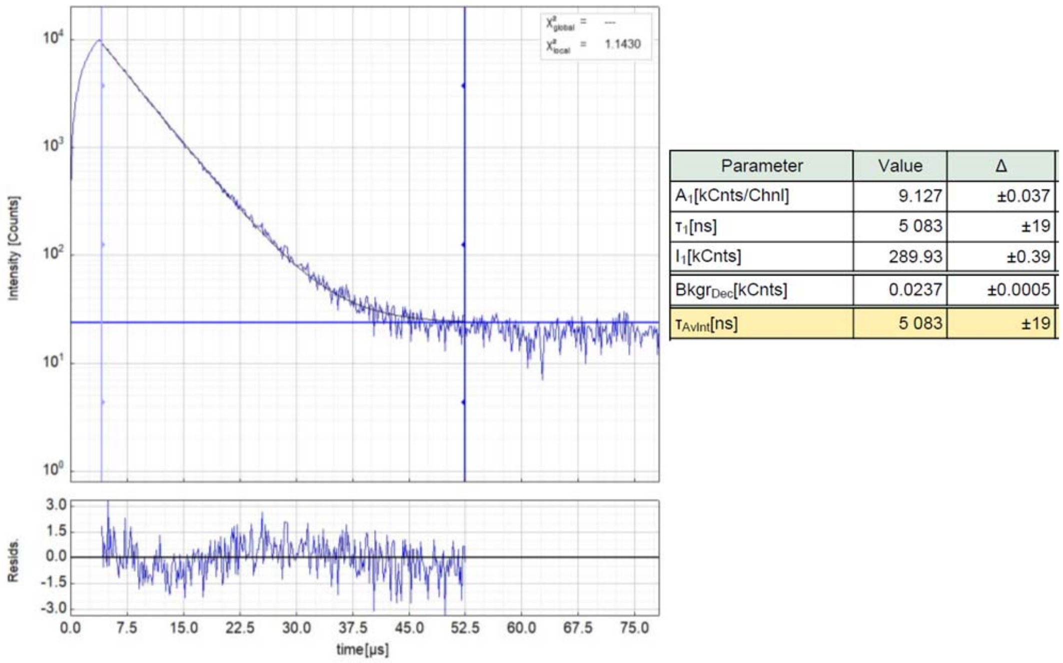

Parameter	Value	Δ
A_1[kCnts/Chnl]	9.127	±0.037
τ_1[ns]	5 083	±19
I_1[kCnts]	289.93	±0.39
$Bkgr_{Dec}$[kCnts]	0.0237	±0.0005
τ_{AvInt}[ns]	5 083	±19

Figure S230. Left: Raw (experimental) time-resolved photoluminescence decay of **8** (10^{-6} M) in DCM at 298 K (λ_{exc} = 405 nm, λ_{em} = 490 nm). Right: Fitting parameters and confidence limits.

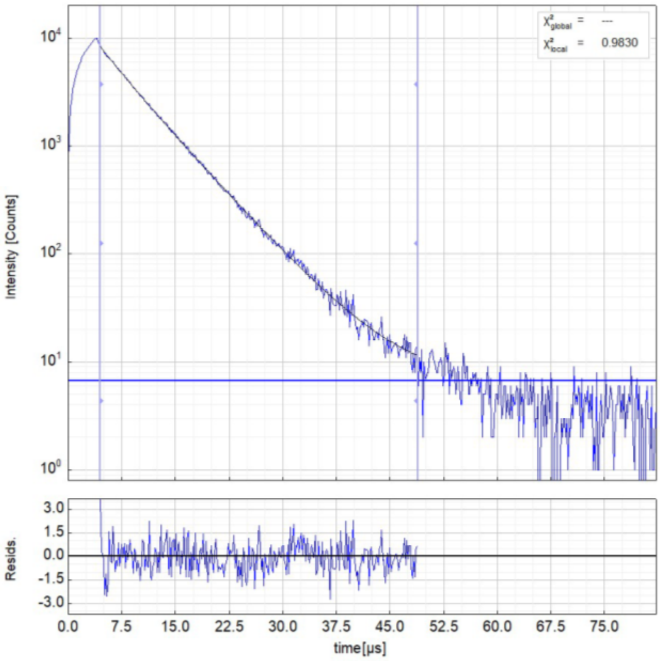

Parameter	Value	Δ
A_1[kCnts/Chnl]	6.4	±1.4
τ_1[ns]	6 170	±340
I_1[kCnts]	244	±36
A_2[kCnts/Chnl]	2.0	±1.2
τ_2[ns]	3 600	±1 300
I_2[kCnts]	44	±37
$Bkgr_{Dec}$[kCnts]	0.0067	±0.0021
τ_{AvInt}[ns]	5 767	±43

Figure S231. Left: Raw (experimental) time-resolved photoluminescence decay of **8** (10^{-4} M) in DCM at 77 K (λ_{exc} = 405 nm, λ_{em} = 490 nm). Right: Fitting parameters and confidence limits.

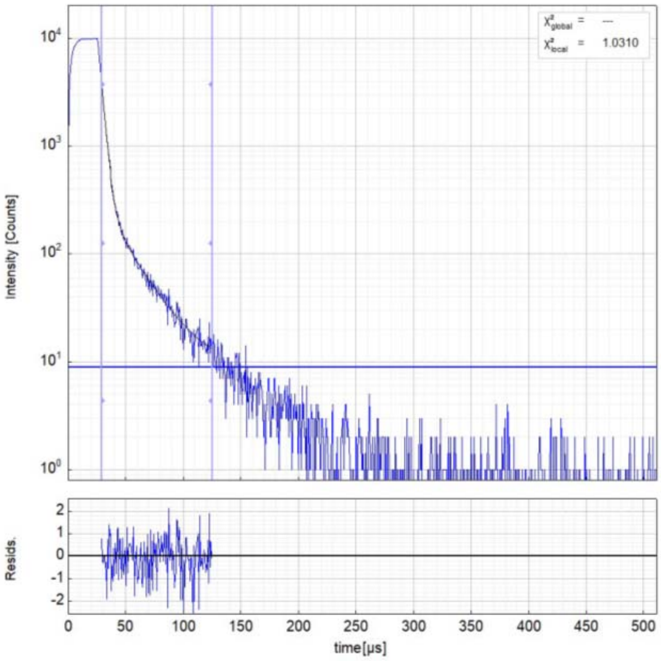

Parameter	Value	Δ
A_1[kCnts/Chnl]	0.295	±0.021
τ_1[ns]	23 000	±1 200
I_1[kCnts]	10.57	±0.23
A_2[kCnts/Chnl]	3.435	±0.027
τ_2[ns]	3 425	±62
I_2[kCnts]	18.38	±0.17
$Bkgr_{Dec}$[kCnts]	0.0089	±0.0015
τ_{AvInt}[ns]	10 570	±560

Figure S232. Left: Raw (experimental) time-resolved photoluminescence decay of **8** (10^{-4} M) in DCM at 77 K (λ_{exc} = 405 nm, λ_{em} = 652 nm). Right: Fitting parameters and confidence limits.

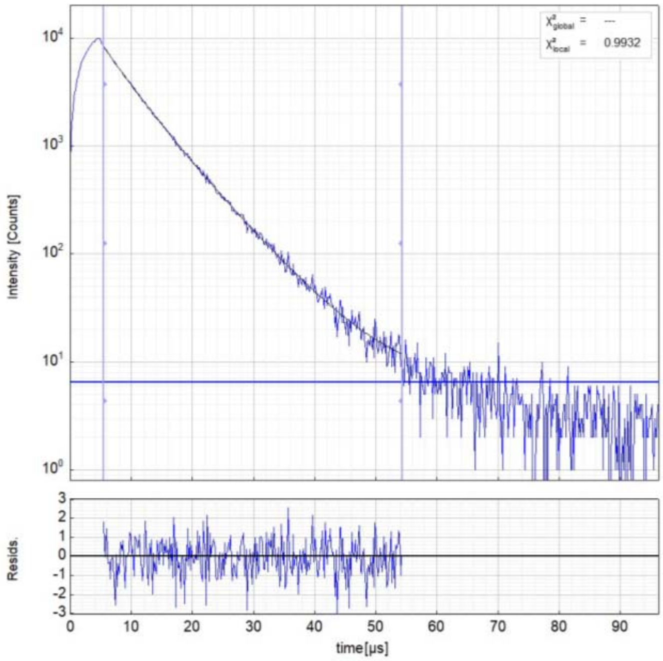

Parameter	Value	Δ
A_1[kCnts/Chnl]	4.27	±0.71
τ_1[ns]	7 300	±360
I_1[kCnts]	195	±25
A_2[kCnts/Chnl]	4.09	±0.66
τ_2[ns]	4 210	±270
I_2[kCnts]	108	±25
$Bkgr_{Dec}$[kCnts]	0.0065	±0.0007
τ_{AvInt}[ns]	6 196	±35

Figure S233. Left: Raw (experimental) time-resolved photoluminescence decay of **8** (10^{-5} M) in DCM at 77 K (λ_{exc} = 405 nm, λ_{em} = 490 nm). Right: Fitting parameters and confidence limits.

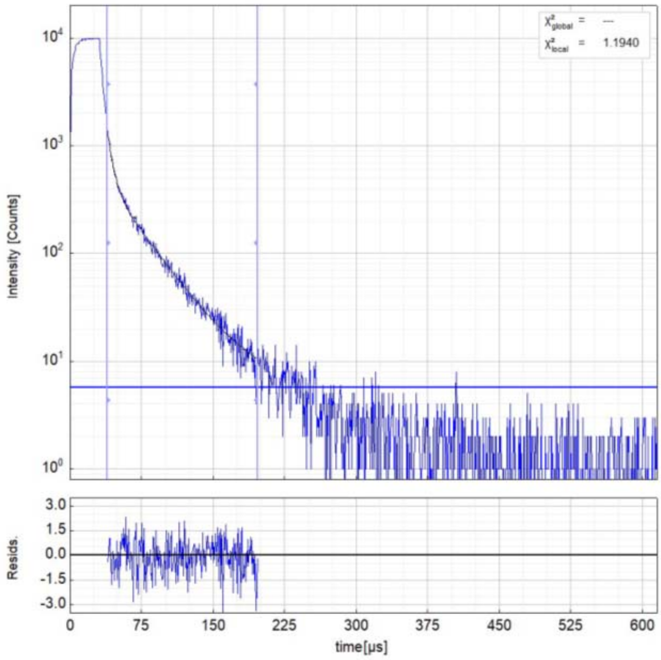

Parameter	Value	Δ
A_1[kCnts/Chnl]	0.4272	±0.0078
τ_1[ns]	35 020	±770
I_1[kCnts]	23.38	±0.35
A_2[kCnts/Chnl]	0.823	±0.018
τ_2[ns]	5 260	±170
I_2[kCnts]	6.76	±0.31
$Bkgr_{Dec}$[kCnts]	0.0057	±0.0010
τ_{AvInt}[ns]	28 350	±510

Figure S234. Left: Raw (experimental) time-resolved photoluminescence decay of **8** (10^{-5} M) in DCM at 77 K (λ_{exc} = 405 nm, λ_{em} = 652 nm). Right: Fitting parameters and confidence limits.

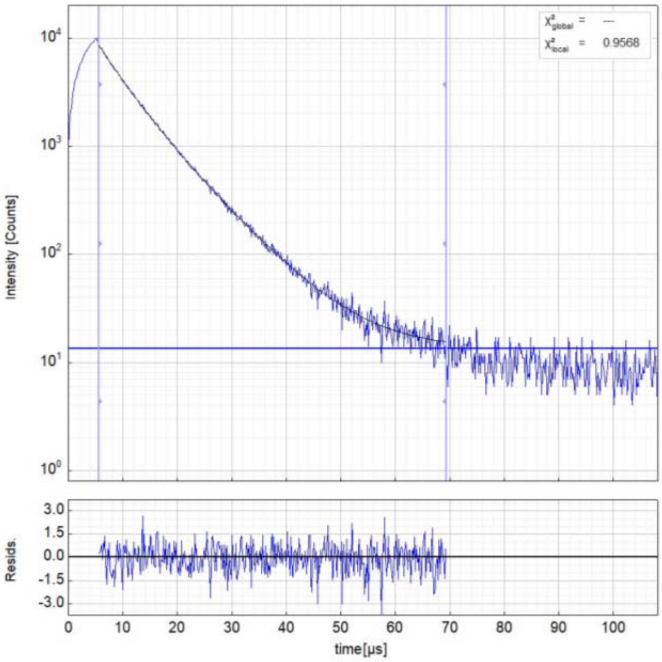

Parameter	Value	Δ
A_1[kCnts/Chnl]	4.34	±0.30
τ_1[ns]	8 260	±150
I_1[kCnts]	224	±13
A_2[kCnts/Chnl]	4.00	±0.29
τ_2[ns]	4 220	±210
I_2[kCnts]	106	±14
$Bkgr_{Dec}$[kCnts]	0.0136	±0.0007
τ_{AvInt}[ns]	6 961	±19

Figure S235. Left: Raw (experimental) time-resolved photoluminescence decay of **8** (10^{-6} M) in DCM at 77 K (λ_{exc} = 405 nm, λ_{em} = 490 nm). Right: Fitting parameters and confidence limits.

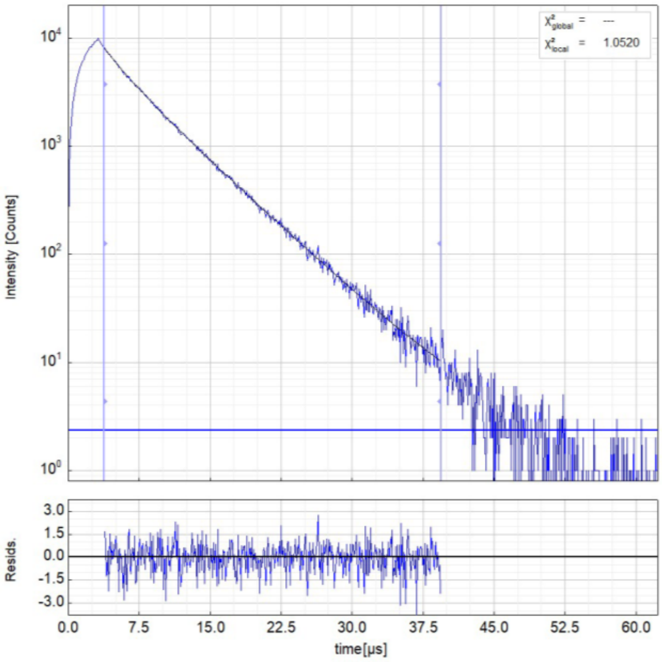

Parameter	Value	Δ
A_1[kCnts/Chnl]	5.63	±0.39
τ_1[ns]	5 420	±110
I_1[kCnts]	382	±18
A_2[kCnts/Chnl]	2.30	±0.40
τ_2[ns]	2 390	±190
I_2[kCnts]	69	±19
$Bkgr_{Dec}$[kCnts]	0.0024	±0.0014
τ_{AvInt}[ns]	4 957	±19

Figure S236. Left: Raw (experimental) time-resolved photoluminescence decay of **9** in PMMA film (2 wt%) at 298 K (λ_{exc} = 405 nm, λ_{em} = 496 nm). Right: Fitting parameters and confidence limits.

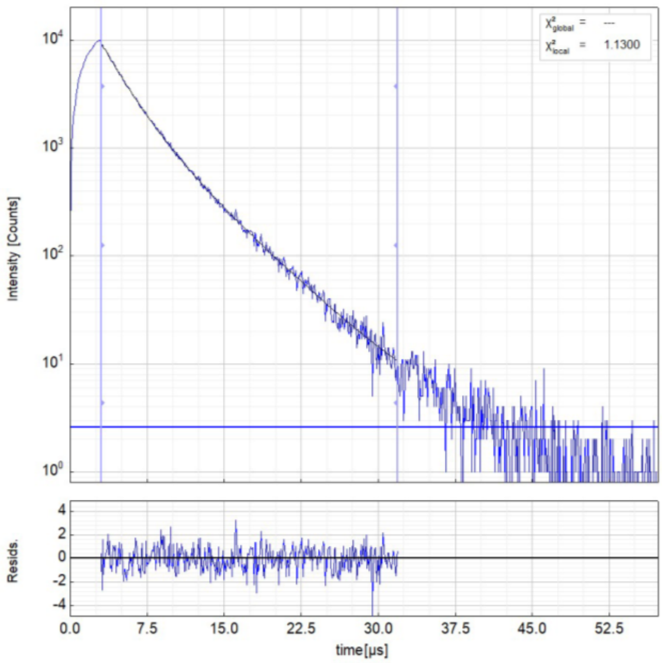

Parameter	Value	Δ
A_1[kCnts/Chnl]	2.86	±0.20
τ_1[ns]	4 920	±110
I_1[kCnts]	175.7	±7.6
A_2[kCnts/Chnl]	6.50	±0.17
τ_2[ns]	2 182	±43
I_2[kCnts]	177.3	±8.0
$Bkgr_{Dec}$[kCnts]	0.0026	±0.0011
τ_{AvInt}[ns]	3 542	±16

Figure S237. Left: Raw (experimental) time-resolved photoluminescence decay of **9** in PMMA film (2 wt%) at 298 K (λ_{exc} = 405 nm, λ_{em} = 640 nm). Right: Fitting parameters and confidence limits.

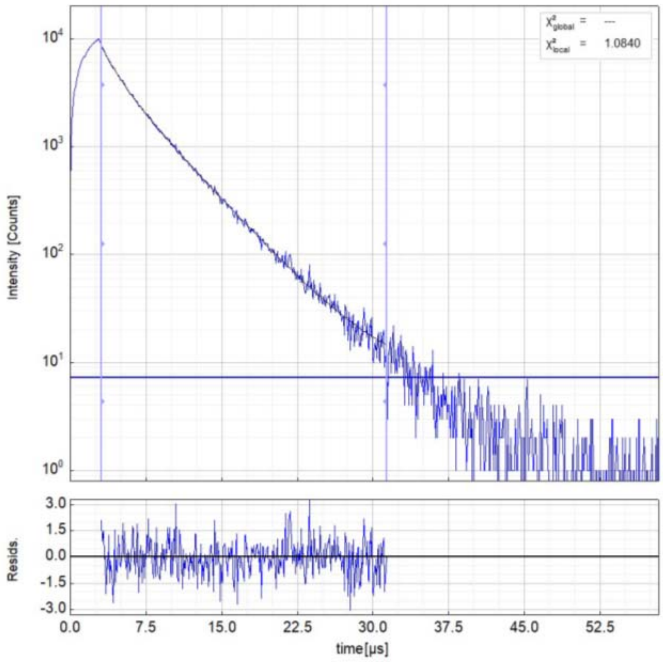

Parameter	Value	Δ
A_1[kCnts/Chnl]	5.06	±0.18
τ_1[ns]	4 314	±56
I_1[kCnts]	272.9	±5.9
A_2[kCnts/Chnl]	3.35	±0.14
τ_2[ns]	1 473	±75
I_2[kCnts]	61.6	±5.5
$Bkgr_{Dec}$[kCnts]	0.0073	±0.0008
τ_{AvInt}[ns]	3 791	±11

Figure S238. Left: Raw (experimental) time-resolved photoluminescence decay of **9** in PMMA film (5 wt%) at 298 K (λ_{exc} = 405 nm, λ_{em} = 498 nm). Right: Fitting parameters and confidence limits.

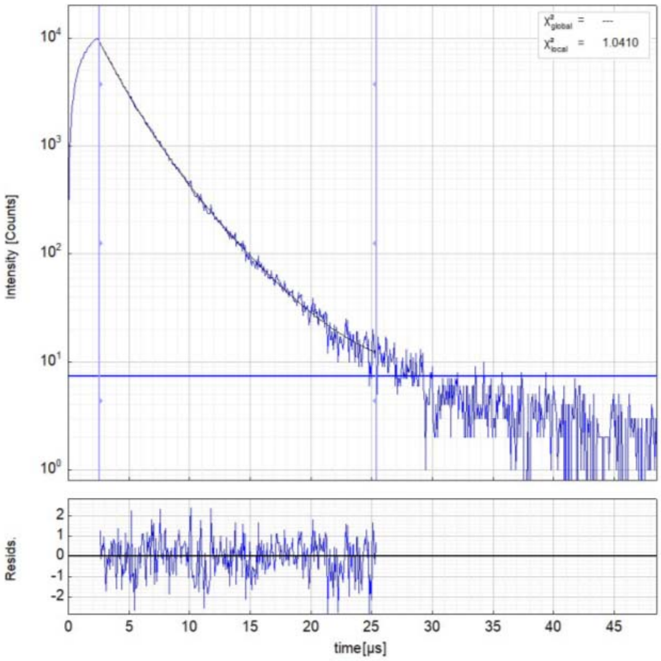

Parameter	Value	Δ
A_1[kCnts/Chnl]	2.47	±0.30
τ_1[ns]	3 640	±130
I_1[kCnts]	112.3	±10.0
A_2[kCnts/Chnl]	6.43	±0.22
τ_2[ns]	1 761	±51
I_2[kCnts]	141.5	±8.9
$Bkgr_{Dec}$[kCnts]	0.0074	±0.0015
τ_{AvInt}[ns]	2 591	±19

Figure S239. Left: Raw (experimental) time-resolved photoluminescence decay of **9** in PMMA film (5 wt%) at 298 K (λ_{exc} = 405 nm, λ_{em} = 640 nm). Right: Fitting parameters and confidence limits.

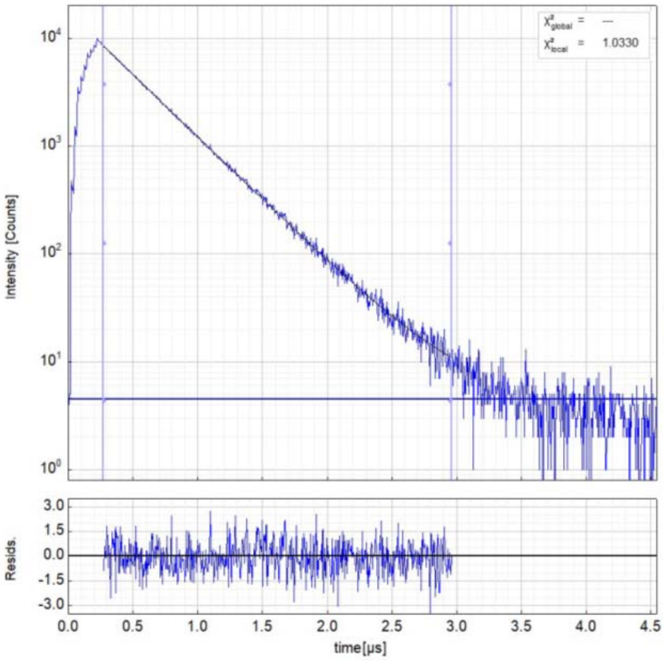

Parameter	Value	Δ
A_1[kCnts/Chnl]	8.355	±0.019
τ_1[ns]	375.12	±0.55
I_1[kCnts]	626.80	±0.72
$Bkgr_{Dec}$[kCnts]	0.0045	±0.0004
τ_{AvInt}[ns]	375.12	±0.55

Figure S240. Left: Raw (experimental) time-resolved photoluminescence decay of **9** (10^{-3} M) in DCM at 298 K (λ_{exc} = 405 nm, λ_{em} = 494 nm). Right: Fitting parameters and confidence limits.

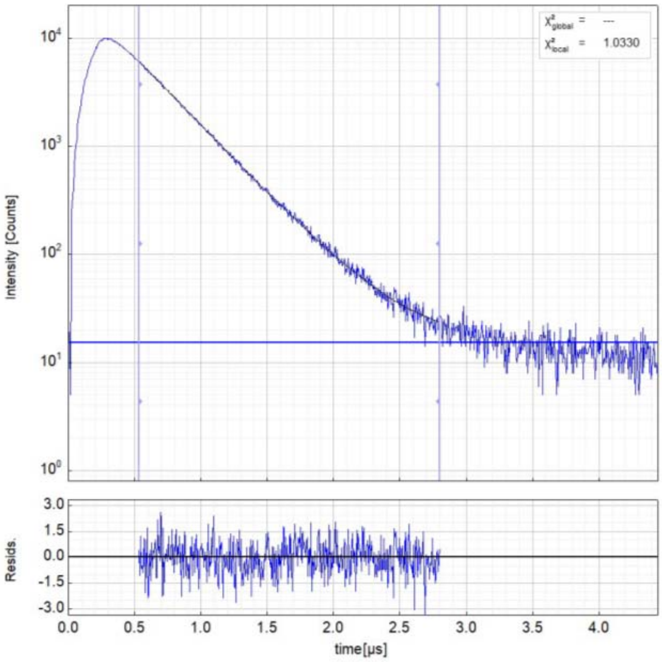

Parameter	Value	Δ
A_1[kCnts/Chnl]	6.034	±0.015
τ_1[ns]	342.69	±0.98
I_1[kCnts]	413.54	±0.43
$Bkgr_{Dec}$[kCnts]	0.0154	±0.0005
τ_{AvInt}[ns]	342.69	±0.98

Figure S241. Left: Raw (experimental) time-resolved photoluminescence decay of **9** (10^{-3} M) in DCM at 298 K (λ_{exc} = 405 nm, λ_{em} = 663 nm). Right: Fitting parameters and confidence limits.

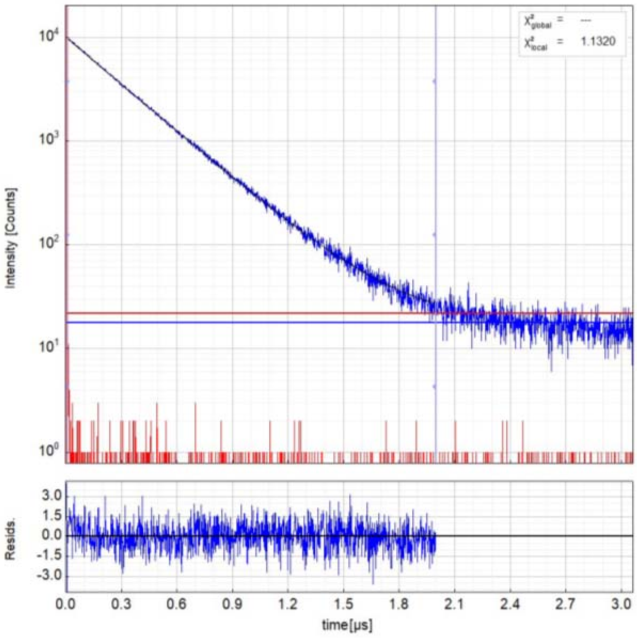

Parameter	Value	Error
A_1[kCnts/Chnl]	15.60	± 0.66
τ_1[ns]	286.36	± 0.33
I_1[kCnts]	2792	± 119
$A_{Rel\ 1}$[%]	100.0000	± 0.0000
$I_{Rel\ 1}$[%]	100.0000	± 0.0000
$Bkgr_{Dec}$[kCnts]	0.0179	± 0.0004
$Bkgr_{IRF}$[Cnts/Chnl]	21.9	± 1.8
$Shift_{IRF}$[ps]	-1600.595	± 0.044
τ_{AvInt}[ns]	286.36	± 0.33
τ_{AvAmp}[ns]	286.36	± 0.33

Figure S242. Left: Raw (experimental) time-resolved photoluminescence decay of **9** (10^{-3} M) in DCM at 298 K (λ_{exc} = 405 nm, λ_{em} = 494 nm). Right: Fitting parameters and confidence limits.

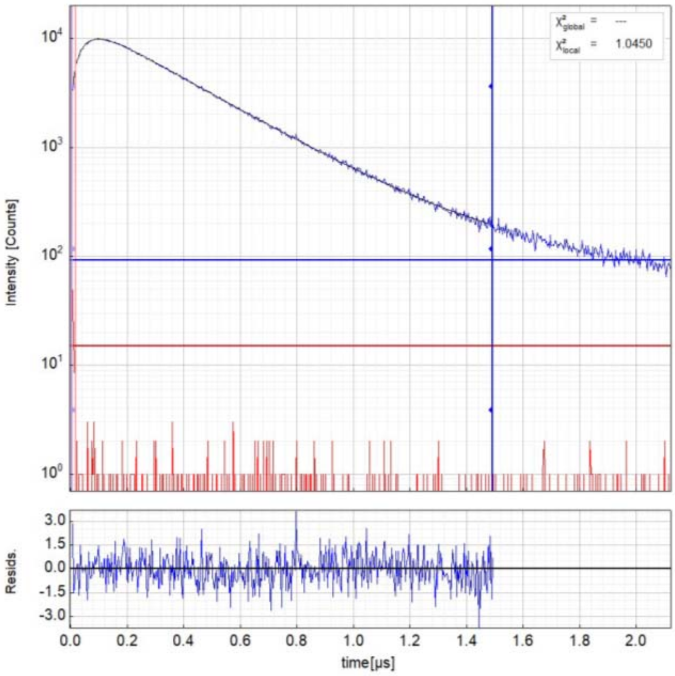

Parameter	Value	Error
A_1[kCnts/Chnl]	29.739	± 0.077
τ_1[ns]	294.60	± 0.84
I_1[kCnts]	2737.9	± 7.3
$A_{Rel\,1}$[%]	100.0000	± 0.0000
$I_{Rel\,1}$[%]	100.0000	± 0.0000
A_2[kCnts/Chnl]	-26.72	± 0.18
τ_2[ns]	49.12	± 0.58
I_2[kCnts]	-410.1	± 3.7
$A_{Rel\,2}$[%]	0.0000	± 0.0000
$I_{Rel\,2}$[%]	0.0000	± 0.0000
$Bkgr_{Dec}$[kCnts]	0.0916	± 0.0030
$Bkgr_{IRF}$[Cnts/Chnl]	15.038	± 0.049
$Shift_{IRF}$[ps]	-3201.076	± 0.017
$\tau_{Av\,Int}$[ns]	294.60	± 0.84
$\tau_{Av\,Amp}$[ns]	294.60	± 0.84

Figure S243. Left: Raw (experimental) time-resolved photoluminescence decay of **9** (10^{-3} M) in DCM at 298 K (λ_{exc} = 405 nm, λ_{em} = 663 nm). Right: Fitting parameters and confidence limits. Fitted as biexponential function with a rise-time.

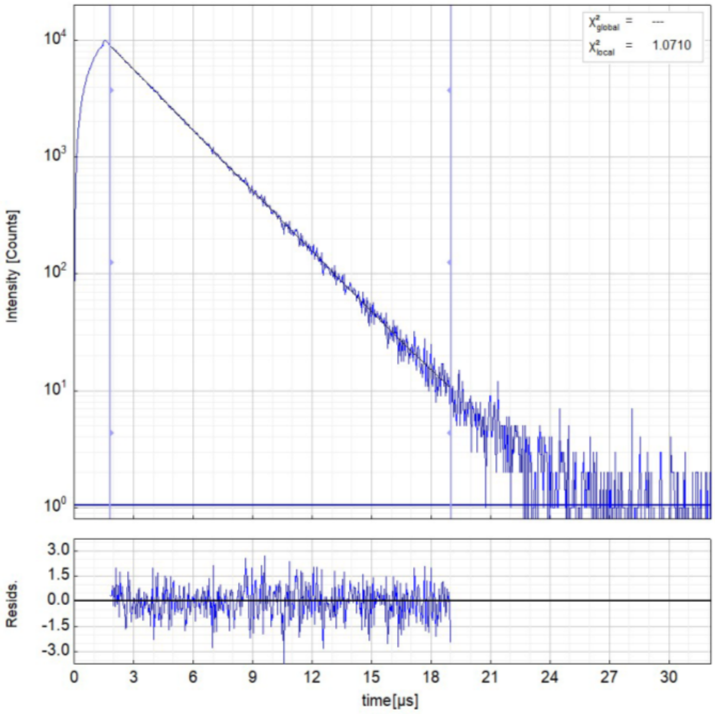

Parameter	Value	Δ
A_1[kCnts/Chnl]	8.949	±0.012
τ_1[ns]	2 504.0	±6.0
I_1[kCnts]	560.2	±1.1
$Bkgr_{Dec}$[kCnts]	0.0011	±0.0008
τ_{AvInt}[ns]	2 504.0	±6.0

Figure S244. Left: Raw (experimental) time-resolved photoluminescence decay of **9** (10^{-4} M) in DCM at 298 K (λ_{exc} = 405 nm, λ_{em} = 494 nm). Right: Fitting parameters and confidence limits.

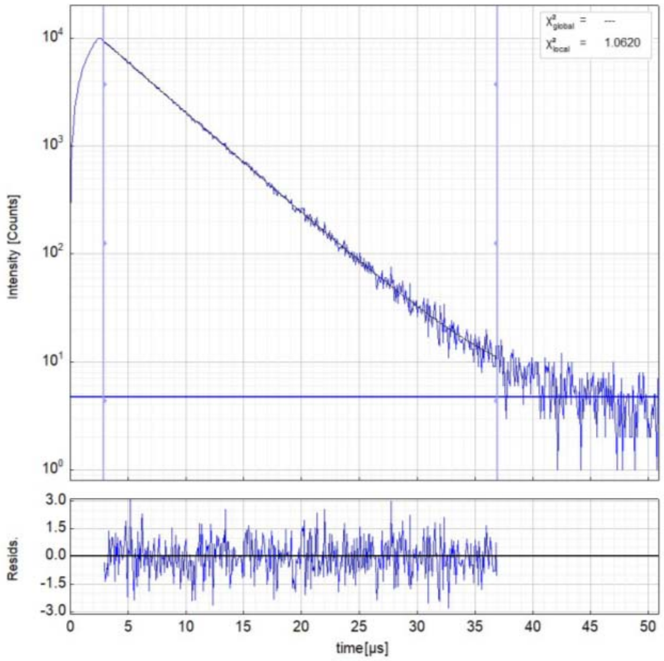

Parameter	Value	Δ
A_1[kCnts/Chnl]	9.221	±0.019
τ_1[ns]	4 663.0	±6.8
I_1[kCnts]	537.45	±0.56
$Bkgr_{Dec}$[kCnts]	0.0047	±0.0006
τ_{AvInt}[ns]	4 663.0	±6.8

Figure S245. Left: Raw (experimental) time-resolved photoluminescence decay of **9** (10^{-5} M) in DCM at 298 K (λ_{exc} = 405 nm, λ_{em} = 494 nm). Right: Fitting parameters and confidence limits.

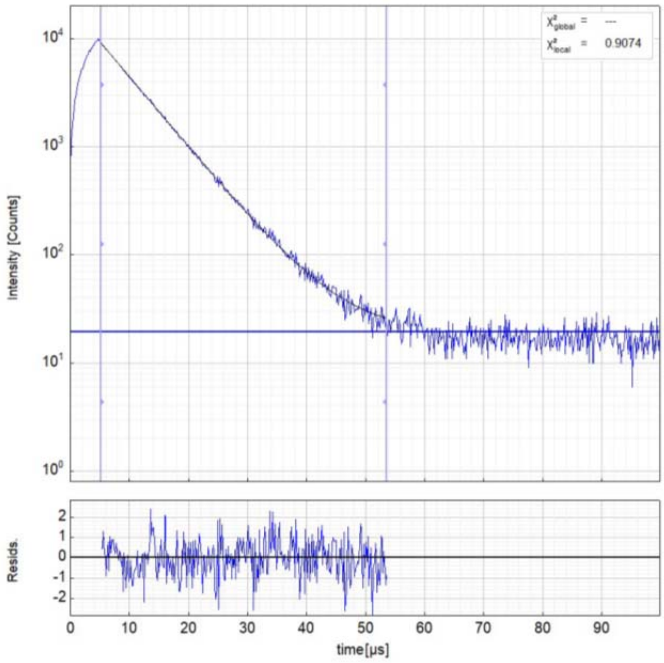

Parameter	Value	Δ
A_1[kCnts/Chnl]	8.917	±0.020
τ_1[ns]	6 690	±13
I_1[kCnts]	372.78	±0.59
$Bkgr_{Dec}$[kCnts]	0.0194	±0.0006
τ_{AvInt}[ns]	6 690	±13

Figure S246. Left: Raw (experimental) time-resolved photoluminescence decay of **9** (10^{-6} M) in DCM at 298 K (λ_{exc} = 405 nm, λ_{em} = 495 nm). Right: Fitting parameters and confidence limits.

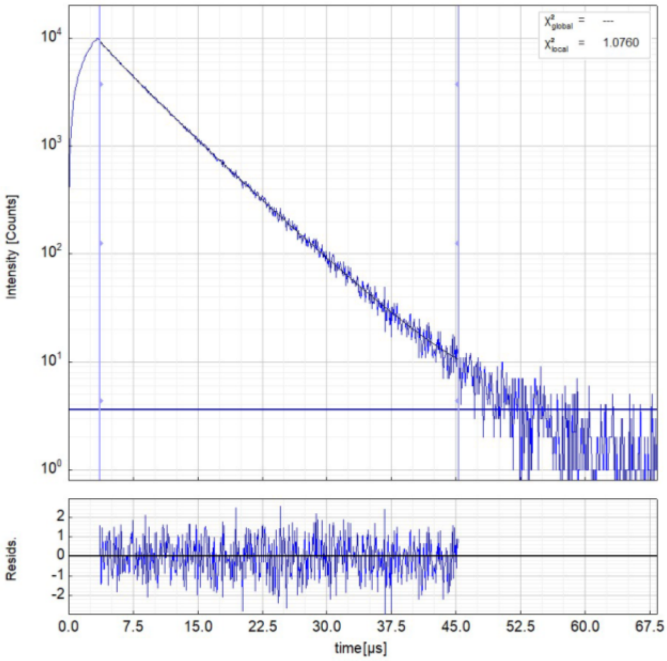

Parameter	Value	Δ
A_1[kCnts/Chnl]	7.20	±0.53
τ_1[ns]	5 989	±100
I_1[kCnts]	539	±32
A_2[kCnts/Chnl]	2.10	±0.49
τ_2[ns]	3 290	±460
I_2[kCnts]	87	±32
$Bkgr_{Dec}$[kCnts]	0.0037	±0.0011
τ_{AvInt}[ns]	5 616	±14

Figure S247. Left: Raw (experimental) time-resolved photoluminescence decay of **9** (10^{-3} M) in DCM at 77 K (λ_{exc} = 405 nm, λ_{em} = 489 nm). Right: Fitting parameters and confidence limits.

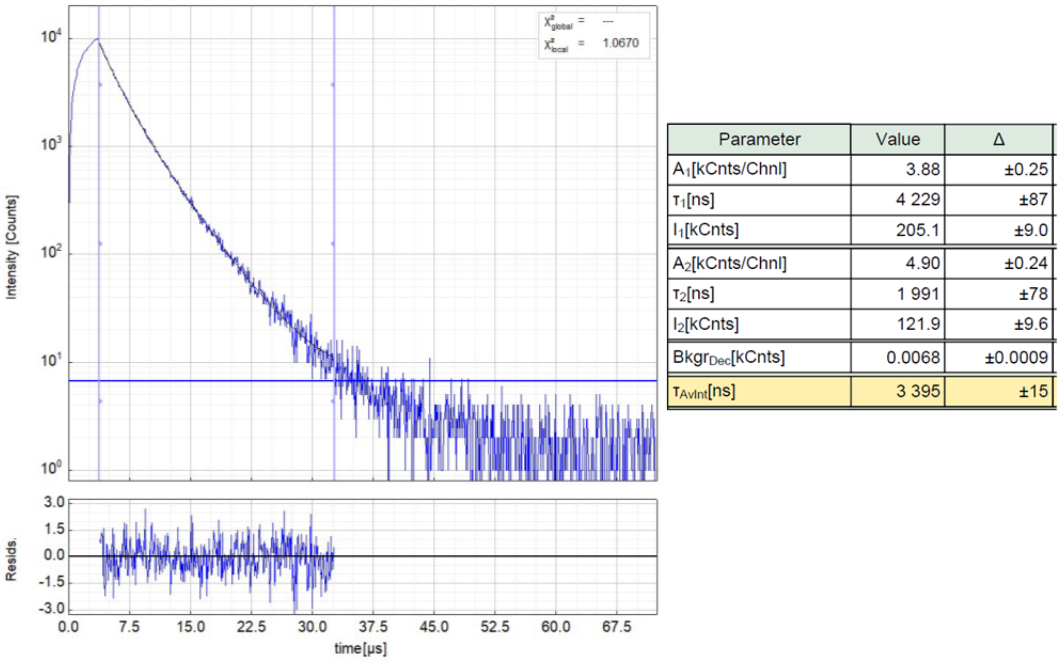

Parameter	Value	Δ
A_1[kCnts/Chnl]	3.88	±0.25
τ_1[ns]	4 229	±87
I_1[kCnts]	205.1	±9.0
A_2[kCnts/Chnl]	4.90	±0.24
τ_2[ns]	1 991	±78
I_2[kCnts]	121.9	±9.6
$Bkgr_{Dec}$[kCnts]	0.0068	±0.0009
τ_{AvInt}[ns]	3 395	±15

Figure S248. Left: Raw (experimental) time-resolved photoluminescence decay of **9** (10^{-3} M) in DCM at 77 K (λ_{exc} = 405 nm, λ_{em} = 627 nm). Right: Fitting parameters and confidence limits.

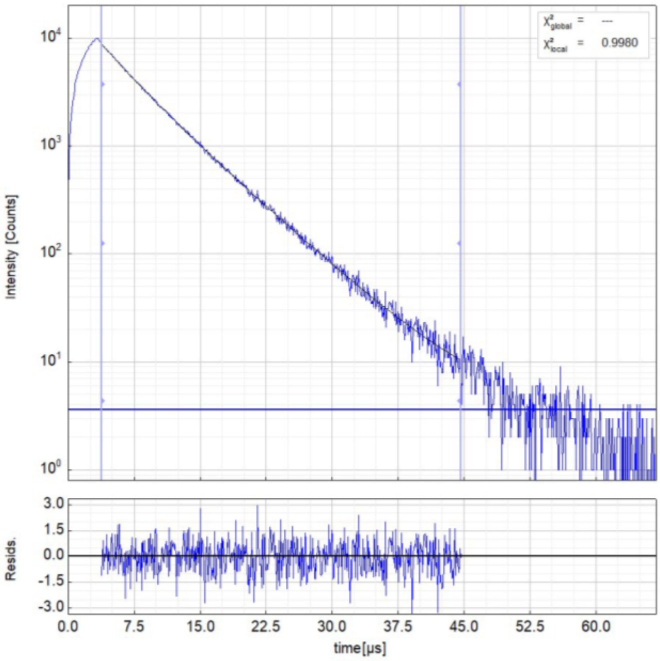

Parameter	Value	Δ
A_1[kCnts/Chnl]	5.71	±0.58
τ_1[ns]	6 060	±160
I_1[kCnts]	433	±32
A_2[kCnts/Chnl]	3.02	±0.55
τ_2[ns]	3 390	±290
I_2[kCnts]	128	±33
$Bkgr_{Dec}$[kCnts]	0.0036	±0.0012
τ_{AvInt}[ns]	5 450	±24

Figure S249. Left: Raw (experimental) time-resolved photoluminescence decay of **9** (10^{-4} M) in DCM at 77 K (λ_{exc} = 405 nm, λ_{em} = 487 nm). Right: Fitting parameters and confidence limits.

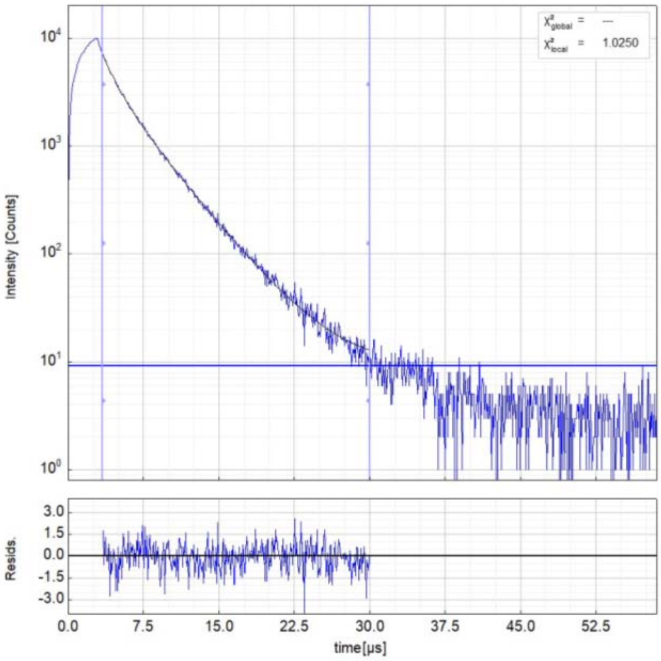

Parameter	Value	Δ
A_1[kCnts/Chnl]	3.76	±0.20
τ_1[ns]	3 831	±77
I_1[kCnts]	179.8	±5.6
A_2[kCnts/Chnl]	3.16	±0.12
τ_2[ns]	1 363	±96
I_2[kCnts]	53.8	±5.9
$Bkgr_{Dec}$[kCnts]	0.0092	±0.0008
τ_{AvInt}[ns]	3 263	±20

Figure S250. Left: Raw (experimental) time-resolved photoluminescence decay of **9** (10^{-4} M) in DCM at 77 K (λ_{exc} = 405 nm, λ_{em} = 641 nm). Right: Fitting parameters and confidence limits.

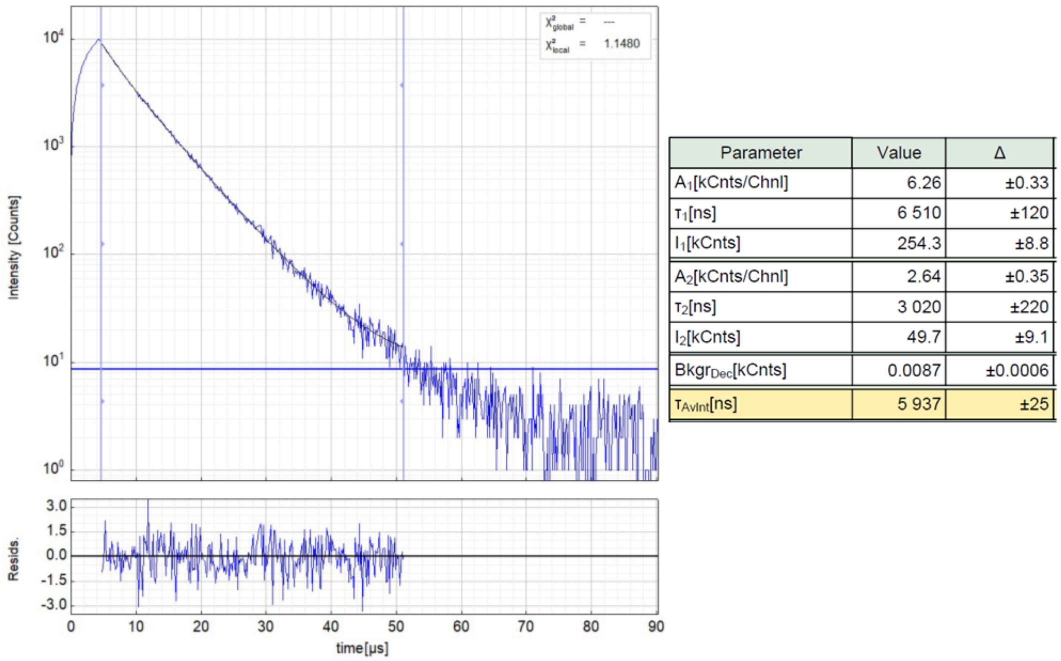

Parameter	Value	Δ
A_1[kCnts/Chnl]	6.26	±0.33
$τ_1$[ns]	6 510	±120
I_1[kCnts]	254.3	±8.8
A_2[kCnts/Chnl]	2.64	±0.35
$τ_2$[ns]	3 020	±220
I_2[kCnts]	49.7	±9.1
$Bkgr_{Dec}$[kCnts]	0.0087	±0.0006
$τ_{AvInt}$[ns]	5 937	±25

Figure S251. Left: Raw (experimental) time-resolved photoluminescence decay of **9** (10^{-5} M) in DCM at 77 K ($λ_{exc}$ = 405 nm, $λ_{em}$ = 486 nm). Right: Fitting parameters and confidence limits.

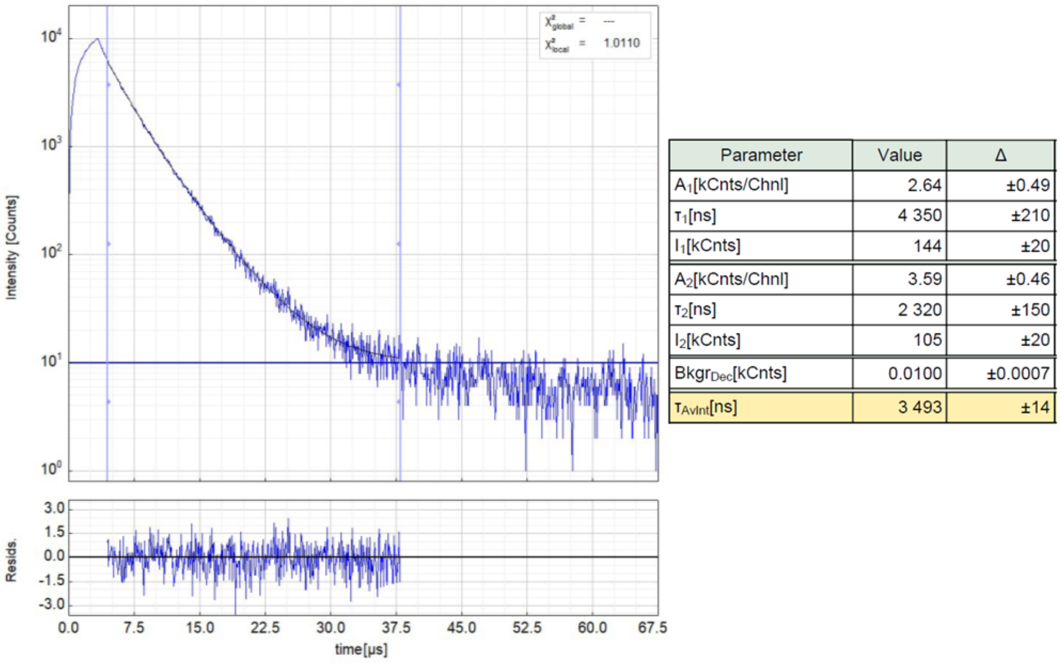

Parameter	Value	Δ
A_1[kCnts/Chnl]	2.64	±0.49
$τ_1$[ns]	4 350	±210
I_1[kCnts]	144	±20
A_2[kCnts/Chnl]	3.59	±0.46
$τ_2$[ns]	2 320	±150
I_2[kCnts]	105	±20
$Bkgr_{Dec}$[kCnts]	0.0100	±0.0007
$τ_{AvInt}$[ns]	3 493	±14

Figure S252. Left: Raw (experimental) time-resolved photoluminescence decay of **9** (10^{-5} M) in DCM at 77 K ($λ_{exc}$ = 405 nm, $λ_{em}$ = 625 nm). Right: Fitting parameters and confidence limits.

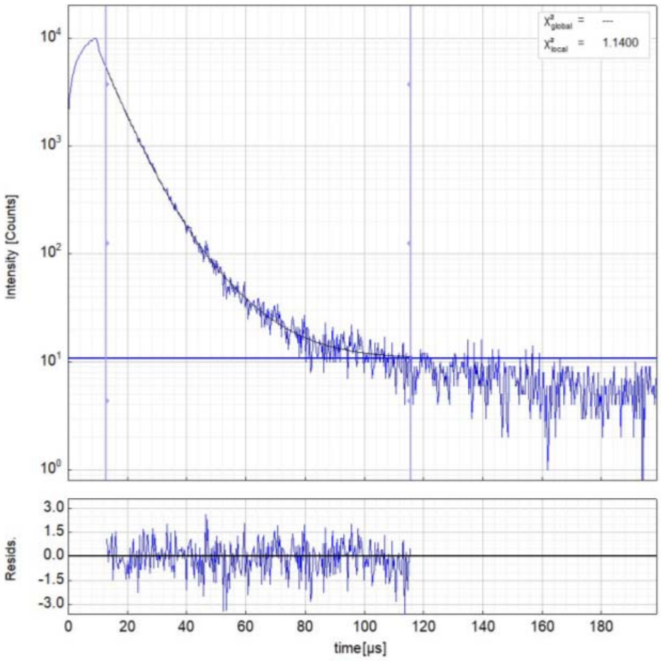

Parameter	Value	Δ
A_1[kCnts/Chnl]	0.77	±0.24
τ_1[ns]	13 800	±1 200
I_1[kCnts]	33.0	±7.4
A_2[kCnts/Chnl]	4.23	±0.26
τ_2[ns]	6 350	±210
I_2[kCnts]	83.8	±8.0
$Bkgr_{Dec}$[kCnts]	0.0107	±0.0006
τ_{AvInt}[ns]	8 433	±82

Figure S253. Left: Raw (experimental) time-resolved photoluminescence decay of **9** (10^{-6} M) in DCM at 77 K (λ_{exc} = 405 nm, λ_{em} = 498 nm). Right: Fitting parameters and confidence limits.

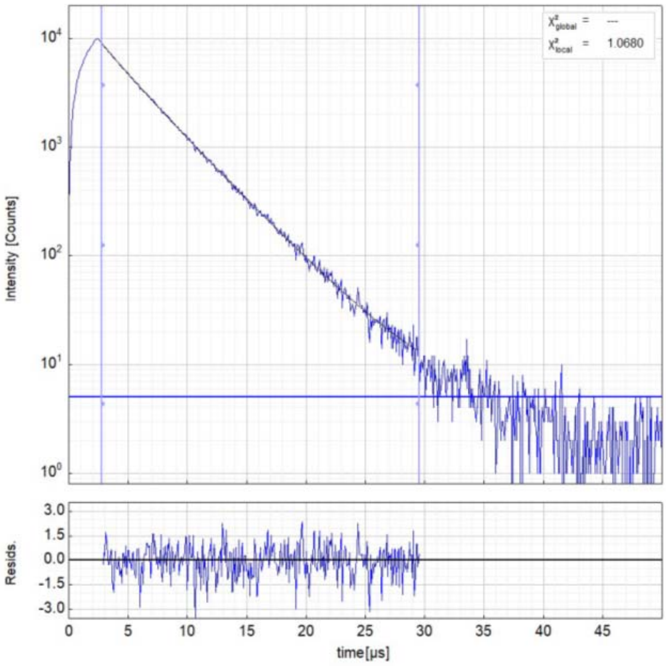

Parameter	Value	Δ
A_1[kCnts/Chnl]	6.7	±4.2
τ_1[ns]	3 980	±770
I_1[kCnts]	340	±180
A_2[kCnts/Chnl]	2.0	±4.1
τ_2[ns]	2 300	±840
I_2[kCnts]	60	±190
$Bkgr_{Dec}$[kCnts]	0.0051	±0.0037
τ_{AvInt}[ns]	3 729	±42

Figure S254. Left: Raw (experimental) time-resolved photoluminescence decay of **9** (10^{-6} M) in DCM at 77 K (λ_{exc} = 405 nm, λ_{em} = 662 nm). Right: Fitting parameters and confidence limits.

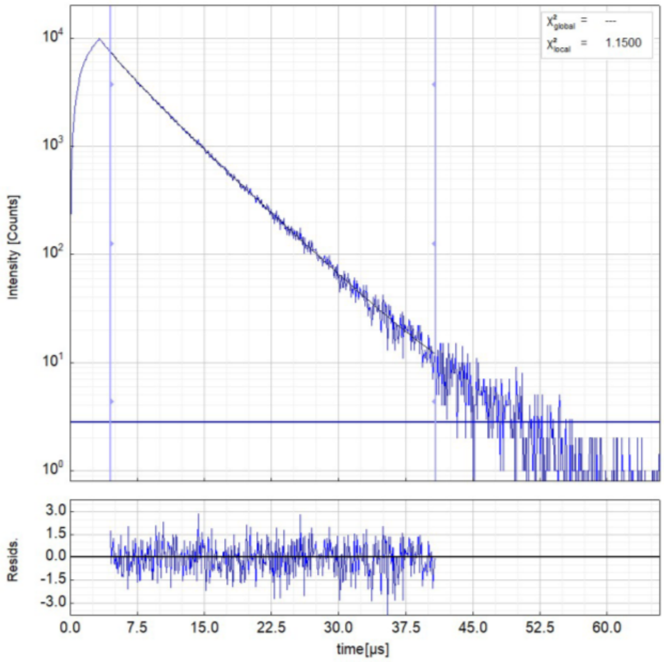

Parameter	Value	Δ
A_1[kCnts/Chnl]	5.82	±0.26
τ_1[ns]	5 634	±73
I_1[kCnts]	410	±14
A_2[kCnts/Chnl]	1.60	±0.24
τ_2[ns]	2 660	±250
I_2[kCnts]	54	±13
$Bkgr_{Dec}$[kCnts]	0.0029	±0.0009
τ_{AvInt}[ns]	5 293	±18

Figure S255. Left: Raw (experimental) time-resolved photoluminescence decay of **10** in PMMA film (2 wt%) at 298 K (λ_{exc} = 405 nm, λ_{em} = 494 nm). Right: Fitting parameters and confidence limits.

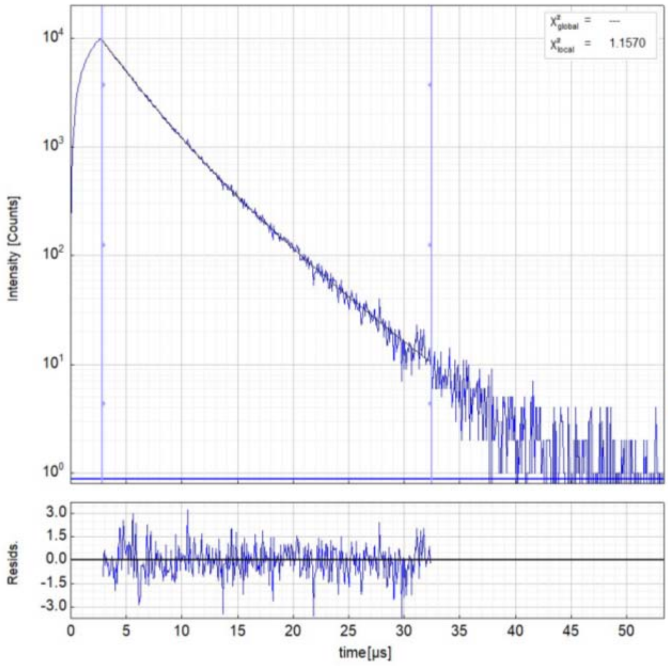

Parameter	Value	Δ
A_1[kCnts/Chnl]	2.74	±0.38
τ_1[ns]	5 210	±260
I_1[kCnts]	178	±17
A_2[kCnts/Chnl]	6.73	±0.36
τ_2[ns]	2 754	±61
I_2[kCnts]	232	±18
$Bkgr_{Dec}$[kCnts]	0.0009	±0.0020
τ_{AvInt}[ns]	3 818	±23

Figure S256. Left: Raw (experimental) time-resolved photoluminescence decay of **10** in PMMA film (2 wt%) at 298 K (λ_{exc} = 405 nm, λ_{em} = 646 nm). Right: Fitting parameters and confidence limits.

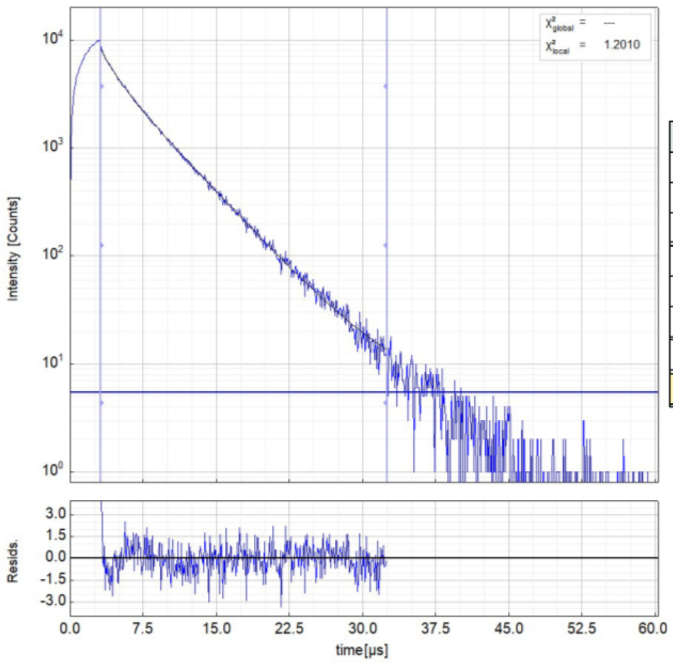

Parameter	Value	Δ
A_1[kCnts/Chnl]	5.32	±0.12
τ_1[ns]	4 517	±34
I_1[kCnts]	299.9	±4.1
A_2[kCnts/Chnl]	2.747	±0.060
τ_2[ns]	1 380	±92
I_2[kCnts]	47.4	±3.4
$Bkgr_{Dec}$[kCnts]	0.0055	±0.0011
τ_{AvInt}[ns]	4 089	±21

Figure S257. Left: Raw (experimental) time-resolved photoluminescence decay of **10** in PMMA film (5 wt%) at 298 K (λ_{exc} = 405 nm, λ_{em} = 498 nm). Right: Fitting parameters and confidence limits.

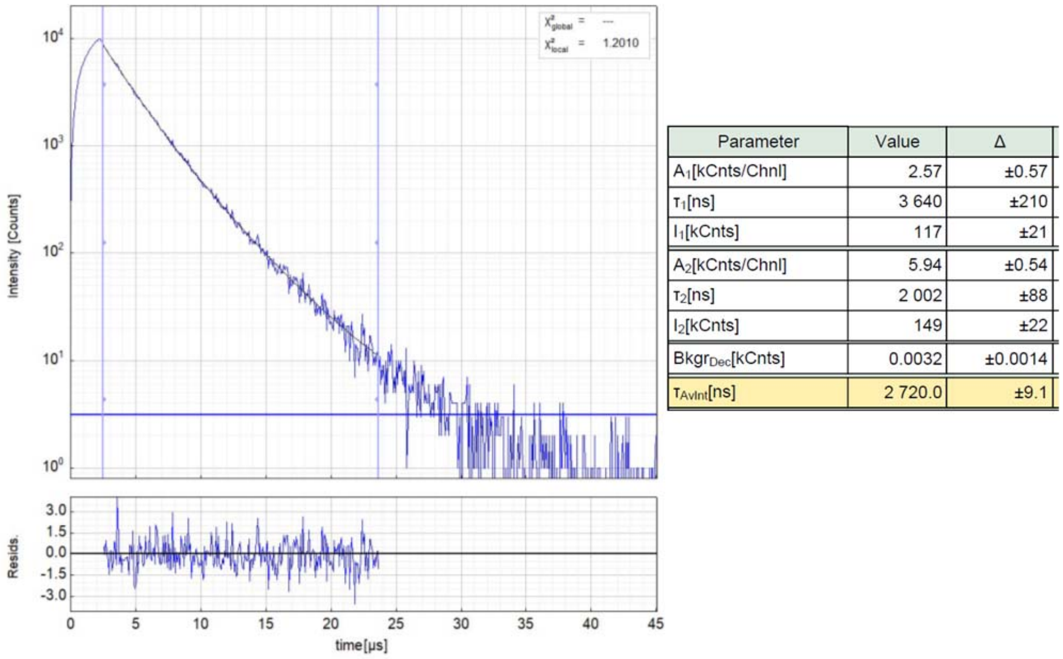

Parameter	Value	Δ
A_1[kCnts/Chnl]	2.57	±0.57
τ_1[ns]	3 640	±210
I_1[kCnts]	117	±21
A_2[kCnts/Chnl]	5.94	±0.54
τ_2[ns]	2 002	±88
I_2[kCnts]	149	±22
$Bkgr_{Dec}$[kCnts]	0.0032	±0.0014
τ_{AvInt}[ns]	2 720.0	±9.1

Figure S258. Left: Raw (experimental) time-resolved photoluminescence decay of **10** in PMMA film (5 wt%) at 298 K (λ_{exc} = 405 nm, λ_{em} = 640 nm). Right: Fitting parameters and confidence limits.

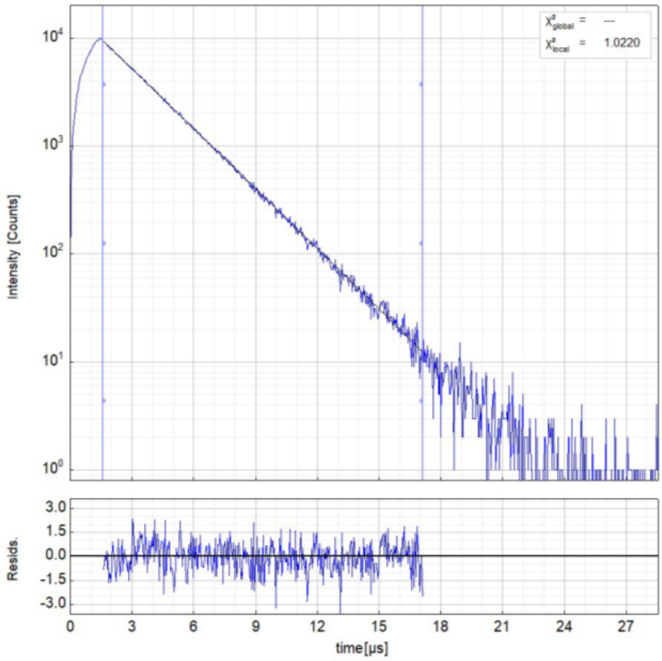

Parameter	Value	Δ
A_1[kCnts/Chnl]	9.244	±0.022
τ_1[ns]	2 349.3	±4.8
I_1[kCnts]	542.90	±0.90
$Bkgr_{Dec}$[kCnts]	-0.0005	±0.0003
τ_{AvInt}[ns]	2 349.3	±4.8

Figure S259. Left: Raw (experimental) time-resolved photoluminescence decay of **10** (10^{-3} M) in DCM at 298 K (λ_{exc} = 405 nm, λ_{em} = 494 nm). Right: Fitting parameters and confidence limits.

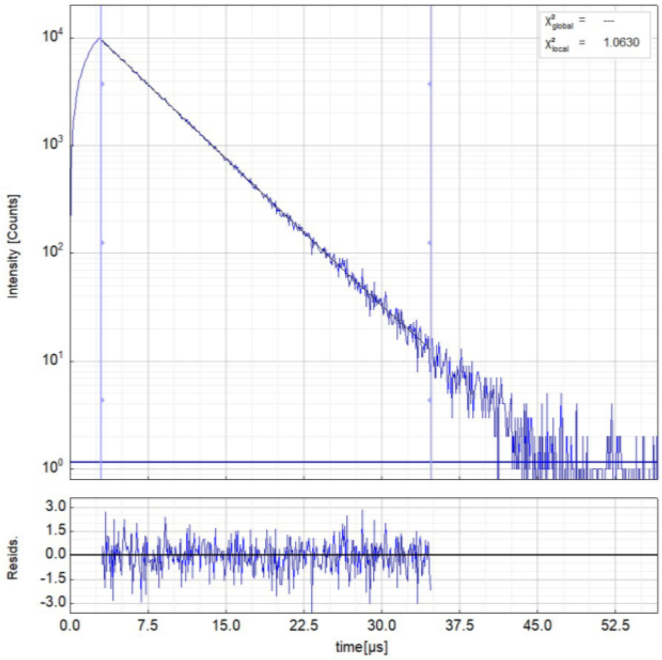

Parameter	Value	Δ
A_1[kCnts/Chnl]	9.459	±0.023
τ_1[ns]	4 730.4	±8.8
I_1[kCnts]	559.25	±0.63
$Bkgr_{Dec}$[kCnts]	0.0012	±0.0006
τ_{AvInt}[ns]	4 730.4	±8.8

Figure S260. Left: Raw (experimental) time-resolved photoluminescence decay of **10** (10^{-4} M) in DCM at 298 K (λ_{exc} = 405 nm, λ_{em} = 492 nm). Right: Fitting parameters and confidence limits.

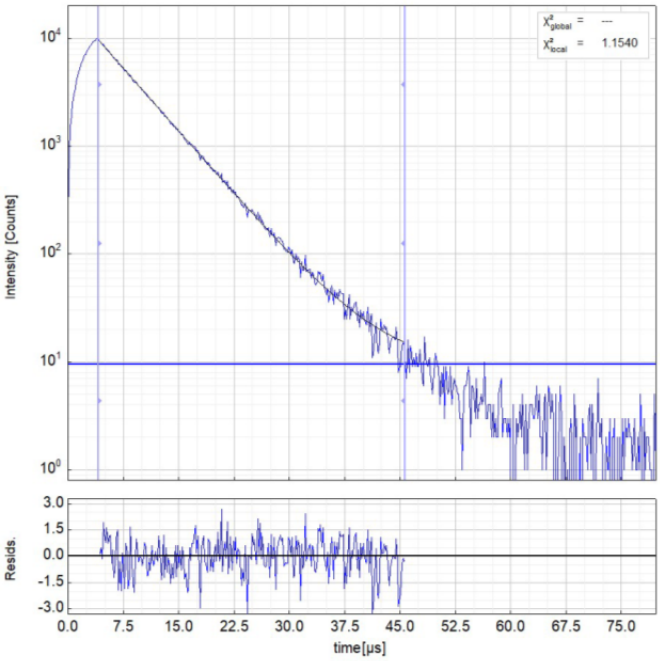

Parameter	Value	Δ
A_1[kCnts/Chnl]	9.302	±0.025
τ_1[ns]	5 584	±17
I_1[kCnts]	324.63	±0.49
$Bkgr_{Dec}$[kCnts]	0.0095	±0.0009
τ_{AvInt}[ns]	5 584	±17

Figure S261. Left: Raw (experimental) time-resolved photoluminescence decay of **10** (10^{-5} M) in DCM at 298 K (λ_{exc} = 405 nm, λ_{em} = 494 nm). Right: Fitting parameters and confidence limits.

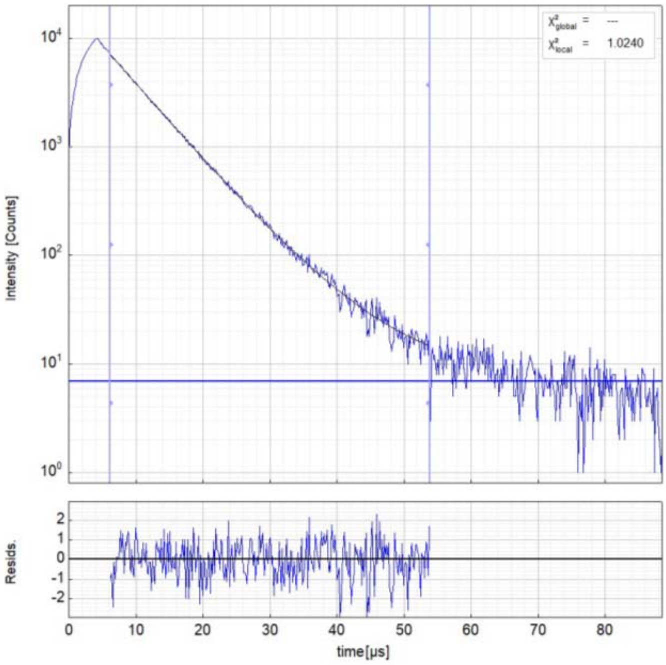

Parameter	Value	Δ
A_1[kCnts/Chnl]	0.28	±0.32
τ_1[ns]	12 000	±6 200
I_1[kCnts]	21	±16
A_2[kCnts/Chnl]	6.77	±0.33
τ_2[ns]	6 060	±120
I_2[kCnts]	256	±15
$Bkgr_{Dec}$[kCnts]	0.0070	±0.0055
τ_{AvInt}[ns]	6 490	±270

Figure S262. Left: Raw (experimental) time-resolved photoluminescence decay of **10** (10^{-6} M) in DCM at 298 K (λ_{exc} = 405 nm, λ_{em} = 494 nm). Right: Fitting parameters and confidence limits.

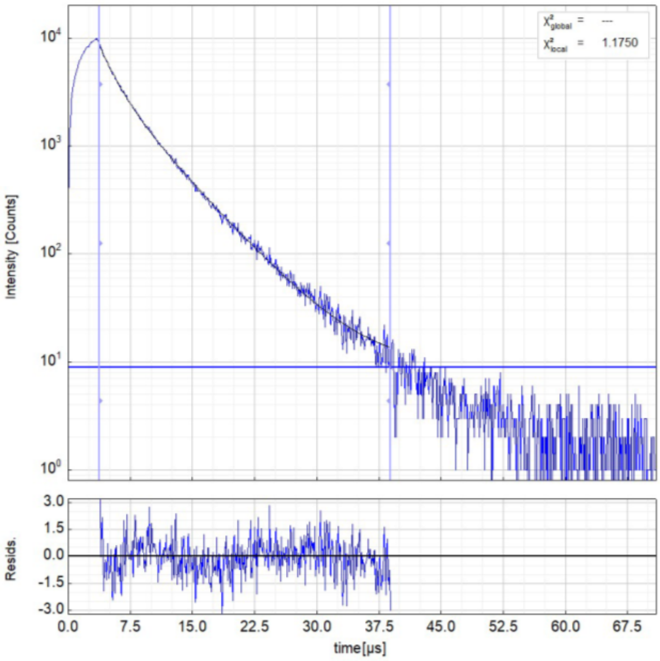

Parameter	Value	Δ
A_1[kCnts/Chnl]	3.73	±0.17
τ_1[ns]	5 238	±98
I_1[kCnts]	244.0	±6.2
A_2[kCnts/Chnl]	4.56	±0.14
τ_2[ns]	1 825	±78
I_2[kCnts]	103.9	±6.9
$Bkgr_{Dec}$[kCnts]	0.0090	±0.0009
τ_{AvInt}[ns]	4 219	±22

Figure S263. Left: Raw (experimental) time-resolved photoluminescence decay of **10** (10^{-3} M) in DCM at 77 K (λ_{exc} = 405 nm, λ_{em} = 484 nm). Right: Fitting parameters and confidence limits.

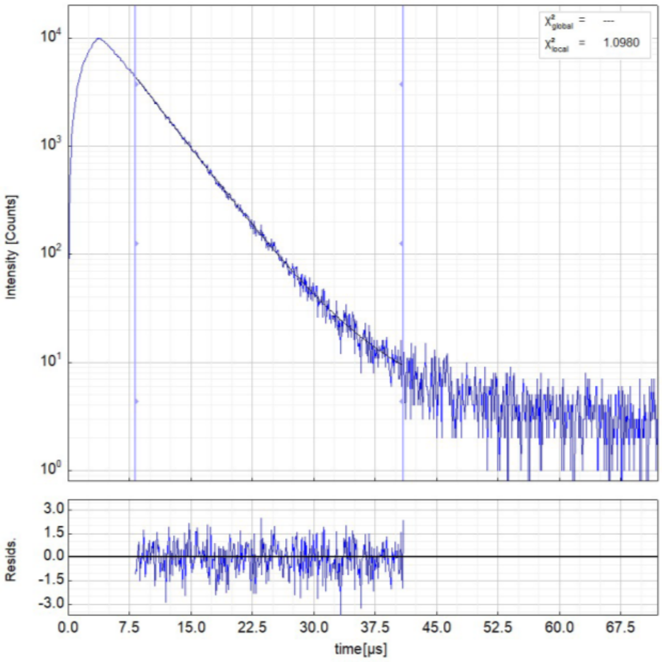

Parameter	Value	Δ
A_1[kCnts/Chnl]	0.030	±0.085
τ_1[ns]	32 000	±26 000
I_1[kCnts]	11.7	±4.1
A_2[kCnts/Chnl]	4.387	±0.013
τ_2[ns]	4 437	±21
I_2[kCnts]	243.3	±3.9
$Bkgr_{Dec}$[kCnts]	-0.0039	±0.0091
τ_{AvInt}[ns]	5 700	±1 100

Figure S264. Left: Raw (experimental) time-resolved photoluminescence decay of **10** (10^{-3} M) in DCM at 77 K (λ_{exc} = 405 nm, λ_{em} = 640 nm). Right: Fitting parameters and confidence limits.

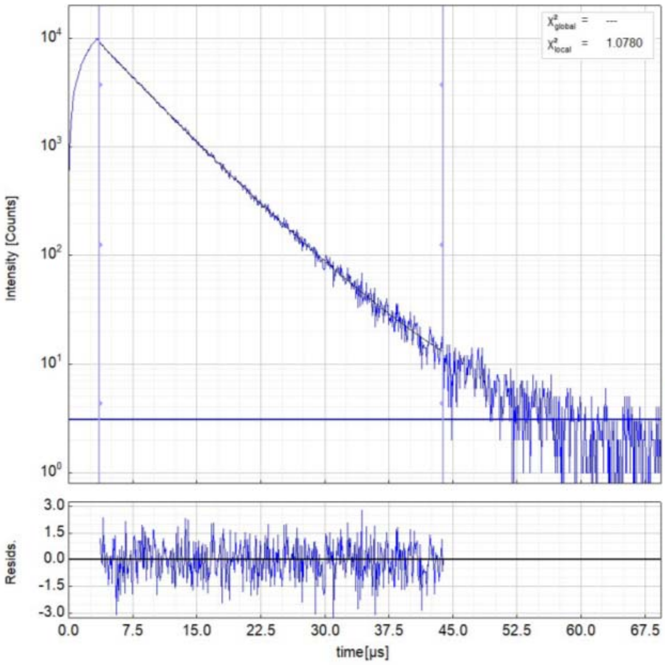

Parameter	Value	Δ
A_1[kCnts/Chnl]	2.0	±1.5
τ_1[ns]	7 400	±1 800
I_1[kCnts]	180	±120
A_2[kCnts/Chnl]	7.1	±1.5
τ_2[ns]	4 920	±280
I_2[kCnts]	440	±120
$Bkgr_{Dec}$[kCnts]	0.0031	±0.0037
τ_{AvInt}[ns]	5 602	±75

Figure S265. Left: Raw (experimental) time-resolved photoluminescence decay of **10** (10^{-4} M) in DCM at 77 K (λ_{exc} = 405 nm, λ_{em} = 484 nm). Right: Fitting parameters and confidence limits.

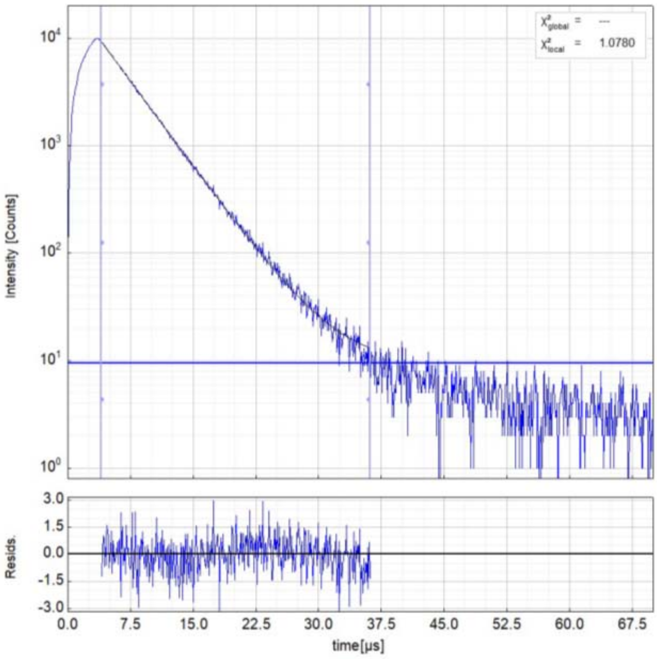

Parameter	Value	Δ
A_1[kCnts/Chnl]	9.127	±0.017
τ_1[ns]	4 108.7	±4.3
I_1[kCnts]	468.71	±0.68
$Bkgr_{Dec}$[kCnts]	0.0095	±0.0004
τ_{AvInt}[ns]	4 108.7	±4.3

Figure S266. Left: Raw (experimental) time-resolved photoluminescence decay of **10** (10^{-4} M) in DCM at 77 K (λ_{exc} = 405 nm, λ_{em} = 634 nm). Right: Fitting parameters and confidence limits.

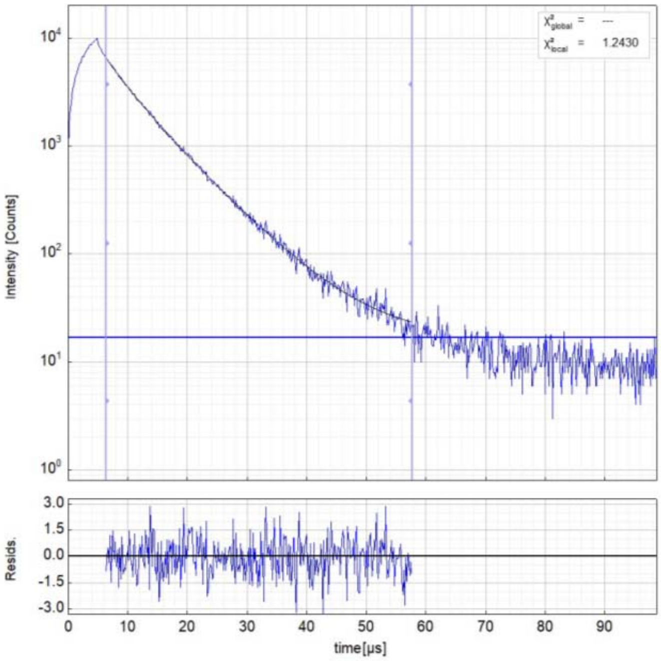

Parameter	Value	Δ
A_1[kCnts/Chnl]	4.13	±0.39
τ_1[ns]	7 950	±250
I_1[kCnts]	205	±13
A_2[kCnts/Chnl]	2.32	±0.34
τ_2[ns]	3 690	±380
I_2[kCnts]	54	±13
$Bkgr_{Dec}$[kCnts]	0.0169	±0.0016
τ_{AvInt}[ns]	7 064	±55

Figure S267. Left: Raw (experimental) time-resolved photoluminescence decay of **10** (10^{-5} M) in DCM at 77 K (λ_{exc} = 405 nm, λ_{em} = 498 nm). Right: Fitting parameters and confidence limits.

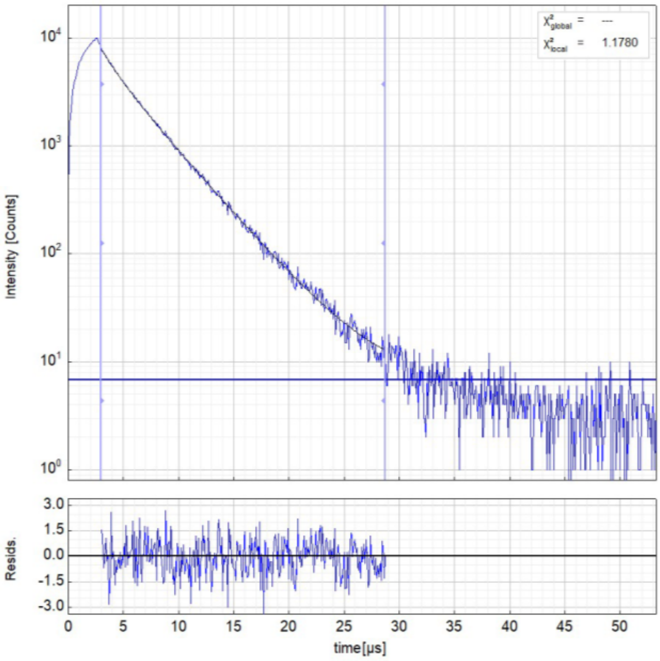

Parameter	Value	Δ
A_1[kCnts/Chnl]	5.68	±0.13
τ_1[ns]	3 750	±40
I_1[kCnts]	266.1	±2.8
A_2[kCnts/Chnl]	2.09	±0.13
τ_2[ns]	1 428	±94
I_2[kCnts]	37.2	±4.3
$Bkgr_{Dec}$[kCnts]	0.0068	±0.0008
τ_{AvInt}[ns]	3 465	±13

Figure S268. Left: Raw (experimental) time-resolved photoluminescence decay of **10** (10^{-5} M) in DCM at 77 K (λ_{exc} = 405 nm, λ_{em} = 648 nm). Right: Fitting parameters and confidence limits.

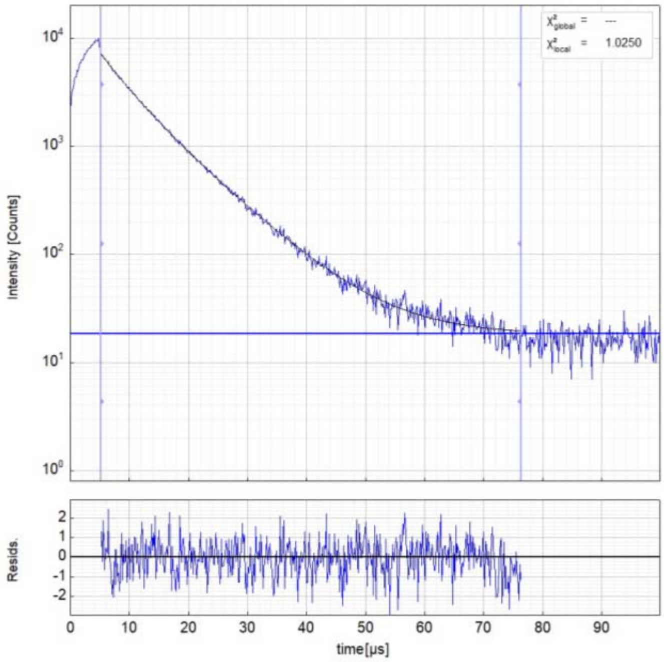

Parameter	Value	Δ
A_1[kCnts/Chnl]	4.31	±0.28
τ_1[ns]	8 760	±180
I_1[kCnts]	235.8	±9.9
A_2[kCnts/Chnl]	2.70	±0.23
τ_2[ns]	4 080	±230
I_2[kCnts]	69	±11
$Bkgr_{Dec}$[kCnts]	0.0184	±0.0006
τ_{AvInt}[ns]	7 699	±16

Figure S269. Left: Raw (experimental) time-resolved photoluminescence decay of **10** (10^{-6} M) in DCM at 77 K (λ_{exc} = 405 nm, λ_{em} = 493 nm). Right: Fitting parameters and confidence limits.

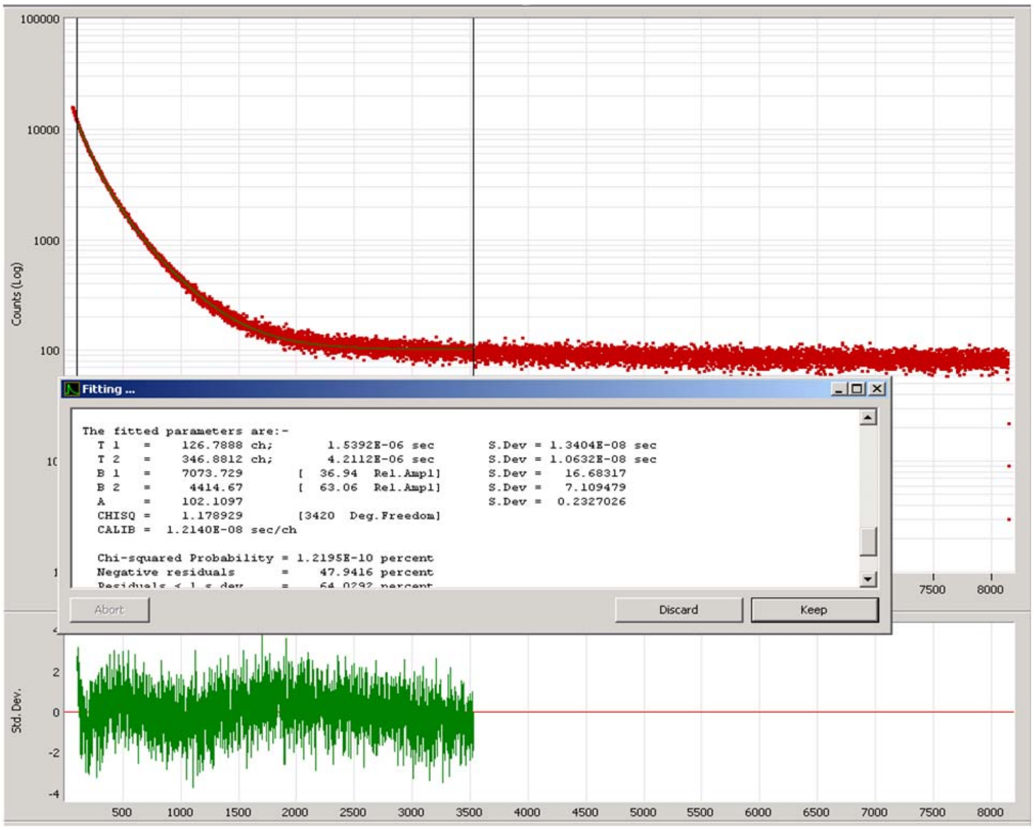

Figure S270. Left: Raw (experimental) time-resolved photoluminescence decay of **16** in PMMA film (5 wt%) at 298 K (λ_{exc} = 390 nm, λ_{em} = 490 nm). Right: Fitting parameters and confidence limits.

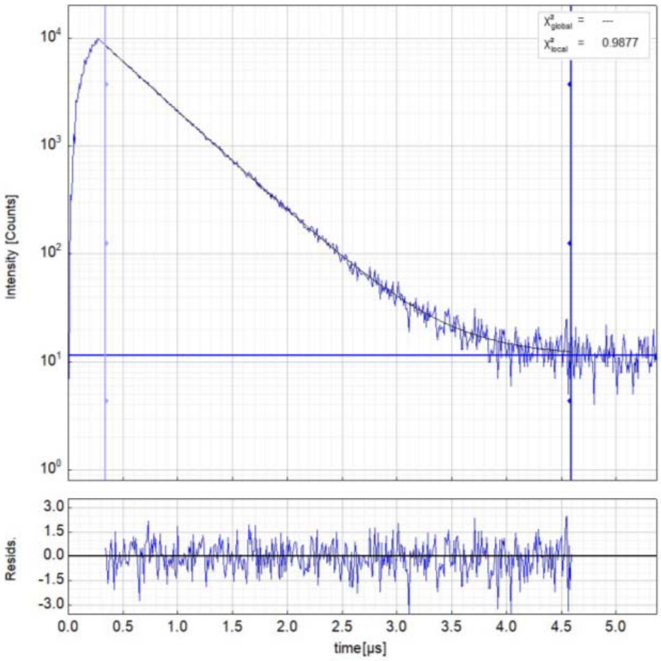

Parameter	Value	Δ
A_1[kCnts/Chnl]	8.519	±0.022
τ_1[ns]	468.32	±0.79
I_1[kCnts]	398.95	±0.65
$Bkgr_{Dec}$[kCnts]	0.0115	±0.0003
τ_{AvInt}[ns]	468.32	±0.79

Figure S271. Left: Raw (experimental) time-resolved photoluminescence decay of **16** (10^{-3} M) in DCM at 298 K (λ_{exc} = 378 nm, λ_{em} = 490 nm). Right: Fitting parameters and confidence limits.

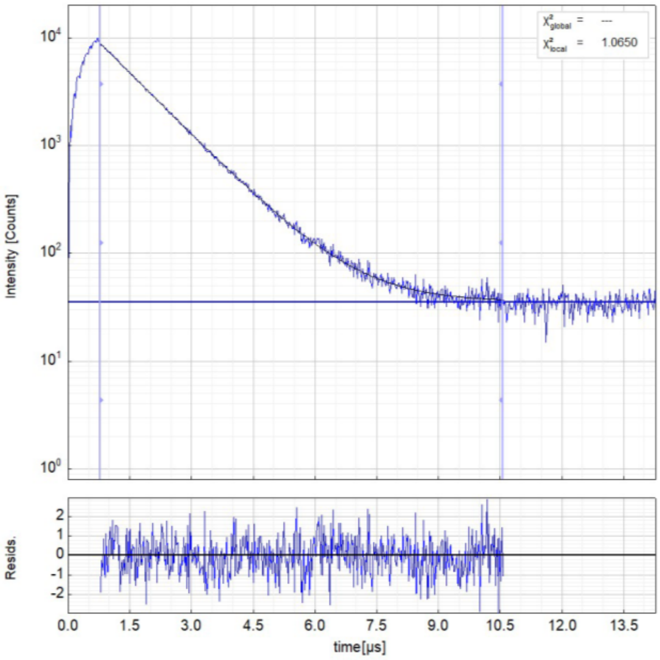

Parameter	Value	Δ
A_1[kCnts/Chnl]	8.689	±0.023
τ_1[ns]	1 131.5	±2.4
I_1[kCnts]	491.54	±0.55
$Bkgr_{Dec}$[kCnts]	0.0356	±0.0004
τ_{AvInt}[ns]	1 131.5	±2.4

Figure S272. Left: Raw (experimental) time-resolved photoluminescence decay of **16** (10^{-4} M) in DCM at 298 K (λ_{exc} = 378 nm, λ_{em} = 490 nm). Right: Fitting parameters and confidence limits.

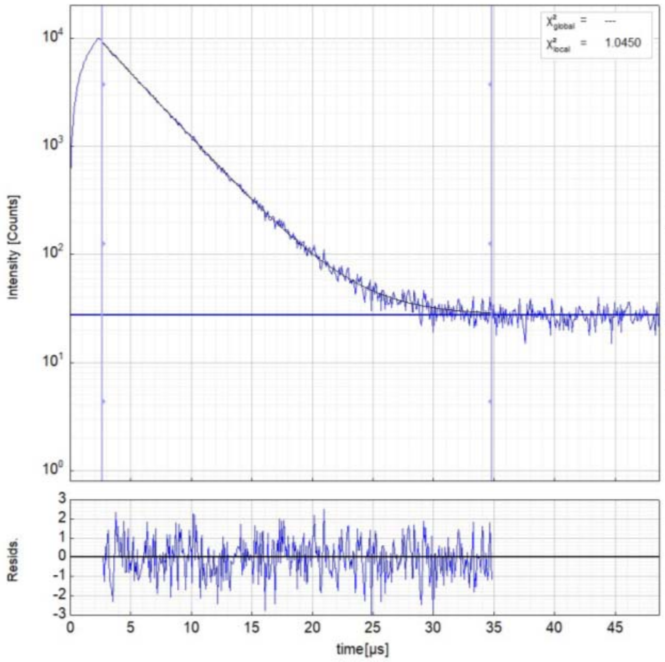

Parameter	Value	Δ
A_1[kCnts/Chnl]	8.885	±0.024
τ_1[ns]	3 607	±11
I_1[kCnts]	400.49	±0.86
$Bkgr_{Dec}$[kCnts]	0.0274	±0.0008
τ_{AvInt}[ns]	3 607	±11

Figure S273. Left: Raw (experimental) time-resolved photoluminescence decay of **16** (10^{-5} M) in DCM at 298 K (λ_{exc} = 378 nm, λ_{em} = 491 nm). Right: Fitting parameters and confidence limits.

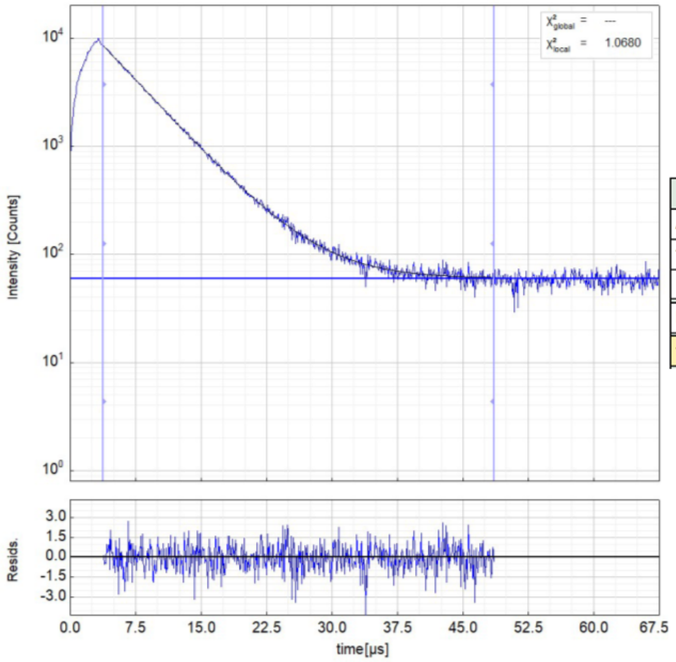

Parameter	Value	Δ
A_1[kCnts/Chnl]	8.372	±0.021
τ_1[ns]	5 003	±12
I_1[kCnts]	523.56	±0.51
$Bkgr_{Dec}$[kCnts]	0.0599	±0.0010
τ_{AvInt}[ns]	5 003	±12

Figure S274. Left: Raw (experimental) time-resolved photoluminescence decay of **16** (10^{-6} M) in DCM at 298 K (λ_{exc} = 378 nm, λ_{em} = 491 nm). Right: Fitting parameters and confidence limits.

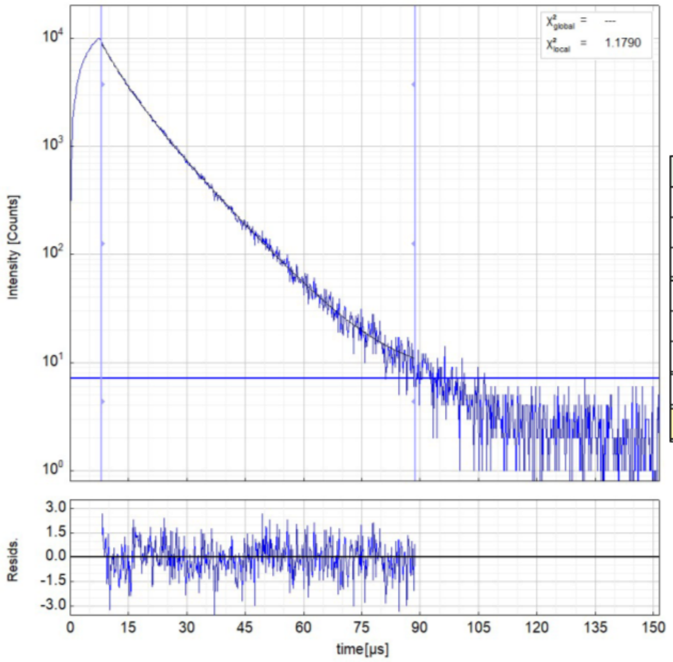

Parameter	Value	Δ
A_1[kCnts/Chnl]	4.81	±0.20
τ_1[ns]	11 240	±150
I_1[kCnts]	337.1	±9.4
A_2[kCnts/Chnl]	4.00	±0.12
τ_2[ns]	4 730	±250
I_2[kCnts]	119	±11
$Bkgr_{Dec}$[kCnts]	0.0072	±0.0007
τ_{AvInt}[ns]	9 544	±39

Figure S275. Left: Raw (experimental) time-resolved photoluminescence decay of **16** (10^{-3} M) in DCM at 77 K (λ_{exc} = 405 nm, λ_{em} = 497 nm). Right: Fitting parameters and confidence limits.

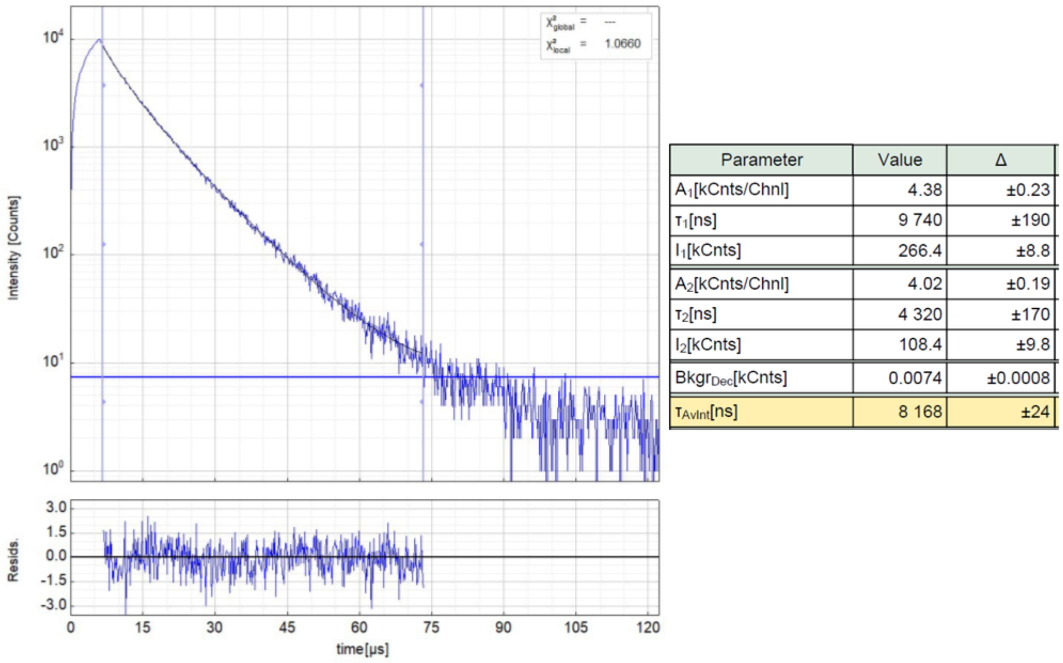

Parameter	Value	Δ
A_1[kCnts/Chnl]	4.38	±0.23
τ_1[ns]	9 740	±190
I_1[kCnts]	266.4	±8.8
A_2[kCnts/Chnl]	4.02	±0.19
τ_2[ns]	4 320	±170
I_2[kCnts]	108.4	±9.8
$Bkgr_{Dec}$[kCnts]	0.0074	±0.0008
τ_{AvInt}[ns]	8 168	±24

Figure S276. Left: Raw (experimental) time-resolved photoluminescence decay of **16** (10^{-4} M) in DCM at 77 K (λ_{exc} = 378 nm, λ_{em} = 485 nm). Right: Fitting parameters and confidence limits.

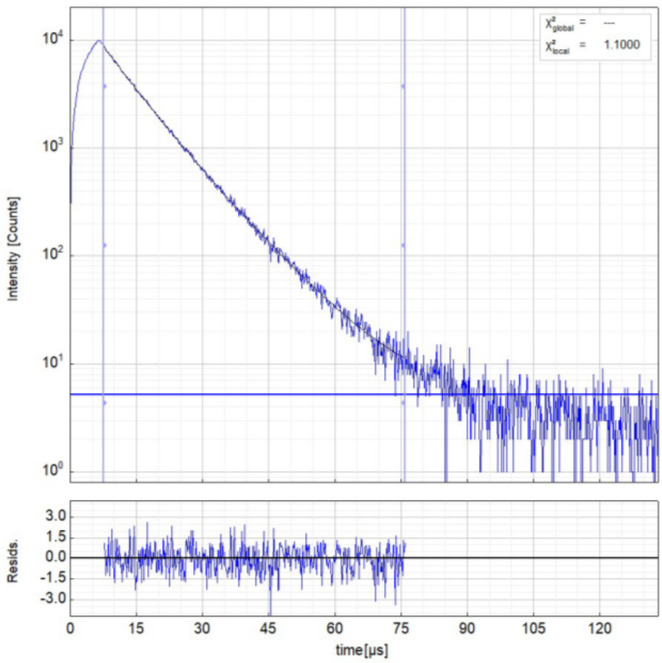

Parameter	Value	Δ
A_1[kCnts/Chnl]	4.2	±1.6
τ_1[ns]	10 410	±980
I_1[kCnts]	273	±81
A_2[kCnts/Chnl]	4.5	±1.6
τ_2[ns]	6 450	±540
I_2[kCnts]	179	±81
$Bkgr_{Dec}$[kCnts]	0.0052	±0.0021
τ_{AvInt}[ns]	8 839	±66

Figure S277. Left: Raw (experimental) time-resolved photoluminescence decay of **16** (10^{-5} M) in DCM at 77 K (λ_{exc} = 378 nm, λ_{em} = 482 nm). Right: Fitting parameters and confidence limits.

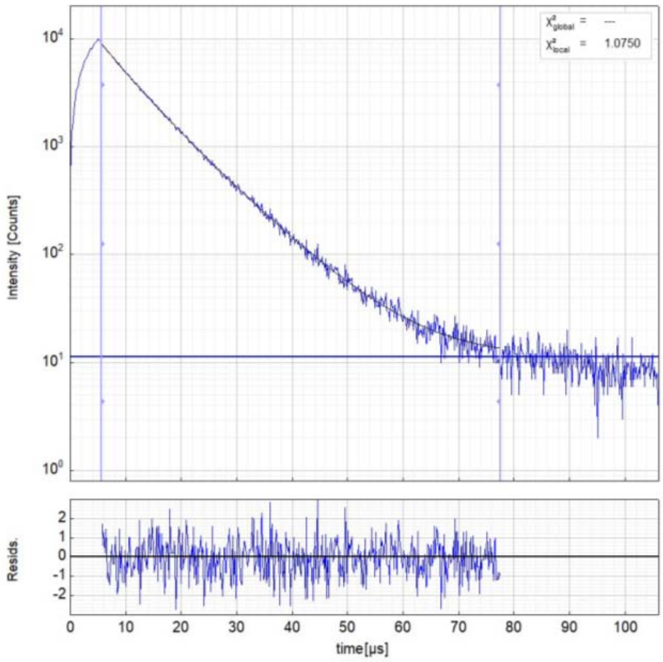

Parameter	Value	Δ
A_1[kCnts/Chnl]	4.95	±0.45
τ_1[ns]	9 370	±200
I_1[kCnts]	290	±21
A_2[kCnts/Chnl]	3.85	±0.47
τ_2[ns]	5 300	±260
I_2[kCnts]	128	±21
$Bkgr_{Dec}$[kCnts]	0.0113	±0.0005
τ_{AvInt}[ns]	8 125	±16

Figure S278. Left: Raw (experimental) time-resolved photoluminescence decay of **16** (10^{-6} M) in DCM at 77 K (λ_{exc} = 378 nm, λ_{em} = 481 nm). Right: Fitting parameters and confidence limits.

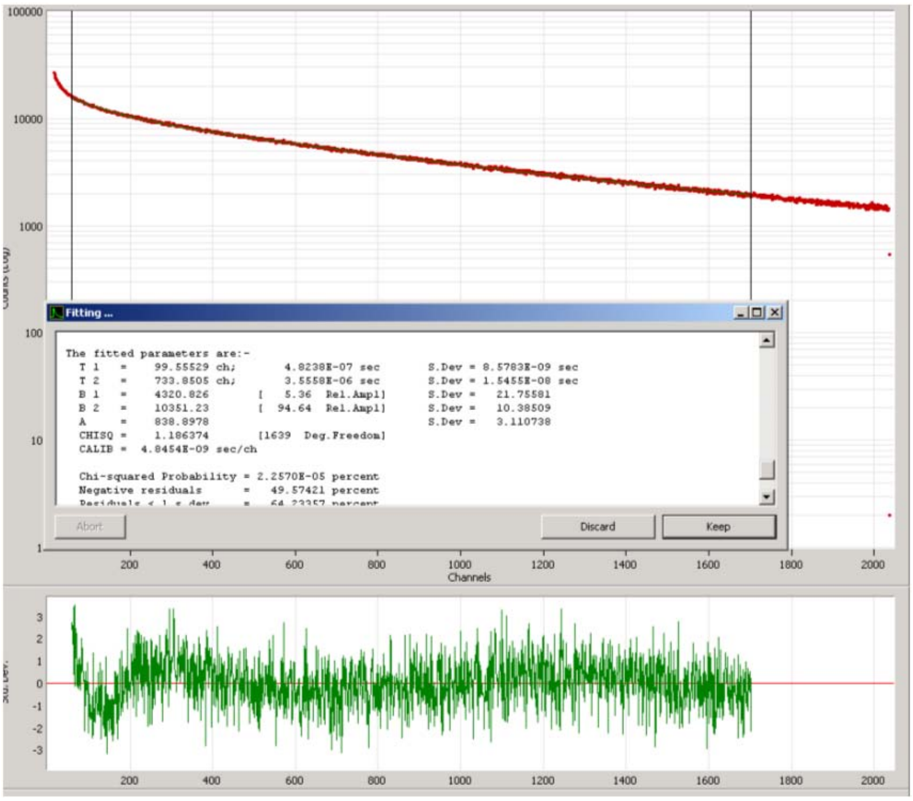

Figure S279. Left: Raw (experimental) time-resolved photoluminescence decay of **17** in PMMA film (5 wt%) at 298 K (λ_{exc} = 390 nm, λ_{em} = 495 nm). Right: Fitting parameters and confidence limits.

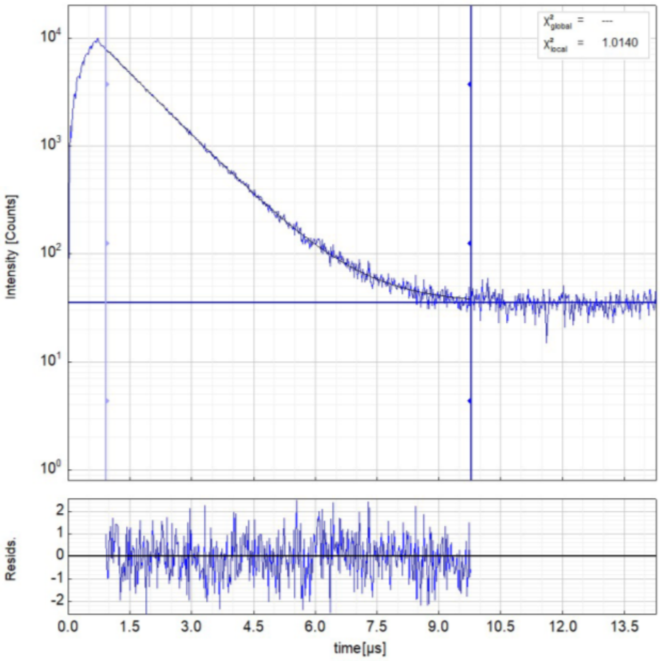

Parameter	Value	Δ
A_1[kCnts/Chnl]	7.826	±0.015
τ_1[ns]	1 131.0	±2.0
I_1[kCnts]	442.55	±0.76
$Bkgr_{Dec}$[kCnts]	0.0352	±0.0008
τ_{AvInt}[ns]	1 131.0	±2.0

Figure S280. Left: Raw (experimental) time-resolved photoluminescence decay of **17** (10^{-4} M) in DCM at 298 K (λ_{exc} = 378 nm, λ_{em} = 494 nm). Right: Fitting parameters and confidence limits.

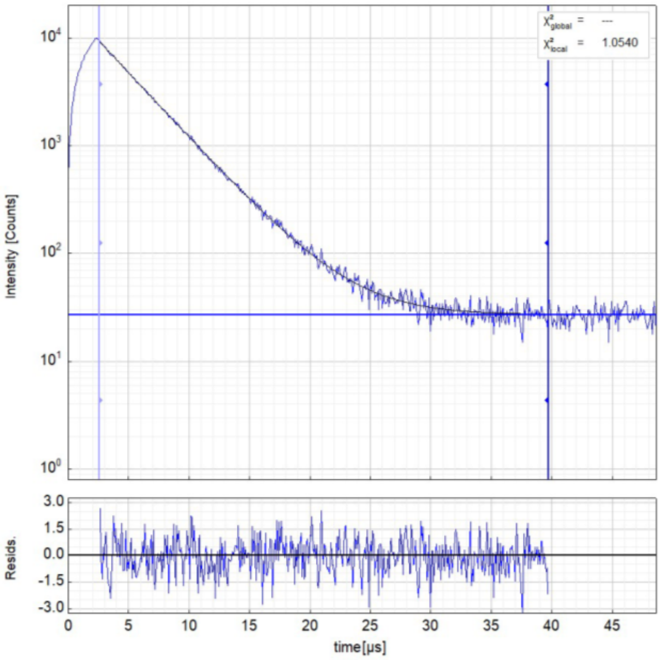

Parameter	Value	Δ
A_1[kCnts/Chnl]	9.094	±0.023
τ_1[ns]	3 605.7	±6.4
I_1[kCnts]	409.83	±0.74
$Bkgr_{Dec}$[kCnts]	0.0271	±0.0004
τ_{AvInt}[ns]	3 605.7	±6.4

Figure S281. Left: Raw (experimental) time-resolved photoluminescence decay of **17** (10^{-5} M) in DCM at 298 K (λ_{exc} = 378 nm, λ_{em} = 494 nm). Right: Fitting parameters and confidence limits.

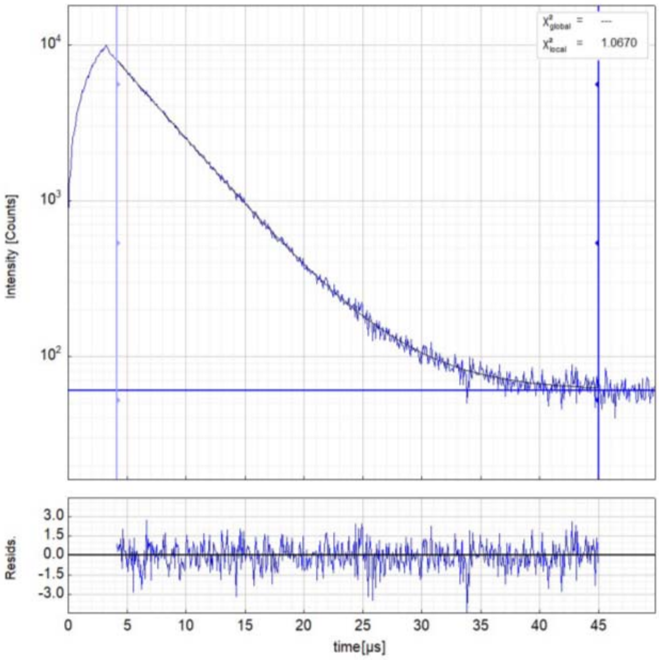

Parameter	Value	Δ
A_1[kCnts/Chnl]	7.860	±0.015
τ_1[ns]	4 998	±13
I_1[kCnts]	491.05	±0.59
$Bkgr_{Dec}$[kCnts]	0.0604	±0.0005
τ_{AvInt}[ns]	4 998	±13

Figure S282. Left: Raw (experimental) time-resolved photoluminescence decay of **17** (10^{-6} M) in DCM at 298 K (λ_{exc} = 378 nm, λ_{em} = 494 nm). Right: Fitting parameters and confidence limits.

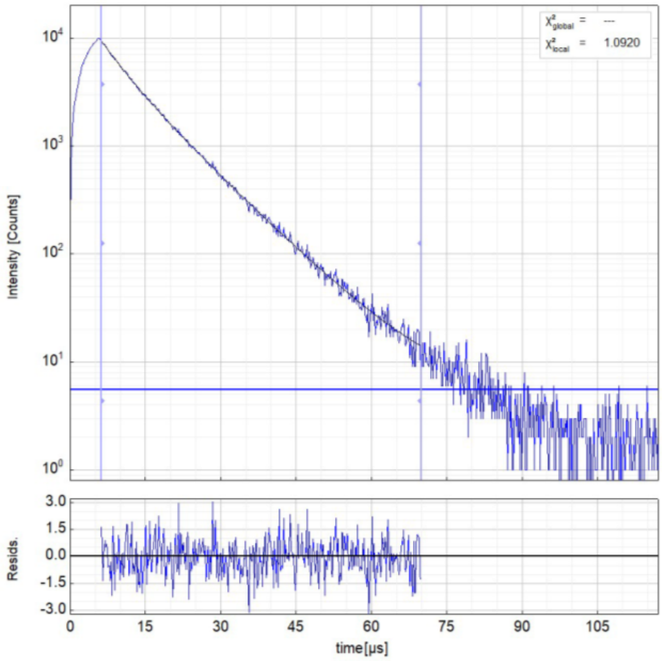

Parameter	Value	Δ
A_1[kCnts/Chnl]	5.82	±0.27
τ_1[ns]	9 760	±140
I_1[kCnts]	355	±12
A_2[kCnts/Chnl]	3.55	±0.24
τ_2[ns]	4 520	±200
I_2[kCnts]	101	±12
$Bkgr_{Dec}$[kCnts]	0.0055	±0.0010
τ_{AvInt}[ns]	8 601	±23

Figure S283. Left: Raw (experimental) time-resolved photoluminescence decay of **17** (10^{-4} M) in DCM at 77 K (λ_{exc} = 378 nm, λ_{em} = 498 nm). Right: Fitting parameters and confidence limits.

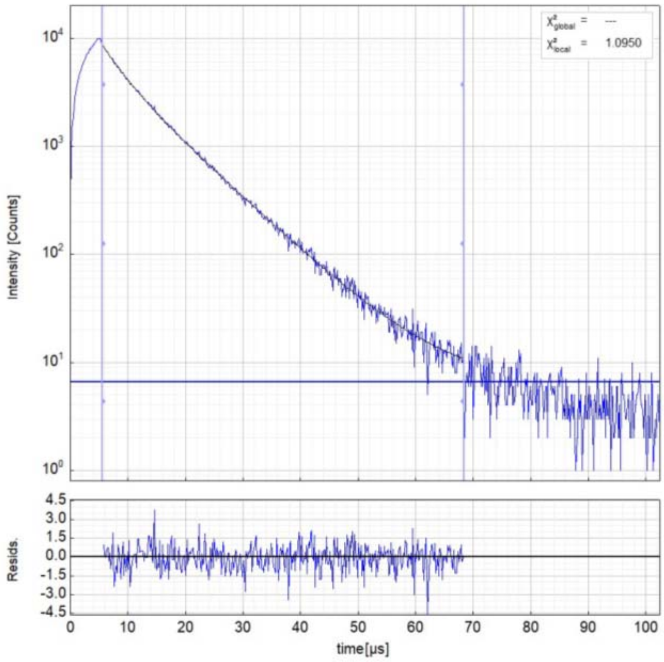

Parameter	Value	Δ
A_1[kCnts/Chnl]	5.22	±0.12
τ_1[ns]	8 806	±76
I_1[kCnts]	287.1	±4.4
A_2[kCnts/Chnl]	3.098	±0.066
τ_2[ns]	3 390	±150
I_2[kCnts]	65.6	±4.1
$Bkgr_{Dec}$[kCnts]	0.0066	±0.0005
τ_{AvInt}[ns]	7 798	±29

Figure S284. Left: Raw (experimental) time-resolved photoluminescence decay of **17** (10^{-5} M) in DCM at 77 K (λ_{exc} = 378 nm, λ_{em} = 496 nm). Right: Fitting parameters and confidence limits.

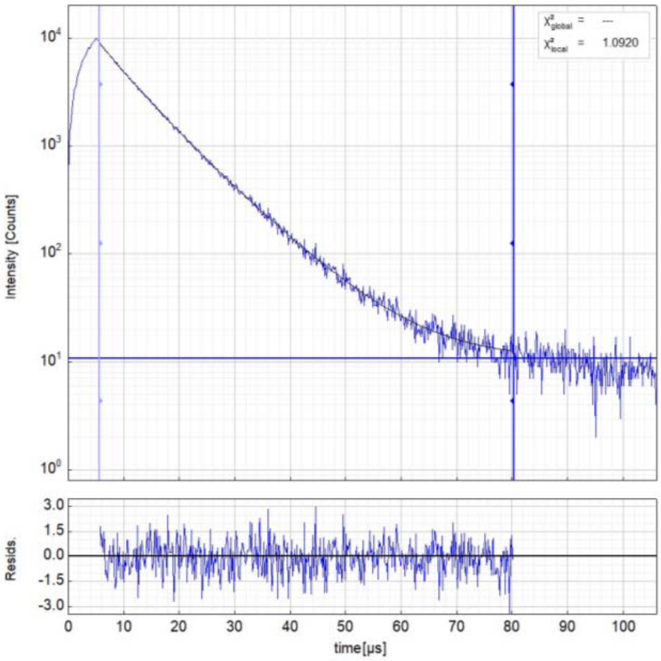

Parameter	Value	Δ
A_1[kCnts/Chnl]	4.66	±0.75
τ_1[ns]	9 510	±340
I_1[kCnts]	277	±33
A_2[kCnts/Chnl]	4.13	±0.73
τ_2[ns]	5 450	±430
I_2[kCnts]	141	±34
$Bkgr_{Dec}$[kCnts]	0.0108	±0.0009
τ_{AvInt}[ns]	8 138	±32

Figure S285. Left: Raw (experimental) time-resolved photoluminescence decay of **17** (10^{-6} M) in DCM at 77 K (λ_{exc} = 378 nm, λ_{em} = 484 nm). Right: Fitting parameters and confidence limits.

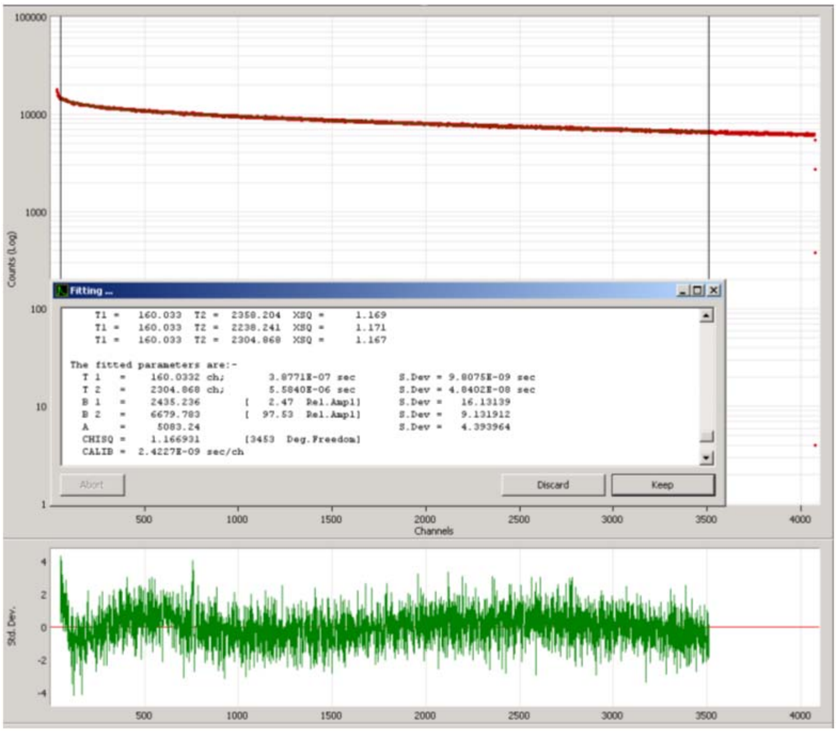

Figure S286. Left: Raw (experimental) time-resolved photoluminescence decay of **18** in PMMA film (5 wt%) at 298 K (λ_{exc} = 390 nm, λ_{em} = 511 nm). Right: Fitting parameters and confidence limits.

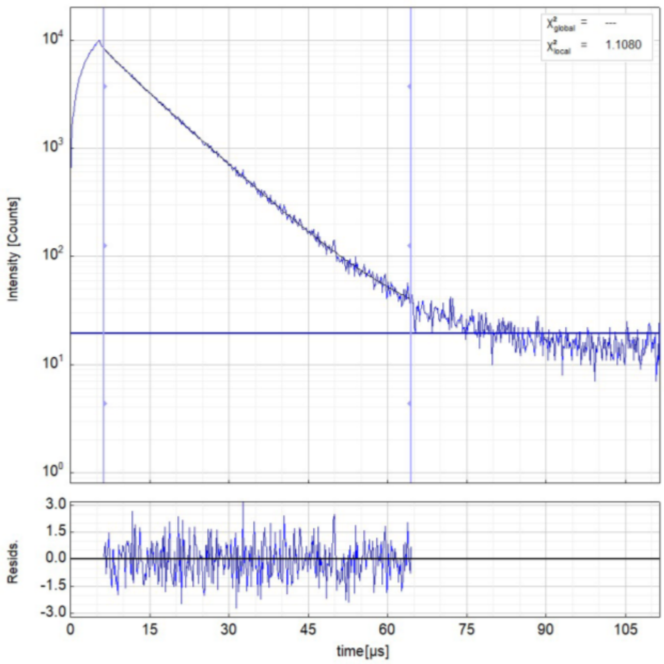

Parameter	Value	Δ
A_1[kCnts/Chnl]	7.54	±0.15
τ_1[ns]	9 866	±79
I_1[kCnts]	464.5	±5.8
A_2[kCnts/Chnl]	0.68	±0.15
τ_2[ns]	3 730	±610
I_2[kCnts]	15.8	±4.9
$Bkgr_{Dec}$[kCnts]	0.0193	±0.0025
τ_{AvInt}[ns]	9 664	±40

Figure S287. Left: Raw (experimental) time-resolved photoluminescence decay of **18** (10^{-3} M) in DCM at 298 K (λ_{exc} = 378 nm, λ_{em} = 520 nm). Right: Fitting parameters and confidence limits.

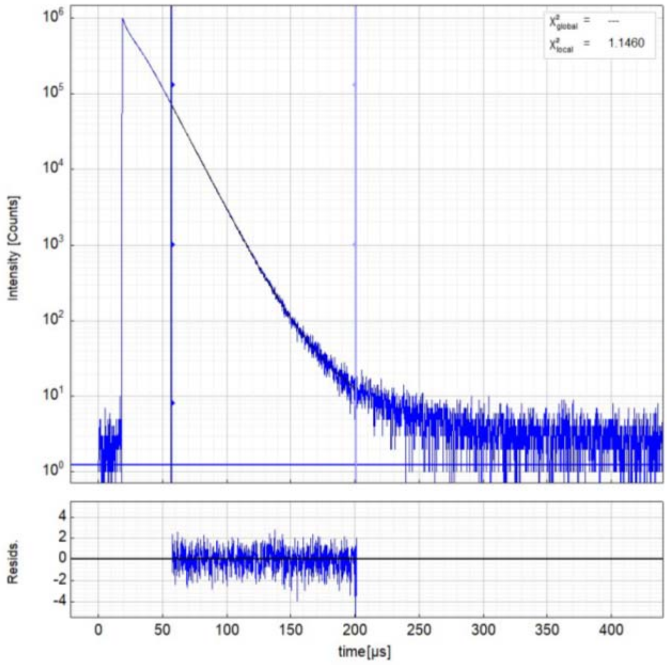

Parameter	Value	Δ
A_1[kCnts/Chnl]	0.49	±0.13
τ_1[ns]	36 700	±7 200
I_1[kCnts]	111	±15
A_2[kCnts/Chnl]	68.963	±0.085
τ_2[ns]	13 395	±26
I_2[kCnts]	5 774	±16
$Bkgr_{Dec}$[kCnts]	0.0013	±0.0048
τ_{AvInt}[ns]	13 832	±82

Figure S288. Left: Raw (experimental) time-resolved photoluminescence decay of **18** (10^{-4} M) in DCM at 298 K (λ_{exc} = 378 nm, λ_{em} = 518 nm). Right: Fitting parameters and confidence limits.

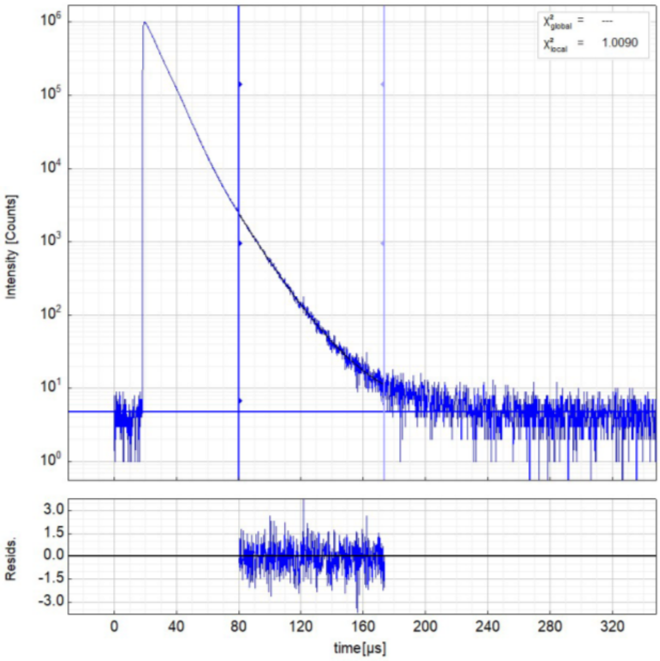

Parameter	Value	Δ
A_1[kCnts/Chnl]	0.30	±0.26
τ_1[ns]	23 400	±7 000
I_1[kCnts]	43	±23
A_2[kCnts/Chnl]	2.09	±0.27
τ_2[ns]	12 220	±590
I_2[kCnts]	160	±27
$Bkgr_{Dec}$[kCnts]	0.0048	±0.0025
τ_{AvInt}[ns]	14 580	±410

Figure S289. Left: Raw (experimental) time-resolved photoluminescence decay of **18** (10^{-5} M) in DCM at 298 K (λ_{exc} = 378 nm, λ_{em} = 495 nm). Right: Fitting parameters and confidence limits.

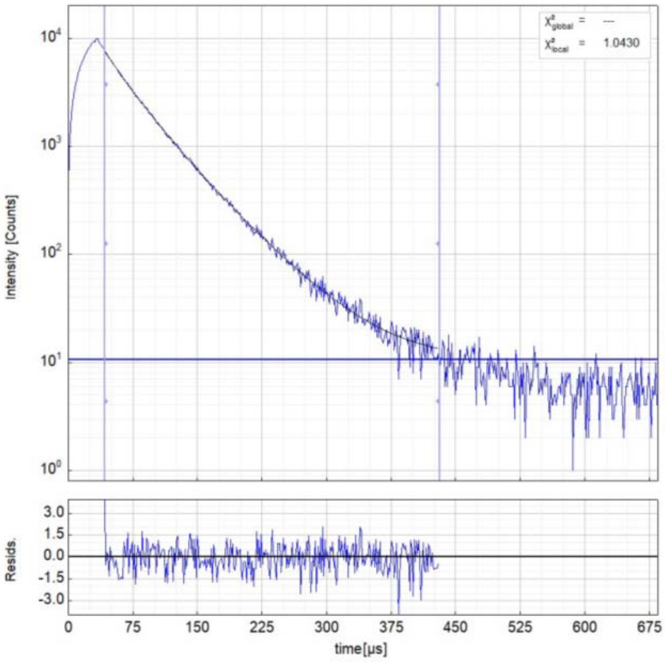

Parameter	Value	Δ
A_1[kCnts/Chnl]	3.96	±0.63
τ_1[ns]	53 600	±2 200
I_1[kCnts]	166	±21
A_2[kCnts/Chnl]	3.61	±0.61
τ_2[ns]	27 100	±2 900
I_2[kCnts]	77	±21
$Bkgr_{Dec}$[kCnts]	0.0107	±0.0010
τ_{AvInt}[ns]	45 220	±180

Figure S290. Left: Raw (experimental) time-resolved photoluminescence decay of **18** (10^{-3} M) in DCM at 77 K (λ_{exc} = 378 nm, λ_{em} = 509 nm). Right: Fitting parameters and confidence limits.

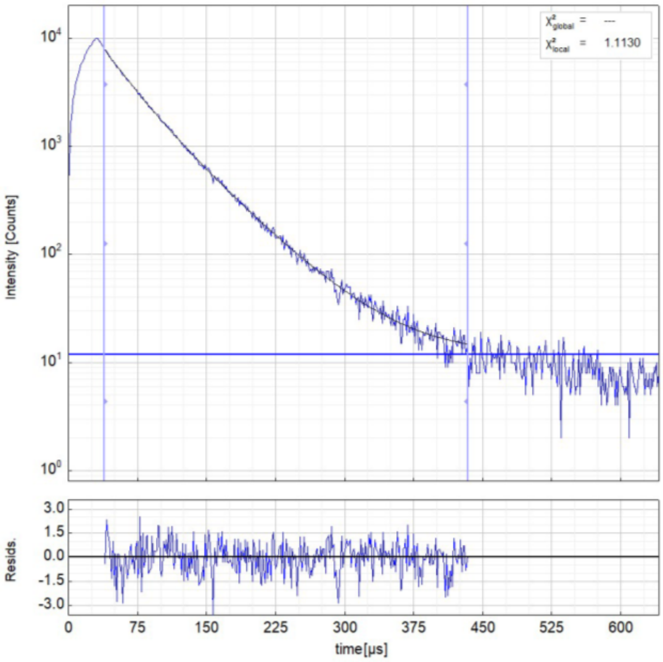

Parameter	Value	Δ
A_1[kCnts/Chnl]	3.90	±0.62
τ_1[ns]	54 900	±2 000
I_1[kCnts]	168	±19
A_2[kCnts/Chnl]	3.80	±0.56
τ_2[ns]	28 700	±2 300
I_2[kCnts]	85	±20
$Bkgr_{Dec}$[kCnts]	0.0118	±0.0007
τ_{AvInt}[ns]	46 010	±190

Figure S291. Left: Raw (experimental) time-resolved photoluminescence decay of **18** (10^{-4} M) in DCM at 77 K (λ_{exc} = 378 nm, λ_{em} = 508 nm). Right: Fitting parameters and confidence limits.

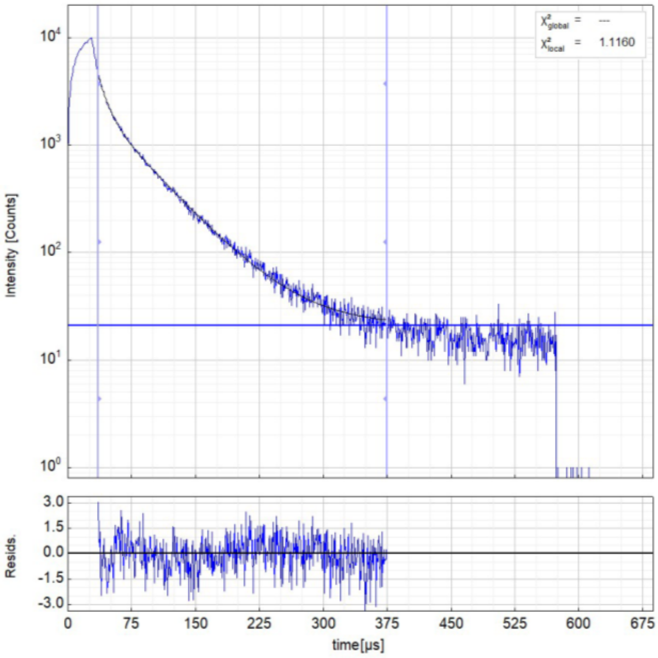

Parameter	Value	Δ
A_1[kCnts/Chnl]	1.983	±0.033
τ_1[ns]	50 960	±380
I_1[kCnts]	157.9	±1.6
A_2[kCnts/Chnl]	2.430	±0.062
τ_2[ns]	10 120	±460
I_2[kCnts]	38.5	±1.2
$Bkgr_{Dec}$[kCnts]	0.0210	±0.0004
τ_{AvInt}[ns]	42 970	±160

Figure S292. Left: Raw (experimental) time-resolved photoluminescence decay of **18** (10^{-5} M) in DCM at 77 K (λ_{exc} = 378 nm, λ_{em} = 490 nm). Right: Fitting parameters and confidence limits.

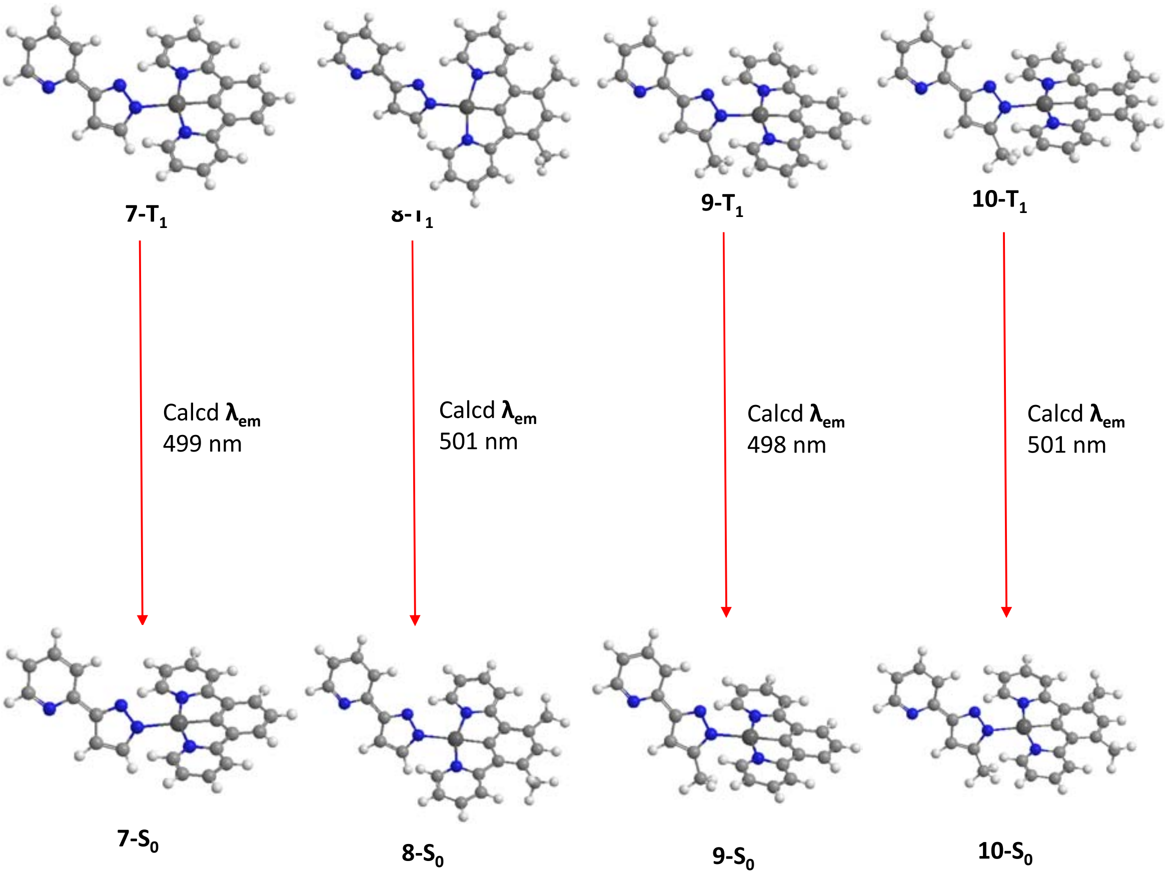

Figure S293. Optimized geometries S_0 and T_1 states of complexes **7-10**.

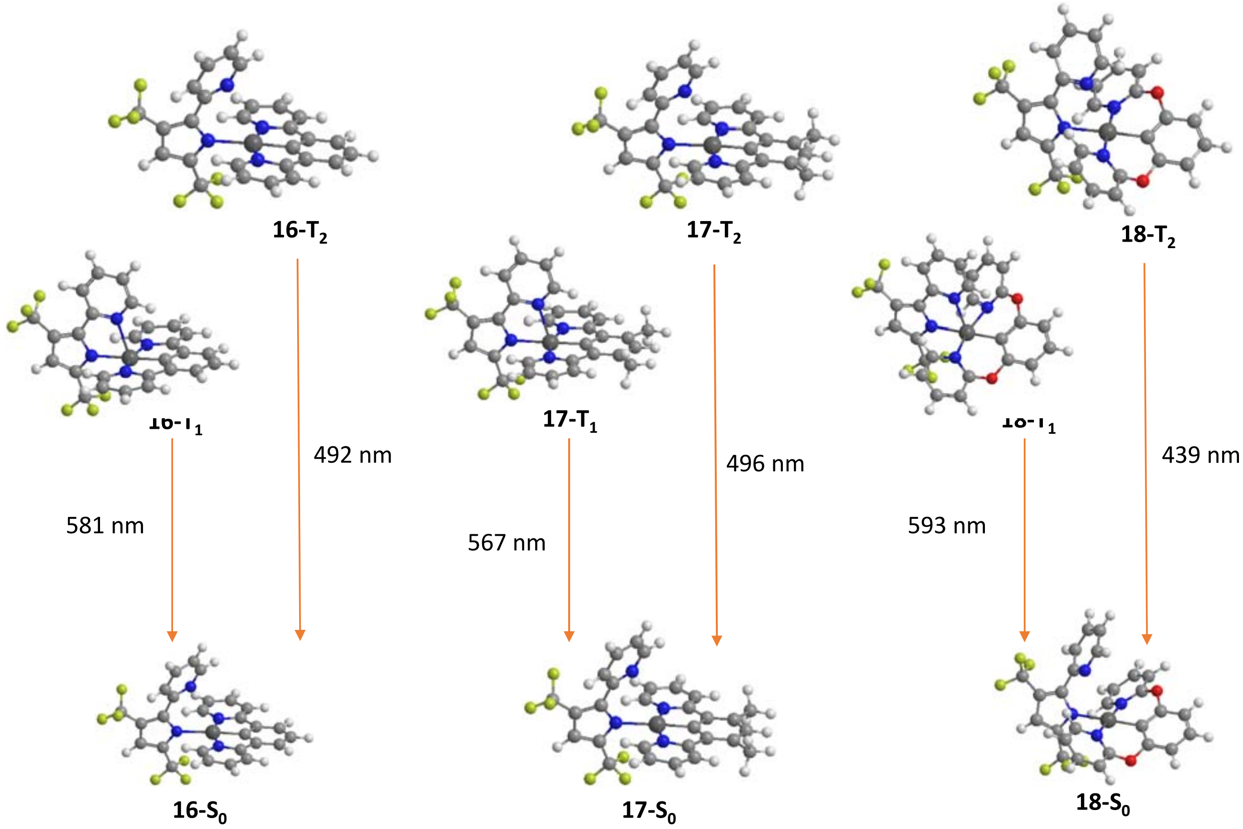

Figure S294. Optimized geometries S_0, T_1, and T_2 states of complexes **16-18**.

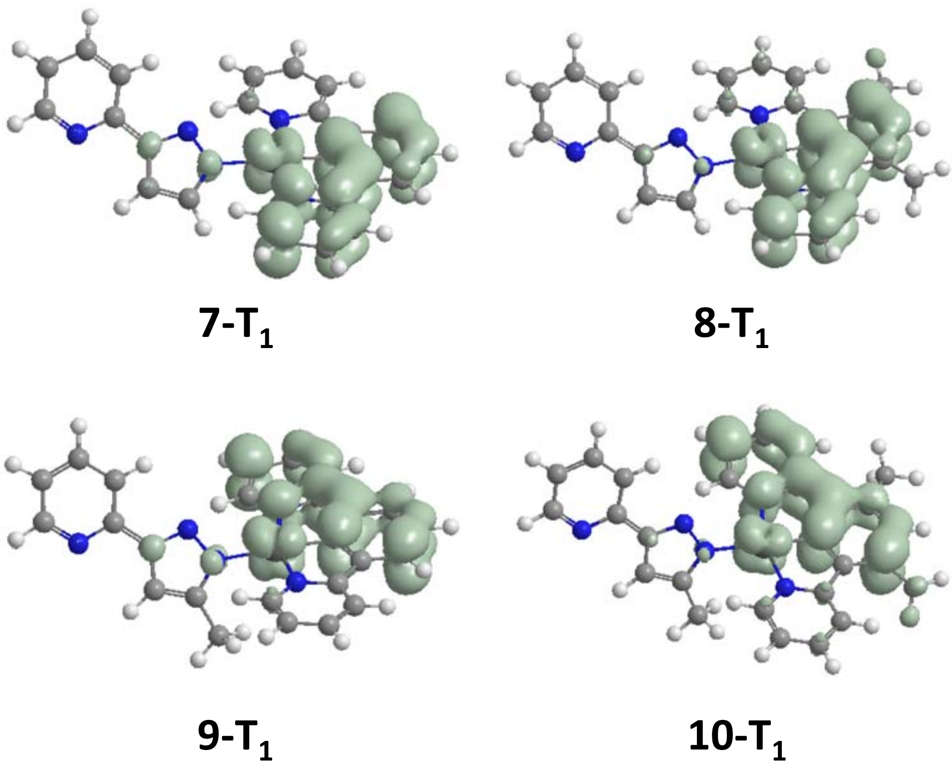

Figure S295. Spin density distribution for the optimized triplet states of complexes **7-10** (isovalue 0.004).

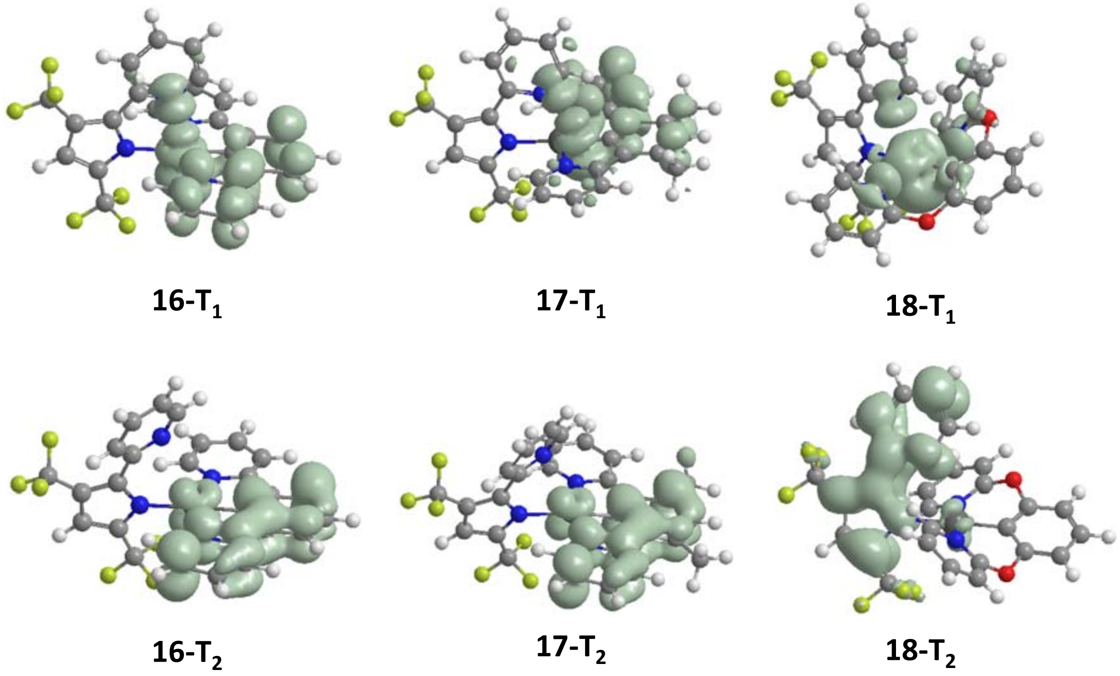

Figure S296. Spin density for the optimized triplet states of complexes **16**, **17** and **18** (isovalue 0.004).

• **References**

(1) Cárdenas, D. J.; Echavarren, A. M.; Ramírez de Arellano, M. C. Divergent Behavior of Palladium(II) and Platinum(II) in the Metalation of 1,3-Di(2-pyridyl)benzene. *Organometallics* **1999**, *18*, 3337-3341.

(2) Schulze, B.; Friebe, C.; Jäger, M.; Görls, H.; Birckner, E.; Winter, A.; Schubert, U. S. Pt^{II} Phosphors with Click-Derived 1,2,3-Triazole-Containing Tridentate Chelates. *Organometallics* **2018**, *37*, 145-155.

(3) Kim, Y. T.; Yoon, M. S. Synthesis of a Platinum-Pincer Complex and Application to Catalytic Silylcyanation. *Appl. Chem. Eng.* **2016**, *27*, 366-370.

(4) Morse, P. M.; Spencer, M. D.; Wilson, S. R.; Girolami, G. S. A Static Agostic α-CH···M Interaction Observable by NMR Spectroscopy: Synthesis of the Chromium(II) Alkyl $[Cr_2(CH_2SiMe_3)_6]^{2-}$ and Its Conversionto the Unusual "Windowpane" Bis(metallacycle) Complex $[Cr\{\kappa^2 C,C'\text{-}CH_2SiMe_2CH_2)_2]^{2-}$. *Organometallics* **1994**, *13*, 1646-1665.

(5) Blessing, R. H. *Acta Crystallogr.* **1995**, *A51*, 33. SADABS: Area-detector absorption correction; Bruker- AXS, Madison, WI, 1996.

(6) SHELXL-2016/6. Sheldrick, G. M. *Acta Cryst.* **2008**, *A64*, 112-122.

(7) (a) A. L. Spek (2002) PLATON, A Multipurpose Crystallographic Tool, Utrecht University, Utrecht, The Netherlands. (b) Yang, X.-J.; Drepper, F.; Wu, B.; Sun, W.-H.; Haehnel, W.; Janiak, C. From model compounds to protein binding: syntheses, characterizations and fluorescence studies of $[Ru^{II}(bipy)(terpy)L]^{2+}$ complexes (bipy = 2,2'-bipyridine; terpy = 2,2':6',2"-terpyridine; L = imidazole, pyrazole and derivatives, cytochrome c). *Dalton Trans.* **2005**, 256-267.

(8) (a) Lee, C.; Yang, W.; Parr, R. G. Development of the Colle-Salvetti correlation energy formula into a functional of the electron density. *Phys. Rev. B* **1988**, *37*, 785-789. (b) Becke, A. D. Density-functional exchange-energy approximation with correct asymptotic behavior. *J. Chem. Phys.* **1993**, *98*, 5648–5652. (c) Stephens, P. J.; Devlin, F. J.; Chabalowski, C. F.; Frisch, M. J. Ab Initio Calculation of Vibrational Absorption and Circular Dichroism Spectra Using Density Functional Force Fields. *J. Phys. Chem.* **1994**, *98*, 11623-11627.

(9) Grimme, S.; Antony, J.; Ehrlich, S.; Krieg, H. A consistent and accurate ab initio parametrization of density functional dispersion correction (DFT-D) for the 94 elements H-Pu. *J. Chem. Phys.* **2010**, *132*, 154104.

(10) Gaussian 09, Revision D.01, Frisch, M. J.; Trucks, G. W.; Schlegel H. B.; Scuseria, G. E.; Robb, M. A.; Cheeseman, J. R.; Scalmani, G.; Barone, V.; Mennucci, B.; Petersson, G. A.; Nakatsuji, H.; Caricato, M.; Li, X.; Hratchian, H. P.; Izmaylov, A. F.; Bloino, J.; Zheng, G.; Sonnenberg, J. L.; Hada, M.; Ehara, M.; Toyota, K.; Fukuda,

R.; Hasegawa, J.; Ishida, M.; Nakajima, T.; Honda, Y.; Kitao, O.; Nakai, H.; Vreven, T.; Montgomery, J. A.; Peralta, Jr., J. E.; Ogliaro, F.; Bearpark, M.; Heyd, J. J.; Brothers, E.; Kudin, K. N.; Staroverov, V. N.; Keith, T.; Kobayashi, R.; Normand, J.; Raghavachari, K.; Rendell, A.; Burant, J. C.; Iyengar, S. S.; Tomasi, J.; Cossi, M.; Rega, N.; S43 Millam, J. M.; Klene, M.; Knox, J. E.; Cross, J. B.; Bakken, V.; Adamo, C.; Jaramillo, J.; Gomperts, R.; Stratmann, R. E.; Yazyev, O.; Austin, A. J.; Cammi, R.; Pomelli, C.; Ochterski, J. W.; Martin, R. L.; Morokuma, K.; Zakrzewski, V. G.; Voth, G. A.; Salvador, P.; Dannenberg, J. J.; Dapprich, S.; Daniels, A. D.; Farkas, O.; Foresman, J. B.; Ortiz, J. V.; Cioslowski, J.; Fox, D. J. Gaussian, Inc., Wallingford CT, **2013**.

(11) Andrea, D.; Häußermann, U. M.; Dolg, M.; Stoll, H.; Preuss, H. Energy adjusted ab initio pseudopotentials for the second and third row transition elements. *Theor. Chim. Acta* **1990**, *77*, 123-141.

(12) Ehlers, A. W.; Bohme, M.; Dapprich, S.; Gobbi, A.; Hollwarth, A.; Jonas, V.; Kohler, K. F.; Stegmann, R.; Veldkamp, A.; Frenking, G. A set of f-polarization functions for pseudo-potential basis sets of the transition metals SC-Cu, Y-Ag and La-Au. *Chem. Phys. Lett.* **1993**, *208*, 111-114.

(13) (a) Hehre, W. J.; Ditchfield, R.; Pople, J. A. Self-Consistent Molecular Orbital Methods. XII. Further Extensions of Gaussian-Type Basis Sets for Use in Molecular Orbital Studies of Organic Molecules. *J. Chem. Phys.* **1972**, *56*, 2257-2261. (b) Francl, M. M.; Pietro, W. J.; Hehre, W. J.; Binkley, J. S.; Gordon, M. S.; DeFrees, D. J.; Pople, J. A. Self-consistent molecular orbital methods. XXIII. A polarization-type basis set for second-row elements. *J. Chem. Phys.* **1982**, *77*, 3654-3665.

(14) Marenich, A. V.; Cramer, C. J.; Truhlar, D. G. Universal Solvation Model Based on Solute Electron Density and on a Continuum Model of the Solvent Defined by the Bulk Dielectric Constant and Atomic Surface Tensions. *J. Phys. Chem. B* **2009**, *113*, 6378-6396.

(15) O'Boyle, N. M.; Tenderholt, A. L.; Langner, K. M. cclib: A Library for Package-Independent Computational Chemistry Algorithms. *J. Comput. Chem.* **2008**, *29*, 839-845.

(16) AIMAll (Version 19.10.12), Todd A. Keith, TK Gristmill Software, Overland Park KS, USA, **2019**.
